# Association between Birth Interval and Cardiovascular Outcomes at 30 Years of Age: A Prospective Cohort Study from Brazil

**DOI:** 10.1371/journal.pone.0149054

**Published:** 2016-02-18

**Authors:** D. Devakumar, P. C. Hallal, B. L. Horta, F. C. Barros, J. C. K. Wells

**Affiliations:** 1 Institute for Global Health, University College London, London, United Kingdom; 2 Federal University of Pelotas, Post-Graduate Programme in Epidemiology, Pelotas, Brazil; 3 Institute of Child Health, University College London, London, United Kingdom; Federal University of Pelotas, BRAZIL

## Abstract

**Background:**

Birth interval is an important and potentially modifiable factor that is associated with child health. Whether an association exists with longer-term outcomes in adults is less well known.

**Methods:**

Using the 1982 Pelotas (Brazil) Birth Cohort Study, the association of birth interval with markers of cardiovascular health at 30 years of age was examined. Multivariable linear regression was used with birth interval as a continuous variable and categorical variable, and effect modification by gender was explored.

**Results:**

Birth interval and cardiovascular data were present for 2,239 individuals. With birth interval as a continuous variable, no association was found but stratification by gender tended to show stronger associations for girls. When compared to birth intervals of <18 months, as binary variable, longer intervals were associated with increases in height (1.6 cm; 95% CI: 0.5, 2.8) and lean mass (1.7 kg; 95% CI: 0.2, 3.2). No difference was seen with other cardiovascular outcomes.

**Conclusions:**

An association was generally not found between birth interval and cardiovascular outcomes at 30 years of age, though some evidence existed for differences between males and females and for an association with height and lean mass for birth intervals of 18 months and longer.

## Introduction

According to the ‘developmental origins of adult disease’ hypothesis, patterns of fetal growth and development shape susceptibility to chronic non-communicable diseases in adult life. Early work focused on the long-term health consequences of low birth weight, which was assumed to indicate fetal malnutrition. Increased non-communicable disease risk following fetal exposure to maternal starvation during the "Dutch hunger winter" seemed to support this hypothesis.[1] However, subsequent work has raised doubts on this, for example maternal circulating nutrients during pregnancy did not predict birth weight.[2, 3] Furthermore, the association between birth weight and later disease risk held broadly across the entire range of birth weight, [4, 5] refuting the notion that fetal malnutrition must be the key stress.

In fact, the dominant environmental influence on the fetus is maternal phenotype, which has multiple dimensions of variability. These include relatively stable traits, such as maternal height and lean mass, and more labile traits such as physical activity profile, micronutrient status, glycemic control and adiposity.[6] The simplest marker of nutritional investment in the fetus is birth weight, which has been linked in many studies with subsequent disease risk. [7, 8] However, birth weight is a composite of many different traits, and is difficult to interpret as an exposure.[9]

An alternative marker of fetal nutritional investment, potentially marking maternal supply rather than fetal acquisition, is the duration of the inter-birth interval. From an evolutionary perspective, mothers must acquire resources from the environment in order to invest in offspring. A shorter period between births may reduce the ability of the mother to replenish her reserves adequately for this purpose or provide competition for her resources. This parameter merits study, because unlike stable maternal traits such as stature, the length of a birth interval is potentially amenable to public health interventions.[6]

Most research on birth interval has tended to focus on its association with perinatal outcomes. Short inter-pregnancy intervals of less than six months from birth to the next conception, including after an aborted pregnancy, [10] are associated with lower birth weight, preterm birth and neonatal mortality. [11, 12] They are also associated with increased maternal mortality and morbidity [13] and, in an assessment of data from 17 developing countries, an inverse association with child mortality was found. [12]

Previous analysis of data from Pelotas, Brazil showed that children born after birth intervals of <18 months were at greater risk of neonatal mortality, of being born with low birthweight and poor growth in childhood. At 19 months of age, children born after <18 month birth interval compared to >71 months had a 0.49 z score lower height-for-age (~1.5 cm) and 0.41 lower weight-for-age (~0.5 kg). Restricting the analysis to children with known gestational age did not alter the outcomes. [14]

There is evidence that birth spacing is associated with childhood nutritional status and child malnutrition,[12] for example a study from El Salvador showed an increased odds of stunting for intervals of less than 36 months. [15] However, little attention has been directed to the question of whether short inter-birth intervals might, consistent with the ‘developmental origins’ hypothesis, increase susceptibility to chronic non-communicable diseases later in life. To date, there is little evidence on the magnitude of the association of inter-pregnancy interval on cardiovascular health of the offspring, or the mechanisms through which this may operate. Here we investigate the question of whether increasing birth interval leads to a change in cardiovascular risks factors in adulthood, using a prospective 30-year cohort from Brazil. We hypothesize that longer birth intervals lead to improved cardiovascular health in adulthood. This approach offers a novel perspective on the developmental origins of health and disease hypothesis, as it disentangles variability in maternal investment from overt maternal malnutrition.

## Methods

The 1982 Pelotas (Brazil) Birth Cohort Study with follow-up at 30 years was used for this analysis (data are in S1 File). The methods in the cohort have been described previously, [16] and full details of the 30-year follow are shown elsewhere.[17] Briefly, pregnant women who were attending all maternity hospitals in Pelotas, Brazil and whose family lived in the urban area of the city, were assessed and their offspring followed up at birth, 1, 2, 4, 15, 18, 19 and 23 years of age. Women were interviewed, including socioeconomic details and education, and anthropometry measured. The cohort included 5914 live born infants.

The 30-year data collection was conducted from June 2012 to February 2013. Participants visited a research centre and answered a questionnaire, had physiological examinations and blood samples taken. Standing height was measured using a CMS stadiometer, accurate to 0.1 cm. Body composition was estimated with dual-energy x-ray absorptiometry (DXA Lunar Prodigy). Visceral and subcutaneous abdominal fat and carotid intima-media thickness were measured using ultrasound (Toshiba Xario). Blood samples were collected by the research team, stored at the research centre and later analyzed commercially.

The study was approved by the Research Ethics Committee of the Faculty of Medicine, Federal University of Pelotas (affiliated with the Brazilian National Council of Research Ethics) and all subjects provided written informed consent.

### Analysis

The exposure variable was birth interval in months (defined as the time period from the birth of the previous sibling to the beginning of the pregnancy of the index child) by maternal recall, treated both as a continuous and a categorical variable. For comparison purposes, we chose the categories previously used in the same cohort: <18 months, 18 to <24 months, 24 to <36 months, 36 to <48 months, 48 to <71 months, ≥71 months. [14] This gave an even spread of data. A binary birth interval variable (<18 months and ≥18 months) was also created to enable our results to be compared to other studies that use this categorization and to enhance statistical power.

For the outcome measures we defined cardiovascular risk factors as body composition (fat mass by DEXA, fat-free mass, visceral fat and subcutaneous fat), body mass index (BMI), blood pressure, carotid arterial thickness, lipid profile, glucose and insulin concentrations at 30 years of age. BMI was calculated as weight (kg) divided by height (m) squared.

Potential confounding variables were decided *a priori* based on previous knowledge. The variables included were maternal age, education and BMI at the beginning of pregnancy, family income at birth and birth order. [18, 19] The associations between exposure and outcome variables and confounding variables are shown in Tables A and B in S2 File.

Univariable and multi-variable linear regression analyses were performed with birth interval (for individuals of birth order two or higher) as the independent variable and cardiovascular risk factors as the outcome variables. The primary analyses included birth interval as a continuous variable. To investigate potential threshold effects, we then included birth interval as a categorical variable. We also stratified by child sex. Model assumptions were tested for linearity by plotting residuals against each covariate and for normality by examining a kernel density plot of residuals. Non-normal residual distributions were seen for glucose and triglyceride values and these were transformed using natural logarithms. Fat-free mass had a binomial distribution, so was adjusted with robust standard errors using the Huber and White method.[20] Analysis was conducted in Stata, version 12 (StataCorp, College Station, TX, USA).

## Results

Offspring were seen at a mean age of 30.2 years. The follow-up rate was 68.1% of the original cohort (3,701 plus 325 known to have died). The main cohort characteristics have been described previously. [17] Birth interval data were present for 3671 individuals (49.3% female), 2,239 (61.0%) of whom had complete cardiovascular data at 30 years. Maternal, fetal and 30-year characteristics stratified by birth interval are shown in Table 1 (with stratification by offspring sex for outcome variables in Table C in S2 File).

**Table 1:** Maternal and Offspring Characteristics at Birth and 30 years, Stratified by Birth Interval, for the 1982 Pelotas (Brazil) Birth Cohort.

	Birth interval					
	<18 months Mean (SD) n = 365	18 to <24 months Mean (SD) n = 278	24 to <36 months Mean (SD) n = 469	36 to <48 months Mean (SD) n = 323	48 to <71 months Mean (SD) n = 414	>71 months Mean (SD) n = 390
**Maternal**						
Age at delivery (years)	25.1 (5.5)	26.0 (5.3)	26.6 (5.3)	28.0 (5.2)	29.2 (5.4)	33.2 (5.0)
Education (years)	6.1 (3.9)	6.4 (4.3)	6.3 (4.1)	6.5 (4.4)	5.9 (4.0)	5.3 (3.8)
Family income (multiples of 1982 minimum wage)	2.1 (1.0)	2.3 (1.1)	2.3 (1.0)	2.4 (1.1)	2.3 (1.0)	2.4 (1.0)
Height (cm)	155.8 (5.7)	157.0 (6.5)	156.3 (5.9)	156.3 (6.0)	156.3 (5.9)	156.5 (5.5)
BMI at beginning of pregnancy (kg/m^2^)	22.4 (3.6)	22.8 (3.7)	23.0 (3.8)	23.3 (3.5)	23.8 (3.8)	25.1 (4.5)
**Fetus/child**						
Sex (% males)	47.1	49.6	47.8	51.1	48.1	47.7
Birthweight (g)	3171 (514)	3203 (530)	3283 (535)	3333 (510)	3287 (510)	3267 (513)
Gestation (weeks)	39.4 (1.98)	39.2 (1.9)	39.4 (1.8)	39.5 (1.7)	39.2 (1.7)	39.3 (1.8)
Birth order (median, IQR)	3 (2, 10)	3 (2, 14)	3 (2, 10)	3 (2, 11)	3 (2, 11)	3 (2, 11)
**30 years**						
Height (cm)	166.6 (9.1)	167.8 (8.8)	167.8 (9.3)	168.2 (9.0)	167.3 (9.1)	167.7 (8.9)
Weight (kg)	74.2 (17.8)	74.8 (17.7)	75.1 (16.9)	75.7 (17.8)	74.8 (16.1)	77.2 (18.4)
Body mass index (kg/m^2^)	26.6 (5.7)	26.4 (5.3)	26.5 (5.1)	26.6 (5.4)	26.6 (5.2)	27.4 (6.1)
Fat-free mass (dexa)	49.6 (10.9)	50.1 (11.1)	50.6 (11.4)	51.2 (11.2)	50.5 (11.2)	50.2 (11.2)
Fat mass (kg)	22.8 (11.2)	23.1 (10.9)	23.7 (11.1)	23.4 (10.9)	23.9 (11.0)	25.8 (12.0)
Visceral fat (cm) (median, inter-quartile range)	5.5 (4.4, 7.0)	5.6 (4.5, 7.1)	5.4 (4.4, 7.1)	5.7 (4.4, 7.2)	5.7 (4.4, 7.1)	5.6 (4.4, 7.3)
Subcutaneous fat (cm) (median, inter-quartile range)	1.9 (1.4, 2.8)	2.0 (1.4, 2.7)	2.0 (1.4, 2.8)	2.0 (1.3, 2.9)	2.1 (1.4, 2.9)	2.2 (1.5, 3.0)
Systolic blood pressure (mmHg)	120.6 (13.4)	120.5 (14.3)	121.1 (13.5)	120.9 (13.2)	120.8 (13.4)	120.4 (13.0)
Diastolic blood pressure (mmHg)	74.9 (9.0)	74.5 (9.1)	75.1 (9.0)	74.3 (8.9)	75.6 (9.5)	75.3 (9.3)
Left carotid thickness (mm)	0.58 (0.03)	0.58 (0.02)	0.58 (0.02)	0.58 (0.02)	0.58 (0.03)	0.58 (0.02)
Right carotid thickness (mm)	0.58 (0.03)	0.58 (0.01)	0.58 (0.02)	0.58 (0.02)	0.58 (0.02)	0.58 (0.02)
Glucose (mg/dL)	88.6 (24.2)	89.2 (26.1)	89.7 (26.1)	90.5 (32.5)	87.8 (17.7)	90.8 (30.9)
Cholesterol (mmol/L)	189.2 (39.2)	185.7 (33.6)	191.3 (39.4)	189.3 (38.7)	190.2 (38.0)	193.0 (45.7)
High density lipoprotein (mmol/L)	58.6 (14.5)	59.0 (14.6)	58.2 (13.9)	58.1 (13.8)	58.3 (13.3)	58.7 (14.7)
Low density lipoprotein (mmol/L)	108.5 (31.0)	105.2 (26.3)	109.2 (28.1)	107.7 (29.0)	108.5 (30.2)	110.3 (29.1)
Triglycerides (mmol/L) (median, inter-quartile range)	88.5 (66, 135)	87 (64, 128)	93 (66, 144)	94 (64, 144)	100 (68, 148)	94 (68, 135)

Multivariable regression analyses found no association between birth interval and cardiovascular risk factors. Inclusion of fat-free mass in the models to adjust for stature made little difference to the outcomes (data not shown). Stratification by gender tended to show stronger associations with birth interval in females than males for anthropometry, body composition and cholesterol (Table 2) but there was evidence of an interaction only for fat-free mass, visceral fat, low-density lipoprotein and the natural logarithm of triglycerides.

**Table 2:** Regression analysis results with birth interval as a continuous variable.

Offspring traits at 30 years	Univariable (95% CI)	Multivariable (95% CI)	Multi-variable stratification by offspring gender
Height (cm)	0.005 (-0.006, 0.016)	0.009 (-0.004, 0.023)	Males: -0.003 (-0.017, 0.012)
			Females: 0.011 (-0.002, 0.024)
Weight (kg)	0.028 (0.008, 0.049)	0.020 (-0.005, 0.046)	Males: -0.007 (-0.043, 0.028)
			Females: 0.035 (0.003, 0.068)
Body mass index (kg/m^2^)	0.009 (0.003, 0.016)	0.005 (-0.003, 0.013)	Males: -0.001 (-0.011, 0.010)
			Females: 0.010 (-0.002, 0.022)
Fat-free mass (kg)*	0.003 (-0.009, 0.016)	0.012 (-0.005, 0.029)	Males: -0.007 (-0.023, 0.008)
			Females: 0.014 (0.000, 0.027)
Fat mass (kg)	0.029 (0.015, 0.043)	0.013 (-0.004, 0.029)	Males: 0.012 (-0.009, 0.034)
			Females: 0.019 (-0.004, 0.042)
Visceral fat (cm) *	0.001 (-0.001, 0.004)	0.002 (-0.002, 0.005)	Males: -0.002 (-0.006, 0.003)
			Females: 0.004 (0.000, 0.007)
Subcutaneous fat (cm)	0.002 (0.001, 0.004)	0.001 (-0.001, 0.002)	Males: 0.000 (-0.002, 0.002)
			Females: 0.001 (-0.001, 0.003)
Systolic blood pressure (mmHg)	-0.001 (-0.017, 0.015)	0.009 (-0.011, 0.030)	Males: -0.014 (-0.041, 0.012)
			Females: 0.019 (-0.005, 0.043)
Diastolic blood pressure (mmHg)	0.005 (-0.006, 0.016)	0.010 (-0.004, 0.024)	Males: 0.013 (-0.018, 0.023)
			Females: 0.014 (-0.005, 0.033)
Left carotid thickness (mm) multiplied by 1000	0.001 (-0.031, 0.032)	-0.007 (-0.047, 0.033)	Males: -0.043 (-0.103, 0.017)
			Females: 0.021 (-0.030, 0.073)
Right carotid thickness (mm) multiplied by 1000	-0.011 (-0.040, 0.017)	-0.002 (-0.036, 0.031)	Males: -0.028 (-0.082, 0.026)
			Females: 0.012 (-0.027, 0.052)
Ln Glucose (mg/dL)	0.000 (-0.000, 0.000)	0.000 (-0.000, 0.001)	Males: 0.000 (-0.000, 0.001)
			Females: 0.000 (-0.000, 0.001)
Cholesterol (mmol/L)	0.008 (-0.039, 0.055)	0.006 (-0.055, 0.067)	Males: -0.052(-0.148, 0.044)
			Females: 0.056 (-0.021, 0.132)
High density lipoprotein (mmol/L)	-0.006 (-0.023, 0.011)	-0.026 (-0.047, -0.004)	Males: -0.031 (-0.059, -0.003)
			Females: -0.015 (-0.045, 0.014)
Low density lipoprotein * (mmol/L)	0.006 (-0.029, 0.040)	0.010 (-0.034, 0.054)	Males: -0.049 (-0.113, 0.014)
			Females: 0.062 (0.001, 0.123)
Ln Triglycerides * (mmol/L)	0.000 (-0.000, 0.001)	0.000 (-0.000, 0.001)	Males: -0.000 (-0.002, 0.001)
			Females: 0.001 (-0.000, 0.002)

*Interaction factor for offspring gender <0.05 (fat-free mass *P* = 0.04, visceral fat *P* = 0.02, low density lipoprotein *P* = 0.02, Ln Triglycerides *P* = 0.01)

When considering birth interval as a categorical variable, compared to <18 months, longer intervals were associated with increases in height of approximately 1.6 cm and with increases in lean mass which were mostly just below the threshold of significance (Table 3). No difference was seen in other cardiovascular outcomes. The results including birth interval as binary variable are shown in Table 4. Similarly only offspring height (1.6 cm (95% CI: 0.5, 2.8)) and lean mass (1.7 kg (95% CI: 0.2, 3.2)) showed an association.

**Table 3:** Multivariable regression results with birth interval as a categorical variable.

Offspring traits at 30 years	Birth interval					
	<18 months (95% CI)	18 to <24 months (95% CI)	24 to <36 months (95% CI)	36 to <48 months (95% CI)	48 to <71 months (95% CI)	≥71 months (95% CI)
Height (cm)	Ref	1.863 (0.291, 3.435)	1.720 (0.333, 3.107)	1.865 (0.334, 3.395)	1.103 (-0.368, 2.574)	1.612 (0.033, 3.190)
Weight (kg)	Ref	0.771 (-2.200, 3.741)	1.055 (-1.572, 3.682)	1.030 (-1.875, 3.935)	0.332 (-2.455, 3.118)	2.320 (-0.668, 5.307)
Body mass index (kg/m^2^)	Ref	-0.346 (-1.265, 0.574)	-0.228 (-1.041, 0.585)	-0.294 (-1.193, 0.605)	-0.222 (-1.085, 0.640)	0.364 (-0.560, 1.289)
Fat-free mass (kg)	Ref	1.479 (-0.495, 3.452)	1.619 (-0.139, 3.377)	2.334 (0.397, 4.270)	1.536 (-0.308, 3.380)	1.728 (-0.243, 3.698)
Fat mass (kg)	Ref	-0.757 (-2.678, 1.164)	0.101 (-1.593, 1.794)	-0.763 (-2.638, 1.112)	-0.119 (-1.912, 1.675)	1.019 (-0.911, 2.949)
Visceral fat (cm)	Ref	0.128 (-0.246, 0.502)	0.051 (-0.279, 0.381)	0.186 (-0.179, 0.552)	0.186 (-0.164, 0.537)	0.207 (-0.168, 0.582)
Subcutaneous fat (cm)	Ref	-0.080 (-0.267, 0.108)	-0.034 (-0.199, 0.131)	-0.112 (-0.295, 0.071)	-0.108 (-0.283, 0.068)	0.020 (-0.168, 0.207)
Systolic blood pressure (mmHg)	Ref	1.168 (-1.190, 3.526)	1.086 (-0.995, 3.167)	0.920 (-1.378, 3.218)	0.926 (-1.284, 3.136)	0.996 (-1.373, 3.364)
Diastolic blood pressure (mmHg)	Ref	0.072 (-1.539, 1.684)	0.873 (-0.549, 2.295)	-0.128 (-1.698, 1.442)	1.033 (-0.478, 2.543)	1.254 (-0.364, 2.873)
Left carotid thickness (mm) multiplied by 1000	Ref	-0.696 (-5.140, 3.749)	0.526 (-3.389, 4.440)	1.269 (-3.082, 5.619)	1.249 (-2.951, 5.449)	-0.832 (-5.430, 3.765)
Right carotid thickness (mm) multiplied by 1000	Ref	-3.167 (-6.935, 0.601)	-1.316 (-4.623, 1.992)	1.643 (-2.000, 5.285)	-3.434 (-6.979, 0.112)	-1.480 (-5.356, 2.395)
Ln glucose (mg/dL)	Ref	0.003 (-0.032, 0.039)	0.011 (-0.021, 0.042)	0.019 (-0.016, 0.054)	0.007 (-0.027, 0.040)	0.030 (-0.006, 0.065)
Cholesterol (mmol/L)	Ref	-5.609 (-12.539, 1.320)	2.262 (-3.907, 8.431)	-1.014 (-7.850, 5.822)	1.488 (-5.046, 8.022)	3.754 (-3.229, 10.738)
High density lipoprotein (mmol/L)	Ref	-0.265 (-2.740, 2.211)	-1.225 (-3.429, 0.979)	-1.261 (-3.703, 1.181)	-1.558 (-3.893, 0.776)	-1.801 (-4.296, 0.694)
Low density lipoprotein (mmol/L)	Ref	-3.869 (-8.888, 1.150)	1.754 (-2.714, 6.222)	-0.581 (-5.533, 4.370)	1.071 (-3.661, 5.804)	2.883 (-2.175, 7.941)
Ln triglycerides (mmol/L)	Ref	-0.055 (-0.156, 0.046)	0.040 (-0.050, 0.130)	0.008 (-0.092, 0.108)	0.083 (-0.012, 0.179)	0.057 (-0.045, 0.159)

**Table 4:** Multivariable regression results with birth interval as a binary variable.

Offspring traits at 30 years	Birth interval	
	<18 months	≥18 months (95% CI)
Height (cm)	Ref	1.636 (0.454, 2.817)
Weight (kg)	Ref	0.998 (-1.240, 3.235)
Body mass index (kg/m^2^)	Ref	-0.192 (-0.885, 0.501)
Fat-free mass (kg)	Ref	1.717 (0.242, 3.193)
Fat mass (kg)	Ref	-0.154 (-1.597, 1.290)
Visceral fat (cm)	Ref	0.136 (-0.145, 0.417)
Subcutaneous fat (cm)	Ref	-0.066 (-0.206, 0.075)
Systolic blood pressure (mmHg)	Ref	1.028 (-0.746, 2.802)
Diastolic blood pressure (mmHg)	Ref	0.614 (-0.599, 1.828)
Left carotid thickness (mm) multiplied by 1000	Ref	0.432 (-2.992, 3.785)
Right carotid thickness (mm) multiplied by 1000	Ref	-1.559 (-4.399, 1.281)
Ln glucose (mg/dL)	Ref	0.012 (-0.015, 0.039)
Cholesterol (mmol/L)	Ref	0.170 (-5.083, 5.424)
High density lipoprotein (mmol/L)	Ref	-1.181 (-3.054, 0.693)
Low density lipoprotein (mmol/L)	Ref	0.242 (-3.563, 4.047)
Ln triglycerides (mmol/L)	Ref	0.027 (-0.050, 0.103)

## Discussion

This study performed an analysis of prospectively collected cohort data and sought to investigate whether a lasting association exists between the length of the birth interval and a range of cardiovascular outcomes at 30 years of age. Overall an association was not found, though potentially differences did exist between males and females, and compared to birth intervals of <18 months, longer birth intervals resulted in increases in adult height of 1–2 cm, with some evidence of increases in lean mass. This association with height is potentially important due to its association with adult health and income. [21]

WHO recommends birth intervals of greater than 24 months.[22] Changing a birth interval is a modifiable risk factor in adulthood that can affect offspring health. In this sense it is associated with ‘liquid capital’ rather than characteristics that are set early in the mother’s life.[6] This makes birth interval particularly interesting from a public health perspective as it is a factor that can potentially be altered to improve child health.

Previous research has shown an association of birth interval with birth weight. A meta-analysis of five cohort studies (including three from Pelotas, Brazil and one each from the Philippines and Zimbabwe) has shown that birth intervals of less than 18 months and longer than 60 months increased the risk of being born small for gestational age, preterm and infant mortality. [23] Other evidence has shown that the greatest risk for low birthweight is with shorter intervals (OR 1.44 (95% CI: 1.30, 1.61) for inter-pregnancy interval of <6 months and 1.12 (95% CI: 1.08, 1.17) for 6–11 months).[24]

Long-term effects are mediated via in-utero and postnatal growth. The mechanisms by which these arise are through maternal folate depletion, sub-optimal breastfeeding or when breastfeeding overlaps with a pregnancy, transmission of infection both vertically and from siblings and other forms of sibling competition.[18] Though potentially important at higher parity, there is limited evidence for the maternal depletion model,[25] however, folate depletion is considered important. [18] The association between birth spacing and child nutritional status shows inconsistent findings. A systematic review in developing countries found that birth intervals are associated with nutritional status in some populations. Of the 22 papers (that include 52 studies, only 5 of which were prospective cohorts) they assess for childhood nutritional status, half show a positive association and half showed no association, with two studies showing mixed results. Five studies assessed birthweight as mediating factor. Four of these still found an association with child nutritional status after controlling for birthweight, indicating both in-utero and childhood mechanisms.[19]

Why might no relationship be seen in cardiovascular outcomes in adults if birth interval is associated with childhood outcomes? There are several possible explanations. Firstly there may truly be no association with adult outcomes. Compensatory mechanisms in childhood could lead to phenotypes and physiology becoming similar within the population over time, erasing effects apparent at earlier ages.

Second, thirty years may not be the best age to evaluate variability in all cardiovascular outcomes. Overt disease tends to occur later in middle-age following decades of unhealthy lifestyle, and it may be that young adults might not reveal deleterious consequences of poor fetal development.

Third, removing firstborns from the analysis was necessary but this might weaken associations between markers of fetal nutrition and adult cardiovascular health. The sample size, while large, would have been insufficient to detect small effects. Studies have shown that firstborns are at elevated risk of chronic diseases in adulthood, in part due to their low birth weight. Research using the same 1982 Pelotas cohort showed increased cardiovascular risk in firstborns, potentially mediated by having a lower birthweight and evidence from a subsequent cohort in Pelotas at 13 years of age, showed an association between being born first and height and blood pressure. [26, 27]

Fourth, the inter-birth interval may not index aspects of maternal investment that matter for adult health. The evolutionary ‘maternal capital’ hypothesis assumes that mothers with greater nutritional resources can invest more in their offspring during fetal life, increasing their homeostatic capacity in adult life. However, there are numerous dimensions of maternal capital, some of which may be more influential than others on fetal development.[6] Although it is widely expected that maternal diet during pregnancy should predict offspring outcomes, few studies have supported this hypothesis. For example, circulating maternal nutrients were not correlated with birth weight among UK mothers.[2, 3] In contrast, maternal lean mass has been associated with birth weight.[28, 29] Likewise, maternal BMI prior to conception has stronger associations with birth weight than gestational weight gain. [30] Indices of stable ‘somatic capital’ may therefore be more important for maternal investment in the offspring than indices of labile ‘liquid capital’.

Finally, it may be that birth interval is more important in populations that have a greater proportion of under-nourished mothers. The mother’s BMI in our sample was within the normal range for all birth interval categories. In this population, the majority of mothers may have had ample time to replenish any nutrient reserves, resulting in our null findings. We should therefore be cautious about generalizing our results to other developing countries where the mothers have poorer nutrition.

### Strengths and limitations

The main strength of this study was the use of a prospective, 30-year cohort study with high follow up rates. The cohort is also one of the largest of its kind and covered >99% of births in the city in one year, so is representative of its location. [17] Loss to follow-up was slightly higher amongst the poorest and richest participants, but overall attrition bias was considered unimportant.[17]

It is possible that residual confounding exists. Contraception use for example was unknown but a later cohort in 1986 showed that contraception was being used by all women who wanted it. [14] Other factors may also be important that affect the nutritional status and potentially cardiovascular outcomes of the child. An example would be information about subsequent birth interval, which would influence the nutritional status of the index child.

### Implications for future policy and research

Calls have been made to encourage increases in birth intervals to be incorporated into international policy. [22, 31] An analysis of birth spacing desire from demographic health surveys in low and middle-income countries shows that this is the most common reason for family planning amongst young women. [32] An evidence base exists that shows an association between birth intervals and neonatal, and some child and maternal outcomes. While still of importance, our results however show limited improvements in cardiovascular risk factors at 30 years of age.

## Conclusions

While short-term advantages in health can be gained by increasing birth intervals to an optimum length, an association was generally not found between birth interval and cardiovascular outcomes at 30 years of age, though some evidence existed for differences between boys and girls and for an association with height and lean mass for birth intervals of 18 months and longer.

## Supporting Information

S1 File Supplementary file containing the data. (PDF)

S2 File Regression coefficients (95% CI) for birth interval, as a continuous outcome variable, and confounding variables (Table A). Regression coefficients (95% CI) for cardiovascular risk factors at age 30 years and confounding variables (Table B). Maternal and Offspring Characteristics averages (mean or median) at 30 years, Stratified by Birth Interval and by gender (Table C).
(DOCX)

## Abbreviations

BMI: body mass index
CI: Confidence interval
