# Supplementary material for: Association between Birth Interval and Cardiovascular Outcomes at 30 Years of Age: A Prospective Cohort Study from Brazil
Source: PLoS One. 2016 Feb 18;11(2):e0149054. doi: 10.1371/journal.pone.0149054 (PMC4758625; doi:10.1371/journal.pone.0149054)
Supplement: S1 File — (PDF) [file pone.0149054.s001.pdf]

pescmae<br>mat education delivery	prenda<br>min wage	pfumomae<br>mat smoking	paltmae<br>mat ht	pgesta<br>birth order	pidgest<br>gest age	ppn<br>BW	psex<br>sex	pint<br>birth interval	dmgtotdxa<br>fat mass DXA	daltura2012<br>ht 30y	dpeso<br>wt 30y	dsysmed<br>BP sys 30y	ddiamed<br>BP dias 30y	dglicose<br>glucose 30y	dcolesterol<br>choles 30y	dhdl<br>HDL 30y	dldl<br>LDL 30y	dtrig<br>Trigly 30y	dECMICE<br>carotid L	dECMICD<br>carotid R
7	1.1-3	15 + tod	1.62	3		1520	mascul	35	6477.623	172.55	54.6	110	73.5	80	165	66	90	42	0.578	
9	3.1-6	n„o	1.55	1		3250	masculin		18865.69	169	78.1	136.5	85.5	91	248	67	160	161		0.5772
4	1.1-3	n„o	1.54	6	40	3520	feminir	14	35389.24	153.05	77.9	110.5	67	55	160	61	83	59	0.5786	0.5774
5	3.1-6	15 + tod	1.56	7	38	1200	feminir	53	18268.63	160.65	58.5	120	70.5	80	206	60	130	62	0.578	0.5671
5	1 ou -	15 + tod	1.57	2	40	3500	mascul	28	20009.91	174.75	82.5	140.5	80	89	174	51	112	75	0.5673	0.5669
6	1.1-3	n„o	1.57	3	40	3560	mascul	36												
4	3.1-6	n„o	1.51	2	38	3440	mascul	46	6253.711	170.7	64.8	133	66	95	100	42	41	78	0.5767	0.5802
12	6.1-10	1-14 par	1.74	1	39	3320	masculin			192.95	92.9	148.5	82.5	104	167	64	84	157	0.5786	0.5786
13	6.1-10	n„o		1	40	3730	feminino		17363.62	170.4	55.5	99	67	88	176	64	99	51		
6	1.1-3	1-14 par	1.62	1		3520	masculin		32300.78	187.85	102.1	136.5	78	106	167	43	76	300	0.5784	0.5816
0	1 ou -	n„o	1.59	6	41	3000	feminir	102	18311.08	171.8	61.4	112	71.5	110	185	72	94	162	0.5789	0.5728
3	1 ou -	n„o	1.64	3	39	3650	mascul	52	22825.01	180.25	81.8	132	83	97	189	60	104	134	0.5802	0.5771
4	1 ou -	1-14 tod	1.51	4		1510	feminir	19												
5	6.1-10	n„o	1.6	1	41	4440	feminino													
0	1.1-3	n„o	1.57	5	40	4230	feminir	24	22984.97	160.8	61.7	108.5	71.5	73	186	72	105	43	0.5786	0.5787
5	1.1-3	n„o	1.56	2	41	3570	feminir	28	27024.6	161.1	67.4	106	66.5	115	173	41	100	179	0.5764	0.5856
3	1.1-3	1-14 tod	1.57	1	41	3680	feminino													
0	1 ou -	n„o	1.4	1		2650	feminino		19838.37	140.6	54.8	114	77.5	113	226	61	150	85	0.5879	0.5822
16	10	n„o	1.58	1	36	2900	masculin		13130.95	179.5	79.1	145	70	99	185	68	104	105	0.5781	0.587
8	1 ou -	1-14 tod	1.56	4	41	3120	mascul	36												
4	3.1-6	n„o	1.47	1		2640	feminino		44097.16	155.9	94.1	119.5	78	92	259	49	167	289	0.5797	0.5969
5	3.1-6	15 + tod		2	40	3000	mascul	45	9078.025	167.3	59.6	126.5	74	63	158	34	115	61		0.592
5	6.1-10	n„o	1.59	2	40	3250	feminir	25	17787.76	160.1	52	119	83.5	82	173	54	104	67	0.57	0.578
4	1.1-3	n„o	1.62	2	40	3450	mascul	42	20372.38	174.2	76.6	136.5	78.5	81	162	58	90	39	0.578	0.5801
0	1.1-3	n„o	1.44	11		3500	feminir	28												
6	6.1-10	n„o	1.63	3	40	4300	feminir	72												
6	1.1-3	15 + tod	1.49	4		2820	mascul	24	21785.96	182.9	90.9	126.5	80	95	178	32	98	247	0.5719	
5	1.1-3	n„o	1.53	3		3640	mascul	17												
16	6.1-10	n„o	1.59	2	40	3450	feminir	23												
3	1 ou -	n„o	1.57	5	40	3310	mascul	28												
9	3.1-6	n„o	1.55	2	39	3050	mascul	79												
10	6.1-10	1-14 tod	1.6	1	40	3650	masculin													
4	1.1-3	1-14 par	1.52	1		1710	feminino		23011.65	155.3	58.2	129.5	77	92	140	57	72	39	0.6324	
3	1.1-3	n„o	1.49	3		3530	feminir	72	28394.87	161.8	68.7	116.5	76	80	215	60	144	62	0.5788	0.5785
11	1.1-3	1-14 tod	1.6	1	41	3750	feminino		22224.01	154.1	59.1	93.5	59.5	89	129	47	51	141	0.6387	0.5729
9	6.1-10	n„o	1.61	2	39	3980	mascul	15												
7	1 ou -	n„o	1.6	1		1180	masculin													
5	1.1-3	n„o	1.61	1	39	3330	masculin													
2	1.1-3	n„o	1.63	6		3280	mascul	39												
5	1 ou -	15 + tod	1.49	1	37	2400	feminino		16284.25	151.85	50.7	97	59	87	135	57	70	37	0.5733	0.5782
3	1.1-3	n„o	1.53	2	39	4000	mascul	57	30519.88	172.8	97.4	164	98.5	76	165	62	82	75	0.5844	0.5796
8	1.1-3	n„o	1.63	1	39	2520	feminino		15215.35	164.5	63	125.5	86	88	190	71	105	74	0.565	0.5781
4	1.1-3	n„o	1.54	1	39	3010	feminino			157.25	125.5	128.5	86	75	207	48	123	234		
6	1.1-3	n„o	1.53	2	39	2800	feminir	31	14196.66	162.9	62.4	125.5	89.5	77	185	68	100	95		
3	1 ou -	1-14 tod	1.58	1	40	3930	masculin		7071.114	175.9	63.2	119	69.5	53	154	46	96	43	0.5781	0.5785
5	3.1-6	1-14 tod	1.58	5	40	3000	feminir	50	17929.65	155.6	55.7	109	70.5	73	229	52	155	128	0.5778	0.5781
9	1 ou -	n„o	1.64	1	40	3410	feminino			174.2		102	51.5						0.577	0.5782

pescmae	prenda	pfumomae	paltmae	pgesta	pidgest	ppn	psex	pint	dmgtotdxa	daltura2012	dpeso	dsysmed	ddiamed	dglicose	dcolesterol	dhdl	dldl	dtrig	dECMICE	dECMICD
5	1.1-3	1-14 tod	1.55	2	29	1330	mascul	13												
5	1 ou -	n„o	1.51	1	38	3000	masculin		15619.82	169.1	67.3	124	78	117	293	70	187	171	0.5793	0.5879
8	1.1-3	n„o	1.61	1	37	2840	feminino		33954.89	161.7	78.5	127	92.5	83	160	68	82	81	0.5767	
14	1.1-3	15 + tod	1.5	2	38	2430	feminino		29076.46	156.4	73.7	136.5	88	80	200	79	94	159	0.5784	0.5796
0	1.1-3	1-14 tod	1.55	3	37	4590	mascul	36												
7	1 ou -	n„o	1.48	1	40	3150	masculin		18939.03	173.7	82	132.5	74.5	92	195	65	120	46	0.5762	0.5691
5	1.1-3	15 + tod	1.38	1	37	2800	masculin		13062.92	154.8	61.4	136.5	75.5	94	255	61	165	153	0.5817	0.6234
12	10	15 + tod	1.57	2	40	3170	feminir	30	43685.03	170.3	88.6	122.5	76.5	85	186	71	104	45	0.5879	0.576
3	1 ou -	n„o	1.55	2	41	2600	feminino													
10	1.1-3	n„o	1.57	3	36	3150	feminir	12	15851.34	163.75	49.9	131.5	78.5	95	164	66	76	99	0.5781	0.5807
4	3.1-6	n„o	1.46	1	41	3550	masculin		17231.97	161.25	71.5	122.5	77.5	91	299	45	233	97	0.6078	0.6005
5	1 ou -	n„o	1.59	1		2900	masculin		26094.49	177.05	91.1	163	96	109	270	57	154	320	0.5831	0.5782
12	3.1-6	n„o	1.65	2	40	3650	mascul	40	31580.44	177.2	91.4	133.5	76.5	93	212	52	125	176	0.5781	
7	1.1-3	1-14 tod	1.56	3	39	3430	feminir	24	25354.99	164.05	67.3	128	92.5	61	194	52	130	47	0.5779	0.578
6	1 ou -	n„o	1.62	3	39	3160	feminir	86	13089.12	160.1	50.3	103.5	66.5	85	214	82	116	52	0.5803	0.578
4	1 ou -	1-14 tod	1.6	1		3110	feminino													
12	3.1-6	n„o	1.61	1	39	3700	masculin		20208.35	185.8	85.1	138.5	78	78	210	58	137	104	0.581	0.5807
4	3.1-6	1-14 tod	1.55	5	41	4000	feminir	38	38251.15	159	91.5	122	81	85	239	70	152	84	0.6332	0.6858
4	1 ou -	n„o	1.61	2	39	2950	feminir	37	23211.76	160.9	66.2	103.5	66.5	73	176	65	89	125		0.5788
2	1.1-3	n„o	1.58	2	38	3500	feminir	26	23720.79	161.2	69.2	142	90	88	249	55	169	136	0.7857	0.6353
12	6.1-10	n„o	1.52	3	39	4000	feminir	78	20895.53			128.5	66.5							
4	1 ou -	n„o	1.47	4	39	2470	mascul	15												
0	1.1-3	n„o	1.52	9	39	3600	mascul	49	13282.96	169.1	66.9	115	70.5	93	158	50	94	80	0.5852	0.569
5	1.1-3	1-14 tod	1.63	1	38	2590	masculin													
3	1.1-3	n„o	1.59	5	38	3100	feminir	97	30355.65	164.6	68.7	105	69.5	93	208	75	109	125	0.5788	0.576
6	1.1-3	n„o	1.58	1		2550	masculin													
1	1 ou -	n„o	1.62	4	38	4100	feminir	24												
1	1 ou -	15 + tod	1.54	3		2810	feminir	34	22501.94	162.2	67.9	116	81	71	140	50	79	45		
12	6.1-10	15 + tod	1.65	3	39	4520	mascul	68												
12	3.1-6	n„o	1.65	4	39	3070	feminir	65												
8	3.1-6	n„o	1.72	2	40	3450	feminir	19												
4	1.1-3	n„o	1.52	1	41	2600	feminino		22328.57	159.55	58	110.5	65	75	200	74	98	125		
2	1 ou -	n„o	1.5	1	37	2310	masculin		20637.92	175.3	86.1	142.5	87.5	89	139	41	89	47		
2	1.1-3	n„o	1.52	4	38	3550	feminir	17												
1	1.1-3	1-14 tod	1.54	5	39	2900	feminir	73												
4	1 ou -	n„o	1.53	13		3800	mascul	93												
5	1.1-3	n„o	1.59	3	40	3450	mascul	17												
6	1.1-3	n„o	1.6	3	41	4470	feminir	49	41571.51	169.5	85.1	112	72.5	67	190	60	113	86	0.5805	0.5775
5	1 ou -	1-14 par	1.51	7	38	3640	mascul	16												
3	1 ou -	n„o	1.54	2	39	2730	mascul	35	7656.808	170.4	58	107.5	58.5	108	210	50	107	214		
1	1 ou -	15 + tod	1.66	2	40	2550	mascul	20	18791.2	168.2	70.6	124	78	80	148	56	83	50		0.5856
0	1.1-3	n„o	1.56	2	42	3940	feminir	21	26345.28	164.2	62.8	93	68	94	176	61	94	144	0.5774	0.5782
12	6.1-10	1-14 tod	1.61	1	39	3630	feminino													
2	1 ou -	1-14 tod	1.58	2	38	2400	mascul	147												
20	6.1-10	n„o	1.44	1	40	4030	masculin			153.35										
1	1 ou -	1-14 par	1.54	1		2550	feminino		34121.49	155.3	74.9	109.5	72.5	104	188	59	114	96	0.5786	0.5787
5	1.1-3	n„o	1.52	2	36	3450	mascul	71	30375.67	172.5	88.2	121	74.5	87	175	41	113	127	0.6862	0.6207
4	1 ou -	n„o	1.5	4	42	3510	mascul	19	10750.17	169.6	65.6	129	82.5	82	145	48	78	66	0.6138	0.5853

pescmae	prenda	pfumomae	paltmae	pgesta	pidgest	ppn	psex	pint	dmgtotdxa	daltura2012	dpeso	dsysmed	ddiamed	dglicose	dcolesterol	dhdl	dldl	dtrig	dECMICE	dECMICD
12	3.1-6	1-14 par	1.6	4	37	3700	feminir	30	38343.17	163.3	76.7	103.5	69.5	89	161	57	89	78	0.587	0.5814
5	1 ou -	1-14 tod	1.57	1		2570	masculin													
11	1 ou -	n„o	1.56	1	37	3500	feminino													
7	1.1-3	1-14 tod	1.58	1	40	2760	feminino		20555.21	159.15	62.6	120.5	78	87	170	85	65	104	0.5283	0.5736
7	3.1-6	1-14 tod	1.49	5	38	2720	feminir	86	25864.48	150.35	59.1	102.5	70.5	80	182	58	115	84		
8	1.1-3	1-14 par	1.63	1	39	2910	feminino		19105.96	163.25	56.7	109	66.5	86	208	68	123	76	0.6292	0.5953
16	10	n„o	1.65	2	40	3610	feminino		19007.95	171.7	68.2	113.5	72.5	72	186	80	93	94	0.5691	0.5687
3	1.1-3	n„o	1.57	2	40	3510	feminir	99	37103.82	159.95	75.6	114	68.5	78	238	78	134	136	0.5762	0.5744
3	1 ou -	n„o	1.53	3		2970	feminir	122	23287.96	149.7	62.1	100.5	68.5	83	188	90	70	123	0.5613	0.5724
5	1.1-3	n„o	1.49	2	39	3600	feminir	14	14799.85	158.85	51.7	108.5	64	81	144	58	72	49	0.5439	0.5792
7	1 ou -	1-14 par	1.62	1	38	2630	masculin													
12	3.1-6	1-14 tod	1.63	2		3350	feminir	18	12336.81	172.6	56.9	114.5	74.5	70	150	72	61	59	0.5722	
1	1 ou -	n„o	1.64	1	40	2530	masculin					119.5	70.5							
4	1.1-3	n„o		6	33	2280	mascul	12												
8	3.1-6	15 + par	1.57	1	39	2910	masculin		24394.96	179.15	90.5	117.5	68	78	151	40	92	144	0.5795	0.5825
7	3.1-6	1-14 par	1.46	3	40	2180	masculin													
5	1 ou -	1-14 tod	1.54	1	42	2300	feminino		23259.18	156.6	57.9	105	66	82	185	61	101	89		0.5763
4	3.1-6	1-14 tod	1.66	9	38	2430	mascul	66	8728.184	170.1	61.5	102.5	56.5	64	231	71	144	92	0.5735	0.5703
5	1.1-3	1-14 tod	1.6	1	39	3200	masculin													
5	1.1-3	n„o	1.56	10	42	4090	feminir	61												
8	1.1-3	15 + tod	1.65	3	43	3970	mascul	56	22827.64	178.8	83.5	131.5	77	81	200	42	127	154	0.5789	0.5793
7	1.1-3	1-14 tod	1.54	2	40	3300	mascul	21												
5	1.1-3	n„o	1.51	2	41	3700	feminir	81	24084.41	157.75	62.1	113	76	86	224	78	125	103	0.5768	0.5782
4	1.1-3	n„o	1.55	4		3330	feminir	41	14600.81	158.95	56.3	110.5	72	90	165	75	76	40	0.5784	0.5504
4	1.1-3	15 + tod	1.64	3		2450	feminir	12	18715.74	164.05	60.1	116	69.5	90	161	61	83	60		
5	1 ou -	n„o	1.57	1	35	2300	feminino		32396.54	162.5	75.7	119	80	83	200	87	95	65		0.5792
5	1.1-3	15 + tod	1.56	3	39	3830	mascul	11	13237.54	175.25	72.9	116.5	74	85	179	46	119	45		
15	10	1-14 tod	1.65	1	38	3150	feminino		23554.25	172.5	68.3	104	63	89	148	71	67	48		
2	1 ou -	1-14 tod	1.46	7	39	3070	mascul	22												
4	1.1-3	n„o	1.56	9		3330	feminir	14	18315.59	162.7	63.7	121	80.5	85	138	46	80	39	0.5783	0.5737
5	3.1-6	n„o	1.45	8	39	3150	mascul	19												
9	1.1-3	1-14 par	1.6	1	43	3500	feminino													
3	1.1-3	n„o	1.54	2	39	4100	mascul	46	34071.41	173.65	96.8	131	78	85	160	62	80	40	0.5713	0.5788
5	1.1-3	n„o	1.41	3	39	3200	feminir	26	22581.68	161.25	57.1	107	63.5	80	224	61	150	67	0.5809	0.5851
14	10	1-14 tod	1.51	1	38	2350	feminino													
5	1.1-3	n„o	1.58	2	37	2750	feminir	20	20743.73	170.3	62.3	109	62.5	130	173	68	96	71		0.5708
8	3.1-6	n„o	1.63	3	42	3330	feminir	35	26191.24	164.85	69.2	118	81	85	188	48	128	67	0.5744	0.5749
1	1.1-3	n„o	1.57	5		2480	mascul	71												
6	1.1-3	n„o	1.53	1	42	3350	masculin													
8	1.1-3	n„o	1.67	4	39	3700	mascul	18												
5	1 ou -	1-14 tod	1.54	1	39	3220	masculin		28059.89	179.3	91.8	117.5	73	100	215	57	136	123	0.5877	0.5982
5	1.1-3	n„o	1.52	1		4000	feminino		50318.79	164.2	105			92	156	55	89	54	0.5782	0.5794
6	1.1-3	1-14 tod	1.57	1	38	2470	feminino		18281.29	157.7	56.2	119	70	96	193	79	98	99	0.5766	0.5779
10	3.1-6	1-14 tod	1.65	1	40	2900	feminino		16112.46	166.55	55.3	112.5	68.5	87	214	85	116	96	0.5782	0.5778
8	1.1-3	1-14 tod	1.57	3		3880	feminir	16	39786.73	163.15	86.4	116	80.5	113	156	53	59	361	0.5787	
5	1.1-3	n„o	1.58	3	41	4800	feminir	44	12361.2	165.65	48.7	113	69.5	101	152	79	56	73	0.5753	0.5767
6	1.1-3	n„o	1.58	1	39	3800	feminino													
9	10	n„o	1.6	2		3020	masculin			176.6	127.7	174	107	88	245	58	161	150	0.5826	0.5782

pescmae	prenda	pfumomae	paltmae	pgesta	pidgest	ppn	psex	pint	dmgtotdxa	daltura2012	dpeso	dsysmed	ddiamed	dglicose	dcolesterol	dhdl	dldl	dtrig	dECMICE	dECMICD
8	3.1-6	15 + tod	1.54	3	37	3000	mascul	47												
8	1.1-3	n„o	1.58	9	40	4050	mascul	50												
12	6.1-10	n„o	1.6	3	40	3650	feminir	18		166		111	68						0.578	0.5764
1	1 ou -	n„o	1.49	7	39	3510	feminir	26												
5	1.1-3	1-14 tod	1.6	3	40	3620	feminir	162	50345.01	161.85	102.1	125	81.5	85	219	64	135	111	0.5786	0.5811
4	1 ou -	15 + tod	1.52	2		1820	mascul	17	7846.627	169.2	67.5	169.5	98	85	180	89	73	62	0.5779	0.5775
4	1.1-3	n„o	1.68	3		4070	mascul	16	27995.42	180.75	91.5	116	70	106	208	47	122	197	0.5788	0.5792
4	1.1-3	n„o	1.55	1	37	3700	masculin		31466.83	175.65	89.1	117.5	76	118	280	50	129	500	0.5812	0.5736
4	1 ou -	n„o	1.57	1	39	2650	masculin		3584.232	173.4	54.3	117.5	72	93	216	66	135	87		
12	3.1-6	n„o	1.6	1	42	3420	feminino													
4	1 ou -	n„o	1.55	1	40	3400	feminino		18542.06	163.05	57.3	112	74	101	162	79	72	51		
5	1 ou -	1-14 tod	1.58	1	41	2990	masculin		19185.72	177.2	81.9	138	79	73	211	52	132	150	0.6064	0.614
16	6.1-10	n„o		1	40	3350	feminino		17830.43	161.3	52.7	129.5	74	93	220	74	108	154		
8	6.1-10	1-14 par	1.58	1	39	3720	feminino		24286.73	161.3	63	129	80	67	196	52	129	62	0.5709	0.5781
5	1.1-3	n„o	1.56	1		2850	masculin													
0	1 ou -	1-14 tod	1.49	2		3730	feminir	17	21088.79	155	65.1	111	67.5	78	150	54	88	47	0.5894	0.5784
5	1.1-3	n„o	1.63	2	38	3660	feminir	23	23491.79	160.65	61.1	106	67.5	81	192	65	112	88	0.5758	0.5769
4	3.1-6	n„o	1.62	2	38	3600	mascul	61	13572.39	185.6	71.5	139	89.5	101	138	52	70	87		
8	1.1-3	1-14 tod	1.56	2	40	3770	mascul	19	6736.16	179.55	63	124	77.5	79	156	50	92	56	0.5624	0.5778
7	1.1-3	n„o	1.6	1	41	3400	feminino													
4	1.1-3	n„o	1.55	3	40	3290	mascul	80												
3	1.1-3	n„o	1.5	2		3120	feminino													
1	1.1-3	1-14 par	1.53	2		2770	feminir	14												
3	1.1-3	n„o	1.46	1	38	2800	feminino		33762.96	159.7	83.8	127	79.5	90	185	53	120	86	0.6362	0.6012
5	1.1-3	n„o	1.52	3	40	3170	feminir	50												
5	1.1-3	n„o	1.56	2		3400	feminir	38	26746.27	159.3	66.8	119.5	80.5	98	220	75	120	109	0.6098	0.6361
0	1.1-3	15 + tod	1.51	3	38	2490	mascul	116												
8	1.1-3	n„o	1.57	1		3370	masculin													
4	1.1-3	1-14 par	1.57	2		4200	feminir	25												
3	1.1-3	1-14 tod	1.57	3	41	2700	mascul	50												
11	10	n„o	1.63	1	41	3190	masculin													
4	1 ou -	1-14 tod	1.58	1	39	3200	masculin		14638.65	173.05	70.3	110.5	68	83	184	55	106	111	0.5905	0.5776
12	3.1-6	15 + tod	1.61	3	41	2600	mascul	28	31697.5	177.1	105.3	149	99	107	174	32	70	389	0.5781	0.5834
14	10	1-14 tod	1.58	3	39	3300	mascul	164	22221.53	172.8	82	134	65	85	209	60	126	119		
15	3.1-6	1-14 tod	1.59	1	36	2570	feminino		22086.83	161.7	60.6	115.5	82	101	242	85	145	65	0.5762	0.5291
7	3.1-6	n„o	1.65	1	40	4050	masculin			191.75	111.2	116.5	72.5	100	211	53	142	92	0.5803	0.5786
7	1.1-3	n„o	1.6	1	42	3900	masculin		10658.06	180.8	63.5	114.5	64.5	72	224	47	162	90	0.5791	0.5716
3	1.1-3	1-14 tod	1.45	2	37	2810	feminir	15	24649.48	155.2	66.4	96.5	65.5	86	169	72	86	76	0.5783	0.5793
5	1 ou -	n„o	1.6	9	39	2950	feminir	16												
7	1.1-3	1-14 tod	1.64	2	38	2800	feminir	40												
5	3.1-6	n„o	1.52	3		2580	feminir	103	16545.26	155.3	50.5	107.5	71.5	91	179	77	78	106	0.5707	0.5684
8	1.1-3	15 + tod	1.58	2		4010	mascul	36	34218.31	169.35	96.7	132	91.5	111	186	44	131	90	0.5998	0.5795
2	1.1-3	n„o	1.48	1	37	3320	masculin													
6	1 ou -	1-14 tod	1.64	2	39	3100	mascul	47	12723.49	174.25	76.8	114.5	68.5	89	164	46	107	55	0.5785	0.6047
0	1.1-3	1-14 tod	1.54	5		2080	mascul	102	23388	152.5	72.4	109	70	89	201	42	127	155	0.6124	0.5797
7	1.1-3	n„o	1.67	5	37	4680	feminir	89		164.6		116	74.5						0.5713	0.5806
4	1 ou -	1-14 tod	1.54	1	39	3630	masculin		20363.06	174.4	81.4	122.5	72.5	89	174	60	99	86	0.5748	0.5793
7	1.1-3	1-14 tod	1.71	1	39	3680	masculin		22635.86	181.5	98.9	132.5	88	90	170	67	82	114	0.5981	0.5797

pescmae	prenda	pfumomae	paltmae	pgesta	pidgest	ppn	psex	pint	dmgtotdxa	daltura2012	dpeso	dsysmed	ddiamed	dglicose	dcolesterol	dhdl	dldl	dtrig	dECMICE	dECMICD
6	1.1-3	15 + tod	1.47	3		2260	mascul	14												
14	3.1-6	n„o	1.54	2	32	2340	mascul	29	36900.7	183.95	108.9	135.5	91.5	69	258	57	134	337	0.5771	0.6033
5	1.1-3	n„o	1.58	2	41	3200	feminir	23	16762.11	157.65	54.3	116	67.5	88	208	70	129	72	0.5804	
4	1.1-3	15 + tod	1.63	2		2600	feminir	23	19550.3	154.3	56.1	113	73.5	153	260	74	151	273		0.5638
9	1.1-3	n„o	1.55	1	38	3300	masculin		4805.534	170.7	62.8	129	64	70	195	64	103	165	0.5767	0.5791
3	1 ou -	n„o	1.5	2		3050	feminir	81												
5	1.1-3	n„o	1.51	3	40	3400	feminir	29	26053.24	152	65.7	110.5	64	88	179	46	123	67	0.5796	0.5771
5	1.1-3	n„o	1.56	3	40	3520	feminir	23	40190	161.3	83	129.5	84	86	150	46	84	75	0.573	0.5771
11		15 + tod	1.64	1	40	3290	feminino													
5	3.1-6	n„o	1.56	4	38	3100	mascul	92												
17	10	n„o	1.53	2	38	3370	mascul	42	14851.59	169.5	72.9	119	65.5	80	179	76	87	72	0.5907	0.5698
5	1.1-3	15 + tod	1.64	1	41	2300	feminino		26739.09	167	65.2	136	84.5	78	190	46	119	180	0.5748	0.571
8	1.1-3	n„o	1.53	1	40	3200	masculin		27303.74	177.1	89.6	130.5	81	74	187	60	109	79	0.5809	0.5621
5	3.1-6	n„o	1.54	12	42	3750	mascul	22	25190.43	175.8	89.3	158	86	102	186	44	113	106	0.5834	0.5821
1	1.1-3	n„o	1.59	5	39	3550	mascul	52	17526.27	181.75	74.3	113.5	67.5	87	209	55	131	145		0.5784
15	6.1-10	15 + tod	1.57	3	39	3460	mascul	104												
4	1.1-3	n„o	1.58	12	40	4060	mascul	53	11534.56	181.6	77.5	130.5	74	97	157	45	83	150	0.5781	0.5865
3	1.1-3	15 + tod	1.42	7	42	2600	mascul	33	16973.94	169.5	75.8	127	70	75	168	45	96	122	0.5785	0.5953
13	3.1-6	n„o	1.55	1	39	3800	masculin		6470.89	177.85	70.9	110.5	67	68	216	58	130	144	0.5789	0.5748
6	1.1-3	1-14 tod	1.56	3	38	3150	mascul	20												
6	3.1-6	1-14 par	1.61	3	36	2750	mascul	160	7921.556	175.25	60	102	62	84	180	70	98	64		
5	3.1-6	n„o	1.59	3	39	3230	mascul	48	10381.77	180.5	62	124.5	74.5	80	148	72	65	39	0.5357	
18	10	1-14 tod	1.66	1	39	3600	feminino		16777.21	164.55	57.4	138	86	92	212	105	84	103	0.5773	0.5682
4	3.1-6	n„o	1.54	3	40	3650	mascul	103	8818.4	174.1	57.6	136	70	93	166	57	93	81		0.5744
1	1 ou -	n„o	1.54	1		2900	masculin		19702.91	177.8	78.9	138.5	68.5	93	260	51	175	82	0.5813	0.5797
5	1.1-3	n„o	1.57	3		3850	feminir	43	17466.31	167.05	59.8	111	74.5	94	169	65	93	42	0.5764	0.5782
5	1.1-3	n„o	1.51	3	40	3180	mascul	63												
4	1 ou -	n„o	1.5	2	36	2350	feminir	19	12422.28	155.45	50	106	67	52	163	57	97	55		
5	1.1-3	n„o	1.69	2	39	3450	mascul	22												
0	1 ou -	15 + tod		5	43	2750	feminino		27166.53	153.25	66.7	116.5	75.5	79	168	58	95	77	0.5866	0.5806
2	1.1-3	1-14 tod	1.48	1	40	2820	feminino		33561.69	163.7	72.9	121.5	81	89	144	46	89	51		
7	1.1-3	n„o	1.62	1	41	2810	masculin		32982.04	191.6	99.6	129	81	81	207	72	126	65	0.6838	0.5999
5	1.1-3	1-14 tod	1.53	1		2350	masculin													
4	1.1-3	15 + tod	1.53	3	40	3100	mascul	41												
6	1.1-3	1-14 par	1.51	2	38	3350	feminir	16												
5	1 ou -	1-14 tod	1.52	11	38	2370	mascul	27												
7	1 ou -	n„o	1.5	2	40	3200	mascul	48	19283.46	162	70.4	150	94.5	126	246	36	129	393	0.5803	0.5692
8	1.1-3	1-14 tod	1.51	1	38	3130	feminino		30461.45	156.85	67.2	111	68	86	218	88	107	91		
5	3.1-6	n„o	1.36	2	39	2940	feminir	51												
9	10	n„o	1.57	3	40	3450	mascul	32	14294.39	182.2	84.2	131.5	79.5	104	217	72	124	112	0.5742	0.5794
5	1 ou -	n„o	1.55	3	37	2700	mascul	64	11723.49	170.7	67	114	64	91	236	61	134	198	0.5792	0.578
9	6.1-10	n„o	1.72	1	38	3800	feminino		25962.99	179.8	70.3	106	70	109	184	71	99	77	0.5784	0.5781
10	6.1-10	n„o	1.55	2	42	2900	feminir	19	38378.96	157.25	85.7	121.5	80.5	112	190	56	107	152		
5	1.1-3	n„o	1.66	3		3000	feminir	79	21714.23	168.6	62.7	113	75	86	164	61	83	92	0.5764	0.5747
5	1 ou -	n„o	1.59	3	37	2770	mascul	25												
7	3.1-6	n„o	1.58	1	40	2200	masculin		26010.85	168.2	92.6	144	84.5	80	188	46	121	126	0.5838	0.5836
11	6.1-10	n„o	1.64	2	42	3600	feminir	12	50317.96	162.55	96	133.5	89.5	101	207	59	131	105	0.587	0.5912
6	1.1-3	n„o	1.61	3	39	3590	mascul	32												

pescmae	prenda	pfumomae	paltmae	pgesta	pidgest	ppn	psex	pint	dmgtotdxa	daltura2012	dpeso	dsysmed	ddiamed	dglicose	dcolesterol	dhdl	dldl	dtrig	dECMICE	dECMICD
3	1.1-3	n„o	1.46	1	40	4120	masculin													
3	1 ou -	15 + tod	1.45	2	40	2700	feminir	32	9842.293	151.8	46.7	111	70	73	224	54	152	83	0.5907	
3	1.1-3	1-14 tod	1.62	2	41	2980	masculin													
11	3.1-6	1-14 par	1.68	1	41	2900	masculin		19008.84	182.5	82.3	130	72.5	73	195	60	121	78		
3	1 ou -	n„o	1.53	1	38	3300	masculin													
7	1.1-3	1-14 tod	1.63	2	43	3870	mascul	31		183.95	123.8	126	85	108	247	49	157	163	0.603	0.5744
11	1.1-3	1-14 tod	1.54	1	38	3470	feminino		17384.16	162.8	53.3	99	63.5	78	165	55	94	88	0.5775	0.5797
11	3.1-6	1-14 par	1.5	1	42	3710	feminino													
5	1.1-3	1-14 tod	1.55	1	40	3200	masculin													
11	1.1-3	1-14 tod	1.49	3	38	3000	feminir	42	31557.52	153.85	65.6	106	74.5	76	151	62	78	99	0.5689	0.5706
11	3.1-6	n„o	1.55	1	40	3970	masculin													
16	10	n„o	1.61	2	41	4350	mascul	15	22306.91	181.75	83.9	128.5	79.5	74	175	59	94	109	0.5754	0.5938
2	1.1-3	n„o	1.48	2		2880	feminir	44	20526.99	152.5	57.7	126.5	81.5	90	239	79	130	116		
5	1.1-3	n„o	1.53	2	38	3050	feminir	41												
5	1.1-3	n„o	1.59	3	37	2730	feminino		14792.21	157.65	47.4	112.5	70	98	189	57	115	104		
6	1 ou -	1-14 tod	1.5	2	39	2950	mascul	27	19536.43	169.45	71.4	140	79.5	91	211	52	143	102		
5	1 ou -	n„o	1.53	4	37	2880	mascul	24												
12	3.1-6	n„o	1.6	1	38	3650	masculin		11612.11	175.2	78.1	116	62.5	80	130	35	79	63	0.5783	0.5851
1	1 ou -	n„o	1.63	1	39	2330	masculin		19883.51	174.8	73.8	112.5	78.5	89	195	51	125	115	0.5731	
13	10	n„o	1.59	2	39	2570	feminir	32												
7	3.1-6	n„o	1.55	2	38	3210	feminir	157												
4	1.1-3	n„o	1.53	7	39	3780	feminir	58	34208.05	163.9	72.3	126.5	83	93	180	54	117	51	0.5754	0.5768
5	1.1-3	n„o	1.64	1	42	3830	masculin													
9	1.1-3	n„o	1.58	1	39	3100	masculin													
15	6.1-10	n„o	1.65	1	38	3900	masculin													
5	1.1-3	n„o	1.61	2	42	2960	feminir	25												
16	3.1-6	n„o	1.59	1	38	2890	feminino		18182.11	170.1	58.1	94.5	61	88	195	73	103	77	0.5796	0.5781
13	3.1-6	n„o	1.66	2		3150	feminir	20	17699.54	170.7	57.1	109	66.5	87	168	44	115	41	0.5734	0.5691
4	1.1-3	n„o	1.58	5	36	3330	feminir	30	34582.45	165.35	85.2	113	78.5	77	224	48	144	116	0.5793	0.578
4	3.1-6	1-14 par		2	39	3700	feminir	212	26471.56	160.95	67.8	116.5	71	69	141	70	60	66	0.5823	0.5836
4	1.1-3	n„o	1.63	2	37	2850	mascul	39	22687.08	171.8	80.5	138	76	90	170	27	100	213	0.5798	0.5878
11	1.1-3	1-14 tod	1.59	1	41	2570	masculin													
3	1 ou -	1-14 tod	1.57	6	38	3060	feminir	86	18635.95	166.3	63.1	96	59	77	128	54	63	42		
4	1 ou -	1-14 par	1.52	1	39	2460	masculin		3787.254	172.7	53.8	119	76						0.5781	0.5782
4	1.1-3	n„o	1.6	3	36	3370	feminir	22	19890.8	160.9	63.9	94.5	60.5	79	179	73	96	75	0.5832	
2	1.1-3	n„o	1.67	6	40	4000	mascul	65	26072.94	179.65	91	149.5	83	101	208	39	113	296	0.5788	0.5778
8	3.1-6	n„o	1.6	4		3760	feminir	29	39783.83	150.95	75.8	113	75.5	75	222	90	116	101	0.5335	0.5788
16	6.1-10	n„o	1.57	4	40	3250	feminir	17												
8	3.1-6	n„o	1.58	2	39	2880	feminir	32	30567.48	162.25	78.1	126	84	87	195	57	125	59	0.598	
5	1 ou -	n„o	1.54	1	37	4020	feminino		31463.23	155.35	68.9	122.5	76	94	167	71	78	105	0.5787	0.5758
5	1.1-3	n„o	1.69	6	38	4030	mascul	18	12034.58	180.1	88.1	142	78.5	96	246	65	168	70	0.6085	0.6328
7	1.1-3	1-14 tod	1.66	6	40	3880	mascul	14	21931.29	174.75	86.4	137	86.5	94	278	53	183	190	0.6535	0.607
4	1 ou -	15 + tod	1.5	10	42	3150	feminir	38												
7	1.1-3	1-14 tod	1.5	1	38	2670	masculin													
6	1.1-3	n„o	1.57	1	40	3570	masculin													
5	1.1-3	n„o	1.54	2	40	2480	mascul	20	14917.13	164.7	76.2	119.5	77.5	65	234	50	143	215	0.5781	0.6645
4	1 ou -	n„o	1.49	2	41	2170	feminir	18												
14	10	1-14 tod	1.54	2	39	3520	mascul	11												

pescmae	prenda	pfumomae	paltmae	pgesta	pidgest	ppn	psex	pint	dmgtotdxa	daltura2012	dpeso	dsysmed	ddiamed	dglicose	dcolesterol	dhdl	dldl	dtrig	dECMICE	dECMICD
6	6.1-10	1-14 tod		3	38	3350	mascul	70												
2	1 ou -	n„o	1.49	3	38	2930	mascul	72	5306.288	163.6	50.7	125.5	78	84	150	62	78	42		
9	1.1-3	n„o	1.55	1	37	2450	feminino		21380.67	153.15	55.6	114.5	69.5	102	193	77	103	71	0.5785	0.5618
2	1.1-3	n„o	1.49	3	42	3200	feminir	65	25971.26	153.95	61.7	107	68	86	202	82	110	64		
6	3.1-6	n„o	1.64	2	42	3450	mascul	19	22977.54	177.5	84.3	118.5	72	89	149	41	100	55	0.5806	0.58
11	1.1-3	n„o	1.58	2	39	3670	masculin		21788.09	172	83.3	123	77.5	94	166	53	95	96	0.5779	0.5822
3	3.1-6	1-14 tod	1.6	1	38	2530	masculin													
4	1.1-3	n„o	1.56	3		2940	mascul	62	2755.263	167.75	52.9	123.5	64	74	169	32	109	149	0.5781	0.5782
5	1.1-3	n„o	1.65	4	39	3250	mascul	66												
8	1 ou -	1-14 tod	1.5	6		2000	mascul	10												
6	1.1-3	n„o	1.51	4	42	3000	feminir	54	22920.59	164.9	62.9	101	70.5	89	229	90	118	119	0.5781	0.5788
9	1 ou -	1-14 par	1.61	1		3230	masculin													
5	1.1-3	1-14 tod	1.49	2	39	2820	feminir	45	20290.98	150.6	55.3	96	64	90	158	70	72	59	0.5677	
8	3.1-6	15 + tod	1.58	1		2500	feminino		30355.49	155.3	70.1	116.5	71.5	101	231	82	118	184	0.5814	0.5849
9	3.1-6	n„o	1.52	1	42	3200	masculin		23438.97	174.15	81.8	111.5	68.5	85	201	57	134	80	0.5766	0.5839
12	6.1-10	n„o	1.53	1	40	3770	feminino		13565.23	172.55	55.6	119.5	77	75	169	88	66	55	0.5628	0.5769
5	3.1-6	n„o	1.54	1	40	3150	feminino		18895.24	154.4	56.6	136	93.5	84	156	66	82	49	0.578	0.5782
4	1.1-3	n„o	1.55	5	41	3300	mascul	31												
3	1.1-3	n„o	1.59	4	38	3300	mascul	51	28740.74	175.9	91.5	139	86	83	242	54	156	127	0.5788	0.5779
5	1.1-3	n„o	1.54	1	37	2410	feminino		42494.64	151.6	82.2	113	68.5	81	173	47	105	102	0.5778	
11	1.1-3	1-14 par	1.59	3	42	3510	feminir	35	21362.04	157.75	61.6	100.5	63	59	225	73	138	68		
4	1 ou -	1-14 tod	1.52	3	40	2700	feminir	15	21820.35	161.2	58.7	104	68.5	83	188	88	89	49	0.5781	0.5798
0	1 ou -	n„o	1.48	7	41	3920	mascul	41												
7	1.1-3	1-14 tod	1.52	2	40	4150	mascul	47	40381.33	171.6	107.3	128.5	80.5	112	211	34	128	220	0.5784	0.5853
9	6.1-10	n„o	1.55	3		2950	mascul	12												
8	1.1-3	n„o	1.52	1	37	2550	masculin													
5	1 ou -	n„o	1.55	3	37	2380	feminir	20	20029.71	156.65	55.3	98.5	63	86	129	55	58	79	0.5844	0.5703
7	1 ou -	1-14 tod	1.59	2	37	2970	mascul	19	10843.38	167.3	67.9	133.5	71	90	215	52	143	85	0.6436	0.5771
6	3.1-6	n„o	1.63	4	40	2000	feminir	56	25524.64	154.3	66.4	109	66	85	157	49	99	61	0.612	0.5777
6	1 ou -	n„o	1.55	1	40	3130	masculin			175.85	121.6	132	84	82	220	55	133	175	0.5775	
5	1 ou -	1-14 tod	1.68	2	39	4250	mascul	49	15462.08	178.3	73.3	112	69.5	98	209	67	123	114	0.5648	0.5789
6	1.1-3	n„o	1.65	1	38	4020	masculin		23172.9	179.7	76.1	134	76.5	80	170	57	90	84	0.5768	0.5762
5	1 ou -	1-14 par	1.53	2	41	2670	feminir	41												
15	3.1-6	1-14 tod	1.53	2	35	2750	feminir	43												
3	1.1-3	1-14 tod	1.58	2		3460	feminir	136	31229.39	166	76.2	116	77.5	97	154	50	90	68	0.6152	0.658
0	1.1-3	n„o	1.6	7		3080	feminir	59	30668.47	171.25	79.6	137	81	78	178	74	86	68	0.6121	0.5794
10	3.1-6	n„o	1.56	1	40	3300	masculin													
5	1.1-3	15 + tod	1.57	3	37	2730	feminir	25	22918.48	161.1	62.2	108	66.5	78	170	64	90	60	0.578	0.5787
12	1.1-3	1-14 par	1.6	1		2900	masculin													
8	1 ou -	n„o	1.65	2	38	3120	mascul	71												
10	3.1-6	1-14 tod	1.64	1	39	3300	feminino		27737.92	167.4	69.2	115	75.5	105	163	56	86	118	0.5812	0.5803
3	1.1-3	n„o	1.65	4	41	3850	feminir	147	36062.9	168	78.5	97	62.5	87	137	47	77	41	0.5475	
5	1.1-3	n„o	1.59	3	40	2980	feminir	23												
5	3.1-6	n„o	1.58	6	41	4000	mascul	17												
5	1.1-3	n„o	1.56	4		3300	mascul	22	20921.09	177.45	82.9	128	74.5	121	150	59	78	50	0.5864	0.5681
14	3.1-6	n„o	1.63	1	41	3590	feminino		17472.62	171.1	62.4	111.5	66.5	77	195	93	86	81	0.5745	0.5771
5	1.1-3	n„o	1.6	1	40	2850	masculin													
5	1.1-3	1-14 tod	1.5	6	41	3200	feminir	12	18191.19	155.65	54.6	109	73	81	136	70	53	45	0.5767	0.5788

pescmae	prenda	pfumomae	paltmae	pgesta	pidgest	ppn	psex	pint	dmgtotdxa	daltura2012	dpeso	dsysmed	ddiamed	dglicose	dcolesterol	dhdl	dldl	dtrig	dECMICE	dECMICD
11	1.1-3	n„o	1.6	1	38	3000	masculin		19275.86	172.2	66.2	131	86.5	77	213	53	140	145	0.5779	0.577
3	1.1-3	n„o	1.53	4	38	3500	mascul	22	12609.33	168.25	63.4	120.5	76	83	149	47	89	45	0.5779	0.5715
7	3.1-6	1-14 tod	1.69	1	38	2780	feminino													
7	1.1-3	1-14 tod	1.64	1		1300	feminino		18369.21	172.5	59	99.5	65	84	131	55	68	35	0.5793	0.5773
6	1.1-3	1-14 tod	1.52	1	37	3400	masculin													
9	10	n„o	1.65	3	39	4000	mascul	55												
8	10	n„o	1.51	3	41	3840	feminir	21	21505.62	163.95	64.2	118	71.5	73	153	54	91	60	0.5777	0.58
11	1.1-3	n„o	1.61	1	39	3700	masculin		18118.35	181.1	86.1	136	82.5	89	142	47	80	91	0.6247	0.5782
5	1.1-3	1-14 tod	1.55	4	41	2800	feminir	36												
4	1.1-3	n„o	1.64	1		2960	masculin		13180.35	176.9	77.3	123	69.5	86	217	63	139	67	0.5767	0.5783
5	3.1-6	n„o	1.58	3	42	3450	mascul	27	12906.75	178.65	71.3	144.5	87	97	198	78	110	82	0.5657	0.5797
9	3.1-6	1-14 tod	1.67	2	41	2900	mascul	45												
5	1.1-3	15 + tod	1.48	2	39	2450	feminir	16	11449.37	151.2	47.3	101	57.5	70	119	49	62	37	0.5457	0.5804
5	1.1-3	n„o	1.55	4	37	3470	mascul	150												
5	1 ou -	n„o	1.61	1	39	3350	masculin		31177.58	182.55	102.1	131.5	73	63	208	49	145	84		
4	1.1-3	n„o	1.54	1	41	3520	masculin													
3	1.1-3	n„o	1.54	4	42	3280	feminir	72												
5	3.1-6	n„o	1.6	6	37	3250	feminir	137	28390.65	167.5	67.9	112	75	60	226	64	143	86		0.5854
8	1.1-3	n„o	1.76	1		3250	masculin													
15	6.1-10	n„o	1.57	3	41	3820	mascul	49	27659.41	168.55	83.8	126.5	78.5	132	200	49	78	653	0.5804	0.5881
3	1 ou -	1-14 par	1.58	2	39	3600	mascul	34												
8	1 ou -	n„o	1.6	2	39	3550	mascul	18	13008.79	172.5	77.1	126.5	82.5	78	175	40	104	199	0.5761	0.5769
5	1 ou -	n„o	1.51	5	39	3250	mascul	51												
3	1.1-3	15 + tod	1.62	2	38	3450	mascul	33	22744.34	176	88.6	125.5	77.5	88	144	35	87	128	0.5791	0.579
14	10	n„o	1.52	1	41	3530	masculin													
7	1 ou -	1-14 par	1.61	1	42	3500	masculin		30499.75	173.25	89.3	115	72.5	78	225	59	140	190	0.5708	0.6136
5	1 ou -	1-14 par	1.53	1	34	2200	feminino		33389.03	163.95	73.7	110.5	69.5	70	220	88	113	106		
5	1.1-3	n„o	1.62	1	38	3650	masculin		16141.8	177.6	85.8	119	73.5	77	167	56	92	76	0.5777	0.6109
10	3.1-6	n„o	1.47	2	40	2880	feminir	19												
5	1.1-3	n„o	1.51	1	41	1900	masculin		15850.75	158.1	58.1	126.5	83	91	169	62	76	121		
5	1.1-3	n„o	1.55	4	40	3440	feminir	39												
5	1 ou -	1-14 tod	1.52	5	40	3010	feminino		35807.98	159.05	83.7	112	69.5	90	199	49	135	72	0.5729	0.5775
10	1.1-3	n„o	1.53	5		3950	mascul	33	13433.19	173.8	78.9	143	81	74	141	51	78	54	0.5788	0.5937
9	1.1-3	n„o	1.42	3	39	3200	mascul	32	4905.329	163.35	48.8	170	99.5	79	233	83	142	59	0.5869	0.5686
3	1 ou -	15 + tod	1.49	2		2600	feminir	21	16074.94	151.5	54.1	127.5	75	78	189	58	115	112	0.5748	0.578
16	3.1-6	1-14 tod	1.65	1	40	3700	masculin													
12	10	1-14 tod	1.67	1	37	3050	feminino		14064.51	167	51.7	97.5	69	74	168	65	85	51	0.5867	0.5903
8	3.1-6	n„o	1.61	2	40	3420	mascul	25	16563.67	169	66.5	118.5	78.5	86	199	58	118	116	0.5819	0.5786
4	6.1-10	n„o	1.59	4	38	3550	mascul	120												
10	1.1-3	1-14 par	1.61	1	38	3430	masculin		4274.237	173	59.9	123	73.5	117	216	62	133	98	0.5757	0.5781
7	1.1-3	n„o	1.59	2	40	3580	feminir	39												
6	1.1-3	1-14 tod	1.6	2	41	3560	feminir	29	20383.02	159.95	59.8	111	77.5	91	177	58	101	117	0.5716	0.5776
11	3.1-6	n„o	1.63	1	43	3570	masculin													
6	1.1-3	n„o	1.59	2	33	1460	mascul	18												
9	1.1-3	1-14 par	1.63	2	40	2970	feminir	97												
5	1.1-3	n„o	1.5	1	40	3600	feminino		19801.08	155.75	58.3	109.5	69.5	85	178	63	102	60	0.5725	0.5823
1	1 ou -	n„o	1.4	1	39	3150	masculin		16866.77	164.8	69.9	126.5	79	81	130	41	70	104	0.5782	0.5735
1	3.1-6	n„o	1.65	5	40	3700	feminir	21												

pescmae	prenda	pfumomae	paltmae	pgesta	pidgest	ppn	psex	pint	dmgtotdxa	daltura2012	dpeso	dsysmed	ddiamed	dglicose	dcolesterol	dhdl	dldl	dtrig	dECMICE	dECMICD
6	3.1-6	1-14 tod	1.59	1	40	3350	masculin													
13	1.1-3	n,,o	1.53	1	35	1650														
16	10	n,,o	1.55	2	40	3820	feminir	94	13671.13	162.2	50.5	102.5	60							
4	1.1-3	1-14 tod	1.51	8	39	2900	feminir	96												
5	1.1-3	n,,o	1.56	1	38	3200	feminino		12782.77	164.95	53.8	102	66	76	169	60	90	50	0.5794	0.5777
1	1 ou -	n,,o	1.63	13		3070	mascul	14												
14	3.1-6	1-14 tod	1.58	1	36	3010	feminino		19990.68	163	63	111.5	69.5	85	192	68	100	76	0.5778	0.5743
4	3.1-6	n,,o	1.6	5	39	3560	feminir	86	22990.4	169.25	67.2	119.5	75.5	91	128	59	56	65	0.5656	0.5786
4	1.1-3	1-14 par	1.5	1	41	3220	feminino													
5	1.1-3	n,,o	1.59	2	40	2800	feminir	56	21325.47	166.35	60.5	125	79.5	88	135	42	82	40		0.5721
15	3.1-6	15 + tod	1.53	7	35	2600	mascul	18												
17	6.1-10	n,,o	1.6	2	40	3570	mascul	26												
1	1 ou -	n,,o	1.57	8	39	3180	feminir	29												
0	1 ou -	1-14 tod	1.54	4		3100	feminir	36	33639.49	151.85	76.9	102.5	61.5	92	172	55	104	112	0.5776	0.5792
6	1.1-3	1-14 tod	1.61	1	38	3010	feminino													
8	1.1-3	n,,o	1.58	1	41	3110	feminino													
11	1 ou -	n,,o	1.55	2	39	3460	feminino		21257.14	156.9	58	98.5	64.5	70	168	44	111	66	0.5762	0.5657
9	1.1-3	1-14 tod	1.54	1	42	2930	feminino		20375.91	158.55	60.8	95.5	60.5	81	182	65	102	62	0.5803	0.5781
13	6.1-10	15 + tod	1.69	1	40	3830	masculin													
16	3.1-6	n,,o	1.52	1	41	3200	masculin		35736.66	168.65	94.5	130	88.5	111	178	43	93	231	0.5606	
14	1.1-3	1-14 par	1.77	1	38	3250	masculin													
5	1.1-3	n,,o	1.59	2	40	3250	feminino		33384.05	160.7	77	106.5	67	76	134	51	66	124	0.578	0.5783
9	1.1-3	n,,o	1.56	2	37	3510	mascul	47	5063.311	187.35	76.2	126	78.5	94	164	54	102	73	0.5781	0.5783
2	1.1-3	n,,o	1.54	1		2870	masculin													
3	1.1-3	n,,o	1.59	1	39	2880	feminino		19057.04	164.7	58.2	99	65.5	80	223	46	152	138	0.5783	0.584
3	1.1-3	n,,o	1.55	2	42	3150	mascul	144	11424.77	173.8	72.9	123	68.5	79	140	61	68	45	0.5659	0.5693
11	1 ou -	n,,o	1.62	3	38	3000	feminir	19												
2	1 ou -	1-14 par	1.53	3	42	3150	feminir	75	24197.94	168	65.7	100.5	67	135	136	61	60	65	0.5718	0.5778
4	1 ou -	15 + tod	1.68	4		2500	feminir	30												
4	1.1-3	n,,o	1.64	3	40	4200	mascul	21	19724.2	183.6	80	121.5	70.5	83	150	54	83	76		
4	1.1-3	n,,o	1.6	1	38	1920	feminino		54092.79	158.1	114.5	128.5	76.5	82	175	52	92	187		0.5814
4	1.1-3	1-14 par	1.6	1	41	3520	masculin													
9	1.1-3	n,,o	1.46	1	39	3060	masculin													
3	1.1-3	n,,o	1.65	5	39	3700	feminir	20	16678.31	162.3	53.9	106	71.5	74	188	63	112	69	0.5821	0.5781
3	1.1-3	n,,o	1.49	1		3320	masculin		9108.364	173.15	68.7	119	68.5	114	186	51	73	277	0.5817	0.5887
10	1 ou -	n,,o	1.57	1	40	3070	masculin		46299.89	171.1	107	160	105.5	96	253	73	162	52		
7	1.1-3	n,,o	1.58	2	39	2250	feminir	29	29197.29	159.7	69.5	113	76.5	89	160	70	72	62	0.5799	
8	3.1-6	n,,o		1	42	3000	masculin		5327.07			145.5	99	84	180	67	93	90		
6	6.1-10	15 + tod	1.55	3	38	3000	mascul	169	16980.39	175.95	73.2	114	71.5	92	187	38	111	178	0.5786	0.5809
6	1.1-3	n,,o	1.55	1	37	3240	masculin													
4	1.1-3	n,,o	1.5	4		2720	mascul	59	8870.042	161.7	59.3	127	70.5	101	182	46	118	125	0.5902	0.5777
4	1 ou -	n,,o	1.52	2	42	3420	mascul	90	19058.52	172.2	77.4	118.5	72.5	105	209	57	124	176	0.5785	0.5769
9	1.1-3	n,,o	1.43	3		3800	mascul	22	28760.87	170.1	89.7	121	74	55	120	28	80	59		0.5708
3	1.1-3	15 + tod	1.6	6		2820	mascul	38												
9	1.1-3	1-14 par	1.59	2	41	2950	mascul	12												
10	3.1-6	n,,o	1.58	2	40	3150	mascul	12												
4	1.1-3	n,,o		5		3450	masculin													
0	1 ou -	n,,o	1.47	2		1800	masculin					176	103.5							

pescmae	prenda	pfumomae	paltmae	pgesta	pidgest	ppn	psex	pint	dmgtotdxa	daltura2012	dpeso	dsysmed	ddiamed	dglicose	dcolesterol	dhdl	dldl	dtrig	dECMICE	dECMICD
7	3.1-6	n„o	1.53	1	39	2900	masculin													
2	1.1-3	n„o	1.54	3	39	4180	mascul	83	16732.61	174.9	69.3	108.5	70.5	101	249	65	151	145		
4	1 ou -	1-14 tod	1.5	2	38	2750	feminir	24	18446.18	150.9	55	116.5	79.5	89	280	94	163	105	0.5781	0.5782
6	1.1-3	1-14 tod	1.49	2		2680	mascul	12												
5	1.1-3	n„o	1.51	3	37	2880	mascul	82												
5	1.1-3	n„o	1.61	1	39	3850	feminino					116	81.5	97	155	62	69	136		
9	3.1-6	n„o	1.61	3	39	2450	feminir	55												
3	1.1-3	1-14 tod	1.49	1		3300	masculin													
7	6.1-10	n„o	1.68	3	39	3850	feminir	18												
6	1 ou -	n„o	1.5	1	36	2810	masculin		10443	165.3	61.9	112.5	68	65	121	39	62	61	0.5726	0.541
1	1.1-3	15 + par	1.52	1		1980	masculin		20231.92	162.35	69.4	135.5	73.5	105	215	66	137	62	0.5434	0.5915
3	1.1-3	n„o	1.61	1		3700	feminino													
4	1 ou -	n„o	1.54	3		1910	feminir	41	13861.35	156	46.4	134	79.5	81	142	56	68	76	0.575	0.5772
5	3.1-6	1-14 tod	1.58	2	40	4150	mascul	75	22358.11	176.35	80.9	120.5	73.5	131	277	52	204	193		
3	3.1-6	n„o	1.59	2	38	3700	feminir	133	46982.04	160.35	105.3	137	94.5	79	208	65	127	64		
5	1.1-3	15 + tod	1.69	1		2900	masculin													
8	1.1-3	n„o	1.52	1		2840	feminino		40162.18	163.1	88.8	111	81	112	200	58	112	187	0.5786	0.5788
12	10	1-14 tod	1.67	1	37	2910	feminino		17470.9	165.9	57.1	104	71.5	87	221	98	100	89	0.5317	0.5116
15	3.1-6	n„o	1.59	1	41	3000	feminino		12621.19	150.5	49.1	109	68.5	76	230	89	115	120	0.5741	0.5601
5	3.1-6	1-14 tod	1.58	2	37	2000	mascul	14												
5	1 ou -	n„o		2		3250	mascul	14												
16	10	n„o	1.57	2	41	2800	feminir	46	23059.01	160.2	59	127	77	86	280	78	163	202		0.5764
5	1 ou -	1-14 par	1.57	3	42	2920	feminir	118	24634.57	168.9	69.6	111	69.5	87	173	49	108	77	0.5798	0.5802
5	1.1-3	1-14 par	1.63	1	39	3350	masculin													
5	1.1-3	n„o	1.61	1	40	3400	masculin		4385.44	182	63	123	68	64	209	85	106	102	0.5654	0.5774
16	6.1-10	n„o	1.55	1	38	2990	feminino			163.4									0.5744	0.5784
5	1.1-3	n„o	1.6	13	38	3370	feminino		23796.13	169.9	66.7	126	79.5	79	174	77	83	66	0.5762	0.58
2	1.1-3	n„o	1.64	3	39	3540	feminir	73	14379.14	171.35	64.1	130.5	87.5	95	176	72	79	126	0.5759	0.5901
0	1.1-3	n„o	1.5	6		2700	feminir	182	30508.49	164.2	70.2	118	75	60	160	55	87	76	0.5722	0.5753
7	1.1-3	n„o		8	42	3390	feminir	34	22140.17	161.35	62.5	135.5	82.5	87	160	69	67	130		
0	1 ou -	n„o	1.64	1		2950	masculin													
3	3.1-6	15 + tod	1.54	4		2580	feminir	136	48417.04	158.25	91.3	118.5	81.5	91	222	78	111	155		
4	1 ou -	15 + tod	1.54	1		1630	feminino		20286.33	152.7	55.8	136.5	93	71	120	42	68	67	0.6033	
17	3.1-6	n„o	1.58	2	37	3900	feminino		14461.78	161.6	49.2	106	60.5	52	180	78	90	46	0.5778	0.5728
5	1.1-3	n„o	1.62	3	41	3950	mascul	30												
9	1.1-3	15 + tod	1.55	2	38	3530	feminir	46	46313.57	169.5	93.8	113.5	68	97	140	65	64	52		
5	1.1-3	1-14 tod	1.53	2	38	2800	mascul	43	17959.05	170.65	74	130.5	76	79	185	47	116	95	0.6126	0.5812
1	1.1-3	15 + tod	1.53	6	40	2970	mascul	12	29552.01	171.25	85.4	128	87.5	92	169	47	94	162	0.5781	
4	1 ou -	n„o	1.48	2		3230	mascul	48	3074	166.8	59.3	141	77.5	103	161	66	81	67	0.5783	0.5736
17	6.1-10	n„o	1.57	1	43	4000	feminino		24499.84	163.9	65.8	110	81	89	196	58	123	94	0.5696	0.5601
7	1.1-3	1-14 par	1.57	2	42	4150	feminir	54	40713.78	164.3	81.5	121.5	79.5	83	185	72	95	76	0.5801	0.5782
1	1 ou -	n„o	1.48	5		3420	feminino		7509.532	156.8	44.7	99	64	96	146	39	77	135	0.5693	0.5752
9	3.1-6	1-14 tod	1.64	6	40	3560	feminir	30	50644.49	168.2	98	109	68	73	150	61	76	72	0.5774	0.5784
7	1 ou -	1-14 par	1.5	1	37	3200	masculin													
8	3.1-6	n„o	1.52	1	41	3100	masculin		25258.55	175.35	85.4	130.5	69	92	237	70	133	194	0.5813	0.576
9	1.1-3	1-14 tod	1.49	2	42	3050	mascul	25	13720.15	175.1	79.4	120.5	74	111	247	67	113	330	0.5709	0.5666
5	1 ou -	n„o	1.6	2	40	3000	mascul	12												
3	1.1-3	n„o	1.57	9		3550	feminir	32	6079.146	164	40.5	94	55	84	148	70	69	41	0.5734	0.5548

pescmae	prenda	pfumomae	paltmae	pgesta	pidgest	ppn	psex	pint	dmgtotdxa	daltura2012	dpeso	dsysmed	ddiamed	dglicose	dcolesterol	dhdl	dldl	dtrig	dECMICE	dECMICD
7	1.1-3	15 + tod	1.65	1	38	1800	masculin		16936.5	169.05	75.8	126.5	69	91	195	52	115	171	0.577	0.578
5	1.1-3	1-14 tod	1.55	3	40	3580	mascul	62	33128.8	169.5	91.9	141	92.5	106	237	66	130	244	0.5877	0.6274
4	1.1-3	n,,o	1.56	1	41	3100	masculin		39119.64	175.85	110.8	132.5	96.5	68	185	36	120	152	0.5797	0.581
5	3.1-6	n,,o	1.61	3	42	3350	feminir	48												
6	3.1-6	n,,o	1.59	2	38	2740	feminino													
4	1.1-3	1-14 tod	1.64	1	38	2600	masculin													
5	1.1-3	n,,o	1.6	7		3370	feminir	26	33675.35	165.1	81.1	112.5	76.5	73	145	54	78	73	0.5776	0.5732
9	1.1-3	1-14 tod	1.67	1	39	3910	masculin													
3	1.1-3	n,,o	1.57	10		2800	feminir	159	20195.13	161.7	57.8	104	72	105	237	61	160	92	0.578	0.5786
5	3.1-6	15 + tod	1.53	3	39	3030	feminir	74	20663.41	152.7	54.5	111.5	74.5	76	190	70	101	105	0.5807	0.5794
4	1 ou -	15 + tod	1.5	2	41	3200	feminir	20	10664.03	156.75	47.1	117.5	80	64	178	60	87	128	0.5791	0.5777
7	1.1-3	1-14 tod	1.5	2	40	2780	mascul	18												
0	1 ou -	n,,o	1.52	4	39	3800	mascul	27												
6	1.1-3	n,,o	1.53	1	41	3600	masculin													
0	1.1-3	n,,o	1.54	2	43	3400	mascul	20	14829.92	168.65	66.6	140.5	86	66	189	54	107	124	0.5795	0.5778
8	1.1-3	n,,o	1.53	2	41	3450	mascul	13	17665.41	170.5	79.4	127.5	74	78	233	66	150	95	0.6436	0.5922
17	6.1-10	n,,o	1.51	3	40	2560	mascul	36	8698.989	168.25	57.4	133	82.5	86	200	63	121	90	0.5786	0.5752
9	1 ou -	n,,o	1.6	1	39	2750	feminino													
1	1 ou -	n,,o	1.53	11	40	3400	feminir	49	16199.58	168	60	100.5	63	81	120	29	70	143	0.5783	0.5776
8	1.1-3	1-14 tod	1.6	2	39	4200	mascul	23	21738.75	170.5	89.8	120.5	64.5	90	170	50	103	110	0.5734	0.5808
13	6.1-10	n,,o	1.61	1	38	3250	masculin													
8	1.1-3	1-14 par	1.62	1	39	3500	masculin													
6	1.1-3	1-14 par	1.45	1	38	3350	feminino		21566.46	153.1	62.8	113.5	80	76	170	70	87	62		
6	1.1-3	n,,o	1.59	2	41	3500	feminir	91												
5	3.1-6	n,,o	1.56	4	39	3500	feminir	25	34706.77	157.3	69.7	112	70	80	144	70	61	48	0.5632	0.5773
1	1.1-3	n,,o	1.54	14	37	3250	mascul	79												
7	3.1-6	1-14 par	1.51	2	38	3000	feminir	67												
9	3.1-6	n,,o	1.61	1	42	3000	masculin		10204.6	174.2	73.7	133.5	81.5	95	144	48	70	119		
3	1 ou -	n,,o	1.57	2	38	3500	feminir	112												
17	6.1-10	15 + tod	1.61	2	40	2750	feminir	25												
5	6.1-10	n,,o		4	39	4100	feminir	90												
0	1.1-3	n,,o	1.53	2	39	2600	mascul	56	31269.87	172.7	97.4	137	86	82	208	52	141	85	0.5783	0.5853
6	1 ou -	1-14 tod	1.52	6	39	2260	mascul	25	6251.053	173.4	63.3	134	79.5	84	162	61	89	73	0.5776	0.5787
10	1.1-3	15 + tod	1.59	2	39	3220	masculin													
7	3.1-6	n,,o	1.57	2	38	2660	feminir	56	23866.04	170.6	61.1	114.5	78	67	242	84	131	166	0.5658	
5	1.1-3	n,,o	1.55	3		3500	mascul	11												
6	1 ou -	n,,o	1.53	1		2750	masculin													
6	1.1-3	n,,o	1.53	1	38	2850	masculin		19514.65	168.9	72.2	133	81.5	84	150	49	82	120	0.5771	0.5863
18	10	n,,o	1.61	2	41	3600	mascul	28												
16	10	n,,o	1.55	2	39	3400	feminir	12	18490.67	155.7	53.8	117.5	82.5	81	192	52	126	116	0.5781	0.5783
5	1.1-3	n,,o	1.56	1	39	3420	masculin		11938.53	172.15	70.1	108.5	54	81	160	57	79	115	0.5721	0.5768
6	1.1-3	n,,o	1.54	2	42	3200	masculin			173.9	125.5	127.5	77	104	230	52	148	172	0.5824	0.5794
5	1.1-3	n,,o	1.52	2	38	2970	feminir	117	26305.72	159.95	63.9	117	74.5	82	235	73	140	115	0.5669	0.5703
13	3.1-6	n,,o	1.61	1	40	3000	feminino													
11	3.1-6	n,,o	1.62	1	40	3720	feminino		32829.44	167	71.9	103	65.5	60	162	72	84	29	0.5696	0.5785
0	1.1-3	15 + par	1.53	2	39	2850	feminir	34	22515.24	152.7	56.5	127.5	87	86	200	70	118	56	0.5786	0.5808
1	1 ou -	1-14 par	1.5	1		3000	masculin		36038.06	164.5	95.2	115.5	72	81	225	69	144	76		0.5766
0	1.1-3	n,,o	1.59	4	39	3200	feminir	15	18141.51	157.5	54.2	115.5	72	97	160	56	88	68	0.579	0.5796

pescmae	prenda	pfumomae	paltmae	pgesta	pidgest	ppn	psex	pint	dmgtotdxa	daltura2012	dpeso	dsysmed	ddiamed	dglicose	dcolesterol	dhdl	dldl	dtrig	dECMICE	dECMICD
6	1.1-3	n„o	1.57	2	39	2550	mascul	16	31723.73	156.3	90.7	134	82	97	168	72	84	87	0.6661	0.6203
5	3.1-6	n„o	1.57	3	40	3800	mascul	46												
5	1.1-3	1-14 tod	1.49	2	42	2600	mascul	36	5816.239	176.45	59.8	123	81	84	149	49	92	37		
6	1.1-3	1-14 tod	1.49	1	41	3000	feminino		30622.29	162.4	69.1	138	90.5	71	191	65	114	48	0.622	0.5777
11	3.1-6	1-14 tod	1.66	1		2950	feminino		17491.25	161	55.1	124.5	77	86	140	59	65	78	0.5781	0.5783
3	1.1-3	n„o	1.51	3	39	2460	feminir	21												
0	1 ou -	n„o	1.54	8		3050	mascul	60												
3	1 ou -	1-14 tod	1.43	4	39	2950	mascul	42	22060.91	169.45	79.7	127.5	76	103	244	52	106	430		
5	1.1-3	1-14 par	1.52	2	40	3950	feminir	35	30415.92	152.85	71.5	110.5	73						0.5787	0.5873
0	1.1-3	n„o	1.58	8		3370	mascul	54												
4	1.1-3	n„o	1.52	1		3100	feminino		38378.38	150.2	75.8	116	77	105	235	67	149	125	0.5777	0.5724
4	1.1-3	n„o	1.57	4	41	3780	feminir	29	28552.24	167.45	78.1	132.5	83.5	101	210	47	150	87	0.5931	0.576
6	1.1-3	n„o	1.59	2	40	3200	mascul	24	21404.46	174.8	86.9	130	76.5	92	170	49	105	57	0.593	0.5857
9	1.1-3	1-14 par		2		2960	feminir	31												
7	1.1-3	n„o	1.39	2		2710	mascul	15	16667.87	166.55	63.2	128	72	95	263	54	155	291	0.5753	0.5785
10	10	15 + tod	1.6	2	39	2900	feminir	24	17545.8	155.9	54.7	117.5	73.5	85	193	62	112	109	0.5561	0.5722
10	6.1-10	1-14 tod	1.55	2	39	2550	mascul	73	21126.36	171.15	79.7	111	68	112	207	56	106	251	0.5796	0.5793
1	1 ou -	1-14 tod	1.47	1	36	2120	feminino		22433.64	153.35	60.8	114.5	66.5	92	161	67	79	83	0.6062	0.5796
5	3.1-6	n„o	1.6	4	38	3250	masculin		20727.36	171.85	83.2	119.5	73.5	83	239	71	142	145		
3	1 ou -	n„o	1.68	2	41	4150	mascul	15		196.3	87.3	113.5	64	106	165	47	90	134	0.5802	0.5786
0	1.1-3	n„o	1.54	2	38	3650	feminir	44												
3	1 ou -	n„o	1.54	2	39	2750	feminir	24	22913.3	154.95	54.5	102	64.5	76	189	82	80	129	0.5677	
8	3.1-6	n„o	1.65	2		3670	feminir	25	23113.69	168.5	62.3	111.5	72	76	210	53	135	147		
7	1 ou -	1-14 tod	1.64	1	38	3750	feminino													
0	1 ou -	n„o	1.54	4	39	2820	mascul	45	26619.08	172.1	89.3	151	85.5	94	195	49	128	94	0.5785	0.5796
3	1.1-3	n„o	1.45	1	41	3800	feminino													
3	1.1-3	n„o	1.63	1		2800	feminino													
6	1 ou -	1-14 tod	1.5	12	37	2750	mascul	45	18571.27	168.3	73.1	122.5	75	93	202	43	103	268	0.5853	0.5778
12	6.1-10	n„o	1.45	3	40	2950	mascul	13	16575.89	177.7	76.8	114	77.5	70	186	51	122	77	0.5526	0.5739
5	1 ou -	n„o	1.54	1	39	3000	masculin													
4	1 ou -	1-14 par	1.56	2	37	3250	mascul	41												
7	1 ou -	n„o	1.66	1	39	3150	feminino		28545.27	168.55	78.8	109.5	75.5	235	245	62	166	164	0.6173	
0	1.1-3	n„o	1.62	2	39	2900	mascul	34	25941.24	188.45	98.5	118	75	81	238	48	150	277	0.6024	0.5815
6	1 ou -	n„o	1.46	1	37	3250	masculin													
12	1.1-3	n„o	1.55	12	38	2920	feminino													
12	6.1-10	n„o	1.65	1	38	3830	masculin													
12	3.1-6	n„o	1.5	1	39	3500	feminino													
0	1 ou -	n„o	1.59	4		3300	mascul	16												
15	6.1-10	n„o	1.56	3	40	3050	feminir	24												
5	1 ou -	1-14 tod	1.55	3	38	3150	mascul	22				112.5	68							
3	1.1-3	n„o	1.63	4	39	3710	mascul	22												
7	1 ou -	15 + tod	1.57	5	42	2460	feminir	14	17925.75	161.65	52.6	105	71	86	162	60	91	65	0.5694	0.5571
15	3.1-6	n„o	1.56	2	40	3170	feminir	74	14514.02	164.35	58.1	108.5	63	87	170	82	77	48	0.5737	
4	1.1-3	n„o	1.54	1	40	4050	feminino													
4	1.1-3	n„o	1.58	3		3650	mascul	56												
5	1.1-3	1-14 par	1.52	3	40	3000	feminir	24	21890.56	153.65	59.2	135.5	81.5	85	178	47	107	77	0.5845	0.579
10	3.1-6	1-14 tod	1.6	3	41	3300	feminir	13												
9	1.1-3	n„o	1.57	2	36	2950	mascul	44	34707.03	185.5	111	136.5	81	99	170	45	95	166		

pescmae	prenda	pfumomae	paltmae	pgesta	pidgest	ppn	psex	pint	dmgtotdxa	daltura2012	dpeso	dsysmed	ddiamed	dglicose	dcolesterol	dhdl	dldl	dtrig	dECMICE	dECMICD
5	3.1-6	n„o	1.5	3		3130	feminir	61	11935.73	161.9	54.5	123	75.5	157	206	69	114	126	0.5808	0.5807
16	3.1-6	1-14 tod	1.5	2		2520	mascul	12	11259.85	166.2	61.2	115	64	88	176	52	93	160	0.5622	0.5775
7	1.1-3	n„o	1.51	1	39	3570	masculin													
6	1 ou -	n„o	1.62	2		3600	masculin			182.4	149.6	147	99	88	239	58	160	130	0.612	0.5973
6	1.1-3	n„o		10		3150	feminir	49	23433.44	166.9	65.4	110.5	69	78	319	62	229	127	0.579	0.5787
11	1.1-3	n„o	1.48	1	41	3800	masculin		11700.81	162.25	64.4	113.5	68.5	83	175	55	99	122	0.5822	0.5995
0	1 ou -	15 + tod	1.55	7	40	3400	mascul	41	3323.882	176.2	58.4	129.5	80	105	138	56	73	40	0.5787	0.5771
6	3.1-6	15 + tod	1.61	3	37	2680	feminir	43	41109.63	163.85	87.2	110.5	73.5	104	226	55	134	253	0.5736	0.5783
2	1 ou -	n„o	1.6	6	42	3540	feminir	50	20674.64	154.75	53.6	92	62	75	153	60	79	40	0.5789	0.5787
1	1.1-3	n„o	1.5	4	39	4050	mascul	44												
12	6.1-10	n„o	1.71	2	40	3900	feminino		20123.04	169.95	63.4	127	86.5	63	158	77	62	92		
6	1.1-3	1-14 tod	1.55	1	40	3500	masculin													
2	1.1-3	15 + tod	1.53	6	42	3250	feminir	25												
4	1.1-3	n„o	1.52	2	42	4050	mascul	105	28596.47	173.2	96.6	130	84	66	175	39	119	105		
0	1.1-3	n„o	1.48	1		2750	feminino			151.7	109.9	133	91	164	330	72	213	146		
10	1.1-3	n„o	1.54	5		3470	mascul	43												
10	3.1-6	n„o	1.7	2	37	3000	mascul	25												
12	1.1-3	15 + tod	1.64	1	39	2960	masculin													
5	3.1-6	15 + tod	1.59	2	37	2660	mascul	52												
5	1.1-3	n„o	1.59	2	38	3300	feminir	39	23553.75	166.3	66.5	105	67.5	79	251	52	169	132		0.5915
7	1.1-3	1-14 tod	1.52	3	37	2800	feminir	42		162.4	124.4	133	94	79	230	58	153	117	0.7595	
10	10	n„o	1.57	1	39	3650	masculin		43571.97	177.2	100.8	123	85	95	270	47	182	210	0.581	0.641
4	1.1-3	n„o	1.65	1	37	2800	masculin													
0	1.1-3	n„o	1.55	7	43	3550	feminir	20												
4	1 ou -	1-14 tod	1.58	3		3600	mascul	52	14808.18	175.5	83.7	145.5	87	77	170	66	87	56	0.5775	0.578
3	1 ou -	n„o	1.49	6	42	3140	feminir	79												
2	1 ou -	n„o	1.6	3	35	2730	mascul	60		175	84.4	123	80.5	89	214	47	119	278	0.7127	0.6921
11	3.1-6	n„o	1.62	2		3110	feminir	45	22959.24	171.15	66.3	103	65.5	82	182	80	83	98	0.5774	0.5789
15	10	1-14 par	1.56	1		3040	masculin													
3	1.1-3	n„o	1.57	2	38	3600	mascul	40	28555.84	179.7	97.7	136.5	66.5	90	179	56	91	145	0.5918	0.5784
6	1.1-3	n„o	1.51	3	38	3430	mascul	42	35102.3	169.6	88.2	125	83	83	212	61	125	152	0.5783	0.5583
5	1.1-3	n„o	1.58	3		3380	mascul	78												
10	1.1-3	n„o	1.64	1	36	3400	feminino		25461.19	165.35	69.3	113.5	73	99	148	60	75	59	0.5798	0.5783
9	1.1-3	15 + tod	1.61	3	38	3300	mascul	63	16525.83	173.55	74.8	116	68	84	206	37	140	196	0.5793	0.5786
1	1.1-3	n„o	1.61	1		3570	masculin		19192.97	183.6	80.6	117.5	73.5	76	213	60	145	61	0.5955	
7	3.1-6	1-14 tod	1.52	3	39	3650	feminir	29	16616.82	170.6	60.8	102	68	73	203	88	97	49	0.5795	0.5773
5	1.1-3	n„o	1.47	6		3250	feminir	58	17459.33	159.1	52.9	106	73.5	59	158	64	80	72		
18	10	n„o	1.68	1	42	3350	masculin													
4	6.1-10	n„o	1.47	4	41	3650	feminir	14	33104.2	153.6	75.1	111	71	83	174	70	90	53	0.5708	0.5793
5	1.1-3	n„o	1.51	4	42	3580	feminir	16	24958.28	159.25	63	112	70.5	81	236	99	121	59	0.5783	0.5788
1	1.1-3	n„o	1.6	2	41	3450	feminir	31												
5	1.1-3	n„o	1.59	6	37	2660	mascul	89	22738.1	185.55	85.2	132	69.5	115	166	74	71	104		
4	1.1-3	1-14 tod	1.49	3	39	3650	mascul	74	20039.33	163.15	75.3	130	88	106	241	40	120	496	0.5737	0.5795
8	3.1-6	n„o	1.61	1	39	3620	feminino		47394.27	166.15	95.2	118	70.5	81	147	62	63	65	0.5762	0.5798
4	3.1-6	n„o	1.65	2	42	3580	feminir	127												
12	1.1-3	n„o	1.51	1	40	3300	masculin		39842.25	164.3	102.2	141.5	86	143	194	48	107	242	0.5898	0.5844
5	3.1-6	n„o	1.64	3	39	3800	feminir	71	11106.41	168.45	51.7	105.5	64	85	148	66	68	40	0.578	0.579
5	1 ou -	1-14 tod	1.46	5		3240	feminir	18												

pescmae	prenda	pfumomae	paltmae	pgesta	pidgest	ppn	psex	pint	dmgtotdxa	daltura2012	dpeso	dsysmed	ddiamed	dglicose	dcolesterol	dhdl	dldl	dtrig	dECMICE	dECMICD
4	1 ou -	1-14 tod	1.6	4		3100	feminir	24												
5	1 ou -	15 + tod	1.58	2	39	2530	mascul	80	13474.21	158.75	64.6	149.5	79.5	106	228	50	138	212	0.6055	0.6337
2	1.1-3	n„o	1.59	3	39	3830	mascul	57												
14	3.1-6	n„o	1.55	1	37	3240	masculin													
4	1.1-3	n„o	1.53	1	42	2900	masculin		15278.72	171.05	63.1	124	69.5	94	195	42	118	186	0.5486	
1	1.1-3	15 + par	1.62	6		3300	feminir	11												
7	1.1-3	1-14 tod	1.61	1		2910	feminino													
7	1.1-3	n„o	1.58	3	40	3430	feminir	96	31187.06	160	75.2	117	77.5	89	197	79	110	53		
3	1.1-3	n„o	1.51	9	40	3000	mascul	31												
7	1.1-3	n„o	1.72	2	38	3500	feminir	30												
2	1 ou -	1-14 tod	1.57	1	43	2890	feminino		51191.79	157	100.7	136.5	87.5	105	185	60	103	85	0.5598	0.574
5	1 ou -	1-14 par	1.53	1	41	2720	feminino		19331.18	155.85	51.7	120.5	75.5	60	250	79	155	73	0.5603	0.5889
4	1 ou -	n„o	1.54	3	42	3550	feminir	62		143.7		116	79						0.6029	0.5817
4	1 ou -	1-14 tod	1.51	2	38	2730	feminir	39	20706.8	147.35	53.1	88.5	56	67	228	56	160	64		
7	1 ou -	n„o	1.64	3	40	3800	feminir	30	17896.61	166.3	61.8	113.5	65.5	82	200	77	104	72	0.5639	0.5427
8	1 ou -	1-14 tod	1.6	1	35	2200	feminino		20388.3	158.6	51.8	104	72	62	142	55	75	71	0.5633	0.578
8	1.1-3	n„o	1.51	2		2880	mascul	11	26978.85	176.65	91	123	69	72	203	43	134	125	0.6665	0.6002
6	1.1-3	n„o	1.61	2	38	3870	feminir	123												
4	1 ou -	n„o	1.5	2	35	2280	feminir	11	31838.96	152.85	69.5	115.5	77	79	160	64	81	78		
7	1.1-3	1-14 par	1.63	1	42	4000	feminino													
5	1 ou -	n„o	1.46	3	39	3250	mascul	48												
3	1 ou -	1-14 tod	1.49	3	38	3050	mascul	16	15995.81	168.55	67.5	114	71.5	119	189	37	95	296	0.5824	0.5927
5	1.1-3	n„o	1.56	1	37	2450	feminino		27407.79	174.7	72.3	118	77	93	167	56	95	58	0.5781	0.5783
5	6.1-10	n„o	1.56	4	39	3600	feminir	50												
12	6.1-10	1-14 tod	1.52	2	40	3100	feminir	62												
5	6.1-10	n„o	1.59	3	38	2900	feminir	32												
6	1.1-3	1-14 tod	1.58	3	39	2730	feminir	19	51680.3	158.65	92.3	117	73	84	182	90	72	105	0.5762	0.5777
5	1.1-3	n„o	1.62	2	39	3670	mascul	86	36354.95	179.05	106.2	136	83	80	235	44	145	256	0.5706	0.5792
3	1.1-3	n„o	1.58	3	42	3460	mascul	108	21468.03	178.05	79.9	124	77	74	159	34	108	80	0.5593	0.5721
7	1.1-3	n„o	1.6	3	40	4300	feminir	28	25608.29	170.25	65.7	105	68.5	68	130	65	56	50		
1	1.1-3	1-14 tod	1.54	4	40	3100	mascul	51												
2	1 ou -	1-14 tod	1.53	2	41	3400	feminir	33												
5	1.1-3	n„o	1.6	1	39	3250	masculin													
5	1.1-3	n„o	1.53	2	39	2520	feminir	26												
3	1.1-3	n„o	1.55	1	39	2720	feminino													
4	1.1-3	1-14 tod	1.56	5	38	4000	mascul	42												
4	1 ou -	n„o	1.63	4	41	2750	feminir	22	18650.54	164.35	55.7	120	90.5	74	160	98	48	54	0.5637	0.5787
3	1.1-3	n„o	1.53	8	38	2650	feminir	16	12810.01	155.5	47.5	122	74	123	198	68	120	67		0.5751
4	1.1-3	n„o	1.63	3		3250	masculin													
6	1.1-3	n„o	1.56	1	40	3580	masculin		23142.06	177.1	83.9	125.5	71	79	151	63	78	85	0.5768	0.5772
6	1.1-3	n„o	1.63	2	39	3180	masculin													
8	3.1-6	15 + tod	1.63	3	40	2800	feminir	44												
12	1.1-3	1-14 par	1.57	2	38	2750	feminir	23	16301.22	164.05	54.7	108	66.5	92	184	99	68	80	0.5715	0.5781
16	10	n„o	1.74	2		3700	masculin		19097.15	177.9	81.9	106.5	61.5	65	188	39	120	200	0.5778	0.5758
0	1.1-3	1-14 tod	1.46	3		3040	feminir	23	26697.15	151.2	63.9	108	66	98	182	49	116	60		0.5745
6	1.1-3	1-14 par	1.49	2	40	3350	feminir	16	27588.18	165	69.4	116.5	75.5	77	187	64	98	112	0.5785	0.5849
12	6.1-10	n„o	1.7	4	39	4600	mascul	21												
5	1.1-3	n„o	1.55	3	39	2990	mascul	123	12809.51	169.5	67.1	129.5	86	73	163	48	102	114		

pescmae	prenda	pfumomae	paltmae	pgesta	pidgest	ppn	psex	pint	dmgtotdxa	daltura2012	dpeso	dsysmed	ddiamed	dglicose	dcolesterol	dhdl	dldl	dtrig	dECMICE	dECMICD
14	10	n„o	1.56	1	42	2700	masculin													
4	1 ou -	1-14 tod	1.51	1		3360	masculin		19200.82	173.9	76.2	126	85.5	71	264	61	161	245	0.5751	0.5666
12	3.1-6	1-14 par	1.55	3	39	3100	feminir	62												
7	1.1-3	15 + tod	1.61	1	42	3200	masculin		25336.48	169.95	82.4	129.5	77	123	240	40	122	438	0.5762	0.5782
16	6.1-10	n„o	1.69	3	40	3300	feminir	18	12023.66	169.1	50.8	97.5	59.5	89	135	49	76	43	0.5793	0.6162
3	1.1-3	1-14 tod	1.6	1	41	3100	masculin		17992.08	180.1	87.4	125	67.5	70	208	40	121	208	0.5794	0.5783
16	10	n„o	1.51	1	41	3220	feminino		18580.13	159.4	54.7	106	62	89	182	66	107	60	0.5603	0.5652
19	3.1-6	1-14 par	1.56	1		2650	feminino													
0	3.1-6	n„o	1.67	1	41	4850	masculin		35231.71	185.05	109.1	133	77	101	217	55	137	105	0.5878	0.5796
2	1.1-3	1-14 par	1.54	2	41	3260	feminino							99	180	35	108	187		
2	3.1-6	1-14 par	1.56	1	40	3220	masculin		25438.19	177.45	88.4	133.5	81	79	136	40	78	92	0.5649	
16	6.1-10	n„o	1.58	1	39	4460	feminino													
5	1.1-3	1-14 par	1.64	5	41	4220	mascul	31		181.9	152.7	134.5	90.5	85	95	31	52	40	0.5801	0.5876
6	1 ou -	n„o	1.48	3	39	2940	mascul	74	47015.05	170.2	113.9	142	95	73	216	51	139	137	0.5801	0.6046
0	1 ou -	1-14 tod	1.63	3		2970	masculin													
16	6.1-10	1-14 tod	1.55	2	42	3140	feminir	30	13877.91	150.15	47.1	116	74	81	200	83	104	59	0.5484	0.5736
12	1.1-3	1-14 par	1.61	3	38	2570	mascul	18	32138.33	175.4	98.1	118.5	67	90	196	62	116	87	0.5897	
8	3.1-6	n„o	1.61	2	39	4100	mascul	43												
7	1.1-3	n„o	1.46	3	38	3150	mascul	20	16472.24	168.15	72.4	136	87.5	94	190	60	108	103	0.5833	0.5769
1	1 ou -	n„o	1.65	2	39	3540	feminir	53	12270.3	172.6	58.1	112.5	77	91	128	68	52	51		
11	10	n„o	1.59	3		3220	feminir	34	20882.18	162.55	60.2	104	67	95	177	69	92	79	0.5826	
9	6.1-10	n„o	1.66	2		3720	mascul	99	21135.57	180.6	86.8	132.5	67.5	69	240	67	150	112	0.5584	0.59
6	1 ou -	1-14 tod	1.51	1	41	2700	masculin		27563.9	173.85	84	112	77.5	101	180	53	115	52	0.598	0.5791
12	3.1-6	1-14 tod	1.57	2	42	3160	feminir	34	30157.86	158.85	66.2	113	74.5	91	223	85	115	101	0.5772	0.578
12	1.1-3	n„o	1.6	7	39	3400	mascul	213	7056.068	173.7	62	113.5	65	82	140	50	70	66	0.5778	0.5635
14	3.1-6	1-14 tod	1.54	1	39	3020	feminino		29068.35	152.6	65.1	110.5	75.5	104	185	78	87	110	0.5677	0.5769
9	1.1-3	n„o	1.58	1	40	3050	feminino		52518.35	157.7	102.1	142.5	90	79	215	78	119	74	0.5558	
5	1 ou -	1-14 tod	1.63	1	40	2550	feminino													
5	1.1-3	n„o	1.51	2		750	feminir	214												
14	10	15 + par	1.55	1		3150	masculin		31528.13	177.95	88.4	128.5	73.5	126	190	66	97	136	0.5866	0.5839
12	3.1-6	1-14 par	1.6	2	41	3030	feminir	20												
9	1.1-3	n„o	1.55	3	38	3710	feminir	36	28078.16	173.4	71.7	114.5	64.5	92	167	72	88	42	0.5684	0.575
8	1.1-3	n„o	1.6	4	41	2600	feminir	26	48023.53	152.6	96.9	121.5	83.5	111	205	41	84	443	0.5885	0.5816
5	1.1-3	n„o	1.54	1	40	3000	feminino			168.4	126.4	113	68.5	79	174	58	85	157	0.5837	0.5753
7	3.1-6	n„o	1.57	1	38	3350	masculin		10282.02	166.2	63.1	107.5	61.5	78	190	64	96	146	0.5777	0.611
9	3.1-6	n„o	1.56	4	39	2850	feminir	81	21470.34	161.55	65.9	104.5	67	69	157	41	100	89		0.5677
7	6.1-10	n„o	1.51	1	42	3150	masculin													
5	1.1-3	1-14 par	1.58	3	35	2000	feminir	18	29668.56	161.4	78.5	118	74	89	208	53	120	163	0.578	0.5783
4	1 ou -	1-14 tod	1.58	3	41	2900	feminir	38												
8	1.1-3	1-14 par	1.55	3	35	1810	mascul	33												
2	1.1-3	1-14 par	1.5	1	38	3450	masculin		24076.5	170.9	91.5	128.5	81.5	97	247	50	150	261	0.5788	
11	1.1-3	n„o	1.55	1	40	3000	feminino		4914.06	157.4	39.7	106.5	67.5	87	179	72	89	54	0.5768	0.556
5	3.1-6	1-14 tod	1.65	2	38	3200	mascul	44												
4	1.1-3	1-14 tod	1.41	1		2600	masculin		18885.15	154.75	67.9	117.5	72	91	199	62	120	131	0.5706	
2	1 ou -	n„o	1.57	11	40	3180	feminir	45	26040.36	158.85	68.3	105	74.5	90	172	72	90	79	0.5782	0.5746
13	6.1-10	n„o	1.51	3	39	3950	feminir	41	30923.64	159.75	67.6	110	69	88	200	80	96	157	0.578	0.5787
9	3.1-6	1-14 par	1.5	1	38	3380	masculin		31009.31	183	89	135.5	81	84	232	76	139	92	0.5837	0.583
5	1.1-3	1-14 par	1.57	2	38	3720	mascul	52												

pescmae	prenda	pfumomae	paltmae	pgesta	pidgest	ppn	psex	pint	dmgtotdxa	daltura2012	dpeso	dsysmed	ddiamed	dglicose	dcolesterol	dhdl	dldl	dtrig	dECMICE	dECMICD
15	1.1-3	1-14 tod		1	40	4200	masculin		26801.37	174.1	90	128	78	75	220	45	160	85	0.5805	0.5642
0	1 ou -	15 + tod	1.52	8	34	1770	mascul	10												
6	1 ou -	1-14 tod	1.6	1	38	2850	feminino		19340.42	156	56.2	109.5	72.5	116	203	78	110	94	0.58	0.56
3	1 ou -	1-14 tod	1.52	2	40	2890	feminir	48	28182.47	159.2	64.9	111.5	72	84	184	52	112	113	0.5766	
14	3.1-6	n„o	1.58	2		2100	feminir	64												
5	1.1-3	1-14 tod	1.52	3	40	3080	mascul	35												
2	1 ou -	1-14 par	1.66	3		3050	feminino													
9	1.1-3	1-14 tod	1.52	8	41	4310	feminir	102	37792.71	164.45	78.6	108	80	82	176	68	93	87		0.5705
3	1.1-3	n„o	1.53	2		1720	feminir	10	21764.94	156.6	60.1	112.5	70	95	180	46	91	245		
5	1 ou -	1-14 tod	1.59	1		3450	feminino		16677.42	175.3	63.5	109.5	68.5	85	153	57	73	112	0.5778	0.5759
5		n„o	1.53	2	40	3700	feminir	88	27055.82	158.7	67.8	105	76.5	88	225	62	153	63		
5	1.1-3	n„o	1.56	3	35	2000	mascul	110	32756.95	179.2	101.5	131	84.5	84	159	56	92	53	0.5779	0.5737
7	1.1-3	1-14 par	1.62	1	41	3500	feminino													
9	1.1-3	n„o	1.6	1	36	2550	feminino		16992.49	169.2	56.9	100	61	76	192	76	106	64	0.5794	0.5752
4	1.1-3	n„o	1.63	2		3720	feminir	149	27467.26	160.8	73.6	101.5	66	83	168	53	105	88	0.5723	0.5514
8	1.1-3	n„o	1.51	1		4000	masculin		37288.21	178.5	108.4	135	89	104	220	58	127	304	0.5805	
3	3.1-6	15 + par	1.47	4	39	2850	mascul	28	14770.12	171.6	78.4	116	67	109	186	46	102	175	0.5795	0.5797
7	1.1-3	n„o	1.55	1		2780	feminino			152.5										
5	1 ou -	n„o	1.48	1	40	3150	masculin													
3	1 ou -	n„o	1.46	2		3230	mascul	11	4005.297	164.3	61.1	99.5	63	74	130	56	66	52	0.5785	0.5624
11	6.1-10	n„o	1.58	2		3800	feminir	12	38981.8	163.9	75	128	87.5	107	231	98	116	63	0.5786	0.5771
5	1.1-3	n„o	1.51	4	39	3650	mascul	168	24372.76	167.65	79.6	120.5	67.5	94	170	57	101	48	0.5984	0.5782
16	10	n„o	1.65	3	38	3250	masculin													
2	1.1-3	n„o	1.51	2	40	2800	mascul	15												
4	1 ou -	n„o	1.58	1	39	3750	masculin		21638.29	169.3	80.2	131.5	74	63	159	50	96	62	0.5801	0.5777
4	1.1-3	n„o	1.5	7	41	3200	feminir	47		154.9		99	56.5						0.5761	0.5798
6	3.1-6	n„o	1.52	3	41	3620	feminir	28	45564.87	157.6	91.8	115	73	80	214	64	113	231	0.5776	0.5804
5	1.1-3	1-14 tod	1.6	2	37	2850	feminir	14		158		105	63						0.5757	0.5791
6	1.1-3	15 + tod	1.54	2	39	3100	feminir	48	16090.29	165.9	52.6	120.5	75	84	146	64	70	46	0.5805	0.5604
4	1.1-3	n„o	1.6	4	43	3440	feminir	18						93	185	36	111	187		
6	1.1-3	15 + tod	1.55	2		3100	feminir	28	27580.59	155.2	69.2	111.5	74	88	156	46	95	84	0.5942	0.5789
10	3.1-6	n„o	1.48	1	41	2820	masculin		12264.21	164.5	54.5	112.5	73	51	209	60	140	51		0.5658
7	3.1-6	n„o	1.53	2	39	2670	mascul	16												
0	1 ou -	n„o	1.5	5	39	3500	feminir	23	24201.29	165.3	69.8	119	74.5	87	133	35	88	41	0.5818	0.5805
10	1.1-3	1-14 tod	1.52	1		2670	masculin													
5	1 ou -	15 + tod	1.49	3	38	2650	mascul	38												
5	1 ou -	1-14 tod	1.53	2	39	2900	feminino													
5	3.1-6	n„o	1.5	1	39	3200	masculin		22924.49	169.55	75.6	132	85	134	207	37	85	500	0.578	0.5842
5	1.1-3	1-14 tod	1.62	4	40	2980	feminir	51												
4	1.1-3	n„o	1.62	1		4100	feminino		46614.59	159.15	98.3	122	82.5	91	231	60	141	159	0.56	
7	3.1-6	1-14 tod	1.63	2	38	3150	mascul	10		182.8	136.2	143.5	85	353	200	41	68	662		
10	10	n„o	1.61	1	38	2900	masculin													
17	6.1-10	n„o	1.52	1	39	3110	feminino		20131.38	148.05	59.6	133.5	81							0.5803
0	1.1-3	n„o	1.46	8		1750	feminir	19												
0	1.1-3	n„o	1.58	6	41	3700	mascul	19	20785.49	159.55	79.6	116.5	71	84	174	53	100	108	0.5729	0.5798
5	1 ou -	1-14 tod	1.59	3	39	2900	mascul	33												
4	1.1-3	1-14 tod	1.54	2	39	3160	masculin		22059.43	162	79.7	131	80.5	100	168	48	106	62		0.5776
5	3.1-6	n„o	1.65	2		3100	feminir	110	14881.67	160.5	55.4	108	71.5	63	145	70	60	86	0.5781	0.5777

pescmae	prenda	pfumomae	paltmae	pgesta	pidgest	ppn	psex	pint	dmgtotdxa	daltura2012	dpeso	dsysmed	ddiamed	dglicose	dcolesterol	dhdl	dldl	dtrig	dECMICE	dECMICD
2	1.1-3	n„o	1.57	4		3450	feminino		54237.49	161	103.8	119.5	71	78	244	64	150	143	0.559	
8	10	n„o	1.48	3	39	3100	feminir	54	23681.88	146	56.7	127	71	91	243	78	140	125		
5	1.1-3	n„o	1.59	5	38	3840	feminir	25	28678.29	171	70.6	113.5	68.5	84	250	66	165	85	0.5818	0.578
5	1.1-3	n„o	1.51	2	41	3950	feminir	19	29384.68	161.95	69	96.5	57.5	85	189	70	99	118	0.5764	
0	3.1-6	1-14 tod	1.54	3		2950	feminino		45076.82	157	89.4	112.5	73.5	75	165	52	100	56	0.578	0.59
3	1.1-3	n„o	1.5	1	40	2980	feminino		10978.79	154.65	47.5	103	64.5	71	166	58	87	96	0.5638	0.5778
4	1.1-3	n„o	1.54	2	40	2500	mascul	80	32724.96	176.8	97.8	109	69.5	84	183	56	113	91	0.5792	0.6747
10	1.1-3	n„o	1.53	1	43	3300	masculin		26866.6	179.35	88.8	120	66	67	253	47	175	125	0.5817	0.6078
7	1 ou -	1-14 tod	1.62	1		3150	feminino													
5	1.1-3	1-14 tod	1.56	3	40	2220	mascul	71	17706.18	167.05	76.2	117	70.5	87	179	55	112	77		
10	1.1-3	n„o	1.66	2	40	3270	feminir	24	55065.28	168.6	104	119.5	79	97	217	75	124	102	0.5709	0.5624
13	6.1-10	1-14 tod	1.56	1	38	2360	feminino		13693.65	148.6	45.2	108	64	71	144	61	72	44	0.5783	0.5739
7	3.1-6	n„o	1.56	2	38	3350	mascul	42	31758.12	175.2	102	123.5	71.5	88	182	36	126	124	0.6245	0.679
7	1.1-3	n„o	1.5	1	39	2630	masculin		7441.775	176.1	63.1	135.5	71	123	206	60	140	52	0.5861	0.5725
8	1.1-3	n„o	1.67	1		2700	feminino													
2	1.1-3	n„o	1.63	3	39	4250	feminir	97												
9	10	n„o		6		3050	feminir	22	40778.8	179.5	83.6	108	71	80	169	75	82	45	0.5916	0.5721
6	1 ou -	1-14 tod	1.59	2	38	3000	feminir	53	32587.54	160.3	72	117	76	95	243	80	136	184	0.57	0.56
5	1.1-3	n„o	1.6	1	40	2800	masculin		12354.65	176.75	73.9	123	63.5	72	179	61	95	184		
4	1 ou -	n„o	1.41	5	35	2350	mascul	48	14311.62	166.35	66	111.5	70.5	91	225	66	136	125	0.6194	0.5766
4	1.1-3	n„o	1.56	1	40	3400	feminino													
3	1 ou -	n„o	1.57	2	39	3770	masculin		31809.56	173.25	92.3	128	81.5	99	234	54	159	99	0.581	0.5781
8	1.1-3	n„o	1.53	1	41	2910	masculin		35223.77	174.2	99.9	107	65.5	87	160	53	99	63	0.6756	0.6668
7	1.1-3	n„o	1.69	1	37	2880	masculin													
3	1 ou -	n„o	1.57	2	38	3250	feminir	17	16341.41	154.85	56.2	119.5	78.5	80	198	89	94	43	0.5629	0.5714
9	3.1-6	1-14 par	1.58	1		2870	feminino													
11	6.1-10	n„o	1.64	1	39	2600	masculin		12340.08	172.9	69	121	76	101	190	48	103	205	0.5798	0.5778
12	3.1-6	1-14 par	1.62	1	42	3650	feminino													
8	1.1-3	n„o	1.6	1	41	3400	masculin													
4	1.1-3	1-14 tod	1.42	1	38	2900	masculin		22930.75	177.9	87.2	133.5	89	89	198	49	124	143	0.5783	0.578
5	1 ou -	n„o	1.64	1	41	3780	feminino		35947.51	162.95	83.5	120.5	69.5	103	172	68	87	88	0.6538	0.5973
15	3.1-6	15 + par	1.56	4	40	3000	feminir	31												
13	10	15 + tod	1.55	3	39	3950	feminir	32	16018.11	157.9	57.8	135.5	79.5	71	175	68	89	90	0.5781	0.5792
10	3.1-6	n„o	1.59	1	38	2970	masculin													
8	1.1-3	n„o	1.53	2	40	3150	mascul	46												
5	1 ou -	n„o	1.55	3	39	3460	mascul	52	19037.13	176.55	79.1	141.5	87	80	194	63	117	51	0.5756	0.546
3	1 ou -	n„o	1.49	2	42	3000	mascul	13	34448.2	174.2	88.4	134	79	107	248	64	168	99	0.5853	0.5771
8	3.1-6	1-14 par	1.53	2	41	3370	masculin		21677.22	171.55	76.6	112.5	62.5	93	178	74	82	81	0.5782	0.5696
15	10	n„o	1.6	1	42	3680	feminino		19283.99	156.7	54.5	102.5	65	91	174	78	82	45	0.578	0.5744
7	3.1-6	1-14 tod	1.68	5	39	4000	mascul	19	10134.55	174.3	59.5	123	74.5	337	255	86	150	65	0.578	0.58
3	1.1-3	1-14 tod	1.59	1	39	2780	feminino		42756.39	166.55	85.4	122	74.5	87	190	63	102	155	0.5777	0.5475
9	3.1-6	n„o	1.57	1	38	3150	masculin		31002.21	166.8	102.6	142.5	81.5	91	217	48	128	406	0.5863	0.5855
8	1.1-3	n„o	1.49	1	36	3150	masculin													
11	3.1-6	n„o	1.47	1	38	3450	masculin		12831.68	168.8	59.3	131.5	74.5	99	175	51	104	98	0.562	0.5755
0	1.1-3	1-14 tod	1.56	2		3240	mascul	40	22781.98	174.5	88.2	155	98	81	231	65	141	109	0.6758	0.6126
10	1.1-3	n„o	1.47	2	37	3060	feminir	31												
3	1.1-3	1-14 tod	1.44	7		2340	feminir	69	31989.28	148.3	69.4	108	70.5	77	187	60	119	74		
14	6.1-10	n„o	1.58	1	42	4850	masculin		43677.24	174.15	112.8	119.5	78	87	135	44	71	109	0.5801	0.5791

pescmae	prenda	pfumomae	paltmae	pgesta	pidgest	ppn	psex	pint	dmgtotdxa	daltura2012	dpeso	dsysmed	ddiamed	dglicose	dcolesterol	dhdl	dldl	dtrig	dECMICE	dECMICD
12	1.1-3	1-14 tod	1.55	3	40	3750	mascul	54	12568.78	166.5	66.1	115	75.5	90	169	70	90	73	0.5764	0.5784
6	3.1-6	1-14 par	1.49	2	40	4470	feminir	62												
11	1.1-3	n„o	1.58	1	38	3850	feminino													
5	1.1-3	n„o	1.52	2	40	3320	feminir	111	28635.6	156	70.8	117.5	80.5	73	183	56	110	91	0.5783	0.5799
3	1.1-3	1-14 tod	1.55	1		3180	masculin		28826.46	165.85	90	119	73	98	182	40	96	290	0.5774	0.5769
9	3.1-6	n„o	1.59	1	36	3150	feminino													
5	1.1-3	n„o	1.55	1		2900	feminino		14304.75	161.1	53.9	116.5	72	83	174	64	96	59		0.5786
11	3.1-6	n„o	1.67	1	40	3150	masculin		23281.78	179.35	87.9	137.5	76	85	202	70	110	98	0.5841	0.5784
2	1.1-3	1-14 tod	1.61	3	39	3000	feminir	12												
5	1.1-3	n„o		4		3800	mascul	51												
5	1.1-3	n„o		2	37	3180	feminir	25												
0	1.1-3	1-14 par	1.52	4	39	3310	mascul	67												
4	1.1-3	n„o	1.55	1	40	3270	feminino													
11	3.1-6	15 + tod	1.6	4	39	3950	feminir	87	33035.98	153.1	75.4	130.5	86	74	184	60	96	188	0.5708	
0	1.1-3	1-14 tod	1.52	2	40	3020	mascul	33		177.7	65.7	125	77.5	75	172	82	77	49	0.5795	0.5906
16	1.1-3	n„o	1.49	2	37	3730	feminino		42868.1	163.9	83.5	121.5	76	87	194	62	109	139	0.5796	0.5782
4	1 ou -	1-14 par	1.59	2	40	3450	mascul	15	24807.14	174	86.7	118.5	77	93	226	61	144	77	0.5899	0.5973
9	1.1-3	n„o	1.48	1	40	3340	masculin		23981.34	158.05	67.1	117.5	69	71	260	67	170	103	0.5787	0.5769
6	1 ou -	1-14 par	1.58	2	38	2510	feminir	22	14996.15	157.1	51.3	137	86.5	77	149	60	78	45	0.57	0.58
0	3.1-6	n„o	1.48	3		3150	feminir	93												
5	1.1-3	n„o	1.53	3		2550	mascul	13	5990.834	166.1	54.8	119	73	90	272	42	210	99	0.5781	0.5787
8	3.1-6	n„o	1.67	1	39	4100	masculin													
11	1.1-3	n„o	1.54	1	39	3500	masculin		10262.55	179.15	71.5	143.5	89.5	81	178	54	109	76	0.5806	0.5781
5	1.1-3	n„o	1.53	7	40	4000	feminir	63	36312.58	154.7	72.7	108.5	72	77	211	73	114	118	0.5766	0.5751
3	1 ou -	1-14 tod	1.6	5	38	3230	masculin					127	72	80	157	54	82	98		
5	3.1-6	n„o	1.65	3		3290	mascul	150												
8	1.1-3	n„o	1.62	2	39	2860	masculin													
6	1 ou -	15 + tod	1.55	2	39	2270	mascul	31												
18	3.1-6	n„o	1.66	2	43	3250	mascul	120	15248.09	170	70.6	112.5	64	75	205	53	120	167	0.5737	0.583
7	1.1-3	n„o	1.48	1	37	3370	masculin		13569.11	171.2	79.3	129	78	91	179	43	96	241	0.5875	0.5839
4	1 ou -	1-14 tod	1.47	3		1940	feminino													
3	3.1-6	n„o	1.57	9	39	3670	feminir	55	28680.35	156.6	66.2	108	73.5	79	147	52	80	92	0.5729	
6	1.1-3	n„o	1.61	2	37	3090	mascul	64	32188.43	176.8	98.1	126.5	82.5	110	199	50	113	175	0.5811	0.5841
9	6.1-10	n„o	1.62	1	38	3220	masculin													
8	1.1-3	n„o	1.5	1	37	2550	feminino		42685.38	156.2	86.8	114	78.5	84	158	54	88	127	0.5792	0.579
2	1.1-3	n„o	1.51	1	39	2600	masculin													
5	1.1-3	n„o	1.65	1	42	3900	masculin		14064.01	182.1	63.9	124	74	88	290	72	186	159		0.5592
9	1.1-3	n„o	1.59	2		2570	mascul	21	28145.01	176.7	79.4	130.5	87	94	220	64	145	75	0.5699	0.5583
6	1 ou -	15 + tod	1.5	3	40	3400	feminir	11	24083.27	158.5	65.1	112	76	77	188	51	123	62	0.5757	0.5763
5	1.1-3	n„o	1.43	1	41	3650	masculin		18832.62	170.3	71.9	131.5	84	93	182	36	102	255	0.5823	0.5619
11	3.1-6	n„o	1.6	4	41	2980	feminir	20	18914.76	166.9	56.6	106.5	69.5	79	160	54	90	72		0.5713
5	3.1-6	n„o	1.48	3	42	3240	mascul	56												
4	1.1-3	n„o	1.52	1	39	3470	feminino		42002.41	162.45	91.6	109	71	86	175	66	89	123	0.5771	0.5771
4	1.1-3	n„o	1.6	3	38	3270	mascul	41	6533.677	168.15	57.4	146.5	89.5	93	158	90	62	46	0.5777	0.578
18	10	n„o	1.48	3		3950	mascul	22	22429.25	174.55	89.8	130	77	72	212	66	132	70	0.5795	0.5789
3	1 ou -	n„o	1.58	5	40	3350	mascul	31												
12	6.1-10	n„o	1.58	2	39	2640	mascul	45	24635.11	179.1	87.4	138	70	207	313	52	128	633	0.5776	
5	1.1-3	1-14 tod	1.54	3	41	3400	feminir	80	42282.05	164.5	83.7	97.5	60	74	178	70	91	76	0.5799	

pescmae	prenda	pfumomae	paltmae	pgesta	pidgest	ppn	psex	pint	dmgtotdxa	daltura2012	dpeso	dsysmed	ddiamed	dglicose	dcolesterol	dhdl	dldl	dtrig	dECMICE	dECMICD
4	1.1-3	1-14 tod	1.66	1	42	2780	feminino		52559.43	164.45	104.8	115.5	77	89	225	59	134	145	0.5583	
4	1 ou -	n,,o	1.52	1	40	2400	feminino		18257.74	151.8	53.2	96.5	62.5	87	139	46	73	93	0.6228	0.5788
3	1.1-3	n,,o	1.59	2	42	2110	masculin													
0	3.1-6	n,,o	1.59	1	41	3220	masculin													
11	1.1-3	n,,o	1.56	3	40	3720	mascul	48												
3	1 ou -	n,,o	1.55	3		3740	feminir	43												
7	3.1-6	n,,o	1.56	5	38	3850	mascul	17	26539.46	176.6	67.8	145	83.5	86	160	44	111	46	0.5781	0.5742
7	1.1-3	n,,o	1.53	1	42	3450	feminino													
6	1.1-3	n,,o	1.57	1	36	2780	feminino		6250.928	159.7	42.2	101	64.5	78	140	55	70	48	0.5587	0.5781
2	1 ou -	1-14 tod	1.5	1		1480	feminino			147.75		117	70						0.5824	0.5781
12	10	n,,o	1.58	2	38	3150	mascul	130	16892.09	177.65	79.1	111.5	65	96	226	65	142	102	0.5708	0.5797
8	3.1-6	n,,o	1.62	1	41	2950	masculin		13294.62	170.1	69.6	117	73.5	99	187	70	102	84	0.5814	0.5794
8	1.1-3	n,,o	1.52	1	39	3050	feminino			149.6	52.7	118.5	72.5							
8	1.1-3	n,,o	1.48	1	40	3650	feminino		31147.83	159.6	66	122.5	76.5	146	171	60	98	83		
4	1 ou -	1-14 par	1.5	1	40	3410	masculin		13701.25	173.85	73.3	133	66.5	81	172	68	88	67	0.5731	0.5768
9	3.1-6	n,,o	1.56	2	41	3050	mascul	47												
4	1.1-3	n,,o	1.6	1	38	2150	masculin													
3	1.1-3	n,,o	1.51	3	39	2860	mascul	64	21328.38	161.95	76.5	121.5	82	88	214	43	149	101	0.58	0.5833
7	1.1-3	n,,o	1.62	2		3800	mascul	12	38268.53	180.1	112.4	140.5	92.5	335	277	56	139	450	0.5981	0.5938
4	1 ou -	15 + tod	1.65	5		2100	mascul	18	12218.28	171.65	70.9	186.5	103						0.5783	0.5758
4	1.1-3	n,,o	1.6	5	42	3470	mascul	58	7294.658	176.6	67.8	138.5	70	100	170	60	91	106	0.5787	0.5684
11	3.1-6	n,,o	1.63	1	41	3430	feminino													
5	1 ou -	1-14 tod	1.58	5	41	3100	feminir	40	19865.3	165.15	61.6	107.5	62	81	156	72	75	40	0.5683	0.5772
5	1.1-3	n,,o	1.71	3		3500	mascul	40												
7	1.1-3	n,,o	1.64	1		2650	feminino		33791.55	161.5	76.6	113.5	78.5	80	177	53	108	51		0.5819
1	1.1-3	n,,o	1.5	2	42	2800	feminino													
4	1.1-3	n,,o	1.53	2		4000	feminir	16	7566.653	153.25	42.2	95.5	61.5	84	182	58	112	90	0.578	0.5777
2	1 ou -	n,,o	1.54	7		3280	feminir	39											0.5758	
5	1.1-3	1-14 tod	1.6	1	36	2950	feminino		27242.63	163.9	71.4	120	82.5	86	295	75	184	183	0.5664	
4	1.1-3	n,,o	1.47	5	38	2900	feminir	34												
13	1.1-3	1-14 tod		1	39	3400	feminino													
4	1 ou -	15 + tod	1.68	2	40	2650	feminir	18												
8	1 ou -	n,,o	1.58	1	40	3200	feminino		15854.41	168.1	62.9	131	80.5	85	185	75	95	70	0.5773	0.5781
4	1.1-3	n,,o	1.69	10		3780	mascul	39	18048.47	179	80.7	129	83	92	169	46	97	128	0.5777	0.574
11	3.1-6	n,,o	1.54	2	39	3810	feminir	45	22475.34	154.4	59.3	120.5	81	80	226	54	148	168	0.5584	0.5776
10	1.1-3	n,,o	1.52	2		2510	mascul	55												
5	3.1-6	n,,o	1.55	6	40	3180	feminir	27	37820.16	165.6	76.5	97	66.5	79	161	62	82	64	0.5816	0.5776
4	1.1-3	1-14 tod	1.56	1		4050	feminino		20869.07	154.65	60.3	117	74.5	86	174	61	100	59	0.5784	0.5579
5	1.1-3	n,,o	1.54	2		3800	mascul	61												
19	6.1-10	n,,o	1.63	2	41	3710	feminir	70	18161.01	163.9	62.7	123	84.5	81	136	68	58	39	0.578	0.578
3	3.1-6	n,,o	1.59	5	41	3450	mascul	26												
6	1.1-3	15 + tod	1.57	2	38	3000	mascul	16	20095.87	175.95	86.3	165	92.5	84	264	49	182	229	0.5679	0.5762
1	1.1-3	n,,o	1.51	3		3050	mascul	36												
10	3.1-6	n,,o	1.5	1	42	3350	feminino		28835.92	156.9	69.5	104.5	63	71	198	65	113	119		
7	1.1-3	n,,o	1.61	6	41	4000	mascul	44												
3	1 ou -	1-14 tod	1.51	3	35	2000	feminir	22												
0	1 ou -	n,,o	1.51	11		2750	mascul	51	20331.7	169	83.7	125	76	93	335	61	196	500	0.6325	0.6002
5	1.1-3	n,,o	1.6	1	42	3100	feminino		29177.36	153.4	70.1	116	70.5	106	228	68	148	78	0.5885	0.5783

pescmae	prenda	pfumomae	paltmae	pgesta	pidgest	ppn	psex	pint	dmgtotdxa	daltura2012	dpeso	dsysmed	ddiamed	dglicose	dcolesterol	dhdl	dldl	dtrig	dECMICE	dECMICD
3	3.1-6	1-14 tod	1.64	3	40	2800	feminir	60	24523.82	168.05	63.4	110.5	73	76	125	47	67	60	0.5758	0.5786
10	6.1-10	n„o	1.51	4	39	3400	feminir	14	42984.11	155.85	82.8	106	66	84	202	45	140	107	0.5974	0.5782
7	1.1-3	15 + tod	1.56	4	39	2300	feminir	51	12186.77	152.05	48	122	78.5	80	202	78	110	73	0.5782	0.5749
4	1.1-3	1-14 tod	1.68	2	39	2920	mascul	24		192.25	127	143.5	74	75	140	32	70	225	0.5884	0.5792
4	1 ou -	n„o	1.53	3	39	2100	mascul	11												
5	1.1-3	1-14 tod	1.49	5	33	2010	feminir	25												
4	1 ou -	n„o	1.53	4	41	3350	feminir	33												
3	1 ou -	1-14 tod	1.48	2		2350	feminino		18016.01	150.2	53.7	115	77.5	83	198	68	118	102	0.58	0.5587
8	1.1-3	15 + tod	1.67	4	39	2150	feminir	10												
5	1 ou -	15 + tod	1.55	2	43	2570	mascul	13												
3	1.1-3	1-14 tod	1.62	3	39	4000	mascul	48	37762.75	188.5	118.9	137	86.5	97	204	42	136	117	0.6681	0.6295
10	1.1-3	n„o	1.58	2	43	3150	feminir	36	43496.05	166.8	93.3	147	100.5	76	140	59	68	51	0.588	0.5881
4	1.1-3	n„o	1.5	1		3040	feminino		36068.69	154.35	76.4	106	68.5	92	184	47	121	109		0.5843
9	1.1-3	15 + tod	1.52	1	40	2960	feminino		21657.31	162.3	61.5	111	68.5	70	190	79	90	96	0.578	0.5481
5	1.1-3	n„o	1.56	1	41	2730	feminino		27308.38	157.25	68	124.5	86.5	115	168	54	92	128		
3	1 ou -	15 + tod	1.58	3		1450	mascul	39												
5	1.1-3	n„o	1.57	1	42	2400	feminino													
7	1 ou -	n„o	1.56	3	36	3230	mascul	22												
16	6.1-10	n„o	1.57	1	38	3270	masculin													
7	1.1-3	n„o	1.54	1		1800	masculin		23546.05	172.65	80.3	113	72	85	157	61	88	48	0.5795	0.5804
5	1.1-3	1-14 par	1.39	3	38	2280	mascul	35												
8	1.1-3	1-14 par	1.62	4	38	3550	mascul	15												
5	1.1-3	15 + tod	1.51	1	39	3020	masculin													
10	6.1-10	n„o	1.58	3	38	4000	feminir	61	30532.73	166.45	72.8	118.5	70.5	90	218	80	117	96	0.5794	0.5775
6	1.1-3	n„o	1.51	7	38	3700	mascul	34												
4	1 ou -	n„o	1.41	5	40	2900	mascul	22												
7	3.1-6	n„o	1.64	1	39	4120	masculin		40734.14	180.95	116.5	131.5	83.5	104	235	53	134	255	0.5843	0.5714
0	1.1-3	n„o	1.63	4	38	3300	mascul	40				119.5	76							
5	1 ou -	n„o	1.6	1		3530	feminino		23729.34	171.15	63.2	105.5	68.5	83	198	67	96	205		0.567
5	3.1-6	n„o	1.58	5	39	3750	masculin		17218.58	180.7	72.7	135	87	74	190	62	109	76	0.5783	0.577
6	1 ou -	n„o	1.62	2	40	3400	feminir	24	36279.05	161.3	74.8	115.5	76	86	205	69	113	141	0.578	0.5766
2	1.1-3	n„o	1.6	3	39	3900	feminir	60												
7	1 ou -	n„o	1.58	4	41	2650	feminir	64	24839.91	155.9	62	104.5	76	81	177	75	91	45	0.5708	0.5748
12	10	n„o	1.65	1	40	3900	masculin													
5	1.1-3	1-14 tod	1.7	1	39	2790	masculin													
12	1.1-3	n„o	1.58	1	38	2950	masculin		27705.25	175.7	85.6	131.5	76.5	96	221	65	142	79	0.5749	0.5762
5	1 ou -	n„o	1.58	1	39	3060	feminino		17374.03	157	50.9	124.5	76.5	83	182	78	88	61	0.5618	0.5783
5	1.1-3	1-14 tod	1.63	2	42	4370	mascul	46	7907.723	178.15	70.5	130	81	96	175	63	97	94	0.5768	0.6149
0		n„o	1.39	3		580	mascul	42												
4	3.1-6	n„o	1.62	6	39	4100	mascul	120	15205.31	178	76.7	118.5	66.5	97	182	74	98	36	0.6363	0.5786
8	1.1-3	15 + tod	1.59	3		3250	mascul	21	23002.06	164.95	82	133.5	81	83	200	67	121	77	0.5782	0.5776
7	3.1-6	1-14 par	1.48	2	42	3500	mascul	25	3315.897	173.8	61.7	124	68.5	90	212	56	131	127	0.5623	0.5784
6	1 ou -	1-14 tod	1.54	1	40	3340	masculin													
8	1 ou -	15 + tod	1.61	1	40	2950	masculin		27396.3	169.8	84.6	116	72.5	102	218	43	99	434	0.6094	0.5789
5	1 ou -	1-14 tod	1.52	3	41	3570	mascul	33												
4	1.1-3	n„o	1.61	3		2200	feminir	12	28815.55	164.2	71.2	144.5	89	91	216	57	141	97		0.583
6	1.1-3	n„o	1.6	2	40	3800	mascul	29	16153.6	177.5	80.5	125	72	69	165	63	80	100	0.6	0.5842
7	10	15 + tod	1.52	5	42	3800	mascul	22	21198.37	174.4	82.7	124	79	108	181	41	127	99	0.5778	0.5699

pescmae	prenda	pfumomae	paltmae	pgesta	pidgest	ppn	psex	pint	dmgtotdxa	daltura2012	dpeso	dsysmed	ddiamed	dglicose	dcolesterol	dhdl	dldl	dtrig	dECMICE	dECMICD
8	3.1-6	1-14 par	1.53	2	37	3510	feminir	60	30572.23	164.5	72.6	105.5	65.5	79	144	50	75	109	0.5705	0.572
9	1.1-3	n„o	1.63	1	41	2600	masculin		34855.39	167.6	90.5	134.5	89	123	244	45	138	389	0.5531	0.5703
9	3.1-6	1-14 tod	1.55	1	43	3500	masculin		24257.99	185.3	95	132.5	78.5	126	172	43	88	242	0.5792	0.6403
9	3.1-6	n„o	1.71	4		2810	feminir	92	38182.95	173.1	84	132	87	90	235	65	152	87	0.5952	0.5971
10	1.1-3	1-14 par	1.53	1	38	3030	masculin		29128.15	167.8	86.1	134.5	84	95	163	61	88	90		0.5783
3	1.1-3	n„o	1.6	1		2720	feminino													
1	1.1-3	n„o	1.52	8	37	4100	feminir	25												
1	1.1-3	n„o	1.52	5	39	3730	feminir	139	19306.14	159.7	53.8	113	71.5	81	206	85	102	121	0.5628	0.579
8	3.1-6	n„o	1.58	1	38	2700	masculin													
8		1-14 tod	1.68	1		3750	feminino		12094.96	157.2	46.3	103.5	69	98	161	67	78	55	0.5618	0.5736
3	1.1-3	n„o	1.49	1	40	3580	feminino		29946.91	159.35	70.6	118.5	70.5	95	149	63	64	121	0.5776	0.5813
14	3.1-6	n„o	1.57	1	43	3660	masculin		27192.08	172	86.6	122	76	159	240	45	115	333	0.6111	0.5822
4	1.1-3	n„o	1.62	1	42	3770	masculin		29896.14	184.05	101.1	145.5	92	82	183	45	121	94	0.5825	0.5789
6	1 ou -	1-14 tod	1.54	1	41	3410	masculin													
3	1.1-3	15 + tod	1.59	2		3310	feminir	14	32164.42	164.2	79.6	113	71	79	125	40	67	60	0.5808	0.5835
5	1.1-3	n„o	1.61	1	39	2850	masculin		27454.77	176.7	89.9	125	79	98	212	40	119	337	0.597	0.5772
5	1 ou -	n„o	1.43	2	40	3500	feminir	13	18223.52	164	61.3	114.5	69.5	67	127	68	47	78	0.578	0.5805
15	10	n„o	1.61	2	36	2810	mascul	39												
12	1.1-3	1-14 par	1.58	4		3350	mascul	46												
6	3.1-6	1-14 par	1.59	4	37	3400	mascul	63	18429.26	168.35	75.2	118.5	77.5	95	196	59	114	155	0.5789	0.583
5	1.1-3	n„o	1.66	1	42	3550	feminino		36424.37	162.75	84.7	135	65	96	208	78	99	103	0.6001	0.6498
15	10	n„o	1.54	2	41	3100	mascul	49	26047.63	179.3	79.8	127.5	82	81	233	56	142	210	0.602	0.5859
4	1.1-3	n„o	1.6	2	39	3050	mascul	44	32840.69	178.65	98.1	133.5	89	86	163	30	102	169	0.5878	0.5908
1	1 ou -	1-14 tod	1.57	1	42	3430	masculin		14906.49	178.6	84.8	132.5	79	99	189	54	115	100	0.578	0.5802
2	1.1-3	n„o	1.62	3	40	3650	feminir	105	46749.85			119	72	77	154	40	96	104		0.5783
5	1.1-3	n„o	1.51	5	40	3100	mascul	72	11881.17	173.05	59.3	133.5	74	91	245	95	138	45		
16	1.1-3	n„o	1.57	1	42	2550	feminino		17264.35	156.45	53.1	113	77	95	142	65	64	87	0.58	0.58
1	1.1-3	n„o	1.42	4	41	3650	feminir	14												
8	1.1-3	1-14 tod	1.51	10	34	2100	mascul	18												
13	1.1-3	n„o	1.73	1	43	4300	masculin			177.7	148	143	85.5	456	159	33	77	333		
7	3.1-6	n„o	1.63	1	37	3150	masculin		25523.8	178.3	87	140	87.5	61	164	47	91	142	0.5812	0.5803
3	1.1-3	n„o	1.5	2		2950	feminir	73	37171.09	155.25	78.3	105.5	58.5	106	166	61	95	72	0.5761	0.5772
10	3.1-6	1-14 tod	1.56	1	41	2800	masculin		12013.96	172	66.5	149	79	93	220	65	135	93	0.5783	0.5808
5	1.1-3	n„o		3	39	3150	feminir	139	33902.52	171.45	82.2	112.5	69	88	194	74	109	66		
9	3.1-6	15 + tod	1.54	1	42	2950	feminino													
3	1 ou -	n„o	1.6	3		3250	feminir	18	28785.39	161.4	65	100.5	66.5	88	215	74	128	50	0.5767	
5	1.1-3	15 + tod	1.52	4	41	3520	feminir	143	14128.54	170.05	57.3	114	71	75	150	55	86	57		
6	1.1-3	15 + tod	1.57	3	40	2500	mascul	29												
4	3.1-6	n„o	1.54	4	39	3550	feminir	57												
5	1.1-3	15 + tod	1.56	2		3050	mascul	57												
5	10	n„o	1.56	4	43	3200	mascul	12												
2	1.1-3	n„o	1.5	10	40	4520	feminir	12												
9	1 ou -	1-14 tod	1.49	1	39	2730	masculin													
1	1.1-3	1-14 tod	1.56	4	38	3200	feminir	22												
10	3.1-6	n„o	1.53	1	40	3560	masculin		22265.62	169.6	77.6	99.5	70.5	66	124	45	66	48	0.5794	0.5616
11	1.1-3	n„o	1.45	4	41	3090	feminir	17												
6	3.1-6	n„o	1.74	3	40	3600	feminir	48	40498.18	168.2	89	112	69.5	77	175	52	102	108	0.5788	0.5841
6	3.1-6	n„o	1.48	1	37	2830	masculin													

pescmae	prenda	pfumomae	paltmae	pgesta	pidgest	ppn	psex	pint	dmgtotdxa	daltura2012	dpeso	dsysmed	ddiamed	dglicose	dcolesterol	dhdl	dldl	dtrig	dECMICE	dECMICD
5	6.1-10	n„o	1.55	3	39	3600	mascul	63	19660.32	174.5	77.5	136.5	82	81	197	65	113	112	0.5816	0.5778
12	3.1-6	n„o	1.55	2	41	3500	feminir	14	41183.76	158.8	79.9	115.5	75.5	85	183	63	93	151	0.584	0.6056
9	6.1-10	1-14 tod	1.6	1	39	3100	masculin		9384.529	173.7	64.8	117	70.5	76	158	63	88	47	0.5577	0.5547
9	1.1-3	n„o	1.6	4	37	3480	mascul	55	5955.987	179	81.4	125	73	63	145	58	80	48	0.5779	0.6126
12	6.1-10	n„o	1.63	2		3500	mascul	51	21091.96	189.55	88.2	115	65	69	152	40	104	66	0.5837	0.5782
5	1.1-3	15 + tod	1.61	2	39	3150	mascul	47												
17	3.1-6	1-14 tod	1.56	1	38	3220	masculin													
7	6.1-10	n„o	1.56	1	41	3850	feminino		21351.22	165.05	60.9	105.5	67	79	161	62	86	66	0.5781	0.5763
5	1.1-3	n„o	1.6	3	40	3320	mascul	130	20815.75	186.15	92.5	135	57	101	162	62	81	60	0.5752	0.5959
7	1.1-3	n„o	1.52	3		3240	mascul	22	30313.59	163.65	94.5	139	73.5	103	209	55	121	189		
8	1.1-3	1-14 par	1.65	2	40	2880	feminir	23	31804.55	164.35	79.4	120	65.5	81	148	39	79	158	0.5816	0.579
8	1.1-3	n„o	1.57	1	39	3200	feminino		17973.77	151.25	50.9	95.5	67.5	81	219	40	134	280	0.5772	0.5774
0	1 ou -	n„o	1.49	5		2450	mascul	52												
5	1.1-3	n„o	1.51	1	42	3950	masculin		20014.03	181.7	87.6	133.5	77	85	158	44	86	150	0.5779	
1	1 ou -	1-14 par	1.49	1		2300	masculin		13834.83	158	64.5	141.5	82.5	83	179	57	112	74		0.5695
6	1.1-3	n„o	1.52	2	42	3750	feminir	45	26842.5	158.65	61	110	72.5	58	141	62	72	66	0.5745	0.5686
5	1 ou -	15 + tod	1.54	2		2220	mascul	14	23317.37	177.2	91.5	145.5	85	95	208	41	112	308		
4	1.1-3	15 + tod	1.57	7	38	3740	mascul	29												
5	1.1-3	n„o	1.55	1	43	2850	feminino		29014.58	164.7	65.2	129	88	86	240	55	170	99		
0	1 ou -	1-14 tod	1.47	2		2800	feminir	27						84	194	70	112	50		
12	6.1-10	n„o	1.56	4	39	2770	feminir	23	20767.35	161.7	55.1	102	72	76	180	90	72	64	0.5668	0.5746
0	1 ou -	1-14 tod	1.63	4		2040	mascul	23												
7	1 ou -	1-14 tod	1.53	1		3050	masculin		4861.15	168.1	68.9	115	66.5	74	152	51	88	101	0.5768	0.5866
7	1 ou -	n„o	1.46	5	42	3100	mascul	27	10728.71	162.35	63.7	128.5	81.5	314	142	52	82	47		
1	1 ou -	n„o	1.46	2	39	3250	feminir	26	24035.93	159.25	65	112.5	64.5	73	168	49	105	85	0.5798	0.5818
5	3.1-6	n„o	1.51	1	41	3610	masculin													
6	1.1-3	15 + tod	1.61	1		1620	feminino													
8	1 ou -	1-14 tod	1.55	1		2430	masculin		19387.38	178.1	77.6	123	71	81	206	48	129	141	0.5776	0.5861
5	1.1-3	n„o	1.52	1	39	3000	feminino													
4	1.1-3	1-14 par	1.61	1	36	3200	masculin													
4	1.1-3	n„o	1.64	1	39	3350	feminino		17386.34	162	54.4	119.5	66.5	90	217	63	139	58	0.5757	0.5777
6	1 ou -	n„o	1.51	1	41	3600	masculin		21753.01	178	81.8	128.5	79	74	138	47	77	67		
15	10	n„o	1.58	2	41	3400	feminir	20												
0	1 ou -	15 + tod	1.56	4		2900	mascul	153	3596.095	178.5	62.9	148.5	63	79	197	45	135	60	0.5769	0.5788
4	1.1-3	n„o	1.56	5	39	3350	mascul	34												
4	1.1-3	n„o	1.67	1		4350	masculin													
0	1.1-3	n„o	1.49	1	38	2950	masculin													
5	3.1-6	1-14 tod	1.54	4	39	3500	feminir	75												
0	1 ou -	n„o	1.52	3	40	3400	mascul	105	13261.09	178.5	78.4	127	77	95	195	53	123	83	0.5784	0.5779
3	1.1-3	n„o	1.48	1	38	3270	masculin													
12	1.1-3	1-14 tod	1.69	2	41	2300	mascul	22												
13	6.1-10	n„o	1.52	3	39	3550	mascul	63												
4	1.1-3	1-14 tod	1.61	1	39	2750	feminino													
8	1 ou -	n„o	1.44	1	41	2400	feminino													
3	1.1-3	n„o	1.59	3		2960	mascul	23	27404.47	167.8	87.2	124.5	79	92	152	58	72	130	0.5804	0.572
4	1.1-3	1-14 par	1.61	2	40	3320	mascul	56	8036.66	171.45	62.8	119	82	92	205	78	114	67	0.5783	0.5649
5	1 ou -	n„o	1.53	2	37	3470	feminir	22	40798.44	165.7	83	123.5	71.5	98	200	60	107	143	0.583	0.5837
9	6.1-10	n„o	1.55	1	40	2800	feminino		23603.3	162.2	59.1	112	68	88	163	56	87	80	0.5789	0.5788

pescmae	prenda	pfumomae	paltmae	pgesta	pidgest	ppn	psex	pint	dmgtotdxa	daltura2012	dpeso	dsysmed	ddiamed	dglicose	dcolesterol	dhdl	dldl	dtrig	dECMICE	dECMICD
5	3.1-6	n„o	1.6	5	39	3320	mascul	43	38142.76	171	103.2	118	82.5	84	172	39	100	192	0.5767	0.5783
9	3.1-6	n„o	1.64	1	40	3480	feminino		20907.12	169.65	59.2	101.5	68.5	93	157	63	83	82	0.5805	0.5768
4	1.1-3	n„o	1.57	7	36	2660	feminir	45	23458.15	162.85	66.5	102.5	72	89	207	50	138	83	0.5893	0.5698
5	1.1-3	n„o	1.65	2	41	3720	mascul	168												
0	1 ou -	n„o	1.57	2		2040	mascul	12												
15	1.1-3	1-14 tod	1.63	1	37	3200	masculin		17546.6	176.75	72.9	128.5	72	82	168	76	78	55	0.5719	
6	1.1-3	1-14 par	1.58	1	39	3000	feminino		44139.58	159.75	88.6	107.5	73	92	209	46	139	135		
8	3.1-6	n„o	1.63	1	39	2580	masculin		15269.26	177.5	72.1	147	93.5	89	208	48	121	215		0.5629
0	1 ou -	n„o	1.67	2		3630	mascul	20	18246.42	170.4	79.6	118.5	60.5	89	210	78	116	64	0.582	0.5782
3	1.1-3	1-14 par	1.52	13	39	2630	feminino		22531.66	155.05	60.2	113	74	86	167	68	63	218	0.5732	0.5733
5	3.1-6	n„o	1.64	2	40	3700	mascul	129	28704.44	183.4	87.5	122.5	76	90	151	39	91	76	0.5802	0.5831
5	1.1-3	n„o	1.53	3		2450	mascul	106	8508.915	171.2	56.2	106	60	81	188	56	104	156	0.5775	0.5781
5	1 ou -	15 + tod	1.51	1	40	2870	feminino		17874.85	153.55	58.5	129	80.5	91	174	75	81	66	0.5775	0.5777
0	1.1-3	1-14 tod	1.52	7		3600	feminino		32254.13	157.85	73.8	107	64.5	90	197	59	116	93	0.5844	0.5799
16	10	15 + tod	1.58	3	41	2770	feminir	32	30530.24	154.7	65.8	111.5	73	111	161	52	96	50	0.5931	0.6016
12	3.1-6	n„o	1.65	1		2650	masculin		34382.71	182.6	94.6	127	81.5	87	190	50	92	238	0.5555	0.5793
9	3.1-6	n„o	1.61	2	41	3450	feminir	21												
9	3.1-6	1-14 tod	1.65	2	39	3530	feminir	37	5925.545	171.1	64.4	120.5	65.5	77	145	69	64	58		
9	3.1-6	1-14 par	1.62	2	38	3250	feminir	27	26953.54	158.45	68.9	111	72.5	90	214	74	115	127	0.5651	0.5774
13	3.1-6	n„o	1.62	2	36	2300	feminir	13	48281.35	169.4	96.3	118	77	91	190	46	114	128	0.5911	0.5881
5	1.1-3	n„o	1.64	1	38	2900	feminino		45739.98	159.8	93	131.5	86.5	76	230	73	129	184	0.5907	0.5808
5	1.1-3	1-14 tod	1.62	2		2550	mascul	17	21741.96	176.9	84.7	121	70.5	96	198	70	110	93	0.5782	
9	1.1-3	1-14 tod	1.54	3	41	3900	mascul	35	22231.29	172.2	94	154.5	93.5	65	217	42	147	168	0.6884	0.6763
12	1.1-3	n„o	1.59	1		3400	masculin		35266.05	183.6	101.5	141	88	111	225	50	154	116		0.5788
10	1.1-3	n„o	1.53	1	40	2940	feminino		42991.65	169.5	93	134.5	88.5	76	197	75	103	93	0.5811	0.5788
3	1.1-3	n„o	1.57	2	39	3050	feminir	36	16719.36	165	59	127	82	108	160	66	79	57	0.5786	0.5729
9	6.1-10	n„o	1.64	1	38	4220	masculin		17345.24	185.5	90.3	143	77	80	187	62	108	63	0.6299	
7	3.1-6	1-14 par	1.56	3		3150	feminir	16												
0	1 ou -	n„o	1.57	9		3600	feminir	33	26288.5	172.6	74.4	103	64	72	183	66	107	61	0.57	0.5703
7	1.1-3	n„o	1.6	1	40	3680	masculin													
7	3.1-6	n„o	1.62	2	41	3850	mascul	31	17182.96	178.3	69.5	116.5	76	79	172	44	107	96	0.5904	0.5783
7	1.1-3	n„o	1.64	1	42	3620	feminino		39715.63	164.95	83.5	110	69.5	91	181	65	101	84		
5	1 ou -	1-14 par	1.48	1	37	1900	feminino		17502.01	147.1	54.6	118.5	74.5	55	170	51	113	37	0.5796	0.5768
2	1.1-3	n„o		4	40	2590	mascul	29												
0	1 ou -	n„o	1.51	10		2880	mascul	76												
7	1.1-3	n„o	1.62	1		3460	feminino		40681.16	156.05	80.3	120.5	83.5	80	277	54	206	100	0.5973	0.5829
0	1.1-3	n„o	1.64	1		3120	feminino		19631.46	160	56.8	118	79	79	240	82	134	133	0.5789	0.574
9	1.1-3	n„o	1.62	4	39	3800	feminir	26	39126.66	172.05	100.3	114.5	80.5	94	170	52	77	155	0.5815	
5	3.1-6	n„o	1.58	4		3550	feminir	58		167.1		103	65						0.5771	0.5734
3	1 ou -	1-14 tod	1.5	5		2650	feminir	36												
6	1.1-3	1-14 par	1.5	1		3330	masculin		18815.54	176.2	84.6	142	90.5	92	165	44	92	157	0.5789	0.578
3	1 ou -	1-14 tod	1.58	3	39	3510	mascul	29	24224.57	185.25	91.2	126	75	101	148	48	70	122		
1	1.1-3	n„o	1.51	4	39	3000	feminir	44	34307	161.15	70.8	110.5	73	83	209	80	117	56	0.5801	0.579
4	1.1-3	15 + par	1.55	2	40	3150	mascul	12	35067.75	169.5	96.9	145.5	81.5	96	219	55	153	63	0.583	0.5707
15	10	n„o	1.6	1	41	2710	feminino		39068.77	165.5	80.8	108	72	72	188	75	95	93	0.5762	0.5788
7	3.1-6	1-14 tod	1.49	1	40	2220	feminino													
8	3.1-6	15 + tod	1.54	3	39	2350	feminir	20	31598.71	161.85	79.6	113	79	89	138	48	74	72	0.5856	0.6315
16	10	1-14 par	1.57	2	41	2850	feminir	23	18654.42	164.7	56.4	117.5	66	91	176	60	99	68		0.5776

pescmae	prenda	pfumomae	paltmae	pgesta	pidgest	ppn	psex	pint	dmgtotdxa	daltura2012	dpeso	dsysmed	ddiamed	dglicose	dcolesterol	dhdl	dldl	dtrig	dECMICE	dECMICD
3	6.1-10	15 + tod	1.57	3	39	3650	mascul	25	33585.56	183.7	107.8	137.5	83	78	193	48	126	162	0.5877	0.5921
11	3.1-6	n„o	1.76	2	39	3150	feminir	23	37124.26	179.3	78.7	107.5	69.5	84	219	88	111	106	0.5619	
5	1.1-3	n„o	1.52	2		3750	mascul	40												
17	1.1-3	1-14 tod	1.6	2	40	3290	mascul	18												
4	1.1-3	1-14 par	1.58	3	38	3000	feminir	72												
8	3.1-6	n„o	1.58	2	40	3420	mascul	13	27047	177.7	90.5	117.5	70	84	166	38	103	135	0.5781	0.5758
5	1.1-3	n„o	1.55	2	39	4000	feminir	62	32308.33	168	76	108	72.5	81	176	75	83	78	0.5792	0.5764
5	1.1-3	n„o	1.58	2	39	3430	mascul	10												
9	1.1-3	n„o	1.56	2	38	3500	feminir	25												
9	1.1-3	n„o	1.55	3	38	3200	feminir	42												
7	6.1-10	1-14 tod	1.72	4	38	4270	feminir	22												
5	1 ou -	n„o	1.66	2	40	3800	mascul	26	24547.78	175.5	85.2	131	68	84	248	47	173	150	0.6319	0.6459
20	3.1-6	15 + tod	1.68	5	39	3530	mascul	14		173.7	142.9	136	77.5	113	172	48	111	90		
1	1.1-3	15 + tod	1.52	4	40	3400	mascul	37												
9	6.1-10	n„o	1.63	3	39	3140	mascul	17												
0	1 ou -	n„o	1.42	5	35	2980	feminir	44												
7	1.1-3	n„o	1.68	1	40	3000	feminino		17699.42	164.55	59.9	113	74	72	252	92	145	87	0.5613	0.578
8	3.1-6	n„o	1.52	2	39	3460	feminir	66												
1	1.1-3	n„o	1.57	2	40	3550	feminir	45												
4	1.1-3	n„o	1.67	1	42	3740	masculin													
2	1.1-3	n„o	1.57	4	38	4000	mascul	94	30977.48	171.8	94.4	131	80.5	94	260	54	165	214	0.6052	0.5888
10	6.1-10	1-14 tod	1.52	7	35	2780	mascul	59	29373.04	179	94.1	143.5	75.5	83	158	57	80	70	0.5758	0.5754
0	1 ou -	1-14 tod	1.57	3		2880	feminir	12	54330.33	157.5	94.2	107.5	73.5	58	217	78	126	75		0.576
17	3.1-6	n„o	1.63	2	40	3450	mascul	41	10924.82	183.95	85.8	125.5	67.5	86	159	54	86	77	0.5785	0.5802
3	1.1-3	n„o	1.52	4	41	2400	feminir	26	28657.22	158	68.9	123.5	75	96	175	49	102	124	0.5529	
4	1.1-3	n„o	1.55	1	39	2950	masculin		32337.48	182.6	89.2	131.5	78.5	105	185	41	93	209	0.5784	0.5766
5	1 ou -	n„o	1.52	2	36	3100	mascul	13	3543.923	159.15	50	128	74	70	161	68	79	58	0.5795	0.5777
6	1.1-3	1-14 par	1.54	3	38	3200	feminir	34	54271.63	156.8	104.8	127	87.5	73	150	47	89	94	0.6077	0.5781
9	1.1-3	n„o	1.6	1	40	3400	masculin													
5	1.1-3	n„o	1.61	1	42	3800	feminino													
4	1.1-3	15 + tod	1.55	3		3120	mascul	19	6619.25	174	59.6	117	63.5	95	183	62	109	48	0.5772	0.5777
2	1.1-3	n„o	1.55	4		2800	feminir	74												
5	1 ou -	1-14 tod	1.55	3		2800	mascul	13												
3	1.1-3	1-14 tod	1.51	3	42	2820	masculin		7822.125	174	67.2	126	82.5	97	194	59	119	65	0.5868	0.5786
17	10	1-14 tod	1.76	1	38	3240	feminino		18715.52	173.9	62.7	105.5	68	101	181	76	90	96	0.5778	0.6475
3	1.1-3	n„o	1.52	10	40	3030	mascul	83												
6	3.1-6	n„o	1.51	1	41	2900	masculin		14754.69	168.9	75.4	131.5	79.5	90	184	47	125	50	0.5781	0.5829
4	1 ou -	n„o	1.68	2		3180	mascul	14												
8	1.1-3	n„o	1.49	2	40	3650	mascul	21	10724.01	170.4	62.5	133.5	84.5	85	170	54	102	60	0.5789	
4	1 ou -	n„o	1.54	1	39	3750	masculin													
2	3.1-6	1-14 tod	1.63	1		1800	masculin		31218.52	183.45	100.1	148	86.5	85	189	47	110	170	0.6053	0.5825
15	10	n„o	1.6	1		3760	feminino		17433.04	168.55	59.6	113.5	73.5	95	189	78	85	107	0.5767	0.5683
11	1.1-3	n„o	1.57	1	40	3200	feminino													
17	3.1-6	15 + tod	1.66	2	38	3160	feminir	34												
5	1.1-3	n„o	1.49	2	38	2800	feminir	40		155.5		117.5	68						0.5775	0.5741
8	1.1-3	1-14 par	1.56	3		3600	mascul	66	13067.2	185.45	75.7	131.5	66.5	85	165	44	98	128	0.5771	0.5784
5	1 ou -	n„o	1.49	6	39	3650	feminir	72	25586.31	157.95	65	110	66.5	87	220	78	123	56	0.5774	0.5778
13	10	n„o	1.48	2		3700	mascul	51	16436.25	177.05	79.9	111.5	62	79	171	63	90	149	0.5777	0.6247

pescmae	prenda	pfumomae	paltmae	pgesta	pidgest	ppn	psex	pint	dmgtotdxa	daltura2012	dpeso	dsysmed	ddiamed	dglicose	dcolesterol	dhdl	dldl	dtrig	dECMICE	dECMICD
3	1.1-3	15 + tod	1.6	1	41	3550	feminino													
2	1 ou -	1-14 tod	1.44	1		2340	feminino		25147.71	155.3	69	113.5	69.5	75	169	50	111	54	0.5781	0.5894
5	1 ou -	1-14 tod	1.47	2	40	2870	feminir	38												
6	3.1-6	n„o	1.6	3	40	3460	feminir	59												
2	1 ou -	n„o	1.46	3		1610	feminir	14	21685.46	160.95	63.9	105.5	63	82	133	43	82	87	0.5711	0.5788
4	1 ou -	n„o	1.55	1	41	3800	masculin													
2	3.1-6	n„o	1.55	5		3250	feminir	15												
8	3.1-6	n„o	1.54	1	41	3600	feminino							87	195	74	109	43	0.5775	0.577
16	10	n„o		3	40	2920	mascul	19												
7	1.1-3	n„o	1.53	1		3300	masculin													
5	1 ou -	1-14 tod	1.47	3	40	3500	mascul	33												
0	1 ou -	1-14 tod	1.46	7	36	2330	mascul	14												
5	3.1-6	n„o	1.56	2	41	4060	mascul	45												
4	3.1-6	n„o	1.57	7	40	3870	feminir	50	13547.94	159.2	54.8	105.5	66	63	78	34	33	52	0.5877	0.5782
8	3.1-6	n„o	1.54	2	37	3750	mascul	63	25893.21	181.1	99.9	159.5	91	116	168	33	80	250		0.5804
9	6.1-10	n„o	1.53	4	39	3640	mascul	142	19803.84	175.9	76.8	108.5	59.5	77	168	38	117	78	0.5783	0.5777
6	1 ou -	n„o	1.53	3	40	2250	mascul	27	5950.365	175.5	65.7	113.5	72.5	98	162	48	83	150		0.5632
17	3.1-6	n„o	1.66	4	40	3740	feminir	90	26988.86	168.3	70.1	114	78.5	86	200	57	125	140		
7	1 ou -	n„o	1.46	2	38	2850	mascul	59												
16	6.1-10	n„o	1.54	2	39	3150	masculin		36786.58	184.1	117.9	125	70	107	230	40	121	326	0.578	0.5917
2	1.1-3	1-14 tod	1.53	6	38	3100	feminir	27	30850.66	160.05	82.8	132.5	89.5	80	215	38	140	211	0.626	0.6125
16	10	n„o	1.6	1	39	2920	masculin		16661.7	174	79.9	113	67.5	64	157	68	78	46	0.5828	0.614
4	1.1-3	n„o	1.46	5	39	3630	feminir	39	34508.04	160.85	79.3	111.5	68.5	95	198	55	130	81	0.5785	0.5848
5	1.1-3	n„o	1.53	2	36	2900	mascul	44	20576.28	178.15	81.9	134.5	75.5	62	205	52	133	95	0.6025	0.5789
9	1 ou -	n„o	1.57	1	40	2660	feminino													
12	10	n„o	1.66	3	38	3360	feminir	18	17901.7	169.3	62.8	111.5	65	77	212	74	109	154	0.5784	0.578
1	1 ou -	15 + tod	1.57	9	41	3260	feminir	22												
2	1 ou -	1-14 tod	1.62	1	36	3600	masculin													
4	1.1-3	n„o	1.55	1	42	2450	feminino													
8	3.1-6	15 + tod	1.61	7	41	3330	feminir	55	34463.5	164.35	84.1	121	78	132	292	75	205	89	0.8959	0.5816
7	1.1-3	1-14 tod	1.59	5	42	2650	mascul	20												
13	10	n„o	1.58	1	39	3610	feminino		23295.05	165.1	62.1	106.5	73	84	145	73	56	72	0.5613	0.5801
6	1 ou -	n„o	1.58	1	37	2970	feminino			161.35										0.5633
5	1 ou -	n„o	1.55	4		3400	feminir	41	42839.05	164.75	85	135	82	90	243	88	138	70		
3	1.1-3	n„o	1.5	2	37	2980	feminir	22	13374.68	163.6	62	118.5	67.5	112	192	72	90	163	0.578	0.5781
5	3.1-6	n„o	1.58	5	40	3280	feminir	41	31350.48	172	73.7	112	70.5	98	157	60	80	141	0.5529	0.5802
4	1 ou -	1-14 tod	1.52	1		2900	masculin		32177.88	167	89.5	112	61.5	71	139	53	66	97	0.579	0.578
10	1.1-3	n„o	1.56	3	42	2590	feminir	23												
17	1.1-3	n„o	1.57	1	40	3750	feminino													
12	3.1-6	n„o	1.6	1	40	3620	masculin		12360.89	176.6	65.9	135	73	91	172	61	103	52	0.577	0.578
5	1.1-3	n„o		6	38	2940	feminir	99	45175.43	163	87	111	78.5	84	239	51	150	220	0.5475	0.5635
5		n„o	1.72	3		3300	feminir	70	17782.61	171.5	63	107.5	65.5	81	185	77	92	80	0.5344	0.5151
3		n„o	1.6	3	38	2850	feminir	48	31360.41	154	66.7	105.5	68	86	263	85	159	71	0.5775	0.5813
7	1.1-3	n„o	1.61	1	38	3300	feminino		59056.29	161.05	108.9	114.5	79	95	242	53	142	270	0.5803	0.5894
1	1 ou -	15 + tod	1.49	2		2690	feminir	62	29829.69	138.5	64.6	113	70	99	202	36	137	201	0.5822	0.5833
11	1.1-3	n„o	1.6	2		2400	feminir	16												
16	6.1-10	n„o	1.63	1	40	3510	masculin													
6	1 ou -	1-14 tod	1.57	1	38	3000	masculin													

pescmae	prenda	pfumomae	paltmae	pgesta	pidgest	ppn	psex	pint	dmgtotdxa	daltura2012	dpeso	dsysmed	ddiamed	dglicose	dcolesterol	dhdl	dldl	dtrig	dECMICE	dECMICD
6	1.1-3	n„o		3		1620	mascul	19												
4	1 ou -	15 + par	1.6	2	38	2950	mascul	57	21291.28	175.2	86.4	138	82	78	167	53	102	67	0.5833	0.6079
7	1.1-3	n„o	1.66	1	43	3060	feminino													
13	1.1-3	n„o	1.58	1	41	3840	masculin		19064.37	176	69.8	126.5	81	99	182	77	95	62	0.5777	0.5781
5	1 ou -	1-14 tod	1.57	3	42	2970	feminir	65		157.35		104	66.5						0.5886	
12	6.1-10	n„o		1	39	3800	feminino													
3	1 ou -	n„o		2		4360	mascul	45	33201.21	181.75	98	126	75	78	268	58	183	100	0.5973	0.5775
7	1.1-3	n„o	1.66	1	39	3340	masculin		4797.972	177.45	64.2	120.5	71.5	91	175	61	100	62	0.5782	0.5781
4	3.1-6	n„o	1.62	2	40	3900	feminir	114												
4	1.1-3	n„o	1.58	2		3430	mascul	19												
0	1 ou -	n„o	1.57	1		2950	feminino													
13	3.1-6	n„o	1.63	1	40	4000	feminino													
1	1 ou -	15 + tod	1.54	3	43	3130	feminir	41	32764.92	161.6	78.4	117	72.5	76	252	72	166	62	0.5881	0.578
16	3.1-6	n„o	1.48	1	40	3820	masculin													
10	3.1-6	n„o	1.51	2	38	2750	feminir	22	18432.55	159.6	52.8	111.5	74	84	184	74	99	45		0.5744
8	1.1-3	n„o	1.57	1	39	3200	masculin													
0	1.1-3	15 + tod	1.51	5		2200	feminir	43	28924.3	159.9	70.8	109	62.5	80	176	45	102	172	0.5863	0.629
0	1 ou -	1-14 tod	1.53	10		3100	mascul	99	30517.48	174.5	87.5	131	75.5	83	176	46	110	108	0.5829	0.5946
12	1.1-3	n„o	1.61	2	39	3500	mascul	27												
0	3.1-6	n„o	1.56	3		3110	mascul	140	26563.47	164.6	73.8	122.5	76	91	153	55	88	40		
16	10	n„o	1.55	1	41	2800	masculin		23273.56	166.1	77.7	102.5	66.5	107	221	72	132	111	0.5874	0.5752
1	1 ou -	15 + tod	1.51	4	39	3110	mascul	13												
0	1 ou -	15 + tod	1.53	1	43	2700	feminino		42205.56	160.65	88.8	131	87.5	107	196	48	130	76	0.5868	0.5947
6	1.1-3	n„o	1.55	2	42	3000	mascul	36												
8	3.1-6	n„o	1.44	1	38	2720	feminino													
5	1.1-3	n„o	1.62	3	39	3350	mascul	18	18968.29	166.6	69.5	122	75	107	243	63	157	93	0.6104	0.573
0	1 ou -	1-14 tod	1.56	5	39	3160	mascul	34												
7	1 ou -	n„o	1.5	1	38	3860	masculin			177.55	123.4	135.5	97	88	277	49	199	125	0.5937	0.5906
16	6.1-10	1-14 tod	1.54	1	40	2790	feminino		14251.93	162.95	53.9	98	55.5	84	178	61	100	91	0.5663	0.5768
2	1.1-3	n„o	1.54	2	41	3400	feminir	117												
5	1.1-3	1-14 tod	1.47	1		1520	masculin		18016.9	163.15	63.7	133	73.5	119	198	62	118	94	0.59	0.59
0	1 ou -	n„o	1.39	8	42	2970	feminir	24	21404.05	154.1	56.4	125.5	80.5	89	149	48	92	66	0.5801	0.5615
4	1 ou -	n„o	1.54	1	41	3230	feminino													
12	10	n„o	1.69	5	38	3150	mascul	51	19638.47	184.45	85.7	126	58	77	262	55	180	106	0.5723	0.5866
5	1 ou -	n„o	1.53	5	37	2850	feminir	75	17038.67	156.65	53.7	117	75.5	82	220	52	150	108	0.5784	0.5786
5	1.1-3	1-14 tod	1.57	2	39	3200	mascul	55												
8	3.1-6	n„o	1.54	1	39	2950	feminino													
2	1 ou -	n„o	1.56	7	39	3340	feminir	26												
5	3.1-6	n„o	1.55	3		3200	feminir	21	44430.18	174	93.4	116.5	73.5	110	185	43	124	73		
10	3.1-6	1-14 tod	1.51	6	39	2400	feminir	12												
4	1 ou -	15 + tod	1.55	6		2970	mascul	58	4029.875	170.6	70	129.5	76.5	77	120	44	68	36	0.5785	0.5703
5	1.1-3	15 + tod	1.58	1	39	3100	masculin													
5	1.1-3	n„o	1.52	5		3670	mascul	39												
5	1 ou -	1-14 par		4		2470	masculin													
11	1.1-3	n„o	1.57	1	39	3250	masculin													
6	3.1-6	1-14 tod	1.54	3	41	3200	feminir	46	11304.05	164.9	49.6	115.5	78	84	230	92	124	84	0.5799	0.5797
5	3.1-6	n„o	1.56	2	38	3520	feminir	77												
0	1 ou -	1-14 par	1.5	8		2600	feminir	11												

pescmae	prenda	pfumomae	paltmae	pgesta	pidgest	ppn	psex	pint	dmgtotdxa	daltura2012	dpeso	dsysmed	ddiamed	dglicose	dcolesterol	dhdl	dldl	dtrig	dECMICE	dECMICD
0	1 ou -	n„o	1.6	3		2150	feminino													
10	3.1-6	1-14 tod	1.6	2	42	2850	feminir	29	25847.35	164.65	65.4	124	85	84	167	74	80	75	0.5781	0.5743
0	1 ou -	n„o	1.5	2	40	2830	feminir	187	22761.84	151.85	63.9	95	61	88	145	41	91	87	0.578	0.6256
8	1 ou -	1-14 par	1.41	1	42	2810	feminino		29518.84	157.25	69.9	117	78	62	169	57	88	103	0.5798	0.5922
11	10	n„o	1.44	3	41	3150	mascul	49												
9	3.1-6	n„o	1.62	2	39	3730	mascul	78												
16	6.1-10	n„o	1.51	1	36	2800	feminino													
8	3.1-6	n„o	1.52	1	36	2950	masculin													
5	1.1-3	n„o	1.48	3	40	3750	feminir	71												
5	1.1-3	n„o	1.54	2		2950	feminir	16	19327.23	151.4	56.1	110	67	122	229	57	146	176		
3	1.1-3	1-14 tod	1.55	1	39	2100	feminino		12894	158.1	46.5	104.5	71	77	186	64	110	75	0.5775	0.5782
4	1 ou -	1-14 tod	1.53	2		1380	feminir	99	22868.14	162.6	66	130.5	85.5	85	178	62	100	110	0.5782	0.5762
8	1.1-3	1-14 tod	1.52	2	35	2000	feminir	50	43301.39	168.2	83.4	112.5	76	87	169	53	107	51	0.5532	0.5653
5	1.1-3	n„o	1.68	2	40	3150	masculin		8458.892	188.25	66.6	110.5	62	80	131	43	70	74	0.5595	0.5782
4	1 ou -	1-14 tod	1.49	1	35	1880	masculin													
0	1.1-3	n„o	1.49	7	37	2880	mascul	62												
8	1.1-3	1-14 tod	1.57	3	42	2600	feminir	74	21357.45	164.65	57.6	106.5	68	88	208	70	109	135		
10	1.1-3	n„o	1.67	4	38	3700	mascul	15		185.8	125	132.5	89.5	106	109	33	43	143	0.5781	0.5808
4	3.1-6	n„o	1.61	1		2630	masculin		17790.71	173	74.7	126	87.5	92	219	47	145	138	0.5794	0.5808
12	3.1-6	n„o	1.51	1	39	2980	masculin													
2	1 ou -	n„o	1.55	2	37	3250	feminir	60												
5	1 ou -	n„o	1.49	3	42	3750	mascul	76	21007.67	170.65	78.3	112	79.5	129	120	37	56	142		0.5692
1	1.1-3	n„o	1.43	2		3530	feminino													
14	1.1-3	n„o	1.58	1	37	2400	feminino		33568.8	164.3	70.5	116	76.5	126	172	45	96	141	0.5855	
12	1.1-3	n„o	1.58	2		3070	feminir	45		166.9									0.6522	0.5788
3	1.1-3	n„o	1.44	2	38	2830	feminir	22	22084.13	153.1	58.1	98	66	101	120	46	55	83	0.5719	0.5783
0	1.1-3	n„o	1.48	3	40	3150	feminir	13												
7	1.1-3	n„o	1.6	1		3110	feminino													
0	1.1-3	1-14 par	1.5	2		2910	feminir	10												
5	1 ou -	n„o	1.65	2	39	3600	feminir	69	64677.12	172.95	115.9	116.5	77.5	84	218	69	126	124	0.5744	0.5781
7	3.1-6	n„o	1.53	3	39	3250	feminir	16												
4	1.1-3	1-14 par	1.51	1	31	1450	masculin		2930.167	172.55	60	114.5	58	105	164	70	73	138	0.5777	0.5772
4	1 ou -	1-14 tod	1.53	1	39	2950	feminino		16779.72	162.8	56.6	104.5	75.5	80	237	70	146	98	0.5809	0.6167
1	1.1-3	n„o		2	41	3500	mascul	27												
1	3.1-6	n„o	1.63	1	38	2980	feminino		40470.79	165.4	81.4	110	67.5	95	206	78	88	197	0.5803	0.6
5	1 ou -	n„o	1.5	1	40	2790	feminino		13072.01	142.85	46.3	101.5	62.5	87	178	48	116	93	0.5655	0.5738
11	1.1-3	n„o	1.54	2	40	3850	mascul	10	10481.04	183.8	69	114	71	87	169	62	85	128	0.5741	0.5787
14	6.1-10	n„o	1.68	1	40	3440	feminino													
4	1.1-3	1-14 tod	1.65	6	36	2450	mascul	95	15882.03	175.3	74.3	132.5	85	85	223	55	147	84	0.5783	0.5755
4	1 ou -	n„o	1.56	5	39	3730	mascul	59	11816.18	162.05	62.4	106	62.5	64	135	35	78	99	0.578	0.5593
2	1 ou -	n„o	1.52	3	41	3220	feminir	34	26107.83	155.1	64.6	105.5	68.5	78	182	53	111	96	0.6163	0.5779
12	6.1-10	n„o	1.58	4	35	2830	mascul	42												
3	1 ou -	1-14 tod	1.55	3		3350	mascul	25	10128.26	178.6	71.1	122.5	81.5	82	177	80	82	47	0.5448	0.5391
0	1.1-3	n„o	1.53	2		2890	feminir	41												
17	1.1-3	n„o	1.58	2	37	2920	mascul	16												
6	1.1-3	n„o	1.54	6		3330	mascul	37												
11	6.1-10	n„o	1.58	2		2000	masculin													
8	3.1-6	n„o	1.59	2	39	3430	feminir	69												

pescmae	prenda	pfumomae	paltmae	pgesta	pidgest	ppn	psex	pint	dmgtotdxa	daltura2012	dpeso	dsysmed	ddiamed	dglicose	dcolesterol	dhdl	dldl	dtrig	dECMICE	dECMICD
7	1 ou -	n„o	1.45	2	39	2370	feminir	20												
5	1 ou -	15 + tod	1.62	5	41	3420	feminir	14												
0	1.1-3	n„o	1.58	3	42	3300	mascul	50	2286.137	164	47.5	130.5	75.5	101	204	60	125	97	0.5766	0.5732
7	1.1-3	n„o	1.44	1	40	2950	masculin													
11	1 ou -	1-14 tod	1.6	1	39	3540	masculin													
15	10	n„o	1.48	1	30	1300	feminino													
1	1 ou -	n„o	1.54	2	37	3300	feminir	48				114.5	70	76	159	71	70	88		
3	1.1-3	1-14 tod	1.58	6	39	3150	feminir	156												
5	1.1-3	n„o	1.42	3	42	3430	mascul	61	41164.59	163.45	104.8	137.5	88	86	218	54	144	116	0.5838	0.5779
7	1.1-3	1-14 tod	1.49	1	37	3320	masculin		25944.34	174.15	79.5	134	82.5	94	245	46	161	168	0.5728	0.5708
5	3.1-6	1-14 tod	1.55	1	38	3200	feminino		24300.76	166.8	62.8	116	76.5	88	210	70	120	85	0.5781	0.5794
4	1.1-3	n„o	1.56	1		2920	feminino													
5	1.1-3	n„o	1.48	2	40	3100	mascul	12												
8	3.1-6	n„o	1.7	2	42	3070	feminir	29												
0	1 ou -	n„o	1.52	4	39	3300	mascul	27												
3	1 ou -	1-14 tod	1.49	1	40	2900	masculin													
7	1.1-3	1-14 tod	1.58	1	41	2700	feminino		25801.12	163	65.6	96.5	60	81	155	56	89	50	0.5782	0.5783
18	6.1-10	1-14 tod	1.59	2	39	3650	feminir	24	36301.38	158.8	78.5	107.5	70	83	226	79	131	76	0.6433	0.576
4	1 ou -	1-14 par	1.51	3	39	3400	mascul	34	15725.65	172.25	69.1	113	66	71	173	39	121	82	0.5791	0.5776
5	1.1-3	15 + tod	1.56	2		650	feminir	34												
5	1.1-3	1-14 tod	1.65	3		3400	feminir	39												
4	1.1-3	n„o	1.63	1	40	3200	feminino		19758.82	163.9	60.8	120	69.5	89	170	61	95	80	0.582	0.7431
5	1.1-3	n„o	1.57	8	39	4100	feminir	13	27374.84	168.25	74.3	124	67.5	89	170	62	90	85	0.58	0.5799
3	1.1-3	n„o	1.5	8		3750	feminir	109												
2	1.1-3	n„o	1.51	4	41	3540	mascul	27	13699.75	171.8	66.3	140.5	73.5	89	179	47	103	122		0.5824
5	1.1-3	15 + tod	1.5	3	39	2880	mascul	50												
6	1.1-3	1-14 tod	1.53	3	41	3500	mascul	14	25058.15	171.5	83.8	125	70.5	91	189	45	116	138	0.5672	0.5693
6	1 ou -	n„o	1.61	5		2270	mascul	25												
4	1.1-3	n„o	1.5	1	39	3590	masculin		8831.539	175.3	74.6	126	61.5	80	161	51	93	62	0.577	0.5786
7	1.1-3	n„o	1.51	1	39	2850	feminino		13245.14	153.15	50.2	121	75	90	147	54	78	83	0.5786	0.5832
0	1 ou -	n„o	1.55	5	43	3230	mascul	25	26360.79	170.7	88.3	127	67	107	223	46	137	217	0.5708	0.5761
16	10	1-14 par	1.56	3	41	3200	feminir	14												
5	1.1-3	1-14 tod	1.45	1		2790	feminino													
5	1.1-3	n„o	1.55	3	41	3400	mascul	37	21046.3	163.9	81.3	121.5	73	105	256	54	176	166	0.5886	0.5987
5	1 ou -	n„o	1.58	2		3200	feminir	15	39451.04	165.45	96.9	125	78	99	174	62	95	74		0.5756
5	1.1-3	1-14 tod	1.51	3		2940	mascul	74	8159.039	173.4	61.1	133	73	105	170	61	76	367	0.578	0.5715
20	10	n„o	1.65	3	39	3700	feminir	30	17368.98	163.6	57.2	124	74.5	55	164	74	72	60	0.5787	0.5548
3	1 ou -	1-14 tod		1		1640	masculin													
11	3.1-6	n„o	1.6	1	41	3850	masculin													
2	1 ou -	n„o	1.58	1		700	feminino													
4	1.1-3	1-14 tod	1.65	1	39	3540	masculin													
14	1.1-3	1-14 par	1.46	1	42	2850	masculin		32373.18	156.95	76.1	134.5	82	112	206	70	124	50		0.6059
0	1.1-3	1-14 tod	1.59	12	37	3270	mascul	49												
7	6.1-10	1-14 par	1.55	4		2670	feminir	33	35970.78	155.1	75.9	132.5	83	108	168	53	102	61	0.5527	
0	1 ou -	1-14 tod	1.58	2		3830	mascul	30												
8	3.1-6	n„o	1.55	3	41	2750	feminir	61												
7	6.1-10	n„o	1.66	1	39	4050	masculin		19399.68	180.15	75.1	127	80.5	103	219	72	116	201	0.5584	0.5658
5	1.1-3	n„o	1.58	1	39	2750	feminino		25624.62	162.7	67.5	94.5	61.5	89	210	63	128	124	0.6036	0.5788

pescmae	prenda	pfumomae	paltmae	pgesta	pidgest	ppn	psex	pint	dmgtotdxa	daltura2012	dpeso	dsysmed	ddiamed	dglicose	dcolesterol	dhdl	dldl	dtrig	dECMICE	dECMICD
4	1.1-3	1-14 tod	1.6	1	39	3150	feminino		40715.55	165.15	86.9	110	71	79	206	60	135	60		
4	1 ou -	15 + tod	1.6	11	40	3500	mascul	151												
4	1 ou -	n„o	1.45	1	40	2290	masculin													
15	10	n„o	1.62	1	40	4050	masculin													
4	1.1-3	n„o	1.5	2	40	3200	mascul	44	4581.169	167.8	52.7	124.5	82	95	179	53	105	77	0.5529	0.5777
4	1.1-3	n„o	1.54	3		3800	mascul	72												
8	3.1-6	n„o	1.5	2	38	3250	feminir	39												
4	1 ou -	15 + tod	1.47	2	40	2650	mascul	27	15754.14	167.25	73.5	115.5	65.5	150	318	49	135	405	0.5855	0.5755
7	1.1-3	1-14 tod	1.66	2	40	2550	feminir	51	29331.6	165	71.3	97.5	61.5	84	150	65	73	68	0.5778	0.5782
3	1.1-3	n„o	1.63	2	41	3120	feminino		31958.6	170.1	79.6	112	64	77	213	84	103	134	0.5784	0.5712
10	1.1-3	1-14 tod	1.5	1	40	2460	feminino		16587.46	157.2	56	142.5	95.5							
9	1.1-3	1-14 tod	1.51	1	39	3320	masculin													
12	1.1-3	n„o	1.6	1		3550	feminino		50841.7	165.55	97.8	127	82	73	248	99	116	86		0.5777
11	6.1-10	n„o	1.57	2	39	3000	mascul	18	27127.96	180.85	94	118	75	145	260	34	102	637	0.5959	0.5879
8	1.1-3	n„o	1.52	3	37	3900	masculin													
5	1.1-3	n„o	1.62	4	42	3400	feminir	134	33547.09	166.55	83.6	129	85.5	81	159	50	100	57	0.5808	0.5757
11	6.1-10	n„o	1.67	4	37	3540	feminir	35												
8	3.1-6	1-14 tod	1.58	2	37	3150	feminir	41												
16	3.1-6	n„o	1.62	2	40	3750	mascul	17												
11	1.1-3	1-14 tod		1	38	2720	masculin		20583.53	175.3	79.6	123	76	87	199	76	105	99	0.5795	0.5783
5	1.1-3	n„o	1.55	11	40	3900	mascul	20	4095.654	167.1	67.7	131	78.5	108	150	77	60	51	0.5776	0.5786
5	1.1-3	n„o	1.53	2		3150	mascul	12												
8	1 ou -	n„o	1.53	2	36	3100	mascul	32												
7	1.1-3	n„o	1.67	3	37		feminir	90	23173.85	162.75	68.1	106	71	67	122	35	71	56	0.577	0.5724
14	10	1-14 tod	1.56	1	40	3250	masculin		25182.27	175.5	83.1	104	68.5	78	202	56	120	140		
10	1.1-3	1-14 par	1.5	2	39	4200	mascul	70	25028.32	175.7	86.2	124	75.5	121	289	53	184	457	0.586	0.5967
1	1.1-3	1-14 par	1.7	6	38	2700	mascul	52												
16	10	n„o	1.5	3	39	3400	feminir	17	21933.27	162.5	61	115.5	74	73	190	79	89	76	0.5781	0.5813
5	1 ou -	1-14 tod	1.54	2	37	3400	feminir	20	27673.66	164.6	69.3	91.5	57	67	200	78	107	61	0.5775	0.5779
6	1.1-3	n„o	1.57	1	37	3100	masculin		24619.05	173.25	84.8	114.5	69.5	69	190	50	124	92	0.5767	0.5854
6	1 ou -	1-14 tod	1.55	3		3080	mascul	61	17313.72	164.85	65	133.5	66	95	176	63	85	160	0.547	0.5749
12	1.1-3	1-14 tod	1.72	2	40	3200	mascul	60	18764.85	187.7	85.5	140.5	90						0.5753	0.5703
7	1.1-3	1-14 tod	1.58	5	39	3100	feminir	113												
5	3.1-6	1-14 tod	1.6	5	40	2050	mascul	119	28911.62	171.9	85.2	113	62.5	75	189	49	120	142	0.6042	0.5788
5	1.1-3	1-14 tod	1.54	3	40	3650	mascul	24	8147.342	173.05	80.9	126.5	70	82	185	62	98	116	0.59	0.604
5	3.1-6	1-14 tod	1.49	3	38	2650	mascul	17												
2	1 ou -	n„o		3		3080	feminir	38												
5	1 ou -	n„o	1.44	4		2490	mascul	31												
8	1.1-3	n„o	1.66	1	38	2860	masculin		8644.475	182.5	67.6	150	78.5	105	169	85	66	67	0.6227	0.6435
14	3.1-6	n„o	1.55	3	38	2920	feminir	84												
6	1 ou -	n„o	1.48	1	41	2920	masculin													
4	1 ou -	n„o	1.53	1	41	3000	feminino		17123.88	158.65	46.6	119	87.5	113	171	62	95	66	0.5778	0.585
6	1.1-3	1-14 tod	1.7	1		3220	masculin		27423.52	183.05	94.4	126	68	89	211	56	139	89	0.5902	0.589
4	1.1-3	1-14 par	1.62	1	42	3150	feminino		42090.05	166.05	85.1	123	80	86	217	64	140	102	0.5781	0.5775
6	1.1-3	1-14 tod	1.63	2	38	2280	feminir	17	47837.83	174.65	104.1	123	77	97	205	56	138	80	0.62	0.59
1	1 ou -	1-14 tod	1.46	4		3500	mascul	24	2762.875	162.8	52.9	128	69.5	86	152	51	82	57	0.578	0.5782
8	1.1-3	n„o	1.53	1		3000	masculin		14641.03	177.3	71.3	118.5	68	63	156	72	74	43	0.5589	0.579
3	1.1-3	n„o	1.58	1		2930	feminino													

pescmae	prenda	pfumomae	paltmae	pgesta	pidgest	ppn	psex	pint	dmgtotdxa	daltura2012	dpeso	dsysmed	ddiamed	dglicose	dcolesterol	dhdl	dldl	dtrig	dECMICE	dECMICD
6	1.1-3	n,,o	1.58	1	40	3300	feminino		48954.14	155.9	93.8	123.5	84.5	87	276	79	162	75	0.5772	0.5838
10		n,,o	1.46	1	39	3320	masculin		25544.04	170.55	87.7	139	66.5	70	218	70	125	159	0.5772	0.5745
4	1 ou -	n,,o	1.59	2		2950	mascul	23	6199.546	171.65	70.9	126.5	59.5	84	164	45	92	122	0.5582	0.575
8	1.1-3	n,,o	1.59	4	41	3900	mascul	15	17759.22	182.4	86.5	124	77.5	75	180	47	106	164	0.5973	0.6043
4	3.1-6	n,,o	1.55	1	40	3000	feminino		18351.01	164.5	56.7	112	71.5	97	236	78	137	127		
4	1 ou -	n,,o	1.59	2	39	2140	feminir	34												
2	1.1-3	n,,o	1.52	12		3250	feminir	21												
9	3.1-6	n,,o	1.56	2	38	2700	mascul	33	36807.75	178.8	103.8	148.5	96	89	244	43	166	219		0.5751
5	6.1-10	15 + tod	1.47	2	42	3400	mascul	168	30847.54	172.85	89.3	131.5	85	82	179	65	100	74	0.5806	0.6622
8	1.1-3	1-14 tod	1.61	3		3650	mascul	26												
4	3.1-6	n,,o	1.55	3	36	2750	feminir	58												
9	3.1-6	15 + tod	1.63	1	39	3040	masculin													
4	1.1-3	1-14 tod	1.63	2	42	2790	mascul	10		172.8	134.8	122	81	137	326	45	177	526	0.59	
7	1.1-3	n,,o	1.53	3	40	3520	feminir	42												
6	1.1-3	1-14 par	1.42	2	40	3330	mascul	27	33285.56	162.6	94.8	126.5	78	94	193	42	99	233	0.5804	0.584
5	1.1-3	1-14 par	1.55	1	37	1790	masculin		25190.81	159.3	77.4	138	85	130	152	54	88	39	0.5779	0.5787
4	1.1-3	15 + tod	1.61	2	37	2800	feminir	82												
6	1 ou -	1-14 tod	1.59	1	41	3660	masculin		9222.075	169.35	63.4	121.5	66.5	99	200	51	120	186	0.576	0.5726
2	1 ou -	n,,o	1.52	2	41	3850	mascul	44	21631.19	167.2	73.5	98	61						0.667	0.5722
16	10	1-14 tod	1.56	4	39	3760	mascul	44	43591.38	176.8	111.2	132.5	83	92	200	64	108	133	0.5814	0.5782
3	3.1-6	1-14 tod	1.57	1		2500	feminino													
6	3.1-6	n,,o	1.48	1	35	2300	masculin		31090.54	162.4	88.8	131.5	84	98	209	52	126	135	0.5785	0.6502
6	1.1-3	n,,o	1.53	1	42	3200	feminino		30875.89	155.4	69.4	109.5	73.5	89	157	47	97	55	0.5496	
1	3.1-6	n,,o	1.44	3	40	2550	mascul	38												
4	1 ou -	n,,o	1.55	1	38	2300	feminino													
15	10	1-14 tod	1.63	1	39	4100	masculin		27386.29	179.4	92	122.5	77	84	159	38	87	145	0.5808	0.6464
11	6.1-10	1-14 tod	1.58	1	37	3000	feminino			156.9		91.5	57.5						0.5664	0.5684
5	1.1-3	n,,o	1.5	4	40	3360	feminir	35	39464.72	155.2	94.2	116	75.5	68	281	74	189	131	0.5888	0.5912
4	1 ou -	1-14 tod	1.5	4	42	3600	mascul	61	10502.35	163.3	69.2	128	70.5	90	170	57	100	76	0.5807	0.5793
5	1.1-3	15 + par	1.5	2	40	2600	feminino		36870.04	156.65	73.6	117.5	83.5	79	195	58	117	119	0.587	
4	1.1-3	1-14 tod	1.51	1	42	2790	feminino		32589.37	154.45	70	105	68	65	182	52	118	48	0.5818	0.5756
5	1 ou -	n,,o	1.48	1		2550	masculin													
12	3.1-6	n,,o	1.52	3	39	3000	mascul	35	29793.16	179.5	92.4	127.5	94	150	226	46	114	577	0.5856	0.5763
17	6.1-10	n,,o	1.49	1		2500	feminino		20453.47	154.65	56.8	106.5	71.5	78	220	71	131	118	0.5736	0.5822
4	1 ou -	n,,o	1.53	1	38	2950	feminino													
10	1.1-3	n,,o	1.52	2	40	3450	feminino		29343.98	162	68.4	112.5	79.5	87	196	70	109	156	0.5291	0.5784
5	1 ou -	n,,o	1.56	2		3240	mascul	21	5998.272	168.95	74.2	136	87.5	83	207	70	120	59	0.5981	0.6101
3	1.1-3	n,,o	1.59	1	40	3450	masculin		25050.05	170.1	87.1	123	77.5	86	255	68	162	127	0.6001	0.5882
3	1 ou -	n,,o	1.63	1	40	2750	feminino													
12	6.1-10	n,,o	1.57	1	40	3770	feminino													
2	1 ou -	n,,o	1.54	6	40	3950	mascul	16	18720.17	179.8	86.5	130	75	75	230	42	141	264	0.5942	0.6019
7	3.1-6	n,,o	1.55	2	41	3000	mascul	142	16027.58	162.2	67	118	78	80	164	62	93	68	0.5664	0.5731
4	1 ou -	n,,o	1.59	4	39	2800	feminir	11												
3	1 ou -	1-14 tod	1.46	4	38	3570	mascul	109	31815.21	169.5	106.6	126.5	74.5	91	209	36	121	281	0.5769	0.5933
5	1.1-3	n,,o	1.52	2	41	3230	mascul	45	16926.65	165.3	65.1	121	72	82	158	57	90	78	0.5729	0.5659
9	1.1-3	1-14 tod	1.7	1	43	4700	feminino		57197.6	170.1	113.6	123	83.5	118	153	54	78	75	0.6043	0.5757
9	3.1-6	n,,o	1.56	4	38	3450	mascul	48												
5	1.1-3	1-14 tod	1.58	1	38	2800	masculin		12977.74	170.9	73.4	140	79.5	80	212	80	120	66	0.5783	0.5782

pescmae	prenda	pfumomae	paltmae	pgesta	pidgest	ppn	psex	pint	dmgtotdxa	daltura2012	dpeso	dsysmed	ddiamed	dglicose	dcolesterol	dhdl	dldl	dtrig	dECMICE	dECMICD
7	1 ou -	n„o	1.53	2	40	2360	feminir	14												
5	1.1-3	1-14 tod	1.5	1	41	2670	feminino		34086.34	156	72.9	109.5	67.5	85	159	43	85	183		
6	1.1-3	n„o	1.5	6	36	2550	feminir	21	22921.02	166.2	59.8	98	66.5	58	159	53	94	54		0.5719
5	1.1-3	n„o	1.57	10	43	3060	feminir	86												
9	10	n„o	1.68	1	41	3650	feminino		22969.37	169.1	67.9	125.5	78.5	63	195	64	117	82	0.5373	0.5806
4	3.1-6	n„o	1.56	1		3600	masculin													
5	1.1-3	1-14 tod	1.47	1	40	2950	feminino													
5	1.1-3	n„o	1.54	3	40	4120	feminir	95												
4	1.1-3	n„o	1.51	7		4000	mascul	124												
3	1.1-3	n„o	1.45	1	41	2650	feminino		26568.38	160.1	70.2	113	75	85	214	62	130	118	0.5819	0.5781
7	1 ou -	n„o	1.58	3	41	3900	mascul	22	29251.8	170.8	92.4	137.5	98.5	82	245	61	170	75		
0	1 ou -	n„o	1.39	1	40	2390	feminino													
10	3.1-6	n„o	1.51	2	38	2500	feminir	22												
5	1 ou -	n„o	1.66	1	41	3200	masculin													
5	1 ou -	n„o	1.58	1	40	2880	masculin													
8	3.1-6	n„o	1.6	3	40	3830	feminir	60	23567.55	160.35	60.9	106	70	250	172	62	75	172	0.5577	
9	3.1-6	n„o	1.58	1	39	3340	feminino		15743.66	167.35	56.4	116	67	65	135	48	65	79	0.5781	0.5774
4	1 ou -	n„o	1.58	4	39	3060	mascul	27	32133.41	177.65	99.3	140	83.5	97	195	44	94	310	0.5784	0.5856
3	1 ou -	n„o	1.54	3	40	3450	masculin		22323.78	177.65	86.5	127.5	72	96	240	79	141	123	0.5788	0.5793
5	1.1-3	n„o	1.7	2	39	3300	feminir	69	47146	167.35	94.7	117	81.5	85	165	51	98	79	0.5744	0.5781
5	3.1-6	n„o	1.6	2	40	3010	feminir	11												
5	1.1-3	1-14 tod	1.57	2	41	3330	mascul	12	21689.18	179	81.9	124	68						0.6202	0.5877
5	1 ou -	n„o	1.65	2	40	3550	feminir	70	47563.35	175.6	107.5	139	93.5	91	219	51	143	148		
7	1.1-3	1-14 tod	1.59	1	40	3580	feminino		32109.51	167.1	75.6	100	66.5	106	189	70	109	80	0.577	0.5784
6	3.1-6	1-14 par	1.61	1	41	3330	masculin		35640.64	176.65	100.8	154.5	99	89	247	52	157	230	0.6543	0.5783
6	1 ou -	1-14 tod	1.6	1	38	2900	feminino		19948.05	160.95	58.8	120	64	77	229	91	132	46	0.5744	0.5713
8	3.1-6	n„o	1.59	2		3600	mascul	41	16863.22	168.4	71.2	123.5	80.5	81	252	75	146	175	0.581	0.5784
1	1.1-3	1-14 tod	1.47	2	40	2900	mascul	122												
16	10	n„o	1.55	2	38	3550	feminir	96	38475.65	150.6	79.6	128.5	87	63	200	52	123	136	0.5788	0.5962
12	3.1-6	n„o	1.59	1	42	2570	feminino		13651.74	166.3	52.6	114.5	74.5	99	231	77	136	102	0.5611	
5	1.1-3	1-14 tod	1.57	3	43	3300	feminir	32	14666.13	164.4	53.4	102.5	60	54	152	43	93	68	0.5624	
5	1.1-3	n„o	1.58	2	42	3700	mascul	70	32396.33	155.9	76.3	123.5	80.5	65	168	51	106	51		0.5626
3	1.1-3	n„o	1.58	2	40	2970	feminir	49	25059.41	180.6	72	109	72.5	75	163	64	84	87	0.5781	0.5748
9	1.1-3	1-14 par	1.58	2	39	3350	masculin													
16	10	n„o	1.68	2	39	3500	mascul	14	10915.71	179.7	78.6	131.5	81	75	214	46	127	191	0.6207	0.5938
2	1 ou -	n„o	1.5	1	39	2420	feminino		11271.97	153.1	45.6	117.5	86.5	81	175	64	95	99	0.5804	0.5794
6	1.1-3	1-14 tod	1.46	2	39	3680	feminir	48	16393.05	159.55	57.2	106.5	69.5	83	175	63	100	77		
5	1.1-3	n„o	1.49	10	40	2030	mascul	30												
5	3.1-6	n„o	1.65	2		4200	feminir	39	28277.04	174.65	70.3	106.5	72.5	88	190	92	88	53	0.5478	
5	1.1-3	n„o	1.61	2	38	2800	feminir	39	21703.21	159.15	63.8	108	71.5	89	168	61	100	57	0.5798	
18	3.1-6	n„o	1.7	1	39	3750	feminino		30337.6	168.05	70.6	110	74.5	83	194	73	110	75	0.6336	0.5802
11	1.1-3	n„o	1.57	4	38	2820	feminir	25												
5	1 ou -	1-14 tod	1.45	2		2930	mascul	10	40412.99	166.6	103.9	131	87	84	193	51	128	111		
3	1.1-3	15 + par	1.49	3	38	2960	feminir	108												
9	3.1-6	n„o	1.47	2		2150	mascul	20	20697.37	168.35	69.5	128.5	78.5	86	200	65	120	81		0.5769
12	3.1-6	1-14 tod	1.47	1	39	2980	feminino		20939.98	158.3	57.2	104.5	61.5	57	168	50	103	61	0.5752	0.5773
4	1.1-3	n„o	1.6	4	38	2430	feminir	75												
5	1.1-3	n„o	1.6	1	34	1490	feminino		45552.24	159.15	86	136	92	85	193	69	100	159	0.5782	0.5738

pescmae	prenda	pfumomae	paltmae	pgesta	pidgest	ppn	psex	pint	dmgtotdxa	daltura2012	dpeso	dsysmed	ddiamed	dglicose	dcolesterol	dhdl	dldl	dtrig	dECMICE	dECMICD
16	10	n„o	1.64	2	39	3640	feminir	31												
4	1.1-3	n„o	1.61	1	41	3400	feminino		40930.7	173.1	93.8	120.5	80.5	80	202	57	134	71	0.5914	0.5922
3	1.1-3	n„o	1.53	1	41	2940	masculin		27360.37	177	88.4	115.5	74.5	100	215	72	121	101	0.6053	0.5804
1	1 ou -	1-14 tod	1.54	1		3110	feminino		31816.35	153.95	73.6	121	77	302	187	49	119	122	0.5727	0.5774
6	1 ou -	1-14 tod	1.62	1	42	2910	feminino													
1	1 ou -	1-14 par	1.57	1	37	3370	feminino		22748.05	161.6	64.5	108.5	67.5	70	185	72	96	79		0.5781
5	1.1-3	1-14 tod	1.59	1	40	2750	feminino		37675.56	163.55	80.8	102	64	87	164	54	99	82		
12	3.1-6	1-14 par	1.68	1	38	2960	feminino		35613.54	173.55	84.3	110	67	97	216	74	98	119	0.5753	0.5707
4	1.1-3	n„o	1.47	2	43	3220	mascul	56	20811.71	172.65	86.9	128	72.5	93	210	48	132	171	0.6358	0.5793
9	1.1-3	n„o	1.59	2	41	3700	feminir	60												
5	1 ou -	1-14 tod	1.47	2	43	3400	mascul	29												
8	1 ou -	1-14 par	1.59	2		3100	feminir	18												
16	3.1-6	n„o	1.56	4	38	2770	feminir	60		152.25	56.6	104	71	85	216	79	100	169		
7	1.1-3	1-14 tod	1.5	1	39	3200	masculin		23133.32	171	74.6	130	83	87	196	49	118	166	0.5888	0.607
2	1.1-3	1-14 tod	1.58	2	32	2620	mascul	74	10494.09	173.25	73.2	117	71.5	89	204	67	125	48	0.6316	0.599
7	1.1-3	n„o	1.61	2	40	3100	feminir	40												
12	6.1-10	15 + tod	1.56	4	39	3700	mascul	75	20359.35	179.8	85	135.5	83	179	246	30	67	539	0.6041	0.5852
20	10	n„o	1.57	2	41	3350	feminir	28	22781.58	161.65	60.7	132	83.5						0.5742	0.5783
4	1.1-3	n„o	1.52	2		3050	feminir	14												
9	1 ou -	n„o	1.65	2	38	3660	mascul	16	19028.82	175.6	74.6	115.5	71.5	95	172	65	84	109		
18	6.1-10	n„o	1.61	2	38	3350	feminir	31	30001.63	167.15	74.2	121.5	80	77	301	76	203	162		0.5797
4	1.1-3	n„o	1.55	1	39	4000	masculin													
5	1.1-3	n„o	1.57	5		1320	feminir	22												
7	1.1-3	n„o	1.46	1	40	2710	feminino													
13	6.1-10	15 + tod	1.63	1	39	3440	masculin		13833.3	177.9	79.1	127.5	75	89	230	71	141	117	0.5802	0.6039
16	6.1-10	n„o	1.51	3	39	3730	feminir	59		165									0.5764	0.5822
4	1.1-3	n„o	1.58	2	39	3650	feminir	133	51143.77	158.15	105.1	126	83.5	88	270	94	158	96		
3	1.1-3	n„o	1.61	1		3350	masculin													
4	1.1-3	n„o	1.41	2	38	3000	mascul	19	12066.69	168.15	64.5	120.5	66.5	85	219	61	135	109	0.6031	0.5759
6	1.1-3	15 + tod	1.6	3	41	2900	mascul	88	28770.2	174.4	90	110	65.5	88	210	54	140	76	0.5697	0.5934
7	1.1-3	1-14 tod	1.56	3	39	3350	feminir	38	21614.67	154.55	59.1	93.5	53.5	71	180	62	99	84	0.5776	0.5746
3	1.1-3	n„o	1.57	3	38	2800	mascul	40												
7	3.1-6	n„o	1.58	1	41	4000	masculin		19088.96	179.2	83.9	117	70.5	72	205	54	139	75	0.5983	0.5879
7	1.1-3	1-14 tod	1.54	1	39	3070	feminino		11703.88	162.6	47.7	99	72	113	134	46	50	179	0.5762	0.5776
7	1.1-3	n„o	1.5	1	39	3800	masculin													
8	1.1-3	1-14 tod	1.62	6		3150	feminir	13	21450.12	160.2	63.4	106.5	67.5	87	271	60	175	171	0.6542	0.5783
17	10	1-14 tod	1.67	2	40	3900	feminir	27												
12	1.1-3	n„o	1.49	2	37	2970	feminir	53	29411.81	158.4	72.2	119.5	77	86	163	65	90	42	0.5744	
6	1.1-3	n„o	1.54	4	39	3500	mascul	35												
0	1 ou -	n„o	1.54	12		2790	feminir	32												
3	1 ou -	n„o	1.53	2	41	2680	feminir	22												
11	6.1-10	n„o	1.5	1	41	3310	masculin													
5	3.1-6	n„o	1.51	2	41	2950	mascul	25												
3	1.1-3	n„o	1.62	1	40	3120	masculin		35698.68	173.2	93.5	138	91	81	220	44	141	208		
5	10	n„o	1.59	4	38	3000	mascul	14	14277.61	170.1	66.9	121.5	65.5	89	185	63	99	127	0.5777	0.5789
4	1.1-3	1-14 tod	1.54	2	40	3660	feminir	20	20196.62	157.7	60.2	121	75	79	185	41	115	147	0.578	0.5782
16	10	n„o	1.57	4	40	3500	feminir	26	36105.64	160.7	72.3	106	73	74	245	89	130	126	0.5737	0.5798
9	10	15 + tod	1.61	2	41	3100	mascul	26	29422.27	181.9	94.4	124.5	66.5	81	198	67	107	106	0.5803	0.5782

pescmae	prenda	pfumomae	paltmae	pgesta	pidgest	ppn	psex	pint	dmgtotdxa	daltura2012	dpeso	dsysmed	ddiamed	dglicose	dcolesterol	dhdl	dldl	dtrig	dECMICE	dECMICD
4	1.1-3	1-14 tod	1.56	1	42	3150	masculin													
10	3.1-6	1-14 par	1.6	1	42	3360	feminino		21269.59	155.35	55.2	129.5	87.5	81	173	78	83	73	0.5751	0.5787
4	1.1-3	n„o	1.53	7	39	3540	mascul	43												
3	3.1-6	n„o	1.47	4	40	3120	feminir	37	18313.81	160.5	52.6	99.5	67	82	175	73	89	66		
11	1.1-3	n„o	1.55	2	40	2800	mascul	57	16840.29	181.6	92	132	68.5	87	198	52	112	168	0.7681	0.5825
6	1.1-3	1-14 tod	1.64	1	39	2980	masculin		39472.02	171.85	101.4	124	70.5	82	212	45	134	177		
0	1 ou -	15 + tod	1.6	6	43	2100	feminir	74	25779.92	162.15	66.8	88.5	52.5	88	234	55	148	176		
6	1 ou -	n„o	1.42	1	39	3030	masculin		11339.73	163.8	66.3	126.5	81.5	91	166	56	98	62	0.5714	
0	1.1-3	n„o	1.5	4	40	3510	mascul	60	4854.583	173.65	63.7	124.5	69	76	156	52	82	98	0.6033	0.5818
5	1.1-3	n„o	1.56	6		4020	feminir	62	28483.23	159.8	71.4	125.5	81	91	212	55	138	100	0.5804	0.5894
12	6.1-10	n„o	1.59	1	41	3000	feminino		23954.71	162	63.7	104	64	83	180	76	98	43	0.5784	0.578
8	1.1-3	n„o	1.61	1	41	3150	masculin		21200.46	172.65	87.4	141.5	88	89	219	35	136	171	0.6483	0.6716
5	1.1-3	1-14 tod	1.64	1	37	3400	masculin		51455.94	178.05	115	165.5	106	86	199	51	113	219	0.5787	0.5746
5	3.1-6	1-14 tod	1.6	2	39	3600	mascul	21		185	124.1	149	87	84	250	48	158	242	0.579	
6	1.1-3	1-14 tod	1.55	1	40	3170	masculin		29823.07	167.1	87.3	120	77	69	217	58	139	108	0.5819	0.5793
9	1.1-3	n„o	1.57	2		800	feminino													
12	6.1-10	n„o	1.7	1	40	3070	feminino													
5	1 ou -	n„o	1.6	1	39	2840	masculin		24657.4	166	80.9	121.5	53.5	65	152	43	95	57	0.5781	0.5715
5	6.1-10	n„o	1.53	3	42	4740	mascul	42												
9	10	n„o	1.56	4	39	3600	mascul	47	14654.54	168.1	72.4	128.5	71.5	106	219	56	148	59	0.5936	0.5787
5	1 ou -	n„o	1.51	1		3360	feminino													
6	3.1-6	15 + tod	1.51	2	39	3250	mascul	30												
7	3.1-6	1-14 par	1.54	3	41	3970	mascul	26	29143.26	177.8	93.1	126.5	77.5	77	198	64	123	73	0.5784	0.573
3	1 ou -	n„o	1.65	7		2450	mascul	22												
2	3.1-6	n„o	1.45	1		2850	feminino		7880.606	147.4	43.8	99	61	97	175	70	90	75	0.56	0.57
4	1 ou -	n„o	1.64	3		1760	mascul	12												
7	1.1-3	1-14 tod	1.54	3		2710	mascul	33	15668.1	168.45	69.9	110.5	75.5	75	232	44	147	267	0.5782	0.578
10	3.1-6	n„o	1.69	2	39	3170	feminino		36238.78	160.5	78.2	103.5	70	74	205	65	125	63	0.5738	0.5796
8	1 ou -	n„o	1.57	1	40	3330	feminino													
14	6.1-10	n„o	1.52	1	38	3150	feminino		39562.34	151	73.5	106.5	71.5	95	218	53	153	88	0.584	0.5771
5	6.1-10	n„o	1.5	3	38	3650	feminir	39	11427.6	154.35	49.1	115.5	70.5	93	108	47	48	60	0.5781	0.5781
10	3.1-6	n„o	1.6	2	39	3300	feminir	64	22873.3	149	59.7	97	63	79	186	69	101	74	0.5783	0.578
1	1.1-3	n„o	1.53	1	42	3600	masculin													
2	3.1-6	n„o	1.61	8	40	3800	mascul	168												
9	1 ou -	n„o	1.54	3	43	3270	mascul	28	23032.01	165.05	82.3	131.5	77	113	180	45	105	157	0.6536	0.6803
4	1.1-3	n„o	1.6	5	38	3350	feminir	48	12109.07	168.85	63.4	120.5	73.5	71	155	57	75	110	0.5782	0.5417
9	3.1-6	n„o	1.68	4	37	3770	feminir	73	45712.97	170.3	98.1	117	71.5	95	175	61	97	84	0.6199	
8	1.1-3	1-14 tod	1.58	2	41	3300	mascul	57	23644.9	187.2	101	147.5	82	102	235	50	159	115	0.5813	0.5977
1	1 ou -	n„o	1.52	2		2850	mascul	69	3151.021	168.3	54.1	131.5	83.5	66	172	64	98	51	0.5773	0.5754
17	10	n„o	1.62	1	38	3150	masculin		30001.28	178.4	109.5	177	87	108	266	57	159	278	0.569	0.5603
8	1 ou -	1-14 par	1.58	4	39	2050	mascul	29	21783.92	163.75	79.1	137.5	81.5	104	221	53	143	122	0.5807	0.586
12	1.1-3	n„o	1.57	1	40	2350	feminino		20912.13	163.55	60.3	108	78.5							
10	10	1-14 par	1.56	2	38	3550	mascul	20	22194.42	173.6	82.1	118	75	92	217	50	122	245		
5	10	n„o	1.61	1		3110	masculin		25173.83	178.1	97.9	142	82.5	109	230	50	124	250	0.5929	0.5547
5	1 ou -	n„o	1.5	4	38	3370	feminir	36												
5	1.1-3	15 + tod	1.53	5		2800	mascul	18	13977.05	173.25	70.6	138.5	72	100	265	48	169	199	0.579	0.5839
5	3.1-6	n„o	1.56	1	36	3800	masculin		27098.15	180.3	94.1	119.5	66	100	146	59	78	65	0.5866	0.5802
3	1.1-3	n„o	1.52	6	34	2490	feminir	46		151.55	44.4	125.5	77.5	76	185	82	90	50		0.5712

pescmae	prenda	pfumomae	paltmae	pgesta	pidgest	ppn	psex	pint	dmgtotdxa	daltura2012	dpeso	dsysmed	ddiamed	dglicose	dcolesterol	dhdl	dldl	dtrig	dECMICE	dECMICD
12	3.1-6	n„o	1.6	2		3300	feminir	24	19478.24	171.4	68.1	106.5	71.5	89	161	78	73	88	0.5781	0.5776
4	1 ou -	15 + tod	1.58	5	40	2630	mascul	12												
2	1.1-3	1-14 par	1.56	3	38	3200	feminir	54	40343.93	165.1	87.1	128	88.5	92	220	49	125	241	0.5783	0.5738
6	1.1-3	1-14 par	1.48	3	38	3400	mascul	39	17294.76	166	74	146	79	68	226	46	156	101		
5	1 ou -	n„o	1.53	4	41	4000	mascul	42												
6	1 ou -	1-14 par	1.61	1	40	3400	feminino		25433.96	167.85	64.5	113.5	84	91	223	99	110	71	0.5778	0.5749
3	1.1-3	n„o	1.53	4	40	3780	mascul	45												
16	1 ou -	1-14 tod	1.69	1		3050	masculin													
6	1.1-3	n„o	1.49	4		2900	mascul	14	6729.936	175.7	64.1	120	74	90	241	114	96	129	0.5783	0.5781
15	6.1-10	n„o	1.65	2	40	3670	feminir	20												
4	1.1-3	1-14 tod	1.57	2		2400	feminir	26	10731.78	161.3	48.7	110.5	72.5	86	152	53	80	58	0.5779	0.5671
11	1.1-3	1-14 tod	1.53	1	42	2780	feminino		31088.58	160.3	70.2	107	69	86	229	70	145	80		
0	1 ou -	1-14 tod	1.44	21	41	2750	mascul	103	3941.458	158.2	49.5	116.5	63.5	102	181	80	77	83	0.5775	0.5801
5	1 ou -	15 + tod	1.52	1		2500	feminino													
15	3.1-6	n„o	1.57	2	38	3760	feminir	55	29239.33	166.2	76	110	70	55	169	79	70	58	0.5715	0.579
3	1.1-3	n„o	1.59	2	40	3350	mascul	24												
8	1.1-3	n„o	1.56	1	40	3250	feminino					106.5	59	90	219	77	136	38		
20	10	1-14 par	1.62	2	37	3000	mascul	48												
6	1.1-3	1-14 tod	1.52	1	39	2820	feminino		40237.22	164.6	83.2	107	72	82	209	64	119	166	0.5725	
6	1.1-3	15 + tod	1.58	3	38	3550	mascul	12	34703.71	166.85	102.1	127	77.5	151	294	36	144	675	0.6538	0.5839
7	1.1-3	n„o	1.5	1		3740	masculin													
0	1 ou -	15 + tod	1.45	3	38	2800	mascul	24		173.8	69.8									
12	3.1-6	n„o	1.51	1	39	3400	feminino		42166.35	159.05	88.1	128.5	75.5	83	189	58	119	71	0.5869	
5	1.1-3	n„o	1.5	1	39	3300	masculin		20257.53	183	79.6	127.5	76.5	77	212	57	138	90	0.5738	0.5773
5	1.1-3	n„o	1.54	6	40	3600	mascul	12	2337.466	172	54.4	137	91	118	185	65	106	56	0.578	0.5786
0	1.1-3	n„o	1.55	1	40	3250	masculin		31271.37	172.2	92.6	148	84	76	200	57	120	154	0.5783	0.5839
14	1.1-3	1-14 tod	1.5	3	40	3230	mascul	52												
7	1.1-3	n„o	1.59	5		1600	mascul	32	4746.801	174.5	57.2	127	67	94	190	64	116	46	0.5781	0.5957
9	1.1-3	1-14 tod	1.57	2	41	3180	mascul	19	22026.87	181.1	82.4	134	70	88	215	56	135	119	0.577	0.5781
11	1 ou -	n„o	1.56	1	41	3900	masculin													
6	3.1-6	n„o	1.6	2		3400	feminir	34												
14	3.1-6	n„o	1.61	1	40	4030	feminino		20423.24	179.65	73.2	106	60.5	121	169	60	90	80	0.5783	0.5819
4	1.1-3	n„o	1.52	2		2710	mascul	12	2743.729			120	67	70	173	32	134	39	0.5782	0.5869
8	3.1-6	15 + tod	1.65	2	37	2700	mascul	39	23769.78	185.55	88.4	126.5	74	82	165	52	99	94		
2	1.1-3	1-14 par	1.58	2	39	3350	mascul	19												
3	1.1-3	n„o	1.65	3	37	2600	feminir	60	42652.3	168.05	90.4	133	87	89	130	38	78	60	0.5771	0.5787
14	1.1-3	1-14 tod	1.58	3	40	2950	mascul	18	22826.53	178.15	85.6	122.5	76.5	103	153	46	79	162		0.5704
7	1.1-3	1-14 tod	1.6	5	40	3340	mascul	59	18716.67	174.3	71	118.5	74	120	216	33	135	200		0.578
0	1 ou -	n„o	1.62	4	41	3000	feminir	60	19495.14	167.7	63.2	109	65.5	114	246	54	180	98	0.578	0.5713
5	1 ou -	1-14 tod	1.55	4	43	3860	feminir	54												
8	1 ou -	1-14 tod	1.45	1	41	2750	masculin		11003.05	173.45	69.2	126	74.5	79	132	50	66	59	0.5658	0.5764
7	1.1-3	1-14 tod	1.55	1	40	2600	feminino													
4	1 ou -	n„o	1.54	1		2560	masculin		18879.05	158.75	70.2	115.5	67.5	84	206	65	130	62	0.5894	
5	1 ou -	1-14 tod	1.64	1	41	3260	masculin		18085.46	180.8	77.9	134.5	73.5	106	210	57	136	90	0.5776	0.5611
3	1.1-3	n„o	1.56	4	39	2480	mascul	51	22165.08	180.3	78.1	121.5	79.5	75	140	62	69	44	0.5771	
4	3.1-6	n„o	1.63	1	40	3560	masculin													
4	1.1-3	n„o	1.46	2	40	3550	mascul	14												
11	3.1-6	n„o	1.52	2	40	2920	mascul	12	36221.38	170.15	99.6	137.5	96	96	280	52	155	389	0.581	0.578

pescmae	prenda	pfumomae	paltmae	pgesta	pidgest	ppn	psex	pint	dmgtotdxa	daltura2012	dpeso	dsysmed	ddiamed	dglicose	dcolesterol	dhdl	dldl	dtrig	dECMICE	dECMICD
0	1 ou -	n„o	1.43	1	33	1760	masculin													
6	1 ou -		1.54	1	39	3350	feminino		25495.36	170.6	68.7	119	84	72	208	82	109	55	0.5793	0.5785
5	3.1-6	15 + tod	1.58	2	37	2000	masculin		22291.95	165.3	72.7	118.5	70.5	78	160	42	87	169	0.5794	0.5765
5	1.1-3	1-14 tod	1.52	3	40	2730	mascul	23	3436.015	175.2	56.4	120	66.5	82	170	60	91	75	0.5779	0.5776
4	1 ou -	n„o	1.54	1	39	3200	feminino		27086.64	155.7	71.8	122.5	71.5	97	229	79	127	90		
9	6.1-10	1-14 par	1.54	3	36	2280	feminir	42	37432.94	155.5	81.3	113.5	82.5	99	148	43	80	163	0.5733	
8	1.1-3	n„o	1.58	1	40	3200	feminino		18571.01	158.45	56.2	118	75.5	82	153	56	81	75		
4	3.1-6	n„o	1.54	2	40	3450	mascul	43	19941.22	177.05	79.4	133	73	89	185	63	106	84	0.6232	0.5773
5	1.1-3	n„o	1.67	1	40	3400	masculin		31561	178.5	87	124	71	82	165	55	85	114	0.5733	0.5927
5	1 ou -	1-14 par	1.6	2		3120	mascul	18												
4	1 ou -	1-14 tod	1.4	3		2580	mascul	38	42345.95	156.75	102.6	129.5	89.5	563	420	25	50	1662		
3	1 ou -	n„o	1.38	2		2980	feminir	23	29177.49	153.7	68.9	117	85	78	195	70	108	112	0.5784	0.5767
6	1.1-3	1-14 tod	1.5	8		4420	mascul	95	35604.88	182.1	107.8	130.5	72.5	137	214	37	96	399		
8	1.1-3	n„o	1.53	3	38	3450	mascul	67	25568.21	173.9	94	143	84	111	220	50	106	343	0.5808	
3	3.1-6	n„o	1.62	9	42	3980	mascul	73	13030.34	173.5	79.1	117.5	66.5	62	231	65	149	69	0.5596	0.6008
11	10	n„o	1.72	3		3600	feminir	19	40228.64	170.3	78	99.5	53.5	69	192	58	118	59	0.5698	0.5759
2	3.1-6	n„o	1.58	2	41	3780	mascul	25	26743.81	171.3	87.6	112	76	87	193	55	122	74	0.5938	0.5726
5	1.1-3	n„o	1.61	1	42	3070	masculin													
6	3.1-6	15 + par	1.49	7		3140	mascul	89												
4	1.1-3		1.53	10	40	3200	mascul	44		173.75	135.4	146	82	163	275	70	151	377	0.5827	0.5943
7	1.1-3	1-14 par	1.52	1	40	2820	masculin		18505.55	165.1	67.1	126.5	65.5	70	167	64	91	57	0.6558	0.5789
5	1 ou -	1-14 tod	1.53	1		2140	masculin													
5	3.1-6	n„o	1.64	2	40	4050	feminir	73												
5	1.1-3	1-14 tod	1.6	3	39	3620	mascul	30												
5	6.1-10	n„o	1.48	1	41	3300	masculin		12177.22	171.3	63.7	124.5	77.5	88	189	63	109	97	0.5781	0.5674
8	1.1-3	n„o	1.61	1	39	2900	masculin		4258.285	170.25	56.5	131	83	97	157	65	80	45	0.5795	0.5818
6	1 ou -	15 + par	1.59	1		2300	feminino													
1	1.1-3	n„o	1.53	1	39	3600	feminino		28030.43	158.2	68.9	105	62.5	93	171	50	100	102	0.5778	
5	6.1-10	n„o	1.53	3		3400	mascul	83	22072.28	162.3	74.9	138	78.5	91	204	51	129	107		
0	1.1-3	1-14 tod	1.62	1		2650	feminino		34111.55	160.45	73.4	113.5	73	77	173	52	103	82	0.593	0.5786
5	1 ou -	n„o	1.51	5	39	3740	feminir	13	15389.92	153.3	50.8	112	68.5	69	180	66	96	98	0.5786	0.5766
3	1.1-3	n„o	1.59	1	38	3700	masculin													
12	1.1-3	n„o	1.67	1	39	3460	feminino		24875.75	163.55	65.7	109.5	70	64	158	60	79	97	0.5773	0.564
7	1.1-3	n„o	1.48	1	39	3400	feminino		57565.89	152.1	99.2	121.5	79	119	250	63	170	102	0.5774	0.581
9	1.1-3	n„o	1.47	1	40	2980	masculin		14642.44	174.65	72.9	123	71.5	80	130	49	73	33	0.5749	0.5748
16	10	n„o	1.59	1	37	2500	feminino													
4	1 ou -	n„o	1.69	2		4500	mascul	15	10780.91	175.95	68.1	127	64	88	150	44	92	69	0.5714	0.5786
5	1.1-3	1-14 par	1.65	2		3840	mascul	15		195.65	107.6	128.5	77	105	134	49	77	46	0.5788	0.5788
3	1.1-3	n„o		6	37	3400	mascul	16	19358.78	164	73.6	127.5	71.5	122	246	73	137	156		
9	3.1-6	n„o	1.5	2	39	3110	feminir	14	19019.48	162.75	59.1	110.5	72	78	159	78	71	53	0.5802	0.5734
0	1 ou -	n„o	1.43	1	40	2770	masculin													
3	1.1-3	15 + tod	1.52	4	41	3150	feminir	15	38692.79	157.2	79.5	147.5	95	106	190	63	105	72	0.578	0.5812
4	1.1-3	15 + tod		3	39	1750	mascul	15												
11	1.1-3	n„o	1.55	3	39	3900	feminir	18	34519.9	168.25	75.9	103.5	63.5	84	219	63	142	70	0.5792	0.5763
0	1.1-3	n„o	1.51	1	39	3600	feminino													
5	1.1-3	n„o	1.62	6		3200	feminir	24	15172.18	164.3	63.5	110	77	75	149	49	90	48		
5	1.1-3	n„o	1.6	2	38	3300	feminir	35	17213.99	159.4	56.8	94.5	59	82	147	47	75	121	0.5777	0.5881
3	1.1-3	n„o	1.51	3		3600	mascul	32	12657.18	177.45	76.4	127	72	98	239	50	150	183	0.5784	0.5779

pescmae	prenda	pfumomae	paltmae	pgesta	pidgest	ppn	psex	pint	dmgtotdxa	daltura2012	dpeso	dsysmed	ddiamed	dglicose	dcolesterol	dhdl	dldl	dtrig	dECMICE	dECMICD
16	3.1-6	15 + tod	1.63	2	36	3150	feminir	54	8608.266	164.1	46.1	101	64	89	199	74	109	63	0.5747	0.562
14	3.1-6	n„o	1.75	3	38	3550	mascul	34	13696.58	185.15	79.4	109.5	63.5	68	133	53	71	39	0.5788	0.5859
5	1.1-3	n„o	1.52	1	41	3660	masculin		15022.4	169.75	63.5	116.5	67.5	85	229	60	149	94	0.5783	0.5751
4	1.1-3	15 + tod	1.56	3	39	3250	mascul	81	29423.74	176.05	100.9	124	80	95	159	51	84	107	0.5798	0.5809
2	3.1-6	1-14 tod	1.55	3	40	3800	feminir	35	24392.01	161.65	64.2	104	65	88	224	76	131	78	0.5806	0.5839
12	10	1-14 tod	1.59	2	38	3400	mascul	19												
3	1.1-3	1-14 tod	1.52	3		3110	feminir	93	33532.23	154.8	71.1	100.5	68.5	72	189	44	127	97		
3	1 ou -	1-14 par	1.58	1	40	2860	masculin													
4	1.1-3	n„o	1.58	1	39	3650	masculin		10715.3	176.6	73	108	63.5	87	158	61	90	68	0.5768	0.5771
4	1.1-3	1-14 tod	1.6	7		3480	feminir	33												
5	3.1-6	1-14 par	1.56	1	39	2650	feminino		39315.87	162.95	89.3	113	71	114	175	45	98	215	0.5885	0.6325
5	1.1-3	15 + par	1.5	2	38	3540	feminir	26	21784.2	161.1	63.8	125	74	85	225	53	148	113	0.5787	0.5763
2	1.1-3	15 + tod	1.53	2	39	3580	mascul	30												
6	1.1-3	1-14 tod	1.55	2	39	2740	feminir	52	35554.59	164.85	84.9	121.5	73.5	99	175	78	75	140	0.5794	0.5804
6	3.1-6	n„o	1.58	1	37	4350	feminino		26098.58	167.5	65	122.5	71.5	144	176	69	91	56	0.5763	0.5803
12	1.1-3	n„o	1.46	2	39	2950	mascul	58	5236.091	167.8	56	118	76	81	196	64	115	79	0.5748	0.5783
4	1 ou -	1-14 tod	1.52	3	38	3580	feminir	28												
				3		3180	masculin		2804.416					89	173	67	95	57		
0	1.1-3	1-14 tod	1.53	1	39	3710	masculin													
0	1 ou -	n„o	1.44	7	40	2500	feminir	16												
12	3.1-6	n„o	1.47	2		3100	mascul	49												
15	10	n„o	1.6	2	41	3050	mascul	44	32278.89	191.5	112.4	145.5	83	86	207	56	130	114	0.6383	0.5897
17	10	n„o	1.52	4	38	4260	feminir	64												
4	1.1-3	15 + tod		2		2900	mascul	50	21172.93	163.75	75	143	95.5	87	248	60	170	101	0.5761	
9	1 ou -	n„o	1.57	1	40	3510	feminino													
3	1.1-3	1-14 tod	1.55	2	40	2750	mascul	30												
3	1.1-3	15 + tod	1.62	4		2920	feminir	24												
20	10	15 + tod	1.54	1		3130	feminino													
12	6.1-10	1-14 tod	1.6	3	39	3620	mascul	15	13858.81	182.75	83.3	111.5	64	85	209	68	119	120	0.5784	0.5778
3	1 ou -	n„o	1.47	1		3400	feminino		22151.87	144.4	56.7	97	62	62	174	52	98	105	0.5671	0.5604
3	1.1-3	n„o	1.62	8	41	3270	mascul	22	41484.34	166.3	104.1	125	80	104	234	55	152	122	0.5829	0.5794
4	1 ou -	1-14 tod	1.62	1		3150	feminino			167.7		111	60.5						0.5813	0.58
12	1.1-3	n„o	1.54	2	39	4000	feminir	17	32554.57	167.45	72.7	110.5	82	90	170	62	92	70		
3	1.1-3	n„o	1.71	3	40	4480	feminir	44												
7	1.1-3	1-14 par	1.67	1		3350	feminino		25705.42	161.6	67	117.5	72	106	230	72	138	129	0.5759	0.5762
12	10	n„o	1.58	2	40	3020	feminir	42	20533.66	159.7	57.8	105.5	68.5	78	160	70	68	128	0.5707	0.5722
16	6.1-10	n„o	1.67	2		3160	feminir	30	23052.45	166.8	65.9	115	71.5	73	191	80	99	65		0.5662
7	1.1-3	1-14 tod	1.55	5	40	2930	mascul	11	8826.795	165.85	60.7	121	75	74	150	57	74	92	0.5815	0.5744
9	10	15 + tod	1.65	3		3170	mascul	113												
8	1.1-3	n„o	1.57	1	41	3850	masculin													
8	1.1-3	1-14 tod	1.59	2	40	2900	feminir	29												
4	1 ou -	n„o	1.5	5	40	3260	feminir	34	15453.71	161.1	54.4	122	72	89	160	36	104	68	0.5765	0.579
9	1.1-3	1-14 tod	1.54	3	38	3530	mascul	43	27252.88	187.2	100.1	132.5	77.5	102	252	49	175	127	0.579	0.5816
1	1.1-3	n„o	1.59	5	39	2900	feminir	11	17352.13	151.7	55	117	72.5	77	165	85	68	46	0.5789	0.5781
6	1.1-3	n„o	1.55	4		1540	mascul	18												
7	1.1-3	1-14 tod	1.53	2	37	2950	feminir	47												
6	1.1-3	1-14 tod	1.66	2	34	2140	mascul	46	30802.03	170.05	101.5	144.5	89.5	93	206	46	117	295	0.5775	0.5781
5	3.1-6	1-14 tod	1.59	2	41	4000	mascul	58	28508.58	174.8	100.6	145.5	92	135	274	35	120	599	0.5816	0.6053

pescmae	prenda	pfumomae	paltmae	pgesta	pidgest	ppn	psex	pint	dmgtotdxa	daltura2012	dpeso	dsysmed	ddiamed	dglicose	dcolesterol	dhdl	dldl	dtrig	dECMICE	dECMICD
1	1 ou -	n„o	1.54	2	40	3220	mascul	42	3432.995	182.7	64	101.5	60	77	156	44	90	61	0.6173	0.5778
5	1.1-3	n„o	1.63	1	36	2210	masculin		12666.47	176.7	67.9	108	69	78	154	61	82	67		0.5655
2	1.1-3	n„o	1.46	1	38	3400	masculin													
9	3.1-6	n„o	1.55	2		2600	feminir	78	16242.24	152.9	56.3	116	73.5	69	152	56	82	57	0.578	0.5486
5	1.1-3	n„o	1.59	3	39	3900	mascul	51	30315.24	178.1	94.9	125.5	73.5	105	179	36	93	277	0.5786	0.5689
6	1.1-3	n„o	1.47	1	37	3270	feminino		23389.72	158.75	62.6	98.5	60	81	197	59	118	79	0.577	0.5791
2	1.1-3	1-14 par	1.55	3	39	3150	mascul	128	33469.53	172.9	92.6	128	72	66	204	62	122	90		0.5933
5	1.1-3	1-14 tod	1.61	2	42	2830	feminir	19	24019.92	161.1	63.7	114.5	71.5	90	154	65	72	70		
9	1.1-3	n„o	1.61	3		3250	feminir	24	18699.36	168.9	57.4	114	79.5	84	184	60	99	178	0.578	0.5782
6	1.1-3	n„o	1.49	1	39	3320	feminino													
4	1.1-3	15 + tod	1.54	2		3150	feminir	70	20522.7	157.7	55.3	108	68.5	72	189	73	107	52	0.5674	0.5747
4	1.1-3	n„o	1.63	1		2920	masculin													
12	1.1-3	n„o	1.62	1	41	3850	feminino		26064.87	170.2	68.6	104	62	82	230	73	138	80	0.5728	0.5738
6	3.1-6	1-14 tod	1.5	10	42	3380	mascul	38												
6	1 ou -	n„o	1.58	3	34	1320	feminir	39	9933.08	168.9	46	109.5	70	68	130	46	66	87	0.5703	0.5772
0	1.1-3	1-14 tod	1.5	2	40	3250	feminir	69	20062.65	155.6	57.3	105.5	66	67	210	99	103	39		
5	1 ou -	15 + tod	1.48	1		1600	feminino		17174.27	158.7	54.6	104	72.5	88	175	39	104	184		0.5751
12	3.1-6	n„o	1.52	2	41	3100	feminir	72												
9	3.1-6	15 + tod	1.49	1	39	3130	masculin		8514.01	166	61.5	119.5	72.5	76	263	57	181	131		0.5795
5	1.1-3	n„o	1.62	1		2200	feminino													
5	1 ou -	1-14 tod	1.54	1	41	3500	masculin													
4	1.1-3	15 + tod	1.59	3	40	3200	mascul	88												
5	1.1-3	n„o	1.52	2	39	3210	mascul	41												
14	10	1-14 tod	1.61	2		3030	feminir	17	25412.71	165.9	64.9	107	73.5	102	161	55	76	145	0.5771	
6	1.1-3	1-14 par	1.61	2	42	3170	feminir	17	29139.68	167.15	68.1	125.5	85	100	150	60	70	81	0.5785	0.5767
7	1.1-3	15 + tod	1.52	2	41	3450	mascul	12	16392.32	173	75.8	129.5	73.5	90	196	63	118	82	0.5827	0.6075
4	1 ou -	n„o	1.59	4	39	2400	mascul	11												
16	3.1-6	n„o	1.62	1	38	3670	feminino													
6	1.1-3	n„o	1.58	3	39	3800	feminir	45	35819.09	169.65	82.9	104.5	72.5	78	132	40	72	87		0.5775
7	1.1-3	n„o	1.6	2	39	3010	feminino													
5	3.1-6	n„o	1.62	3	36	3720	feminir	135	27145.26	165	65.9	115.5	79.5	96	248	51	170	207	0.5812	0.5694
7	3.1-6	n„o	1.56	1	35	2300	feminino													
5	1.1-3	n„o	1.55	3	38	3470	mascul	56												
9	3.1-6	1-14 par	1.6	4	38	3070	feminir	75												
5	3.1-6	n„o	1.54	4	38	3430	feminir	26	31338.93	162.9	66.5	128	76.5	86	164	63	91	55	0.5731	0.5782
6	1 ou -	n„o	1.54	1	40	3100	masculin		18447.12	179.95	75.1	114	73	94	200	50	127	104	0.6072	0.5888
2	1.1-3	n„o	1.59	5		3050	mascul	35				96	55.5	91	168	49	102	85	0.5752	0.5764
5	1.1-3	15 + tod	1.56	8		2100	mascul	11												
5	1 ou -	1-14 tod	1.64	1	40	3170	masculin													
8	3.1-6	n„o	1.54	2	37	3400	mascul	30	27437.52	164.6	82.3	132.5	91	92	225	46	147	153	0.6327	0.5749
0	1 ou -	1-14 tod	1.5	2	34	1550	masculin													
4	1 ou -	15 + tod	1.65	3		2410	masculin													
3	1.1-3	n„o	1.55	2	40	3400	mascul	72	17274.01	167.9	72.6	105	73	85	149	43	82	126	0.5759	0.5773
7	1.1-3	1-14 par	1.63	1	40	3130	masculin		23543.7	168.8	84.2	120	64	88	167	41	96	164	0.5982	0.5766
13	3.1-6	n„o	1.49	6	39	3930	mascul	25	13704.91	169.3	66.6	112.5	66	86	142	55	65	145		
6	1 ou -	1-14 tod	1.62	1	33	2340	masculin													
6	1 ou -	n„o	1.59	2	39	3100	mascul	18	8305.827	172.7	69	120	67	95	156	55	83	77	0.5714	0.573
7	1.1-3	1-14 tod	1.48	3	41	2980	feminir	51	44426.11	151.55	89.8	131	90	82	230	57	139	241		0.5794

pescmae	prenda	pfumomae	paltmae	pgesta	pidgest	ppn	psex	pint	dmgtotdxa	daltura2012	dpeso	dsysmed	ddiamed	dglicose	dcolesterol	dhdl	dldl	dtrig	dECMICE	dECMICD
6	10	n„o	1.52	1	39	3720	feminino		15197.89	157.9	48.6	107.5	75	83	208	68	124	59	0.5828	0.5781
2	1 ou -	15 + tod	1.58	1		2600	masculin		36869.32	170.85	106.7	137.5	78.5	238	175	48	102	119		
8	1 ou -	1-14 tod	1.64	7		2450	masculin		3339.271	172.4	59.7	114	71	68	164	63	88	57	0.5804	0.5784
3	1 ou -	1-14 par	1.48	2		3020	feminino													
4	1.1-3	n„o	1.5	1		2700	masculin													
7	1.1-3	n„o	1.51	3	39	3250	mascul	17	29016.65	174.75	84.5	126.5	76.5	79	203	57	123	105		
0	1 ou -	n„o	1.51	9		2960	mascul	17												
8	1 ou -	1-14 par	1.59	1	41	4050	masculin		29258.66	175.7	91.4	118.5	70.5	113	191	50	121	91	0.578	0.5748
7	1 ou -	n„o	1.52	3	40	3800	mascul	28												
4	1.1-3	n„o	1.52	1	37	3000	masculin		29993.16	175.95	91.8	131.5	88	83	228	45	153	124	0.6176	
4	1.1-3	n„o	1.56	3		1570	feminir	10	18755.03	152.3	57.4	113.5	84	113	223	70	137	83		
8	6.1-10	n„o	1.6	4	39	3900	feminir	45	22749.5	169.3	66.4	110	60.5	79	190	59	111	81	0.5761	0.5741
12	3.1-6	n„o	1.51	4	39	3570	mascul	69												
9	1.1-3	1-14 tod	1.55	4	38	2560	feminir	15	15931.36	153.55	51.7	94.5	61	69	273	51	195	134	0.5785	0.5551
5	1.1-3	n„o	1.61	3	37	3300	mascul	36												
4	1.1-3	n„o	1.55	5	38	3710	feminir	17	26537.25	165.45	68.5	123.5	79	97	250	75	145	161	0.5897	0.5787
3	1 ou -	1-14 par	1.52	5	40	3800	feminir	142	40034.93	164.3	87.2	127.5	87.5	126	222	76	125	126	0.5768	0.5786
5	3.1-6	n„o	1.53	3	39	3580	mascul	98	25205.09	173.9	81.9	141	97.5	101	215	82	108	101	0.5721	
5	1.1-3	n„o	1.53	1	40	3070	feminino		27779.13	147.1	61.6	122.5	81.5	84	178	70	99	71	0.5791	0.5769
6	1.1-3	1-14 tod	1.64	3		3220	mascul	15	19232.03	169.6	70.5	111	68	87	170	66	89	72	0.5815	0.5792
0	1 ou -	1-14 tod	1.55	1	36	3180	feminino													
5	1.1-3	n„o	1.48	3	35	2720	feminir	52												
12	6.1-10	n„o	1.55	3	38	3450	mascul	76	19156.26	171.35	77.9	134	79	91	293	52	212	83	0.5786	0.5789
2	1 ou -	n„o	1.51	8		2900	feminir	15	27748.09	154.5	61.4	109	67	88	169	57	100	52	0.5692	0.554
2	1.1-3	1-14 tod	1.58	8		3000	feminir	150	56971.19	166.25	111.5	120	74	90	155	43	94	93	0.5922	
5	1.1-3	n„o	1.53	2	40	2800	feminir	51												
9	1.1-3	1-14 par	1.49	1	42	3240	masculin													
5	1 ou -	n„o	1.42	1	39	3110	masculin		10186.05	170.8	69	106	57	79	182	63	107	106	0.5776	0.578
4	1 ou -	1-14 tod	1.5	2	39	2650	mascul	39	7138.944	167.8	65.7	148.5	96.5	83	189	53	120	76	0.5815	0.5828
11	3.1-6	n„o	1.54	3	40	2930	feminir	44	32970.05	161.5	76.4	126.5	89.5	81	249	64	164	132	0.5789	0.5738
5	1.1-3	n„o	1.56	3	40	3210	feminir	29	20902.47	170.1	63.1	111.5	73	78	255	38	182	210		
9	1.1-3	1-14 tod	1.5	1	42	3120	feminino			158.85		105.5	62.5						0.5674	0.5486
7	1.1-3	1-14 tod	1.57	4	37	3680	feminir	30												
0	1 ou -	n„o	1.58	2		2760	feminir	59	56634.01	169.65	112.8	138	100.5	80	200	68	122	63	0.5834	
6	1.1-3	n„o	1.6	1	42	3450	feminino		47735.3	162.9	93.2	124	80.5	84	169	65	86	63		0.5729
3	1 ou -	n„o	1.52	2	38	3500	feminir	29	30780.59	169.95	77.9	126.5	81.5	84	152	55	89	50	0.5848	0.5783
3	1 ou -	15 + tod	1.6	5	41	3770	feminino		23820.58	163.1	67	104.5	67	113	225	71	139	64	0.57	0.5775
7	1.1-3	1-14 par	1.56	1		2800	masculin													
12	1.1-3	15 + tod		2	39	3090	mascul	36												
7	1 ou -	15 + tod	1.53	2		2200	mascul	28												
1	1 ou -	n„o	1.43	12	38	3050	mascul	47	10507.87	162.45	58.3	135.5	75	95	155	55	75	102	0.5779	0.5781
2	1.1-3	n„o	1.48	3		3000	feminir	15												
0	1.1-3	n„o	1.59	1	40	3630	feminino													
0	1.1-3	1-14 par	1.55	3	39	3600	mascul	150	23144.22	172.6	82.7	138	83.5	102	198	86	101	61		
3	1 ou -	15 + tod	1.58	3	30	1730	mascul	13	32932.8	167.3	88.7	124	80.5	103	183	42	108	139	0.5723	0.5803
16	6.1-10	n„o	1.6	1	40	3300	masculin													
7	1.1-3	n„o	1.59	1	41	3060	feminino		36595.43	162.4	76	112	69	86	256	45	202	58	0.5886	0.7136
4	1.1-3	n„o	1.54	1		2700	feminino													

pescmae	prenda	pfumomae	paltmae	pgesta	pidgest	ppn	psex	pint	dmgtotdxa	daltura2012	dpeso	dsysmed	ddiamed	dglicose	dcolesterol	dhdl	dldl	dtrig	dECMICE	dECMICD
12	3.1-6	n„o		1	38	2470	masculin													
6	1.1-3	1-14 par	1.71	4		2860	mascul	25	15270.72	171.25	78.4	126	81.5	357	175	41	117	88	0.5791	0.6278
5	1.1-3	n„o	1.6	1		2770	masculin													
6	1.1-3	1-14 par	1.53	1	40	2680	feminino		28030.85	148.7	61.3	124	75	77	225	49	150	129	0.5791	0.5885
9	1.1-3	1-14 par	1.67	3	37	2780	mascul	48												
3	1.1-3	n„o	1.6	1	37	3550	feminino		26824.07	164.6	70	108	71	70	244	95	122	110	0.5787	0.5785
5	1 ou -	1-14 tod	1.69	2		2230	feminir	21	22766.29	162.35	63.3	100	59.5	71	148	53	78	75	0.5794	0.5793
5	3.1-6	1-14 tod	1.5	5	41	2730	masculin													
12	6.1-10	n„o	1.53	1	42	3050	feminino		8440.664	160.7	45.9	102	68.5	65	172	81	70	74		
3	1 ou -	n„o	1.65	11	40	3680	mascul	45	30361.21	173.25	98.5	121	68	72	209	45	117	264	0.5802	0.6394
1	3.1-6	n„o	1.6	1	39	3550	masculin		44153.75	178.6	113.9	148	84.5	131	223	43	134	233	0.6284	0.5892
6	6.1-10	n„o	1.56	2	38	3440	feminir	65												
1	1 ou -	n„o	1.56	4		2790	mascul	13												
3	1 ou -	15 + tod	1.55	1	38	2700	feminino		15527.41	161.85	61.4	108	72.5	76	175	56	112	61	0.5786	0.578
7	1.1-3	n„o	1.66	2		3300	feminir	15												
5	1.1-3	15 + tod	1.52	4	41	3200	mascul	129	18814.9	165.45	67.5	129	79	79	218	51	86	492	0.579	0.57
5	1.1-3	n„o	1.6	3	38	3330	mascul	61	30195.75	168.45	95.9	124	82	78	174	66	93	85	0.5897	0.5998
4	1.1-3	1-14 par	1.53	3	39	3700	feminir	63												
15	1.1-3	n„o	1.48	1	40	3800	masculin													
4	1.1-3	n„o	1.56	1		2700	masculin		32430.29	173.1	90.2	135	76	78	195	46	124	189	0.5795	0.5856
1	1.1-3	n„o	1.52	4	40	4200	feminir	142	13696.51	155.1	53.4	110	58.5	97	176	57	106	94	0.578	0.5784
16	6.1-10	n„o	1.63	1	41	3700	masculin													
2	1.1-3	n„o	1.52	1	39	2880	feminino													
6	1.1-3	15 + tod	1.56	4	32	1900	feminir	150	31749.36	165.7	74.9	118	71	82	239	49	173	81		
11	10	n„o	1.62	2	41	3580	mascul	35												
12	10	n„o		1	35	2620	masculin													
1	1.1-3	n„o	1.59	2	38	3700	mascul	43	15501.18	174.55	84.9	152	86	107	143.23	46.88	82	71.205	0.5816	0.587
4	1.1-3	15 + tod	1.51	3	27	770	feminir	43												
5	1.1-3	1-14 tod	1.57	1	39	3020	masculin		15686.02	180.35	78.5	120	68.5	68	155	49	84	113	0.578	
5	1.1-3	n„o	1.55	1	39	3000	masculin													
11	1.1-3	n„o	1.62	1	38	3560	masculin		9090.939	185.55	66.5	133	68	91	218	49	143	120	0.5719	0.5685
11	3.1-6	n„o	1.61	2	38	2850	mascul	14	27190.35	176.45	85.7	135	81	86	165	60	86	94	0.5791	0.5789
4	1.1-3	1-14 tod	1.57	1	40	3750	masculin			180.75	129.4	145	84.5	257	235	46	131	320		
4	1.1-3	15 + tod	1.6	4	42	3100	feminir	40												
8	3.1-6	1-14 par	1.55	1	38	2850	feminino			163.95									0.5775	
7	1.1-3	n„o	1.6	1	42	3040	masculin		28168.41	171.8	101.1	127.5	76	87	203	46	112	266	0.5794	0.5826
3	1 ou -	1-14 par	1.61	1	42	3300	masculin		6269.503	183	73.5	120	66	83	160	35	118	45	0.5685	0.6013
12	3.1-6	15 + tod	1.63	1	39	3180	masculin													
4	3.1-6	n„o	1.64	6	40	3370	feminir	141												
3	3.1-6	n„o	1.53	2	39	3000	mascul	127	38767.59	171.5	102.6	136	77	95	174	51	100	127	0.5695	
5	3.1-6	15 + tod	1.56	7	38	1840	feminir	53	8781.881	156.2	46.6	121.5	81	56	163	58	93	52	0.5531	0.5777
7	3.1-6	n„o	1.58	3	42	3440	mascul	77		176.8	133.2	132	87.5	92	149	43	81	134	0.5944	0.5834
8	1.1-3	n„o	1.63	1	43	2700	masculin		26426.84	179.6	92.4	123.5	69	80	129	44	65	92	0.5803	0.6153
6	1 ou -	n„o	1.52	1	41	3280	masculin													
7	3.1-6	n„o	1.61	1	40	3150	masculin		24768.6	177	79.9	129	78.5	147	237	41	110	505	0.5794	0.5743
5	1.1-3	n„o	1.51	1	42	3350	masculin		27230.58	170.55	86.6	116.5	72.5	83	232	48	158	239	0.5781	0.5799
5	6.1-10	1-14 tod	1.58	7	39	3140	feminir	67	23518.96	157.9	62.6	108.5	69.5	81	182	67	107	53	0.5928	0.5967
15	10	n„o	1.72	1	38	3320	feminino		45253	169.5	92.3	131.5	88.5	117	204	69	118	70	0.5786	0.5782

pescmae	prenda	pfumomae	paltmae	pgesta	pidgest	ppn	psex	pint	dmgtotdxa	daltura2012	dpeso	dsysmed	ddiamed	dglicose	dcolesterol	dhdl	dldl	dtrig	dECMICE	dECMICD
17	6.1-10	n„o	1.47	2	40	3350	feminir	15	20123.27	153.65	55.7	96.5	60.5	84	135	61	60	79	0.5356	0.5684
5	1.1-3	n„o	1.5	3	42	3500	feminir	31												
5	3.1-6	15 + tod	1.51	1		3000	masculin		19922.3	164.7	73.9	137	89.5	94	160	50	95	77	0.5898	
7	1.1-3	n„o	1.53	3	41	2950	mascul	51												
15	6.1-10	n„o	1.45	2	39	3820	mascul	71	43669.21	177.7	108	135.5	82	92	245	58	160	121		
5	3.1-6	n„o	1.65	1	38	4020	masculin		11222.01	181.5	70.4	131	70	80	135	56	67	50	0.5781	0.5858
14	10	n„o	1.55	5	40	3900	mascul	36												
8	3.1-6	15 + tod	1.52	1	36	2480	masculin													
8	1.1-3	1-14 tod	1.56	1	39	3480	masculin													
5	1.1-3	n„o	1.51	4	41	2540	mascul	18	14275.59	167.4	78.1	134.5	79	119	185	49	90	240	0.581	0.5777
5	1.1-3	1-14 tod	1.58	1	42	3340	masculin													
6	1.1-3	15 + tod	1.57	4		2920	feminir	14												
17	6.1-10	n„o	1.59	5	39	2880	mascul	17	16545.84	173.4	74.7	123.5	69	84	201	79	108	78	0.5762	0.5958
4	1 ou -	n„o	1.52	4	37	3200	mascul	15												
3	1.1-3	15 + tod	1.46	1		3020	feminino		25670.23	162	66.7	124.5	84	80	280	70	153	383	0.5608	0.5754
5	1.1-3	n„o	1.57	3	41	2700	mascul	11	33470.21	177.15	83.7	123	82	92	179	45	122	78	0.5812	0.5791
3	6.1-10	n„o	1.5	3	38	3680	mascul	20	21678.5	173.9	75.8	122	68	55	153	41	91	85	0.5788	0.5782
10	3.1-6	n„o	1.48	1	39	3870	feminino													
11	3.1-6	n„o	1.57	1		3420	masculin		14614.97	173.6	69	114	71	83	171	53	94	115		
5	1.1-3	1-14 tod	1.58	1	39	2600	masculin		27791.01	155.45	82.9	141.5	68.5	102	258	60	149	288	0.5797	
18	3.1-6	n„o	1.63	1	38	3500	masculin													
8	6.1-10	n„o	1.57	2		3200	feminir	29	15361.04	166.15	53	110	68.5	88	221	78	119	123	0.5847	0.5773
16	6.1-10	n„o	1.61	3	39	3000	mascul	21												
9	3.1-6	n„o	1.56	1		2750	masculin													
5	1 ou -	15 + tod	1.55	4		3550	mascul	11	5193.451	178.4	58.6	104.5	70	76	125	42	70	56	0.5764	0.5745
2	1.1-3	n„o	1.61	3	40	3780	feminir	36	42968.45	168.5	93.1	131	81	119	228	76	117	187	0.5808	0.6518
4	1 ou -	n„o	1.68	1	42	4350	masculin		24186.92	187	94.7	122.5	68.5	116	192	57	93	195	0.5671	0.5816
0	1.1-3	1-14 tod	1.64	7		2900	feminir	24	43909.46	156.05	87.6	113	74.5	69	157	49	96	78	0.5784	0.5819
3	1.1-3	1-14 tod	1.36	3	34	1910	mascul	53												
7	1.1-3	n„o	1.6	4	39	3780	mascul	69	23752.96	177.7	86.4	158.5	103	102	187	46	91	284	0.5998	0.5838
4	1 ou -	n„o	1.56	7	40	3680	feminir	40	12186.89	161.35	50.7	119	79.5	98	178	61	105	99		
5	1 ou -	1-14 tod	1.5	1		2330	feminino		31592.56	156.1	70.9	112.5	75.5	84	197	47	120	174		
2	1.1-3	n„o	1.53	5	41	2960	mascul	15	20780.23	175.05	74.4	122	68	72	258	73	160	125	0.5781	0.5308
12	1 ou -	n„o	1.54	2		2980	feminir	21												
5	3.1-6	15 + tod	1.59	2	36	3600	mascul	42	20507.13	175.1	84.4	121	71.5	90	225	47	138	197	0.5774	0.5555
6	1.1-3	n„o	1.51	1	39	3400	feminino		30360.58	161.3	72.4	122	74.5	85	233	57	155	142	0.5777	0.5787
5	1.1-3	1-14 tod	1.54	8	41	3050	feminir	100	35730.3	157.55	74.9	116	75.5	71	238	52	166	114	0.5758	
11	1.1-3	n„o		2	37	3600	mascul	29	12189.02	168.6	70.2	102	55	84	123	46	61	64	0.5792	0.5777
4	1.1-3	n„o	1.61	7	38	3100	feminir	112	64245.44	166.85	117.3	153	108.5	108	244	63	155	152	0.6394	0.5861
4	3.1-6	n„o	1.65	2	40	3450	masculin													
8	3.1-6	1-14 tod	1.47	1	40	3060	feminino		37213.35	149.1	76.2	116	77.5	79	235	65	152	67	0.5813	0.5836
8	1.1-3	n„o	1.59	3	38	2660	feminir	25	46942.82	168.75	99.2	126	75	70	157	42	90	148		
4	1.1-3	15 + tod	1.59	13		3200	feminir	13												
4	1.1-3	n„o	1.47	6	39	2680	feminir	23	41603.24	152.55	73.1	99	74	82	200	44	143	117	0.563	0.5694
6	3.1-6	1-14 tod	1.5	1	35	2550	masculin													
3	1.1-3	1-14 tod	1.58	4	41	2730	feminir	72												
1	1 ou -	n„o	1.5	2	39	3200	mascul	24	4572.207	177.45	61.5	127.5	73.5	95	177	68	92	85	0.578	0.5633
11	10	n„o	1.6	1	38	4350	feminino													

pescmae	prenda	pfumomae	paltmae	pgesta	pidgest	ppn	psex	pint	dmgtotdxa	daltura2012	dpeso	dsysmed	ddiamed	dglicose	dcolesterol	dhdl	dldl	dtrig	dECMICE	dECMICD
0	1.1-3	n„o	1.54	1	39	3240	feminino													
9	3.1-6	n„o	1.61	3	40	2970	feminir	28												
9	1.1-3	n„o	1.54	1	40	2950	feminino		34107.93	160.05	69.6	103.5	70.5	77	172	65	94	67		
8	3.1-6	n„o	1.55	2		2300	mascul	31	8379.41	171.85	56.3	122.5	80						0.5505	0.5724
7	3.1-6	n„o	1.5	1	38	3050	feminino													
5	1.1-3	n„o	1.57	4		1530	mascul	28	8416.423	170.75	66.5	133.5	83	125	160	54	81	126	0.5879	0.5777
0	1 ou -	n„o	1.57	3	42	3350	mascul	22	21945.8	165	72.1	103	55.5	77	125	43	69	70	0.5784	0.5797
2	1.1-3	n„o	1.51	1	42	3550	feminino													
4	1.1-3	1-14 par	1.48	4	40	3070	feminir	17	16700.21	150.25	52.9	129.5	80	89	183	50	120	76	0.5764	0.5665
0	1 ou -	n„o	1.55	5		3150	mascul	59	14351.83	169.5	76.8	131	72	87	223	59	120	224	0.579	0.5791
8	1.1-3	n„o	1.45	2	40	3350	feminir	48	34455.83	167.8	79.3	117	77	77	182	75	90	69	0.6143	0.5799
7	1 ou -	1-14 par	1.47	9	39	2610	feminir	41												
0	1 ou -	n„o	1.6	16	40	3600	feminir	22	44519.42	157.8	94.9	118.5	75	108	173	61	98	80	0.593	0.5781
6	3.1-6	n„o	1.57	7	38	3230	mascul	30	21987.86	174.85	83.5	123.5	71.5	105	216	52	124	153	0.6785	0.6325
15	1.1-3	n„o	1.52	2	37	3100	mascul	53	21386.77	170	77.5	150.5	106.5	83	139	40	86	72	0.5831	
9	1 ou -	n„o		1		2700	feminino		16150.37	157.3	49.6	145	89	83	216	77	115	116	0.5756	0.5652
0	1 ou -	n„o	1.51	6	40	3300	mascul	14												
1	1 ou -	n„o	1.55	2	39	4000	mascul	72	11754.64	171.65	69.5	116	73.5	89	239	62	160	44	0.5905	0.5716
14	6.1-10	n„o	1.63	1	41	3200	feminino													
9	1.1-3	n„o	1.6	3	37	3050	mascul	53												
2	1 ou -	1-14 tod	1.55	2		3540	feminir	52	31831.9	163.6	79.2	123	83.5	93	249	43	159	236	0.5784	0.5788
6	3.1-6	n„o	1.6	7	40	3800	mascul	15	36033.71	166.8	87.2	122.5	79	89	174	41	107	121	0.5815	
7	1.1-3	n„o	1.48	1	40	3000	feminino													
4	1.1-3	1-14 tod	1.58	1	40	3050	feminino													
7	1 ou -	1-14 tod	1.54	2	40	3400	feminir	31												
5	1 ou -	1-14 par	1.58	1		3720	feminino		30316.96	182	90.8	130.5	86.5	94	176	41	108	202	0.6106	0.5784
5	1.1-3	n„o	1.5	1	39	3820	masculin													
16	10	n„o	1.56	2	36	2850	feminir	42												
6	1.1-3	n„o	1.63	1	37	3400	masculin		12412.04	187.45	80.1	112	64.5	375	160	34	68	201	0.58	0.5804
20	6.1-10	n„o	1.61	3	38	3140	mascul	29	30989.49	182.15	88.5	140	83.5	98	207	62	107	178	0.5837	0.5833
18	10	n„o	1.63	1	37	3000	masculin		3998.09	184	66.3	124	69	88	134	39	55	154	0.5783	0.5787
4	1.1-3	n„o	1.42	4	38	2600	masculin		12578.09	164.7	63	148	93	81	240	50	151	223		0.5625
9	10	n„o	1.59	2	36	3150	mascul	75												
6	1 ou -	n„o	1.54	4	36	2500	mascul	34	10193.42	175.2	69.8	123.5	80	91	230	89	122	70	0.5786	0.5781
2	1.1-3	n„o	1.56	2	33	1310	feminir	19	27976.11	167.95	69.7	137	76	82	179	61	108	63	0.5777	0.5763
10	1.1-3	1-14 par	1.48	1	40	3590	feminino													
5	1.1-3	n„o	1.62	3	38	4500	feminir	72	58644.86	165.8	113.4	112	74	78	202	50	132	122	0.6298	0.5809
16	3.1-6	n„o	1.81	1	39	3100	masculin													
7	1.1-3	n„o	1.63	2	38	2990	mascul	65												
5	1.1-3	1-14 tod	1.56	1	40	2780	masculin													
12	3.1-6	n„o	1.49	1	41	2100	feminino													
4	1.1-3	n„o	1.6	2		3200	mascul	42	6628.433	176.8	62.9	141.5	78.5	76	176	60	106	42	0.5361	0.578
6	1.1-3	n„o	1.53	1	39	2700	masculin		15980.47	171.1	74.2	121	73.5	97	190	43	115	190		
7	6.1-10	1-14 tod	1.48	1	41	3580	feminino		23970.3	156.2	61	108	69.5	97	220	74	127	81	0.5738	0.5688
5	1 ou -	n„o	1.5	1	39	3300	feminino		10403.97	152.2	45.6	99	67	84	206	62	126	98	0.5767	0.5733
6	1.1-3	1-14 tod	1.53	2	41	3510	feminir	12	25663.53	162.95	68.3	112.5	67.5	79	264	59	177	119	0.5789	0.5794
5	1.1-3	n„o	1.49	2	41	3600	feminir	15	30357.41	160.5	68.8	106	67.5	86	210	59	130	80	0.5668	0.5734
5	1 ou -	1-14 tod	1.5	2	39	3360	mascul	10	11137.5	163.25	69.4	119.5	73	144	214	65	130	104	0.5782	0.5825

pescmae	prenda	pfumomae	paltmae	pgesta	pidgest	ppn	psex	pint	dmgtotdxa	daltura2012	dpeso	dsysmed	ddiamed	dglicose	dcolesterol	dhdl	dldl	dtrig	dECMICE	dECMICD
3	1 ou -	1-14 tod	1.6	2	41	2750	feminir	33	33920.59	160.05	76.5	119	79.5	78	275	68	172	183	0.5834	
5	6.1-10	n„o	1.56	3		3800	feminir	20	14002.72	162.2	52.1	101.5	64.5	88	176	54	111	69	0.5779	0.5715
5	3.1-6	n„o	1.59	2	39	3290	mascul	18	44006.68	177.1	109.4	139	91	84	173	52	106	81	0.5805	0.5817
0	1 ou -	n„o	1.5	5		3650	feminir	34	22927.47	154.7	61.2	102	63.5	86	143	50	80	52		
5	1.1-3	n„o	1.51	3	38	3660	feminir	76	34331.66	167.1	74	98	62	78	229	83	136	43	0.5875	0.5683
6	6.1-10	n„o	1.47	1	36	2680	feminino		33985.93	156.85	72.9	115.5	74	80	212	63	131	117	0.5638	
11	3.1-6	n„o	1.62	1	38	2850	feminino													
9	1.1-3	n„o	1.72	1	38	4450	feminino		43715.44	169.4	94.8	120.5	88	93	232	77	136	121		0.5804
5	3.1-6		1.59	4	41	4050	feminir	73	9423.655	163.8	46.2	101.5	66	88	152	55	87	73	0.5765	0.5756
14	6.1-10	n„o	1.64	1	39	3400	masculin													
4	1.1-3	n„o	1.54	7	37	3000	feminir	60	17117.34	159.1	53	124.5	79.5	74	178	64	95	86	0.56	0.553
12	3.1-6	1-14 tod	1.51	2	40	2880	feminino			160.6	60.6	117.5	74.5	88	193	64	113	91	0.5684	0.5635
9	1.1-3	n„o	1.51	1		2020	feminino													
6	6.1-10	n„o	1.52	4	38	3640	mascul	54												
0	1.1-3	n„o	1.59	1		3000	masculin													
5	3.1-6	15 + tod	1.54	8		2590	mascul	22		176.35	139.9	184	111	89	199	83	95	82	0.5886	0.5913
7	1.1-3	1-14 tod	1.53	3	42	3340	feminir	20	24559.77	161.7	64.9	96	57.5	83	169	63	86	57		
1	1 ou -	15 + tod	1.52	2	39	3120	mascul	29												
13	10	1-14 tod	1.59	1	40	4170	feminino													
4	1 ou -	1-14 par	1.5	4	41	2870	feminir	15												
3	1.1-3	n„o	1.53	10	39	3000	feminir	15	15020.18	156.3	53.9	115.5	77.5	85	212	63	129	72	0.5782	0.5779
1	1.1-3	1-14 tod	1.45	5	39	3150	feminir	13												
5	1.1-3	n„o	1.62	8	39	3480	masculin		22248.83	179.15	79.2	125.5	82.5	90	231	46	149	195	0.5781	0.5788
0	1 ou -	1-14 par	1.41	1	42	3500	masculin		7634.533	166.5	58	111	68	124	140	54	69	64	0.5665	0.578
7	1.1-3	1-14 tod	1.61	1	40	3950	feminino		59887.41	170.75	116	113.5	73.5	82	158	46	99	61		
4	1.1-3	n„o	1.53	1		2280	feminino		27984.86	152.7	67.2	116.5	73.5	86	205	61	104	233	0.6439	0.5984
10	1.1-3	n„o	1.54	2	41	3470	mascul	43												
12	3.1-6	1-14 tod	1.63	1	35	2600	masculin													
3	1.1-3	n„o	1.65	2	40	3500	mascul	91	21418.47	181.1	83.1	131	89.5	77	179	37	125	95	0.5677	0.5798
5	1 ou -	n„o	1.46	2	39	3030	feminir	38												
8	1.1-3	1-14 tod	1.54	2	39	2950	mascul	36	18035.22	175.4	76.9	110.5	71.5	176	175	59	106	44	0.5776	0.5779
4	1 ou -	n„o	1.63	6	36	2050	mascul	50	27620.9	174.9	88.8	134.5	89.5	78	186	46	122	111	0.5769	0.5931
5	1 ou -	1-14 par	1.61	3	42	3300	feminir	42	45952.68	168	100.1	176	119.5	75	230	77	125	175	0.583	0.5737
0	1 ou -	1-14 tod	1.5	6		2910	mascul	45												
6	1.1-3	1-14 tod	1.62	1	40	3800	masculin		33414.24	171.15	96	145	85.5	105	182	44	117	143	0.5772	0.576
7	1.1-3	n„o	1.56	1	42	2720	feminino		22413.71	143.25	51.1	113	67.5							
2	1 ou -	n„o	1.59	2		3480	feminir	66												
7	1.1-3	1-14 tod	1.54	2	37	2420	mascul	10												
4	1 ou -	n„o	1.51	3		3250	feminir	49												
9	1 ou -	15 + tod	1.53	2		1900	mascul	82												
9	1.1-3	n„o	1.52	1	39	2950	masculin													
7	1.1-3	1-14 tod	1.57	1	41	3520	feminino		45279.37	164	89.2	114	82	84	222	60	150	89	0.5813	0.5793
6	1.1-3	15 + tod	1.56	2	40	2870	feminir	12	29673.73	159.35	71.3	118.5	75.5	88	223	63	144	94	0.5704	0.5788
2	1.1-3	n„o	1.51	6	41	3650	feminir	15	13617.77	157.75	56.4	114.5	74	69	226	61	155	84	0.5836	0.5802
5	1 ou -	n„o	1.44	2		1730	feminir	193												
14	1.1-3	n„o	1.61	1	40	3370	masculin													
3	1.1-3	n„o	1.7	6	39	3870	mascul	69												
2	1.1-3	n„o	1.49	1	38	3050	masculin		9289.23	179.9	74.3	140.5	70.5	95	125	44	72	47	0.573	0.5782

pescmae	prenda	pfumomae	paltmae	pgesta	pidgest	ppn	psex	pint	dmgtotdxa	daltura2012	dpeso	dsysmed	ddiamed	dglicose	dcolesterol	dhdl	dldl	dtrig	dECMICE	dECMICD
2	1.1-3	15 + tod	1.66	1	39	3330	masculin		18989.93	182.35	81.6	139	73	90	213	64	135	45	0.5783	0.5808
3	1.1-3	n„o	1.65	3	39	3200	mascul	28	19902.23	172	74.1	112.5	75	93	178	56	103	99	0.5866	0.5784
8	3.1-6	n„o	1.57	1	39	3530	feminino		43825.75	167.65	89.2	115	78	77	200	97	85	67	0.5781	0.578
5	3.1-6	1-14 tod	1.53	2	38	2900	feminir	25		159.6										0.5532
1	1.1-3	n„o	1.46	1	33	1990	masculin		7521.471	177	63.7	126.5	79.5	78	145	74	63	35	0.5796	0.5848
0	1 ou -	1-14 par	1.52	2	42	1800	mascul	12												
4	1.1-3	n„o	1.51	1	39	3900	masculin		20879.02	179.8	82.1	116	68.5	109	192	48	87	250	0.594	0.5844
9	1 ou -	15 + tod	1.59	9		4010	mascul	40	24299.33	178.7	90.8	124	76	86	184	41	122	103	0.6744	0.6573
7	1 ou -	15 + tod	1.46	1	40	3100	feminino			160.25		116	61						0.578	0.5697
11	3.1-6	1-14 par	1.6	2	39	3610	mascul	12	34622.68	184.05	98.8	139.5	93.5	104	246	47	150	287	0.5958	0.5754
5	1.1-3	n„o	1.53	2	37	3170	feminir	66												
5	1.1-3	1-14 par	1.76	3	39	3500	feminir	18	48621.8	166.75	98.8	106.5	70.5	83	188	62	105	94	0.5895	0.5907
3	1.1-3	1-14 tod	1.57	7		3650	feminir	12	20813.46	168.3	63.8	103.5	61.5	81	158	75	68	59	0.58	0.5605
4	1 ou -	15 + tod	1.51	1	40	2780	feminino		40209.28	152	84.5	133.5	87.5						0.5826	0.5729
8	3.1-6	1-14 tod	1.5	10	38	3330	mascul	51	17900.25	166	71	108	64.5	88	194	52	109	173	0.5671	0.5724
15	6.1-10	n„o	1.6	1	39	3300	masculin		22254.72	189.75	82.2	137	72.5	91	197	61	117	78	0.5676	0.5704
3	1.1-3	1-14 tod	1.56	3	40	2530	feminir	12	30024.88	154.9	71.2	117.5	63	77	179	65	100	75		
6	1.1-3	n„o	1.47	2	43	3460	feminir	26	23273.87	158.2	65.9	126	84.5	100	165	60	95	97		
7	1 ou -	n„o	1.47	1	36	3000	masculin		7097.057	171.8	57.7	112	68	88	219	64	140	76	0.5737	0.578
8	1.1-3	n„o	1.62	3	38	3560	mascul	53												
8	10	1-14 par		2	41	2700	feminir	78												
4	1.1-3	n„o	1.57	3	39	3180	mascul	17	25920.54	186.4	101.9	143.5	66.5	81	144	51	80	56	0.5794	
5	1.1-3	1-14 tod	1.45	3	39	2980	mascul	14												
7	1.1-3	15 + tod	1.54	2	39	3000	feminir	45	31376.89	161	70.7	136	89	90	280	83	178	69		
5	1.1-3	n„o	1.55	2	36	1650	feminir	24	31288.51	153.5	71.9	114.5	82	90	210	50	140	113	0.5841	0.5693
9	1.1-3	n„o	1.5	4	41	3580	masculin		20182.13	163.75	70.4	152.5	100	89	202	49	111	193	0.578	0.5663
5	1.1-3	n„o	1.52	1	38	2700	feminino		29111.01	167.9	73.2	167.5	106	85	120	53	50	52	0.5795	0.5438
0	1.1-3	n„o	1.52	6	40	2630	mascul	68	26113.38	172.7	82.8	132	62	76	198	43	125	152	0.603	0.5854
10	3.1-6	1-14 tod	1.59	2	40	3600	mascul	51	31139.91	173.45	100.1	119.5	82	86	198	38	104	375	0.602	0.5789
8	1.1-3	n„o	1.61	1	37	2800	feminino		31695.74	168.85	72.1	108.5	66.5	81	179	79	88	89	0.5823	0.5778
11	10	n„o	1.46	1		2850	feminino		12653.19	156.75	47.5	109.5	70	78	149	58	73	56		0.5781
4	3.1-6	1-14 tod	1.63	1	41	3450	feminino													
4	1 ou -	n„o	1.58	3	41	3520	feminir	25												
7	3.1-6	1-14 tod	1.68	3		3530	mascul	30	34914.62	170.2	92	149	87	104	166	58	96	78	0.5786	0.5807
12	6.1-10	n„o	1.68	2	41	3190	feminir	34												
6	1 ou -	15 + tod	1.6	1		990	masculin													
8	1.1-3	n„o	1.52	2	40	3780	feminir	51	16331.48	164.8	55.9	115	61.5	89	214	88	93	135	0.5788	0.5848
4	1 ou -	1-14 tod	1.42	6	40	2900	feminir	74	9778.416	146.15	37.5	102.5	65	84	129	61	53	85		
2	1.1-3	n„o	1.5	5	36	2970	feminir	70	34371.19	160.8	82.4	131.5	74	77	207	79	120	52	0.593	0.5808
4	1.1-3	n„o	1.6	3	41	3750	mascul	83												
8	1.1-3	n„o	1.56	1		3000	masculin		14926.69	176.9	96.4	130.5	65.5	104	150	34	85	120	0.6167	0.5822
4	1 ou -	n„o	1.6	1	42	3800	masculin													
6	3.1-6	1-14 tod	1.57	3	41	3200	mascul	31	3566.61	179.55	61.9	136	79.5	85	153	58	86	45	0.5789	0.5782
8	1.1-3	n„o	1.53	3		3500	feminir	37	20508.73	163.6	63	104	70	78	210	90	102	101	0.5577	0.5719
2	1.1-3	n„o	1.49	4	39	2820	mascul	68	9387.172	173.9	67.2	114	69.5	55	185	80	87	48	0.5814	0.5788
9	3.1-6	n„o	1.58	2	42	4880	mascul	34	28336.59	179.95	91.1	132	86.5	96	173	39	92	245	0.6862	0.5796
6	1.1-3	15 + tod	1.51	2	41	3400	mascul	41	20778.35	183.6	78	135.5	71.5	79	174	70	97	40	0.5808	0.5754
4	1.1-3	n„o	1.62	1	40	3730	masculin		19652.24	184.15	80.8	128.5	71	68	100	33	52	116	0.5781	0.5786

pescmae	prenda	pfumomae	paltmae	pgesta	pidgest	ppn	psex	pint	dmgtotdxa	daltura2012	dpeso	dsysmed	ddiamed	dglicose	dcolesterol	dhdl	dldl	dtrig	dECMICE	dECMICD
3	1.1-3	1-14 tod	1.51	2	39	3030	feminir	84	34956.4	158.8	76.2	122	84.5	104	203	61	104	221	0.5758	0.5844
5	1.1-3	1-14 tod	1.53	1	42	2880	feminino		32647.5	167.6	79.6	114	72	102	197	68	114	61	0.5786	0.5785
12	3.1-6	n„o	1.57	1	41	3950	masculin		37171.23	177.5	107.5	144	89	90	150	42	88	122	0.6313	0.5961
5	1.1-3	n„o	1.59	3	38	3000	mascul	64												
3	1.1-3	15 + tod	1.63	3	40	3800	mascul	28	15967.14	176.4	80.6	114.5	64	121	187	40	90	233	0.5809	0.5711
2	1.1-3	n„o	1.58	4	40	3500	feminir	87	22693.71	165.9	62.9	117	80	84	187	66	107	87	0.5785	0.581
1	1 ou -	1-14 tod	1.5	1		3200	masculin													
5	1 ou -	n„o	1.51	2	39	3220	feminir	102												
4	1.1-3	n„o	1.54	9		2300	masculin		24805.75	177.15	87.9	123.5	74	90	230	55	117	358	0.5808	0.7221
11	1.1-3	n„o	1.5	1	38	3100	masculin													
1	1 ou -	n„o	1.56	1	42	4670	masculin		30570.05	185.45	95.4	113	69.5	80	147	48	80	50	0.5822	0.5984
9	1.1-3	1-14 tod	1.57	1	42	3500	feminino		57483.64	165.4	109.6	102	74	81	237	58	164	83		
19	10	n„o	1.54	3	38	3200	mascul	26												
5	1 ou -	n„o	1.62	1	40	4300	feminino		21824.3	172.95	65.2	106.5	67.5	76	148	53	79	102	0.5732	0.5675
5	1.1-3	n„o	1.54	2	36	3480	feminir	108	26987.79	165.35	70.8	108	72.5	77	201	81	105	69	0.5776	0.5719
14	6.1-10	n„o	1.61	1	37	2900	masculin													
8	3.1-6	n„o	1.65	2	40	3500	feminir	24	30540.87	172	74.5	99	67.5	71	225	78	136	48	0.574	0.5337
6	1.1-3	n„o	1.57	2	41	3560	feminir	33												
5	1.1-3	n„o	1.49	5	38	3500	feminir	55												
3	1 ou -	n„o	1.49	2	37	3230	mascul	25	14218.68	166.05	65.9	129.5	74	88	186	55	122	44	0.6696	0.6093
5	1.1-3	n„o	1.53	1	38	3150	masculin													
1	1.1-3	1-14 tod	1.56	2	40	3810	mascul	22	13966.21	171.8	86	143	78.5	89	187	54	119	58	0.5592	0.5708
1	1 ou -	15 + tod	1.44	13	40	2550	feminir	25	11357.37	147.7	43.7	119	79							
5	1.1-3	n„o	1.61	4		2400	feminir	15	18325.08	156.85	58.9	123.5	81	91	155	66	69	111		
7	3.1-6	n„o	1.53	1	37	3900	feminino													
8	1.1-3	n„o	1.52	3		3160	feminir	24	22805.63	158.55	61.3	105.5	60	77	167	65	88	58	0.5736	0.579
0	3.1-6	1-14 tod	1.49	5		3370	mascul	24	23967.87	179.7	83.8	109	65.5	92	146	64	72	35	0.5709	0.5747
2	1.1-3	1-14 tod	1.51	1	39	1970	feminino													
5	3.1-6	15 + tod	1.56	3	38	2950	mascul	58	36807.32	167.1	82.1	101.5	67.5	105	270	76	158	277	0.5786	0.5789
7	1.1-3	n„o	1.57	2	40	3550	feminir	35	22653.95	154.4	56.8	98	64	93	159	57	91	88	0.578	0.578
16	10	1-14 tod	1.51	3	39	2950	mascul	93	16938.47	164.75	68	111	68	94	207	59	133	72		
4	1.1-3	n„o	1.59	2	39	4350	mascul	53	26998.38	180.15	84.3	146	73	82	157	47	91	59	0.5952	
6	1 ou -	1-14 tod	1.54	1	43	3210	masculin													
6	1 ou -	n„o	1.48	3	42	2700	feminir	15	52581.4	152.7	97.9	130	82.5	79	163	69	86	52		
6	1.1-3	1-14 tod	1.58	3	42	3120	mascul	15		177.15	120.6	154.5	103	110	221	48	138	179		
5	1.1-3	n„o	1.59	2	37	3730	feminir	13	16479.6	159.8	54.6	119.5	79	67	159	63	87	44	0.5853	0.5848
2	1 ou -	1-14 tod	1.54	1		2450	feminino		43592.65	160.9	85	124.5	78	70	191	48	124	95	0.5784	0.5782
4	1.1-3	1-14 tod	1.53	1	40	3530	masculin			174.3	129.6	128.5	79.5	102	204	48	111	193	0.5807	
19	10	1-14 par	1.75	3	42	2900	feminir	42	36981.64	172.2	76.7	130	92	75	179	67	93	91	0.5789	0.5836
0		n„o	1.48	1		2650	feminino													
11	1.1-3	n„o	1.58	1	40	3230	feminino		15684.98	164.35	57.3	133.5	96	74	177	73	87	67	0.5731	0.5602
6	1.1-3	1-14 tod	1.55	1		2650	feminino		44835.11	166.7	96.2	109	70	88	155	46	90	119		
4	1.1-3	15 + tod		5		3380	mascul	53	16607.75	161.75	62.6	121	84.5	85	245	58	168	113		
1	1 ou -	15 + par	1.54	6		3050	feminir	22												
5	1.1-3	15 + tod	1.53	1	36	3300	feminino		27184.67	158.6	63.5	97.5	72	77	154	56	89	39	0.582	0.5752
3	3.1-6	n„o	1.41	2	39	2840	feminir	66	26686.38	153.25	63.4	125	76	77	188	63	113	60	0.578	0.5844
5	1.1-3	1-14 tod	1.61	1	41	2950	masculin		30001.26	175	88.7	132	75	86	170	52	90	156	0.5785	0.5824
14	10	15 + tod	1.57	3		2550	feminir	24												

pescmae	prenda	pfumomae	paltmae	pgesta	pidgest	ppn	psex	pint	dmgtotdxa	daltura2012	dpeso	dsysmed	ddiamed	dglicose	dcolesterol	dhdl	dldl	dtrig	dECMICE	dECMICD
8	3.1-6	n„o	1.58	2	41	3400	feminir	19	41311.48	166.4	89.2	130	78	78	160	72	69	122	0.5822	0.5766
4	1 ou -	15 + tod	1.48	3		4920	mascul	48	28867.98	165.9	89.6	134	94.5	130	245	39	124	454		
0	1.1-3	n„o	1.59	5	39	5120	mascul	107												
5	1 ou -	n„o	1.55	1	37	2080	masculin		19683.29	186.75	86.1	151.5	83	80	146	50	74	62	0.5793	0.5776
11	3.1-6	n„o	1.59	1	40	3140	masculin		23461.84	181	87.4	131	77.5	93	146	42	86	123		
7	1.1-3	n„o	1.45	1	41	4050	masculin													
4	1.1-3	n„o	1.56	1		3750	masculin		27444.89	173.35	98.6	120	76.5	85	140	32	85	93	0.626	0.5822
0	1.1-3	n„o	1.53	1		2950	feminino													
6	1.1-3	n„o	1.5	7		1730	mascul	10												
5	1 ou -	1-14 tod	1.58	4	41	3630	feminir	28	54569.64	155.3	104.5	124	89.5	144	236	47	135	351		0.616
9	3.1-6	n„o	1.56	1	39	3400	feminino		12589.51	162.1	49	131.5	85.5	89	143	52	81	53	0.5738	0.5774
9	1 ou -	15 + tod	1.66	1	40	3340	masculin													
4	1 ou -	1-14 tod	1.58	1	41	4350	masculin		27444.62	169.4	93.6	123	69	92	179	64	103	48	0.5853	0.578
2	1 ou -	1-14 tod	1.69	3		2900	mascul	24												
16	10	15 + tod	1.64	2	39	3600	mascul	63	24542.57	178.1	90.4	135	78	104	178	44	98	223	0.5839	0.5789
3	1 ou -	n„o	1.64	4		3330	feminir	19	24705.88	160.35	63.7	94.5	65.5	77	155	64	65	140		
14	10	n„o	1.58	3	40	3100	feminir	10												
4	1 ou -	1-14 tod	1.58	3	39	3580	feminir	18												
12	1.1-3	n„o	1.55	1	39	4150	feminino													
16	6.1-10	n„o	1.61	3	39	3530	mascul	35	32346.36	180.1	97.9	128.5	83	83	209	42	130	194	0.578	0.5783
6	1.1-3	n„o	1.54	2	39	3000	feminir	32	27735.43	157.95	68.1	124	73.5	96	136	40	81	110	0.6087	
1	3.1-6	n„o	1.52	10	37	4240	feminir	32	50036.23	165.9	104.8	130.5	90	96	202	55	119	191	0.5881	
2	1.1-3	n„o	1.47	1	39	2500	masculin		24021.2	168.25	82.3	122.5	72.5	90	160	48	87	108	0.5622	
12	3.1-6	n„o	1.66	1		2800	masculin		9923.373	182.15	72.2	118.5	66	130	153	56	83	84	0.5756	0.5753
8	1.1-3	n„o	1.5	5	40	3260	mascul	54												
9	3.1-6	1-14 par	1.58	2		3900	masculin		14580.71	184.3	85.1	115	76.5	93	192	44	123	108	0.6173	0.6336
4	3.1-6	n„o	1.63	1	38	4070	feminino		42271.46	160.1	88.6	119	81	121	168	60	95	75	0.5728	0.5769
2	1.1-3	n„o	1.55	2	39	3300	feminir	16	44056.61	153.8	86.5	108.5	77.5	91	208	57	132	109	0.5786	0.5893
4	1.1-3	n„o	1.57	7	38		feminir	23												
5	1 ou -	1-14 par	1.59	2	40	3500	feminir	61	12534.22	157.85	51	129	91	69	202	60	109	155	0.6278	0.5808
8	1.1-3	1-14 tod	1.6	1	38	3200	feminino													
6	3.1-6	1-14 tod	1.52	3	38	3400	mascul	21	13634.12	165.4	68.1	114.5	62.5	77	172	67	98	56	0.5778	0.5793
8	1.1-3	n„o	1.61	1	39	3580	feminino		33126.91	162.25	77.1	120.5	80.5	103	182	66	93	155		
16	3.1-6	n„o	1.65	2	38	3650	feminir	24												
16	10	n„o	1.67	3	40	3330	feminir	41	42551.87	166.15	80.2	112.5	65	87	142	62	64	115	0.5774	0.5782
3	1 ou -	1-14 tod	1.49	3		1690	feminir	71												
2	1.1-3	15 + tod	1.57	2	39	3120	mascul	17												
3	1.1-3	1-14 tod	1.57	2	40	3580	mascul	65	21062.38	177.25	85.2	113.5	71.5	88	256	55	177	85	0.6282	0.5972
13	6.1-10	1-14 par	1.65	2		3300	mascul	23	41714.09	173	109.4	129	82	87	219	70	130	131	0.6112	0.5815
11	1.1-3	n„o	1.56	3	39	3920	feminir	18												
16	3.1-6	n„o	1.52	1	39	4100	feminino		38028.9	158.15	86.6	133	89	88	155	34	81	207	0.5851	0.5876
6	1.1-3	15 + tod	1.61	2		3030	mascul	16		175.7	123.7	132.5	86	84	185	45	116	180		
16	10	1-14 par	1.58	1	35	2170	masculin		16528.13	178	68.4	114.5	57.5	78	161	56	90	94	0.5842	0.5805
8	3.1-6	15 + par	1.63	3	39	4330	mascul	106		188.95	94	124	64.5	87	172	63	100	60	0.5765	0.6228
4	1 ou -	n„o	1.58	1		3000	feminino													
0	3.1-6	1-14 tod	1.54	7		3450	feminir	105	52424.03	151.8	98.2	103	72.5	87	184	70	102	68	0.58	0.59
2	1.1-3	1-14 tod	1.64	6	40	2980	feminir	19	11419.81	166.05	55	106	69	64	138	61	69	38	0.578	0.5779
10	1.1-3	n„o	1.57	2	41	3510	feminino		20888.4	160.1	57.8	119	84	82	150	44	91	97	0.5643	

pescmae	prenda	pfumomae	paltmae	pgesta	pidgest	ppn	psex	pint	dmgtotdxa	daltura2012	dpeso	dsysmed	ddiamed	dglicose	dcolesterol	dhdl	dldl	dtrig	dECMICE	dECMICD
5	1.1-3	1-14 tod	1.52	6	40	3050	mascul	46	13324.49	170.9	72.4	139	79.5	94	196	37	123	128	0.5779	0.6107
4	1.1-3	n„o	1.56	1		3260	masculin													
11	1.1-3	n„o	1.61	9	39	4120	mascul	20												
5	3.1-6	15 + tod	1.61	8	40	3870	feminir	86	48867.74	166.8	106.5	128.5	76	97	178	84	70	100	0.5812	0.5847
12	3.1-6	n„o	1.6	2	40	2930	mascul	14	37530.6	180.95	97.5	133.5	87.5	83	241	39	152	261	0.5788	0.56
10	3.1-6	1-14 tod	1.6	2	39	3080	feminir	45	38904.75	166.6	81.5	117.5	71	82	185	45	123	86		
3	1.1-3	n„o	1.46	1		2600	masculin		6194.797	171.95	58.1	138	82.5	77	168	39	109	102	0.5629	0.5772
0	1.1-3	n„o	1.47	3		3550	mascul	49												
4	1 ou -	n„o	1.58	1	40	3250	masculin		12380.62	176.65	70.1	139.5	75	81	190	55	118	78	0.5767	0.5721
4	1.1-3	n„o	1.62	5		3390	mascul	45	26694.12	174.85	86.4	119	74	76	189	47	126	80	0.5744	0.5889
7	1.1-3	n„o	1.56	2		2990	mascul	54												
4	1.1-3	1-14 par	1.43	1		3870	masculin													
9	3.1-6	n„o	1.66	1	39	3400	feminino			169.35		127.5	78.5						0.5942	0.5809
7	10	1-14 tod	1.58	4	39	2910	mascul	27												
3	1.1-3	n„o	1.64	4	36	3900	mascul	58												
3	1.1-3	n„o	1.52	1	42	3340	feminino		30694.66	164.6	80.4	124	78.5	95	190	52	116	115	0.5793	0.5802
15	1.1-3	1-14 tod	1.55	1	40	3480	feminino		21768.04	164	59.8	117.5	79.5	85	214	80	120	87	0.5802	0.5545
0	1 ou -	n„o	1.63	2	36	2870	mascul	89												
5	1.1-3	1-14 tod	1.55	4	42	3820	mascul	44												
5	1 ou -	15 + tod	1.58	1		2570	masculin		5446.415	177	71.2	118.5	81.5	85	158	74	71	72	0.5868	0.5804
5	1.1-3	n„o	1.56	3	40	2650	mascul	75												
10	3.1-6	n„o	1.6	3	42	3750	feminir	17												
5	1 ou -	n„o	1.56	9	39	3550	mascul	63												
4	1.1-3	n„o	1.55	1	35	2670	feminino		15187.32	158.1	49.9	115	79	77	154	42	100	89		
7	1 ou -	n„o	1.66	5	35	3100	mascul	12												
16	6.1-10	n„o	1.63	1	41	3500	feminino													
5	1 ou -	n„o	1.57	7	39	3250	feminir	23												
0	3.1-6	n„o	1.56	8	42	3560	mascul	28	9730.468	168.2	64.6	141	83	89	231	86	127	723	0.5767	0.5781
4	1.1-3	1-14 tod	1.65	6	36	2500	mascul	95	23754.41	174	83.3	140	89.5	93	212	68	130	59		
0	1 ou -	n„o	1.48	1	42	3160	masculin		19494.27	177.25	78.4	120	77	94	170	48	91	171	0.5796	0.6062
5	3.1-6	n„o	1.61	3	39	2800	feminir	13	53155.27	158.1	104.6	120	82	80	199	46	112	222	0.6032	
5	1 ou -	15 + tod	1.55	1	41	3400	masculin													
4	1.1-3	n„o	1.44	4		3250	mascul	30	21628.52	167.05	76.3	132	89	172	296	45	142	639	0.6211	0.6095
5	3.1-6	n„o	1.6	1	40	4050	masculin		30919.58	182.05	91	117.5	73.5	93	197	47	106	264	0.5786	0.5786
4	1 ou -	n„o	1.56	2	39	3380	mascul	14												
4	1 ou -	15 + tod	1.63	1	39	2590	feminino		27905.35	155	65.6	150.5	98	97	231	73	131	188	0.5637	0.5773
6	3.1-6	n„o	1.53	2	38	2400	feminir	20	20348.47	165.2	59.7	109.5	67	83	140	42	84	72	0.5729	
5	1.1-3	n„o	1.56	1	39	3030	masculin		24701.68	183.35	88.8	142.5	78.5	123	187	46	100	215	0.5803	0.5716
12	6.1-10	n„o	1.67	2	38	4450	feminir	20												
4		1-14 tod	1.43	5		3120	feminir	12												
5	1.1-3	n„o	1.68	1	40	3350	feminino		35745.84	177.35	85.9	133.5	85.5	93	211	62	135	88	0.5804	0.5922
5	1.1-3	n„o	1.61	4		4070	mascul	17												
7	3.1-6	15 + tod	1.44	2	39	3100	feminir	26												
16	6.1-10	n„o	1.59	1	39	3730	masculin		10578.84	176.65	76.9	120	66	64	205	63	122	126	0.5782	0.5755
1	1.1-3	n„o	1.59	2		3680	mascul	23												
5	1.1-3	1-14 tod	1.5	4	40	2730	mascul	27												
3	1 ou -	15 + tod	1.49	8	38	2530	mascul	13	10756.84	158.05	57.8	135	83	74	200	67	110	131	0.5783	0.578
7	3.1-6	n„o	1.58	1	42	3650	masculin													

pescmae	prenda	pfumomae	paltmae	pgesta	pidgest	ppn	psex	pint	dmgtotdxa	daltura2012	dpeso	dsysmed	ddiamed	dglicose	dcolesterol	dhdl	dldl	dtrig	dECMICE	dECMICD
7	1.1-3	n„o	1.54	3	40	3500	feminir	57	42482.15	162.55	97	133.5	81.5	67	176	50	104	119	0.6032	0.5853
0	1 ou -	1-14 tod	1.54	4		4200	feminir	60												
16	3.1-6	n„o	1.7	1	37	2950	feminino													
5	1.1-3	n„o	1.64	1		3550	masculin		36056.77	184	115.7	141.5	88	94	206	39	136	177	0.6195	0.68
0	1 ou -	n„o	1.4	4		3380	feminir	44		155.45		107.5	60.5						0.6035	0.5819
5	1.1-3	n„o	1.6	1	40	2910	feminino		33945.53	159.55	71.8	128	82	128	200	55	115	131	0.5611	0.5789
0	1.1-3	n„o	1.47	2	43	3530	feminir	172	31991.46	159.75	73	102	65	79	194	70	109	82	0.5744	
2	1.1-3	n„o	1.64	2	41	3770	feminir	24	20030.97	164.8	63.4	139	94	86	200	70	102	191	0.5835	0.5803
5	1.1-3	n„o	1.49	3	42	3400	feminino													
6	3.1-6	n„o	1.48	2		3800	mascul	33												
8	3.1-6	1-14 tod	1.55	3	41	2480	feminir	14												
2	1.1-3	n„o	1.49	11	41	3420	feminir	27	25765.42	158.5	64.9	110	76	108	248	48	177	120	0.5756	0.5769
8	1 ou -	15 + tod	1.67	3		3400	mascul	23		177.3	139.1	148.5	98.5	106	190	94	71	122	0.5845	
14	3.1-6	1-14 tod	1.6	1	37	3520	feminino		42301.55	160.8	89.5	107	70	62	180	72	84	95	0.579	0.5837
5	1.1-3	1-14 tod	1.54	2		2700	feminir	20												
4	1 ou -	1-14 tod	1.52	1	41	2950	feminino		8225.769	164.6	46.9	90.5	60	90	170	71	84	61		
5	1.1-3	n„o	1.5	1	39	3150	feminino		42583.66	161.9	85.4	117.5	79.5	62	130	55	63	57	0.5899	0.5785
12	10	n„o	1.5	2	38	3200	feminir	49												
12	3.1-6	n„o	1.52	2	38	3100	feminir	25	16022.3	153.95	50.9	111	70	64	225	65	139	115	0.5991	0.5777
9	1.1-3	n„o	1.51	3	41	3700	feminir	30	23487.07	157.95	58.7	98	62.5	88	227	71	133	81	0.577	0.5544
5	1.1-3	1-14 tod	1.58	2	37	3500	mascul	12	8138.844	172.55	69.2	119.5	60	69	145	51	79	105	0.5818	0.5764
0	1.1-3	n„o	1.44	4	39	2470	feminir	33	29033.85	148.35	66.1	123.5	84	97	176	47	111	125	0.5679	0.5752
6	1.1-3	n„o	1.57	2	41	3830	masculin		14356.71	168.3	68.9	122	70	88	189	64	109	91	0.5678	0.5794
3	1 ou -	n„o	1.57	2	41	2930	feminir	18	23032.84	158.1	59.4	122	81.5	95	200	73	114	65	0.5782	0.5784
12	3.1-6	n„o	1.53	1		3150	feminino													
4	1 ou -	15 + par	1.57	4		1900	mascul	13												
3	3.1-6	n„o	1.58	4	40	3600	mascul	17	5454.961	177.3	65.6	133	77	106	168	61	97	74	0.5782	0.5777
1	1.1-3	15 + tod	1.5	1	42	2850	feminino			162.3	134.8	129	73.5	118	173	58	91	127	0.6408	
0	1.1-3	1-14 tod	1.48	2	38	2880	feminir	49	23974.13	149	60.5	122.5	79	70	145	43	87	53	0.5779	0.5418
10	3.1-6	n„o	1.56	1	39	2870	masculin		37185.99	171.8	97.8	121.5	66.5	121	161	34	84	260	0.6274	0.5625
13	3.1-6	n„o	1.51	1	37	3500	masculin		24585.96	174.6	84.9	126.5	70	76	178	54	103	125	0.5759	
11	10	1-14 par	1.68	1	40	3400	feminino													
13	6.1-10	n„o	1.57	3	38	2950	feminir	12												
2	1 ou -	n„o	1.56	5	39	3890	feminir	12												
7	1 ou -	n„o	1.5	1		2760	masculin		31002.8	177.7	99.4	140	76	82	245	52	150	211		
8	3.1-6	n„o	1.49	2	40	3900	feminir	60	17029.59	161.95	60.7	157.5	104	81	295	74	185	171	0.579	0.5781
3	3.1-6	n„o	1.66	1	38	4500	masculin		8874.386	183.75	78.5	122.5	72	60	206	78	110	102	0.5706	0.5464
3	1 ou -	n„o	1.54	3	41	3800	feminir	36	19090.87	161.05	58.4	109.5	77	73	190	61	110	77	0.5726	0.5768
16	10	n„o	1.55	1	39	3000	feminino													
5	1 ou -	n„o	1.44	3	38	2950	mascul	30												
6	1.1-3	1-14 tod	1.56	1	42	3220	feminino													
4	1.1-3	1-14 par	1.46	1		2990	feminino													
8	1 ou -	1-14 tod	1.51	1		2480	feminino		25980.9	153.05	60.6	126.5	66.5	67	185	62	108	83	0.5658	0.5672
2	1.1-3	1-14 par	1.62	14	41	2950	feminir	21	30519.38	160.8	70.5	114	70	107	235	66	150	85		
8	3.1-6	n„o	1.53	1	38	3600	feminino			161.05	69.1									
7	1 ou -	n„o	1.51	1	37	3120	feminino		24862.82	152.2	71	108.5	71.5	81	223	46	134	222	0.5772	0.6017
9	6.1-10	n„o		2	40	3820	mascul	35	18265.84	178.8	83.7	148.5	88	80	175	60	103	76	0.5786	
10	6.1-10	15 + tod	1.57	2	38	2990	masculin													

pescmae	prenda	pfumomae	paltmae	pgesta	pidgest	ppn	psex	pint	dmgtotdxa	daltura2012	dpeso	dsysmed	ddiamed	dglicose	dcolesterol	dhdl	dldl	dtrig	dECMICE	dECMICD
3	1 ou -	n„o	1.57	1	41	3700	masculin		39658.48	176.2	106.4	135	82.5	93	159	45	102	70	0.5899	0.5808
8	3.1-6	n„o	1.52	5	38	2950	feminir	17	44503.82	157.7	91	112.5	71	96	180	65	83	218	0.5522	0.5784
7	3.1-6	n„o	1.5	2	39	3770	feminir	20												
1	1 ou -	n„o	1.63	4		3240	mascul	20												
2	1.1-3	n„o	1.45	10	43	3400	mascul	166	16127.59	184	82.3	121	69	85	147	50	90	45	0.5781	0.5753
12	1.1-3	n„o	1.58	2	43	3250	mascul	54												
3	3.1-6	n„o	1.51	4		3050	feminir	105	29458.7	158.75	72.1	98.5	62.5	94	212	57	114	171	0.5781	0.5788
5	1.1-3	n„o	1.62	1	42	3920	masculin		37176.97	174	94.7	130	87.5	93	155	35	83	243	0.5714	0.5799
5	3.1-6	n„o	1.55	3	38	3820	mascul	85	38459.45	182.25	118	165.5	103.5	84	220	64	135	92	0.6395	0.584
5	3.1-6	n„o	1.52	1	38	2950	masculin		31011.21	168.9	89.6	131	78.5	94	169	44	99	138		0.5824
5	1 ou -	1-14 par	1.53	3	40	3450	mascul	34	13628.25	182.3	70.9	138.5	86.5	76	239	72	151	87	0.5798	0.58
5	1.1-3	15 + tod	1.53	4	37	2430	feminir	60	15136.03	149.3	49.3	112	74.5	91	183	54	99	160		0.5691
0	1 ou -	n„o	1.48	3	39	3220	mascul	48												
2	1.1-3	n„o	1.51	4	40	2650	mascul	20	23617.61	159.9	75.9	111.5	65	81	156	40	96	135		
5		n„o	1.56	1	40	2580	feminino													
13	3.1-6	n„o	1.69	4	41	3760	feminir	29	31485.71	163.6	71.4	116	77	99	258	83	150	124	0.5756	
5	3.1-6	n„o	1.6	1	39	3650	masculin													
0	1 ou -	1-14 tod	1.52	5	41	3150	feminir	179	42884.3	154.7	88.4	118	68	118	217	51	150	132	0.5856	0.6023
8	1.1-3	1-14 par	1.61	2	39	3800	mascul	29	22642.55	180.65	90.8	125	83	89	152	51	90	66	0.5754	0.5793
8	3.1-6	n„o	1.58	2	42	4450	mascul	47	40443.82	181.5	107.1	143	82.5	119	170	61	98	50	0.5789	0.5916
0	1 ou -	15 + tod	1.47	2		2380	mascul	19												
4	1.1-3	n„o	1.53	2	38	2940	feminir	12	17270.29	167.7	59.7	113.5	73.5	82	148	52	88	76	0.5566	0.5758
4	1.1-3	n„o	1.61	1	38	2920	feminino													
2	1.1-3	n„o	1.46	10		2200	feminir	78												
8	1 ou -	15 + tod	1.62	4		3480	masculin													
6	1.1-3	n„o	1.5	9	41	3800	mascul	25	8074.81	171.45	67	110	57	93	155	40	87	92	0.5771	0.5781
4	1.1-3	n„o	1.62	3	38	3400	feminir	34	27826.53	155.2	71.2	123	84.5	117	182	86	76	70	0.5769	0.5779
5	1.1-3	n„o	1.52	1	41	3330	masculin													
5	1.1-3	n„o	1.46	3	40	2680	feminir	20	25646.56	162.25	63	135	84.5	86	245	68	160	93	0.578	0.5762
4	1.1-3	n„o	1.64	1	39	3020	feminino													
12	10	n„o	1.58	1		3260	feminino			169.4		90.5	61.5						0.5811	0.5795
5	1.1-3	1-14 tod	1.47	1	41	2500	masculin		16545.15	165.15	69.9	123	75.5	93	157	37	108	71	0.616	0.6022
6	1 ou -	1-14 par	1.51	2	40	2870	mascul	34	3135.805	175.3	60.1	124.5	63	72	240	50	144	269	0.5935	0.578
4	1.1-3	n„o	1.65	1	41	3300	feminino		33944.19	164.4	77.3	104	63	86	232	58	150	112	0.6013	0.5801
7	1.1-3	15 + tod	1.7	2	41	3470	feminir	58	44828.06	170.4	87.4	132	91.5	72	189	54	118	93	0.5777	0.5756
6	1.1-3	n„o	1.53	1	39	3050	masculin													
7	1.1-3	n„o	1.58	5	40	3230	feminir	128		168.1		118	77						0.5749	0.5809
11	3.1-6	n„o		2	40	3750	mascul	66	27866.84	178.35	78.2	110.5	67	92	143	49	83	112	0.5791	0.5679
14	10	n„o	1.6	1	39	3180	feminino		24678.16	166.75	69.5	98	55	84	157	55	73	217		0.5766
8	1.1-3	n„o	1.51	1	40	2600	masculin		20357.68	172.55	70.1	124.5	68	91	180	46	116	79	0.5782	0.5815
13	6.1-10	n„o		1	40	3170	masculin		12353.65	176.7	57.5	95	55.5	85	145	39	81	117		
16	10	1-14 tod	1.58	2	40	3260	feminir	22	18773.38	157.8	53.8	103	69	86	156	77	67	77		
5	1.1-3	n„o	1.55	3	39	3400	mascul	29	30584.96	177.15	92	150.5	102.5	98	238	42	131	372	0.5791	
8	10	n„o	1.5	2	40	3400	mascul	11												
8	1.1-3	n„o	1.61	1	42	2470	masculin		5442.688	177.05	56	105	65	75	157	64	87	47	0.5638	0.5422
4	1.1-3	1-14 par	1.57	2	38	3400	feminir	29	35362.42	165.55	78.5	105.5	64	85	191	59	122	55	0.5776	0.5785
4	1.1-3	n„o	1.53	2	40	4150	mascul	58												
3	1.1-3	n„o	1.57	4	41	4170	mascul	127												

pescmae	prenda	pfumomae	paltmae	pgesta	pidgest	ppn	psex	pint	dmgtotdxa	daltura2012	dpeso	dsysmed	ddiamed	dglicose	dcolesterol	dhdl	dldl	dtrig	dECMICE	dECMICD
5	1.1-3	n„o	1.56	1	39	3000	masculin		14556.84	173.4	68.4	110	58	82	185	56	113	59	0.5783	0.577
3	1 ou -	n„o		2		2000	mascul	86												
5	1.1-3	n„o	1.51	1	41	3870	feminino			164.3		97	57.5						0.5784	0.5589
5	1.1-3	n„o	1.51	3	40	3750	mascul	37	6852.812	173.35	73.9	116	60	85	156	58	91	46	0.5765	0.5773
4	1 ou -	n„o	1.63	4	40	2900	mascul	106	29499.37	181.15	87.1	135	90	76	220	57	129	166	0.5809	0.5786
2	1 ou -	1-14 tod	1.54	1		3050	masculin													
18	10	n„o	1.6	1	42	3800	masculin		14487.91	187.55	79.4	119.5	63	66	190	65	108	79	0.576	0.5768
9	1.1-3	n„o	1.5	2		2560	mascul	20	17933.66	180.2	79.1	114	70.5	89	139	49	79	90	0.573	
9	6.1-10	n„o	1.53	4	39	3300	feminir	116												
11	1.1-3	1-14 tod	1.72	8		3300	mascul	39	12946.47	189.5	78.5	139	77	84	186	51	120	169	0.5643	0.5742
5	1.1-3	n„o	1.6	4		3720	mascul	46	10688.54	181.1	64.5	113.5	66	80	202	60	125	69	0.5754	0.5777
5	1.1-3	n„o	1.54	1	39	3200	masculin													
11	3.1-6	n„o	1.56	1	39	3600	feminino		38945.88	164.8	81.7	116.5	80.5	79	302	89	178	192	0.5824	0.579
5	1.1-3	n„o	1.47	1		3780	masculin		4508.113	166.3	53.1	108.5	61.5	81	176	81	82	52	0.5789	0.5714
16	10	n„o	1.47	4	39	2930	feminir	42												
5	1.1-3	1-14 tod	1.5	1	40	3200	feminino													
7	1.1-3	1-14 tod	1.54	2		3460	feminir	16	35562.07	152.5	78	117	74	88	170	63	88	80	0.5878	0.5792
1	1 ou -	1-14 tod	1.63	1		2650	masculin		12558.77	177	81.7	114.5	77.5	76	156	50	93	54	0.5786	0.5782
5	1 ou -	1-14 par	1.57	1	39	3390	feminino													
6	1 ou -	n„o	1.54	4	36	2640	mascul	34												
3	3.1-6	1-14 tod	1.56	2	41	4330	mascul	31												
4	1 ou -	n„o	1.53	3	40	3360	feminir	55	25599.76	162.9	69.9	106	74.5	81	207	63	130	84	0.558	0.584
15	10	1-14 tod	1.59	1		3000	feminino		23816.27	158.05	62.4	99	70.5	73	215	74	127	98	0.5788	
3	1.1-3	n„o	1.56	2	40	3780	mascul	20	35952.85	172.3	109.6	128.5	78	92	164	47	102	74	0.5786	
10	3.1-6	n„o	1.63	1	38	2800	feminino		17558.21	156.4	53.6	108	70	102	203	100	88	42	0.5309	0.562
8	1.1-3	n„o	1.6	9		3150	feminir	68	20704.45	165.6	60.5	97	63	49	104	50	46	31	0.5918	0.6
16	10	n„o	1.59	1	40	3150	feminino		20996.7	161.7	64.3	104	59	84	243	82	147	79	0.5788	0.5788
6	3.1-6	n„o	1.63	2	42	3350	mascul	15												
7	1.1-3	1-14 tod	1.54	2	40	2840	feminir	39	21385.06	150.8	62.3	120.5	68.5	70	180	55	113	77	0.5844	0.5766
6	1.1-3	1-14 tod	1.63	1	40	3120	feminino		33866.43	168.35	78.8	127	81.5	82	225	64	142	108	0.581	0.5913
5	1.1-3	n„o	1.53	2		3000	feminir	23	47392.29	152.6	104.3	128	92.5	291	219	40	139	406	0.6225	0.5851
4	1 ou -	n„o	1.58	2	41	3350	mascul	12	14347.89	179.05	82	128.5	78.5	81	197	70	116	50		
6	1 ou -	1-14 tod	1.57	1		2750	feminino													
19	10	n„o	1.58	2	38	3360	feminir	20												
			1.54	5		3650	feminino		24887.79	154.1	61.5	96.5	63.5	96	170	61	94	50	0.5781	0.5774
0	1 ou -	n„o	1.57	4		4050	mascul	47												
11	6.1-10	n„o	1.67	2	40	3400	mascul	54	8576.24	186.3	67.3	117.5	66.5	94	179	70	94	79	0.5782	0.5788
17	3.1-6	n„o	1.63	3	39	3820	mascul	12	9600.156	179.25	68.8	124	68	87	126	50	65	45	0.5762	0.5865
10	1.1-3	n„o	1.59	1	41	3650	feminino													
7	1.1-3	n„o	1.54	1	41	3500	masculin			180.5	135.2	155	97	131	219	48	138	216		
5	1.1-3	1-14 tod	1.58	2		2900	feminir	42	46704.95	162.25	87.9	121.5	80.5	94	195	43	108	265	0.6938	0.7099
5	1 ou -	n„o	1.56	3	41	2840	feminir	15												
8	1 ou -	n„o	1.52	1	39	4520	masculin		18211.07	174.9	73.6	125.5	78.5	81	182	61	103	97	0.5734	0.5747
4	1 ou -	n„o	1.5	2	42	3800	mascul	12	9883.467	171.65	70.2	135	75.5	96	195	78	99	57	0.5879	0.5783
7	3.1-6	1-14 par	1.62	2		2480	mascul	12												
0	3.1-6	n„o	1.67	2	38	3250	mascul	45	4997.884	186.75	72.8	116	65	84	152	64	78	62	0.5837	0.5785
9	3.1-6	n„o	1.56	1	39	3230	masculin													
4	1.1-3	n„o	1.6	4	37	3100	feminir	29	21598.04	150.1	55.4	100.5	66	85	178	71	89	68		

pescmae	prenda	pfumomae	paltmae	pgesta	pidgest	ppn	psex	pint	dmgtotdxa	daltura2012	dpeso	dsysmed	ddiamed	dglicose	dcolesterol	dhdl	dldl	dtrig	dECMICE	dECMICD
6	1.1-3	1-14 tod	1.62	1	41	3200	feminino		42753.09	162.5	86.6	122	82	110	188	57	116	121	0.5782	0.5804
7	1.1-3	n„o	1.54	1	39	3200	feminino													
5	1 ou -	n„o	1.47	1	40	2580	masculin		18839.6	164.2	85	121	68	97	225	48	139	208	0.5791	0.5826
2	1.1-3	1-14 tod	1.58	6	42	3960	mascul	42												
16	6.1-10	n„o	1.5	3	35	2400	feminir	14	17814.1	153.5	51.6	100	62	99	287	75	198	82	0.5552	0.5739
4	1 ou -	n„o	1.48	1	39	2900	masculin													
5	3.1-6	n„o	1.6	1	40	2830	feminino		34212.03	164.45	72.9	114	80.5	73	210	75	105	184	0.5766	0.5776
4	1 ou -	n„o	1.44	8	39	3530	feminir	27	16312.38	155	54.9	98	60	67	172	62	100	71	0.5669	0.5662
4	1.1-3	1-14 tod	1.52	6	40	3380	mascul	17												
3	1.1-3	n„o	1.56	1	40	2940	feminino		49762.4	163.9	92.2	122	74.5	82	168	38	102	158	0.583	0.5851
5	1.1-3	n„o		1	38	2850	masculin		23255.77	173.35	90.3	118	61	81	191	59	121	49	0.613	0.6478
4	1.1-3	n„o	1.53	2	41	3620	mascul	15	17520.75	173.45	79	127	80	91	186	45	117	116	0.5839	0.5799
7	3.1-6	n„o	1.48	2		2590	mascul	73												
18	6.1-10	n„o	1.62	2	38	3620	mascul	47												
7	1.1-3	1-14 tod	1.53	2		3050	feminino													
3	1.1-3	1-14 tod	1.49	3	40	2850	feminino		26187.52	157	66.9	107	65	83	165	44	108	84	0.5786	0.5938
17	10	1-14 tod	1.57	5	37	2830	mascul	31	17343.08	172.6	77.3	117	70.5	98	233	48	113	399	0.5983	0.588
5	1.1-3	n„o	1.56	2	41	3300	feminir	15		162.75	145.8	138	81.5	102	200	56	129	60	0.5831	
5	1.1-3	1-14 par	1.52	4		2710	mascul	12	27261.56	173.5	83.7	113	68.5	85	253	49	175	141		
0	1.1-3	1-14 tod	1.51	6	39	2540	feminir	24		161.4		108.5	67						0.5783	0.577
8	3.1-6	1-14 tod	1.59	3	37	2820	mascul	13	29566.72	175.95	88.1	110.5	68	100	217	78	127	84	0.5716	
11	1.1-3	1-14 tod	1.78	1	42	3320	feminino		25837.89	166.25	67.3	128.5	85.5	84	230	89	108	134	0.5899	0.6614
8	1 ou -	15 + tod	1.61	1	41	3380	masculin					121	80.5	109	204	60	120	87		
17	10	n„o	1.56	2	40	3880	mascul	23	26403.27	179.95	93.3	132.5	72.5	87	250	62	153	190	0.5724	0.5804
1	1 ou -	15 + tod	1.57	2		2880	mascul	14	6797.473	168.7	61.6	126	71.5	105	191	80	90	96	0.5781	0.5792
1	1 ou -	15 + tod	1.56	1		2450	masculin		7795.569	162.85	56.2	125	69	112	202	36	120	186	0.5641	
5	1 ou -	n„o	1.6	2	38	3780	mascul	30												
5	1.1-3	1-14 tod	1.59	2	38	3400	masculin													
5	1 ou -	1-14 tod	1.49	3	42	3050	feminir	32												
3	1.1-3	15 + tod	1.61	3		3360	feminir	33	30503.87	162.95	68.4	107	67	81	230	66	138	154	0.5676	0.5618
3	1 ou -	n„o	1.57	3	40	3850	feminir	129		166.45									0.5837	
16	10	n„o	1.72	1	40	4100	masculin													
5	1.1-3	n„o	1.6	2	38	3950	mascul	34				128	70	89	182	49	118	60	0.5789	0.5782
15	10	n„o		2	41	3020	feminir	17	28862.32	160.45	68.4	118	81	92	196	65	110	101	0.5633	
7	3.1-6	n„o	1.56	1	37	3550	feminino		44960.4	164.2	95	155	106.5	119	239	58	158	155	0.6017	0.6028
9	1.1-3	n„o	1.61	2	38	2950	feminir	97	14241.98	163.7	49.4	121	75.5	91	189	78	100	83	0.5785	0.5787
4	1 ou -	1-14 tod	1.5	1	41	2550	feminino		31873.35	156.2	71.3	111.5	71	92	208	70	124	50	0.5792	0.5795
5	1.1-3	n„o	1.56	5		2980	mascul	104	23828.03	168.4	69.3	114.5	68	87	224	63	140	98	0.5728	0.576
8	3.1-6	n„o	1.62	2	39	3900	feminir	28	36725.86	163.2	80.1	101.5	64.5	80	160	49	98	50	0.5761	
7	1.1-3	n„o	1.58	2	39	4100	mascul	87	18374.59	171.2	75.2	120.5	62	91	240	94	122	110	0.5846	0.5747
4	3.1-6	1-14 tod	1.51	1	42	3050	feminino		26158.7	152.7	63.5	108	70	78	220	64	145	72		0.5777
12	6.1-10	n„o	1.58	3	40	3250	feminir	24												
7	1.1-3	1-14 tod	1.53	3	36	2700	feminir	46	5241.676	159.1	41.9	109.5	68	116	184	68	106	58	0.5748	0.5781
0	1 ou -	1-14 par	1.55	5		3250	feminir	14												
7	3.1-6	n„o	1.55	2	39	3350	feminir	52	55910.27	159.65	112.3	109	72	73	195	69	115	86	0.5768	0.5822
12	1.1-3	n„o	1.62	3	41	3000	feminir	52	22259.83	162.15	57.9	105	72.5	85	197	65	101	137	0.5774	0.568
14	10	n„o	1.63	3	39	3980	feminir	96												
5	1.1-3	n„o	1.52	2	41	3650	mascul	13	4975.42	170	59.6	139.5	90	86	190	50	120	118	0.5767	0.5784

pescmae	prenda	pfumomae	paltmae	pgesta	pidgest	ppn	psex	pint	dmgtotdxa	daltura2012	dpeso	dsysmed	ddiamed	dglicose	dcolesterol	dhdl	dldl	dtrig	dECMICE	dECMICD
3	1 ou -	1-14 par	1.56	2		3000	feminir	17	21465.88	158.9	63.4	108	71.5	85	165	57	91	78	0.5769	0.5785
8	1.1-3	n„o	1.58	2	38	3200	mascul	30	5935.487	175.35	60.7	134.5	82.5	86	198	60	121	73	0.5787	0.5767
3	1 ou -	n„o	1.67	1	40	3670	masculin		38926.15	184.1	103	132	90	93	198	47	128	126	0.5889	0.616
5	3.1-6	15 + tod	1.54	3	36	2380	feminir	25	26128.53	170.7	65.9	98	63	95	182	66	96	107	0.5795	0.5802
10	3.1-6	15 + tod	1.55	2	38	3100	mascul	35	14180.27	173.1	71.3	135	70	71	98	39	43	40	0.5782	0.575
2	1.1-3	n„o	1.6	2	39	3660	mascul	77	4764.605	175.8	68	129.5	70	77	132	46	75	55	0.5771	0.5773
2	1 ou -	1-14 par	1.59	4		3130	mascul	48	4669.725	180.45	70.9	134	62.5	72	184	68	107	55	0.571	0.5672
2	1 ou -	1-14 tod	1.66	1	41	2630	feminino													
10	1.1-3	n„o	1.53	1	43	3300	feminino		18667.35	161.8	59.8	101.5	58.5	65	190	70	107	88	0.5783	0.5776
8	3.1-6	n„o	1.62	1	41	3460	feminino		34438.82	162.9	76	104.5	59.5	74	218	68	129	96	0.5889	0.5831
5	1.1-3	n„o	1.54	2	42	3780	mascul	47	31237.67	169.85	90.1	124.5	80	81	190	41	116	182	0.5861	0.5764
9	3.1-6	n„o	1.56	2	40	2550	feminir	14	14714.27	157.15	49.4	133.5	94	76	169	58	97	91	0.5746	0.5623
13	3.1-6	1-14 tod	1.66	2	42	3800	mascul	14	21848.08	177.75	81.3	127	80	90	199	47	118	166	0.5802	0.5789
16	10	1-14 tod	1.62	4	40	3670	feminir	34												
5	3.1-6	1-14 par	1.58	3	39	3500	mascul	48												
4	1.1-3	n„o	1.49	2	39	3470	feminir	57	13063.54	159.3	55.4	101	61	80	200	72	119	40	0.5669	
5	1.1-3	1-14 par	1.71	1	40	3730	masculin													
0	1 ou -	15 + tod	1.55	5	37	2810	feminir	19	21941.42	166.95	69.8	114.5	70	72	157	61	86	42	0.5775	0.578
8	1.1-3	n„o	1.57	2	38	3900	mascul	28												
4	1.1-3	n„o	1.53	4	39	3900	mascul	24	33618.69	179.7	104.8	137.5	89	96	261	59	178	114	0.6121	0.6274
6	1.1-3	n„o	1.51	3	37	2700	feminir	151	22378.26	162.3	64.2	104.5	62	75	171	62	100	37	0.6277	0.5939
1	1 ou -	1-14 par	1.53	4		3570	feminir	14												
2	1 ou -	1-14 tod	1.49	7		2550	feminir	36	27042.5	158.05	75.7	120	78.5	72	165	66	86	97	0.5762	
10	3.1-6	n„o	1.58	2	40	3950	feminir	64	43780.58	156.9	85.4	112.5	70.5	85	215	58	138	106	0.5701	0.5792
4	1 ou -	n„o	1.56	5	42	3320	feminir	19	60866.43	164.95	118.6	132	87	116	219	46	97	436	0.583	
12	6.1-10	n„o	1.62	3	39	2950	mascul	23												
4	1.1-3	1-14 par	1.58	2	41	4030	mascul	11												
5	1.1-3	1-14 tod	1.55	1	41	4160	feminino		44062.71	168.7	94.7	117	74.5	82	237	76	150	79	0.5833	0.5831
3	1 ou -	n„o	1.54	4	41	3960	feminir	88												
5	1.1-3	n„o	1.52	2	41	2650	feminir	15												
11	3.1-6	n„o	1.57	2	39	3160	feminir	83												
8	1 ou -	15 + tod	1.5	1	35	2300	masculin													
7	1 ou -	n„o	1.57	3	39	3100	mascul	46												
3	1 ou -	n„o	1.59	5	39	3320	feminir	36												
7	1 ou -	n„o	1.65	2	39	4800	feminir	38	36927.36	171.6	82.1	99.5	62.5	83	183	73	100	36	0.5783	0.5798
4	3.1-6	n„o	1.53	1	40	3940	masculin		2668.172	167.25	54.8	117	72.5	113	150	69	64	57	0.5681	0.5779
13	6.1-10	n„o	1.6	1	39	3500	masculin		16072.89	173.35	71.4	114.5	70	96	196	50	124	134	0.5823	0.5813
0	1 ou -	1-14 tod		5		2590	mascul	14												
15	3.1-6	n„o	1.6	1	41	3700	masculin		40280.07	181.45	108.9	123	79	82	197	49	120	126	0.5807	0.5781
8	1.1-3	n„o	1.54	1	36	1450	feminino		43372.23	154.3	81.9	118	87	81	176	51	112	79	0.5858	0.5823
5	1.1-3	n„o	1.46	1	39	3350	feminino		34557.33	158.5	74	117.5	80	89	156	75	73	61		
14	10	1-14 tod	1.67	3	39	3600	mascul	44	18730.63	173.2	73.8	115.5	66	111	150	51	81	80		
4	1.1-3	1-14 par	1.55	2	37	2680	mascul	51	42162.82	173	103.4	124.5	75	112	179	66	99	75	0.5803	0.5719
7	1.1-3	n„o	1.6	1	39	3550	feminino		39252.92	166.2	85.3	113.5	73	90	241	89	125	135	0.5776	0.602
17	6.1-10	n„o	1.68	2	40	3300	mascul	14												
4	1.1-3	1-14 par	1.58	2	40	2750	feminir	13	25208.64	160.95	70.2	109.5	58	87	182	64	108	58	0.5783	0.5803
2	1.1-3	n„o	1.51	1		3200	masculin		23763.88	174.95	85.7	125.5	80	169	288	37	138	615	0.708	0.6131
4	1 ou -	15 + tod	1.51	2		3010	feminir	85												

pescmae	prenda	pfumomae	paltmae	pgesta	pidgest	ppn	psex	pint	dmgtotdxa	daltura2012	dpeso	dsysmed	ddiamed	dglicose	dcolesterol	dhdl	dldl	dtrig	dECMICE	dECMICD
7	1.1-3	n,,o	1.58	3		3320	feminir	41	19097.67	157.65	55.2	107	68	85	224	76	120	114	0.5792	0.571
8	1.1-3	n,,o	1.53	1	42	3530	masculin													
12	1 ou -	n,,o	1.64	4	36	1700	feminir	27												
2	1 ou -	n,,o	1.49	2	39	2820	feminir	60	19552.73	158.2	58.6	100	64	87	200	65	108	148		
3	1.1-3	n,,o	1.61	2	41	3100	mascul	19	5155.044	172.45	64.1	129.5	71	60	135	40	78	68	0.5778	0.579
0	1.1-3	n,,o	1.61	1	41	3900	masculin		8880.183	173.4	67	118.5	71.5	82	162	46	100	66	0.5801	0.5788
12	6.1-10	1-14 par	1.68	2	39	2370	mascul	66	16186.72	180.55	74.4	123	75	74	225	60	148	69	0.5704	0.5753
5	1 ou -	n,,o	1.56	2	40	3450	feminir	26	31601.21	163.65	80.6	125	76	87	150	40	96	100	0.5773	0.6506
9	1.1-3	n,,o	1.59	7	40	2830	feminir	11												
8	1.1-3	n,,o	1.53	1	41	3750	masculin													
0	1 ou -	1-14 par	1.55	2		3120	feminir	16												
7	1 ou -	1-14 par	1.51	1	39	3880	masculin													
5	3.1-6	n,,o	1.54	10	40	3270	mascul	60												
8	1.1-3	n,,o	1.53	3	40	4000	mascul	15												
12	1.1-3	n,,o	1.59	2	39	3550	masculin		10235.44	184.05	78.3	118.5	73.5	57	120	40	64	54	0.5781	0.5773
9	1.1-3	1-14 tod	1.45	1	41	3240	feminino		21738.68	152.5	58.9	101	61	68	145	44	90	77	0.5746	0.578
4	1.1-3	1-14 tod	1.57	4	39	3650	mascul	26	14717.7	168.15	76	136	59.5	84	206	47	112	259	0.7256	0.7356
7	1 ou -	n,,o	1.58	2	38	3430	feminir	41	24072.93	163.4	63	112	74	69	169	57	98	63	0.5786	0.5755
3	1.1-3	n,,o	1.51	3	39	2900	feminir	111	21545.91	154.1	58.4	112.5	68	77	176	40	115	112	0.5711	
17	3.1-6	n,,o	1.62	3	35	2300	feminir	106	23343.76	166.8	66.6	142	90	74	226	72	117	221	0.5811	0.5781
3	1 ou -	15 + tod	1.48	5		2940	feminir	14	32497.29	154	79	115	77	186	341	35	99	839	0.5781	0.579
2	1.1-3	n,,o	1.55	5	40	3370	feminir	84	42023.55	162.3	89.3	118	83.5	93	176	64	100	78		
8	3.1-6	n,,o	1.6	1	39	3550	feminino		30152.17	163.5	67.1	120.5	79.5	83	205	64	126	80		
6	6.1-10	n,,o	1.47	3	41	3270	mascul	53												
7	1.1-3	n,,o	1.55	2	40	3700	mascul	23												
5	1.1-3	1-14 par	1.58	2	37	3000	feminir	36												
3	1 ou -	1-14 tod	1.48	1		3050	masculin		53028.74	165.9	116.3	146	98.5	100	279	58	179	241		0.5805
16	10	n,,o	1.56	5	37	2600	feminir	48												
5	1.1-3	n,,o	1.59	2	42	4200	mascul	60	25505.66	174.75	86.5	119.5	71.5	102	180	56	103	74	0.5823	0.5817
2	1.1-3	1-14 par	1.58	3	40	4250	feminir	31	26013.15	170.8	74.6	118	77	128	140	60	69	39	0.5836	0.5785
8	3.1-6	1-14 par	1.5	5		3020	feminir	12	32248.37	168	82.1	119.5	80.5	87	222	48	141	212	0.5823	
0	1.1-3	1-14 tod	1.55	6		3700	feminir	36	49560.32	164.7	106.8	114.5	79	85	161	71	83	45	0.6164	
2	1 ou -	1-14 tod	1.63	1		2960	masculin		11441.12	175.5	71.2	128	74	71	179	80	81	52	0.5781	0.5781
1	1 ou -	1-14 tod	1.56	2	39	3000	feminir	20												
18	10	1-14 par	1.68	2	39	3330	feminir	42	26620.04	173.15	69.5	103	66	102	230	78	116	203	0.5787	0.5828
8	1.1-3	n,,o	1.47	5	42	3580	mascul	56	11796.08	173.4	75.6	115.5	68	88	136	61	54	101	0.5778	0.5782
4	3.1-6	1-14 tod	1.64	2	41	3810	feminir	115	24249.16	159.95	66.3	114.5	67.5	80	188	60	110	91	0.576	0.5782
0	1 ou -	n,,o	1.51	6	40	3480	mascul	35												
7	1.1-3	n,,o	1.57	3	39	4600	mascul	34												
16	3.1-6	n,,o	1.51	2	38	3500	mascul	16												
2	1 ou -	n,,o	1.57	1	39	3250	masculin		8653.943	177.4	65.4	145.5	80.5	96	230	79	132	94	0.578	0.5757
0	1 ou -	1-14 tod	1.58	3	39	3370	mascul	10												
0	1 ou -	n,,o	1.55	6	42	3980	mascul	66	15489.34	179.9	74.2	123	71	89	181	57	111	64	0.5755	0.5498
5	1.1-3	1-14 par	1.68	1	38	3220	masculin		13444.66	181.2	64.1	114.5	69.5	66	192	66	117	36		
12	3.1-6	1-14 tod	1.51	2		1440	feminino		18358.39	164.25	59.5	120.5	81							0.5796
8	1.1-3	15 + par	1.65	3	41	3480	mascul	32	18501.11	174.3	74.4	109.5	74.5	91	209	55	141	62	0.5683	0.5691
6	1.1-3	n,,o	1.52	1	40	2950	masculin					122.5	80.5	130	163	40	99	180		
0	1.1-3	n,,o	1.54	1	37	3100	feminino													

pescmae	prenda	pfumomae	paltmae	pgesta	pidgest	ppn	psex	pint	dmgtotdxa	daltura2012	dpeso	dsysmed	ddiamed	dglicose	dcolesterol	dhdl	dldl	dtrig	dECMICE	dECMICD
12	3.1-6	1-14 par	1.51	2	40	3060	feminir	38	24954.26	160.4	64.4	119	78.5	86	219	64	123	197	0.5795	0.583
3	1 ou -	n„o	1.6	1	41	3550	feminino		19427.82	156.85	60.6	111.5	71	83	158	62	79	57	0.5753	0.5745
8	3.1-6	15 + tod	1.63	7	40	2670	mascul	205	14514.76	171.4	69.3	116.5	73	120	166	38	97	207	0.5805	0.5783
3	1 ou -	15 + tod	1.48	1	40	2600	masculin		23247.84	176.3	87.1	138	73	85	189	51	115	123	0.5802	0.5423
4	1.1-3	n„o	1.6	3	38	3000	feminir	57	28244.49	161.7	68.4	120	83	85	195	52	125	100	0.5774	0.5786
3	1 ou -	1-14 tod	1.55	2		3350	mascul	14	8969.053	170.2	73.1	131.5	77	91	196	59	121	104	0.6311	0.5833
3	1 ou -	15 + tod	1.49	2		2700	feminir	21												
5	1 ou -	15 + tod	1.62	5	40	2470	feminir	11	46343.59	162.45	93.5	130	91.5	73	219	74	117	197	0.5737	0.5693
3	3.1-6	n„o	1.58	4	43	3200	mascul	53	22158.99	171.2	77.1	116	76.5	80	197	68	105	114	0.5785	0.5722
5	1.1-3	n„o	1.53	1	36	2750	masculin		48657.34	171.6	110.2	133.5	85.5	75	175	48	110	90	0.5794	0.5805
8	1.1-3	n„o	1.55	2		3150	masculin		19562.02	180.25	78.1	145	83.5	149	251	61	146	244	0.5872	0.579
5	1.1-3	1-14 tod	1.59	4		3330	mascul	119												
11	3.1-6	1-14 par	1.58	1	41	3350	feminino		26015.84	156.95	61.1	108	65	86	145	49	78	111	0.5781	0.5782
0	1.1-3	n„o	1.65	2		3200	feminir	29	20946.21	157.65	59.9	112.5	78	72	172	79	77	39	0.5775	0.5705
5	3.1-6	n„o	1.67	2	40	3400	feminir	49												
7	3.1-6	n„o	1.59	2	39	3200	mascul	23												
7	1.1-3	n„o	1.51	3	38	3050	mascul	35	17473.3	169.95	63.2	118	78	106	165	32	84	259	0.578	0.5891
3	1.1-3	n„o	1.49	6		2350	feminir	25												
9	1.1-3	n„o	1.61	2		2970	feminir	28	22047.3	161.9	65	121.5	71.5	85	155	38	85	165	0.5787	0.5781
2	1 ou -	n„o	1.6	1	26	800	feminino													
3	1.1-3	15 + tod	1.55	5		2950	feminir	63	34409.96	160.1	78.3	126	83.5	105	229	70	140	108		
5	1.1-3	n„o	1.55	5	39	2800	mascul	29	22783.4	175.15	95.8	130.5	87	121	234	61	124	270		
4	3.1-6	n„o	1.64	4	43	2850	mascul	135	18866.82	180.05	73.9	115	71						0.5888	0.5779
3	1.1-3	1-14 tod	1.48	3		2670	mascul	23												
1	1.1-3	n„o	1.63	3		2500	feminir	90	23708.2	156.5	60.7	102	65.5	95	226	37	147	166		
6	1 ou -	1-14 tod	1.54	1	38	2430	feminino													
2	1 ou -	n„o	1.51	6	41	2890	feminir	86	36378.5	161	83.2	128.5	86	100	201	57	112	173		
3	1 ou -	n„o	1.58	1		3200	feminino		43812.15	151.05	93.9	199.5	121	244	166	62	79	114	0.7189	0.5924
3	1 ou -	n„o	1.62	4	39	3180	mascul	21												
6	1.1-3	n„o	1.59	1	40	3270	masculin													
2	1.1-3	1-14 tod	1.58	4	39	2800	feminir	117												
5	1 ou -	n„o	1.5	3	41	2750	feminir	45	16826.74	166.5	56.6	146.5	98	72	130	51	66	48	0.5781	0.5758
9	1.1-3	1-14 tod	1.57	3	40	3030	mascul	20												
3	3.1-6		1.51	1	41	4150	masculin		14373.06	175.5	68.6	121.5	68.5	87	193	54	130	53	0.5733	0.5758
4	1.1-3	15 + par	1.45	3		3180	mascul	24												
0	3.1-6	n„o	1.48	3	39	3670	mascul	84	21510.2	183.55	84.8	132.5	78.5	65	170	56	99	117	0.5781	0.5773
16	1.1-3	n„o	1.61	1	41	3100	masculin													
3	1 ou -	15 + tod	1.49	6	35	2650	mascul	13												
3	1 ou -	n„o	1.61	2	37	3520	mascul	59												
4	1.1-3	15 + tod	1.44	3	42	3250	feminir	62	33895.28	164.7	78.4	138	83.5	76	178	58	105	80	0.5813	0.5793
8	1.1-3	n„o	1.63	1	38	3270	masculin		8191.692	172.55	71.2	115	67	77	214	70	123	93	0.5785	0.5516
6	3.1-6	1-14 tod	1.42	1	39	3220	feminino													
6	6.1-10	1-14 par	1.6	1		2490	feminino		50080.39	154.55	92.4	150	99	72	188	54	118	73	0.5839	0.5795
5	1.1-3	n„o	1.55	2	38	3600	mascul	58	9224.473	173.15	68.2	131.5	78	88	169	52	93	98	0.5773	0.5758
8	1.1-3	1-14 tod	1.61	1		3180	feminino		24745.74	163.1	62.6	111.5	75	75	209	79	114	95	0.5778	0.5768
8	3.1-6	15 + tod	1.55	2	42	3300	feminir	40	13717.07	154.7	48	118.5	82.5	83	206	68	120	134	0.5702	0.5759
3	1 ou -	1-14 par	1.57	3	42	2750	feminir	78	56507.81	159.05	116	121	80	79	178	53	110	60	0.5916	
15	6.1-10	n„o	1.55	1	41	3450	masculin		9662.287	168.75	68.9	117	64	92	182	71	89	143	0.5702	0.5782

pescmae	prenda	pfumomae	paltmae	pgesta	pidgest	ppn	psex	pint	dmgtotdxa	daltura2012	dpeso	dsysmed	ddiamed	dglicose	dcolesterol	dhdl	dldl	dtrig	dECMICE	dECMICD
5	3.1-6	n,,o	1.54	6	38	3500	mascul	26												
3	1 ou -	n,,o	1.56	2	39	3000	feminir	49												
4	1.1-3	1-14 par	1.5	3	39	3540	mascul	30												
5	1 ou -	n,,o	1.61	1	39	3120	feminino		14606.17	158.6	54.1	119.5	69.5	79	189	45	125	101		
5	1 ou -	n,,o	1.63	5	42	3560	feminir	17												
16	6.1-10	n,,o	1.72	1	41	3100	feminino													
4	1.1-3	n,,o	1.56	2		3000	feminino		24653.95	157.5	62.8	127.5	89	93	258	64	170	155		
5	3.1-6	n,,o	1.63	3	40	4220	feminir	44	27437.37	166.2	77.2	101	67.5	66	216	79	123	66	0.5704	0.5583
3	1.1-3	n,,o	1.57	2		2840	masculin		23017.14	175.6	80	131	83	95	212	45	141	165	0.5787	0.5782
5	1.1-3	n,,o	1.56	3	39	3900	mascul	22												
2	1.1-3	n,,o	1.57	3		2500	feminir	23	18285.27	159.4	52.3	109	67	86	139	47	74	86	0.5713	0.5734
5	1.1-3	n,,o	1.6	2	41	2730	feminir	32	16746.63	162.6	55.4	114	72.5	80	150	43	94	48	0.5996	0.5781
4	1.1-3	n,,o	1.51	1	41	2650	masculin		31411.84	168.95	82.9	104.5	70	101	266	51	160	321	0.5799	0.5811
4	1.1-3	1-14 par	1.54	2		3130	mascul	34												
17	3.1-6	n,,o	1.51	1	38	3250	masculin		25872.78	169.2	87.1	136	88.5	83	216	61	140	76		
5	1.1-3	n,,o	1.6	1		1820	masculin													
5	1.1-3	n,,o	1.51	1	39	2880	feminino													
8	1.1-3	1-14 tod	1.65	1	40	3700	masculin		30079.18	177.05	94.2	159.5	87	115	218	43	120	328	0.5848	0.5839
3	6.1-10	n,,o	1.59	2	36	3660	feminir	194	43116.38	164.45	85.9	98.5	67	64	135	57	71	40	0.5795	0.5683
20	10	1-14 par	1.68	1	41	4500	masculin			187.95	129.8	153.5	87.5	97	198	49	114	213	0.5789	0.6332
3	1 ou -	1-14 tod	1.54	1		2800	feminino													
11	1.1-3	n,,o	1.62	2	42	3650	mascul	48												
0	1.1-3	1-14 tod	1.54	1		3180	masculin													
6	1.1-3	1-14 tod	1.58	2		2400	feminir	26	57422.05	161.75	107.8	127	77.5	88	245	82	140	113		
3	1.1-3	n,,o	1.66	3	39	3210	mascul	151												
4	1.1-3	n,,o	1.56	3		2850	feminir	28	17456.2	161.4	62.3	134	78.5	59	194	59	116	115	0.5776	0.5787
5	1.1-3	1-14 par	1.58	2	36	2830	feminir	64	38612.51	151.45	80.1	108	65	106	165	35	101	141	0.5686	0.5651
9	1.1-3	n,,o	1.58	2		3190	mascul	11	8273.628	167.95	68.8	123	81.5	87	169	54	91	106	0.5778	0.578
12	3.1-6	n,,o	1.58	1	42	3150	masculin													
9	1.1-3	1-14 tod	1.59	4	40	3750	mascul	84												
4	1 ou -	15 + tod	1.53	2	42	2980	feminir	33	31005.19	165.1	71.8	99	65	51	140	80	51	36		
0	1 ou -	n,,o	1.62	3	40	3050	mascul	46	2492.416	174.15	53.9	119	70	86	202	61	131	70	0.5784	0.5781
5	1.1-3	n,,o	1.65	6	38	3450	mascul	47	30381.66	178.75	101.4	118	71	142	269	33	112	529	0.5841	0.5808
12	3.1-6	n,,o	1.64	2	38	3630	mascul	54		181.5	157.2	132	97.5	106	210	44	140	195		
16	3.1-6	n,,o	1.58	4	40	3350	mascul	20												
4	1.1-3	1-14 tod	1.58	5	40	3630	feminir	55												
3	3.1-6	n,,o	1.64	1	42	3020	feminino		22716.21	179.95	65.3	128.5	80	66	228	74	136	105	0.5639	0.5766
16	10	n,,o	1.65	3	38	2440	feminir	41												
6	1.1-3	1-14 tod	1.43	2		3000	feminir	31												
12	1.1-3	n,,o	1.57	1	41	4120	feminino													
9	3.1-6	n,,o	1.55	2	39	3660	mascul	47												
16	10	n,,o	1.62	2	41	3550	mascul	155	43442.34	187.6	106.3	120.5	81	88	171	42	106	123	0.5734	0.5763
5	1.1-3	1-14 tod	1.7	2	40	3300	mascul	23		182.65	92.6			85	135	46	72	118	0.5742	0.5781
5	1 ou -	n,,o	1.58	1	43	2980	feminino		30566.1	154.5	69	132	90.5	74	215	62	133	107	0.5781	
12	1.1-3	n,,o	1.61	1	37	3000	feminino		22418.78	162.9	60.3	101.5	72.5	53	156	62	79	115	0.5551	0.5583
7	1.1-3	1-14 tod	1.57	2	39	2200	feminir	20	25920.78	153.95	58.2	109	68.5	89	215	95	99	105	0.5645	0.5753
5	3.1-6	n,,o	1.53	1		2000	masculin		17652.96	158.5	71.6	110	74.5	79	190	65	105	119	0.5795	0.5921
5	3.1-6	n,,o	1.65	2	39	3750	mascul	121	17777.23	181.85	74.8	114	68.5	61	150	68	76	45	0.5784	0.5755

pescmae	prenda	pfumomae	paltmae	pgesta	pidgest	ppn	psex	pint	dmgtotdxa	daltura2012	dpeso	dsysmed	ddiamed	dglicose	dcolesterol	dhdl	dldl	dtrig	dECMICE	dECMICD
7	1.1-3	n„o	1.54	2	39	2810	feminino		20461.43	161.6	62.2	126	76.5	96	167	75	84	51	0.5785	0.5786
3	1 ou -	n„o	1.5	3	40	3190	mascul	50												
3	3.1-6	15 + tod	1.46	4	38	3000	mascul	26												
5	1 ou -	n„o	1.45	1		2100	masculin													
9	3.1-6	n„o	1.5	3	40	2600	feminir	21												
6	1 ou -	n„o	1.56	6		3410	mascul	18	14320.86	176.2	73.9	137	82.5						0.5896	0.5781
8	1.1-3	n„o	1.66	1	40	3140	feminino		40774.92	173.9	82.3	108	72.5	97	160	45	102	70		
5	1.1-3	1-14 par	1.56	1	41	3650	masculin		16401.98	167.55	69	117.5	76.5	93	200	53	130	71	0.5762	0.5728
7	1.1-3	n„o	1.53	3	40	3000	feminir	24	6399.77	158.4	45.9	108	63.5	68	174	77	86	73	0.5776	0.5746
2	1 ou -	n„o	1.55	5		2880	mascul	26												
2	1.1-3	n„o	1.57	9		2860	feminino		45771.88	154.8	92.9	127	79	74	170	51	105	81		
3	3.1-6	15 + tod	1.63	1		3680	feminino		44425	164	85.6	115	76	69	165	63	92	43	0.5781	0.5896
4	1.1-3	n„o	1.57	3	40	3280	mascul	89												
6	1.1-3	n„o	1.57	1		2900	masculin		32296.96	173.25	89.4	131.5	88	70	182	48	120	72	0.5784	0.5834
4	1 ou -	1-14 tod	1.64	3	43	3480	mascul	28	9060.46	167.9	62.3	124.5	80	85	193	55	121	67	0.5793	0.5742
4	1 ou -	n„o	1.61	8	36	3450	mascul	34	12912.42	169.85	77.4	140.5	90	87	158	47	96	64	0.6187	0.5861
8	1.1-3	n„o	1.52	3	38	2680	feminir	24	17955.55	157.1	59.5	122.5	81	76	190	68	111	80	0.5747	0.5794
8	1.1-3	n„o	1.61	1	37	2840	feminino		37421.64	172.2	86.2	111	74.5	99	254	99	120	187	0.5797	0.5804
9	10	n„o	1.51	2	38	3150	mascul	14												
2	1.1-3	1-14 par	1.53	1		3450	masculin													
5	1 ou -	1-14 par	1.53	4	37	3700	mascul	13												
4	1 ou -	15 + tod	1.6	3	38	3150	mascul	20												
11	1.1-3	n„o	1.53	1	34	1980	feminino													
5	3.1-6	n„o	1.59	4		2930	feminir	45												
0	1 ou -	n„o	1.6	6		3410	mascul	13												
4	1 ou -	1-14 tod	1.49	2	37	2670	feminir	12												
0	1 ou -	15 + tod	1.66	4		3500	mascul	120												
3	1 ou -	n„o	1.55	1	37	2330	masculin													
2	1.1-3	1-14 tod	1.54	14	38	3730	mascul	14	8851.515	172.5	64.9	135.5	80	80	190	91	80	74	0.5788	
6	3.1-6	n„o	1.66	8	37	3300	mascul	57	35971.14	166.4	89.6	126	85.5	92	288	70	197	144	0.6839	0.6593
			1.63	1	39	2700	masculin													
7	1.1-3	15 + tod	1.52	7	42	2850	feminir	25												
1	1 ou -	1-14 tod	1.5	3	34	1870	mascul	19												
4	1 ou -	n„o	1.52	4	38	2500	feminir	12												
11	6.1-10	n„o	1.57	1	39	3350	feminino		26508.29	160.3	63.3	110	79.5	77	118	81	29	55	0.5776	
12	3.1-6	15 + tod		1		2140	masculin		20779.28	174.6	79.6	135	86.5	121	181	65	77	173		
5	1 ou -	n„o	1.55	1	40	3270	feminino													
7	1.1-3	1-14 tod	1.66	1	38	3500	masculin		17577.5	182.5	88.3	113.5	71	103	157	35	73	296	0.5705	0.5796
0	1.1-3	1-14 tod	1.6	6	43	3640	feminir	46	27601.41	167.45	78.2	116	76	93	150	53	80	104	0.5786	
5	1 ou -	n„o	1.51	2	41	3970	mascul	12	21694.99	172.1	81.1	138.5	87.5	92	190	41	98	247	0.582	0.5779
5	1.1-3	n„o	1.57	5		2400	mascul	237												
6	1.1-3	1-14 tod	1.58	2	40	4150	mascul	37	28927.68	167.8	81.5	131.5	78.5	101	164	39	93	121	0.5781	0.5803
4	1.1-3	1-14 par	1.68	3	38	2800	feminir	34												
6	1 ou -	n„o	1.59	1	39	2420	feminino		11407.63	158.45	47.2	112.5	74.5	114	156	50	99	41	0.5777	0.5783
7	1.1-3	n„o	1.51	1	39	3680	feminino		29371.74	157.9	71.1	111.5	75.5	85	150	57	78	52	0.5753	0.5793
4	1.1-3	n„o	1.44	5	37	3200	mascul	68												
3	1 ou -	n„o	1.48	7	40	3500	feminir	29												
12	3.1-6	n„o	1.51	1	39	2300	feminino		8657.813	154	42.2	107.5	70	76	166	60	89	71		

pescmae	prenda	pfumomae	paltmae	pgesta	pidgest	ppn	psex	pint	dmgtotdxa	daltura2012	dpeso	dsysmed	ddiamed	dglicose	dcolesterol	dhdl	dldl	dtrig	dECMICE	dECMICD
7	1 ou -	n„o	1.52	1	40	3700	feminino		21054.29	173.8	66.9	105.5	66	109	227	78	122	108	0.5804	0.5843
6	3.1-6	n„o	1.58	2	40	3800	mascul	56	20433.88	173.6	80.2	122.5	76.5	68	229	78	126	60	0.5818	0.5785
2	1 ou -	n„o	1.55	1	40	2970	masculin		29212.04	165.75	82.7	120.5	82.5	86	194	63	88	220	0.6038	0.5848
8	1.1-3	1-14 tod	1.55	3	41	3590	mascul	90	25595.87	170.4	83.1	128.5	85.5	99	176	38	111	128	0.6041	0.5804
5	1.1-3	n„o	1.56	2	40	3550	feminir	82												
2	1.1-3	n„o	1.6	1	41	3400	masculin		21183.99	173.05	81.5	134	79	97	247	47	142	303	0.5785	0.5958
7	1 ou -	n„o	1.51	2	36	2220	mascul	15												
5	1.1-3	n„o	1.54	1	30	1650	masculin													
2	1.1-3	n„o	1.42	1	36	2820	masculin													
3	1.1-3	n„o	1.61	2		3520	mascul	19	15070.63	174.6	70.9	122.5	76	88	178	60	97	66		
7	1.1-3	n„o	1.54	1	37	2980	feminino		28843.74	157.85	69.6	123	68	104	293	67	176	502	0.5814	0.5864
11	1.1-3	n„o	1.47	1	40	3150	masculin													
5	3.1-6	1-14 tod	1.69	2	40	4000	mascul	58	4678.622	183.7	75.2	130	76	94	197	64	105	98	0.5808	0.5783
16	6.1-10	1-14 par	1.64	2	39	4100	mascul	11												
7	3.1-6	1-14 tod	1.47	1	38	2350	masculin													
4	1.1-3	n„o	1.55	3	37	4000	mascul	62	22204.66	174.5	81	135.5	86.5	105	157	37	99	87	0.5808	0.5769
4	1 ou -	n„o	1.57	5		2770	feminir	12												
3	1 ou -	1-14 tod	1.63	1	40	2730	feminino													
16	6.1-10	n„o	1.61	6		2950	mascul	39	6772.041	179.8	76.5	126	72	85	233	75	141	46	0.5633	0.5775
3	1 ou -	1-14 tod	1.52	1	41	3100	feminino													
8	1.1-3	n„o	1.56	3	38	3700	feminir	66	36926.51	162.25	77.1	117	79	97	216	67	111	209	0.5795	0.5578
0	1 ou -	n„o	1.56	3		2100	feminir	13												
9	3.1-6	1-14 tod	1.5	2		3450	mascul	27												
5	1.1-3	n„o	1.58	1	41	3800	masculin													
7	1.1-3	1-14 tod	1.55	1	40	3440	masculin		10210.38	162.6	64	110.5	66	105	225	51	149	147	0.5775	0.5816
5	1.1-3	1-14 tod	1.65	1	39	3340	masculin		29779.8	182.1	94	136	73.5	79	191	61	98	99	0.5802	0.5783
5	1 ou -	n„o	1.47	1	37	3150	masculin		10557.08	166.6	62.6	137	81	105	224	63	145	88	0.662	0.6332
15	3.1-6	1-14 tod	1.66	1	38	3100	feminino													
15	6.1-10	n„o	1.53	1	41	3300	feminino		11047.55	161.5	46.6	115.5	72	84	140	53	76	39	0.5664	0.5745
16	10	n„o	1.62	1	39	3700	masculin			184.9	123.6	151.5	90	84	225	39	131	312	0.6341	0.5766
9	3.1-6	n„o	1.6	3	41	4300	mascul	30	36467.46	184.9	117.7	134	83	86	223	51	140	147	0.6806	
3	1 ou -	1-14 tod	1.53	10	39	3020	mascul	21												
4	1 ou -	n„o	1.49	5		3500	feminir	20												
6	1 ou -	n„o	1.62	3	38	3180	feminir	15												
12	3.1-6	n„o	1.59	1	37	3020	feminino													
2	1 ou -	n„o	1.55	4	37	3700	mascul	14	23534.28	181.8	90.7	113	72.5	97	165	52	91	133	0.5944	
5	1.1-3	1-14 par	1.55	2		3700	feminir	23	40302.59	167.1	100.7	122.5	85.5	90	155	56	90	67	0.5772	0.5781
1	1 ou -	15 + tod	1.7	10		2940	feminir	94	28456.47	176	74.2	99.5	61.5	83	189	55	112	106	0.5788	0.5797
13	10	n„o	1.61	4	40	3640	feminir	24												
8	3.1-6	n„o	1.5	2	39	3350	feminir	18												
9	10	n„o	1.5	3	38	4100	mascul	78												
3	1 ou -	15 + tod	1.67	1		2830	masculin		20139.96	184.5	81.4	133.5	82.5	85	169	46	113	52		
8	1.1-3	1-14 par	1.49	1	40	2700	masculin		20855.84	167.55	76.6	115	74	78	178	46	107	166		0.5799
5	1 ou -	15 + tod	1.49	5		3000	mascul	12	16436.55	172.25	69.1	119.5	73	110	232	51	148	184	0.5775	0.5799
9	6.1-10	n„o	1.62	1	38	3250	feminino													
6	1.1-3	1-14 tod	1.56	1		2830	feminino		27885.33	159	66.5	111	70	81	209	58	138	66	0.5795	0.5925
5	1.1-3	1-14 tod	1.6	10		3400	mascul	66	34110.59	166.1	106.1	120.5	95	86	165	34	119	58	0.5811	0.597
0	1 ou -	1-14 tod	1.53	10	38	2800	feminir	69		154.3									0.5769	0.5593

pescmae	prenda	pfumomae	paltmae	pgesta	pidgest	ppn	psex	pint	dmgtotdxa	daltura2012	dpeso	dsysmed	ddiamed	dglicose	dcolesterol	dhdl	dldl	dtrig	dECMICE	dECMICD
3	1.1-3	n„o	1.5	2	39	3350	mascul	19	27553.51	169.3	86.9	123.5	64.5	116	129	30	58	227	0.5775	0.5779
6	1.1-3	1-14 tod	1.56	1	42	3500	feminino		25718.97	160.95	65.9	100.5	64	64	140	55	71	74		
11	1.1-3	1-14 tod	1.55	1	41	3540	masculin		48777.81	173.85	116.8	131.5	84	94	229	55	145	151		
4	1 ou -	1-14 tod	1.48	1	38	2500	feminino		26206.99	162.7	70.8	109.5	75	92	159	46	100	91		0.6212
9	6.1-10	n„o		1	40	3200	masculin		27259.27	173.7	87.5	148	96.5	99	259	52	139	355	0.5739	0.5764
12	1.1-3	n„o	1.63	3	40	3100	mascul	10												
0	1 ou -	1-14 tod	1.55	2		950	masculin													
0	1 ou -	1-14 tod	1.53	1	39	2900	masculin		27504.82	181.5	92	139	76	91	170	37	115	99	0.6068	0.5836
8	1.1-3	1-14 tod	1.66	3		2950	feminir	17												
5	1.1-3	1-14 tod	1.58	5	38	3500	mascul	40	3337.433	164.5	48	125	79	82	189	65	114	57	0.5659	0.5679
4	1 ou -	n„o	1.53	1	39	3780	feminino		38206.95	162.4	92.8	116.5	76.5	91	178	65	92	82	0.5785	0.5778
4	1.1-3	n„o	1.58	2		3200	feminir	11												
3	1.1-3	n„o	1.56	3	39	3750	feminir	65	29761.23	161.75	69.5	105.5	74.5	82	243	54	154	196		
11	6.1-10	n„o	1.6	1	38	3170	feminino		21394.95	163.45	58.3	93	58	97	172	61	70	383	0.5718	0.5728
5	1.1-3	n„o	1.7	3	42	3400	feminir	66	24163.25	169.5	65.5	113	77	92	250	75	158	66		
3	1.1-3	n„o	1.57	1	40	3670	feminino		17000.46	166.6	58.6	96.5	58	70	164	63	89	53	0.5783	0.5813
7	6.1-10	n„o	1.59	1	39	2450	masculin		13281.46	178.15	68	104.5	69.5	99	228	61	129	174		
5	1.1-3	n„o	1.6	7	40	3000	mascul	209	14144.42	185	70.4	142.5	86	123	122	31	68	79	0.5758	0.5773
15	1.1-3	n„o	1.43	1	43	2980	masculin		18830.99	177.8	78	123.5	65	84	155	35	85	211	0.5775	
12	3.1-6	n„o	1.59	2	38	3500	feminir	59	32428.29	161.05	77.4	101.5	71	60	147	51	75	118	0.5751	0.5837
16	3.1-6	n„o	1.66	2	39	3500	mascul	54												
12	3.1-6	n„o	1.63	3	39	4150	mascul	73												
11	1.1-3	1-14 tod	1.54	1	37	2700	feminino													
6	3.1-6	1-14 tod	1.61	2	40	2980	feminir	34	26109.7	163.5	62.3	99.5	67	86	273	106	142	91	0.5777	0.5813
7	3.1-6	n„o	1.52	3	40	3050	feminir	71	18514.59	157.5	53.3	116.5	75	100	216	85	110	70	0.5776	0.578
6	3.1-6	n„o	1.65	1	40	3580	masculin													
16	10	n„o	1.45	5	40	2800	feminir	34												
8	1 ou -	n„o	1.52	4	38	3500	mascul	46												
6	1 ou -	n„o	1.4	3	41	3020	mascul	26												
5	1.1-3	n„o	1.58	1	39	3950	feminino		40122.7	158	81.8	114	74.5	89	212	63	131	104	0.6133	0.5795
2	1 ou -	1-14 tod	1.51	9	39	3000	feminir	14												
4	3.1-6	1-14 tod	1.54	1		3500	masculin		9526.609	176.65	75.4	123	73.5	88	144	34	77	171	0.5806	0.5751
12	6.1-10	n„o	1.56	3	39	2840	feminir	16	20881.54	165.95	61.1	114	72.5	92	197	78	84	168	0.5779	0.578
8	3.1-6	n„o	1.56	3	39	2980	masculin													
11	3.1-6	1-14 par	1.65	1	40	3370	feminino													
12	1.1-3	n„o	1.61	2	40	3800	masculin													
0	1.1-3	n„o	1.59	11		2360	feminir	56	22076.32	165	69.3	99	62	56	151	57	80	56	0.561	0.5322
7	1 ou -	n„o	1.39	2	39	3400	mascul	23	3043.562	150.7	40	128.5	81	111	174	57	103	62	0.5812	0.5947
7	1 ou -	n„o	1.55	1	36	2950	feminino		28801.12	155.8	65.7	122.5	78	146	210	37	114	319	0.5791	0.5775
12	6.1-10	1-14 tod	1.55	2	39	3570	feminir	42	35439.1	161.6	78.1	119	84	84	201	53	123	222	0.5826	
7	1.1-3	n„o	1.53	1	38	2870	feminino													
7	1.1-3	n„o	1.48	3	39	3100	mascul	45	17444.88	168.8	72.8	120.5	66	89	168	49	98	101	0.5799	0.5606
9	1 ou -	n„o	1.53	2	41	3650	mascul	75	18113	171.1	70.8	127	75.5	83	202	58	125	97	0.5786	0.597
4	1 ou -	1-14 tod	1.48	2	40	2500	mascul	46	12900.77	165.1	60.1	119.5	76	104	235	41	152	274	0.606	0.5995
8	3.1-6	n„o	1.6	1	42	2300	feminino		21636.2	158.25	55.5	109.5	78.5	79	175	62	100	80	0.573	0.5516
7	1 ou -	1-14 tod	1.57	1		1250	masculin													
2	1 ou -	1-14 tod	1.56	3	38	3360	mascul	24												
7	3.1-6	n„o	1.62	2	35	2800	mascul	36												

pescmae	prenda	pfumomae	paltmae	pgesta	pidgest	ppn	psex	pint	dmgtotdxa	daltura2012	dpeso	dsysmed	ddiamed	dglicose	dcolesterol	dhdl	dldl	dtrig	dECMICE	dECMICD
4	1.1-3	n„o	1.54	4		2870	feminir	13	38665.13	159.2	84	119.5	80	82	175	62	98	72	0.579	0.5746
3	1.1-3	n„o	1.49	10		3480	feminir	20	13262.97	161.85	54.2	99.5	56.5	74	140	34	95	57	0.5761	0.5569
16	3.1-6	n„o	1.6	2	38	2850	feminir	124	19913.17	155.7	56.7	116.5	65	61	168	74	73	89	0.5649	0.5737
5	1.1-3	n„o	1.6	3	38	3600	mascul	16	8627.242	179	70.6	137	90.5	75	147	51	80	79	0.5663	0.5772
5	1 ou -	n„o	1.67	2	39	3950	feminir	106												
5	1 ou -	n„o	1.57	5		3300	feminir	16												
7	3.1-6	n„o	1.51	2		3070	mascul	38	3930.644	158.6	48.9	119	75	70	192	93	92	36	0.5663	0.5524
16	6.1-10	n„o	1.59	2	38	3700	mascul	68	11736.78	179.6	66.4	124	64	69	250	65	161	113	0.5775	0.5479
14	10	n„o	1.56	5	34	1780	feminir	116												
2	1 ou -	15 + tod	1.56	1	41	3230	feminino		48554.79	176.05	103.9	116.5	74	81	162	61	74	101	0.5756	
16	6.1-10	n„o	1.61	1	38	3780	masculin		37798.53	181.4	112.5	140	81	89	219	53	126	220	0.5808	0.5776
5	1.1-3	1-14 tod	1.55	7	42	3650	mascul	32	30786.59	178.6	91.3	130	90	94	186	49	112	149		0.5787
5	3.1-6	n„o	1.58	1	41	3570	masculin		24507.01	176.7	81.6	126.5	84.5	66	208	52	121	186	0.5778	0.6113
12	6.1-10	n„o	1.5	4			mascul	108												
8	1.1-3	n„o	1.56	2	40	3120	mascul	54												
4	1 ou -	n„o		1		3270	feminino		25137.48	173.15	71.7	109.5	68	58	165	62	95	51	0.5771	0.5629
5	1.1-3	n„o	1.55	1	41	3500	feminino		15488.13	165.5	60.5	121.5	78	99	130	58	55	65	0.578	0.5767
5	1 ou -	1-14 tod	1.56	2	35	2780	feminir	15	40178.6	157.8	83.8	141	99.5	87	190	74	97	98	0.5684	0.5783
16	6.1-10	15 + tod	1.58	2	37	3300	feminir	30												
2	1 ou -	1-14 tod	1.49	3		3560	mascul	34	18203.33	171.6	77	120.5	77	100	229	79	131	65	0.5413	0.579
4	1.1-3	1-14 tod	1.52	2	40	3380	mascul	22												
0	1 ou -	n„o	1.5	1	40	3370	feminino		20960.39	162.4	64.3	114	76.5	79	200	108	68	92	0.578	0.5825
5	1.1-3	1-14 tod	1.66	3	40	3240	mascul	13												
4	1.1-3	15 + tod	1.52	2	27	600	mascul	18												
12	1.1-3	n„o	1.58	3	41	4120	feminir	18												
6	1.1-3	1-14 tod	1.54	2	38	3400	feminir	27												
6	1.1-3	n„o	1.61	1	38	3110	masculin		26977.88	174.5	88.9	143.5	84	78	232	63	118	209	0.5927	0.5842
5	1.1-3	1-14 par	1.57	1	42	3450	masculin													
4	1.1-3	n„o	1.57	2	40	3600	mascul	18	25386.15	176.05	83.8	139.5	84.5	91	275	53	171	259	0.5921	
5	3.1-6	n„o	1.53	2	37	2950	feminir	69						79	155	47	99	39		
6	1.1-3	1-14 par	1.63	1	39	3480	masculin		32922.04	177.25	95.4	143	90	75	166	53	98	92	0.5798	0.5815
4	1 ou -	n„o	1.62	1		4160	masculin													
5	1 ou -	1-14 par	1.51	3	42	3460	feminir	67												
3	3.1-6	n„o	1.51	3		2920	feminir	79												
17	1.1-3	1-14 par	1.57	1	41	2770	masculin		41566.94	173.45	103.4	123.5	76	111	180	36	106	239	0.6162	0.6307
5	1 ou -	n„o	1.48	1	39	3460	feminino													
13	3.1-6	1-14 tod	1.58	2	43	3360	feminir	12	24598.69	166.1	66	124	75.5	97	154	74	69	58	0.5964	0.5817
9	3.1-6	n„o	1.58	2	40	3640	feminir	61	47606.46	159.9	96.6	124.5	82	78	241	63	164	84	0.6088	0.5954
16	10	n„o	1.52	2	39	3100	feminir	58	12044.18	156.1	48.5	101	66.5	64	169	67	79	119	0.5753	0.5705
7	1 ou -	n„o	1.45	3	39	3050	feminir	75	23706.04	156.55	62.3	108.5	69	64	125	52	69	32		
16	10	n„o	1.62	3	41	3100	feminir	12												
15	6.1-10	n„o	1.61	2	39	3800	mascul	28	32657.18	183.9	102.6	143.5	83						0.579	0.5781
9	1.1-3	n„o	1.63	1	38	3160	masculin													
17	10	n„o		3	40	3480	mascul	75												
0	1.1-3	n„o	1.45	3		3300	feminir	20												
4	1 ou -	n„o	1.53	1	40	3330	masculin													
3	1 ou -	n„o	1.44	4		2680	mascul	13												
5	3.1-6	n„o	1.52	2	37	2480	feminir	32												

pescmae	prenda	pfumomae	paltmae	pgesta	pidgest	ppn	psex	pint	dmgtotdxa	daltura2012	dpeso	dsysmed	ddiamed	dglicose	dcolesterol	dhdl	dldl	dtrig	dECMICE	dECMICD
16	3.1-6	n„o	1.54	1	40	3250	feminino													
13	3.1-6	n„o	1.62	7		3130	mascul	16	2757.182	180.25	61	133.5	88.5	78	229	56	147	145	0.5772	0.578
11	10	15 + tod	1.56	2	38	3080	mascul	26												
5	1.1-3	n„o	1.55	2	41	2850	feminir	34												
4	1.1-3	n„o	1.52	2	40	2780	mascul	27	10918.46	160.9	60.5	109.5	64.5	76	155	41	100	101	0.5789	0.5782
4	1 ou -	n„o	1.44	1	41	3150	masculin		17389.33	155.1	59.3	136.5	79.5	95	224	72	138	96	0.5786	0.586
10	1.1-3	n„o	1.61	1	38	3700	feminino		39045.27	168.1	83.3	114	81	79	200	79	101	116	0.5814	0.5792
5	1.1-3	n„o	1.49	1	41	3230	masculin		11420.26	169.75	64	115.5	64.5	103	191	38	116	194	0.5785	0.5916
5	3.1-6	n„o	1.55	5	38	3510	mascul	232												
4	1 ou -	n„o	1.55	2	39	2400	mascul	23												
5	3.1-6	n„o	1.67	3	40	3100	mascul	68	3765.641	169.3	57.7	116	64.5	75	136	52	64	91	0.5698	0.571
4	1 ou -	n„o	1.53	4		3170	feminir	28		160.2	51.8	108.5	73	53	162	56	92	48		
1	10	1-14 tod	1.62	3	39	3150	mascul	64	5489.26	174.7	63.4	110	72.5	71	189	62	102	155	0.5759	0.5788
8	1.1-3	1-14 par	1.51	1	40	3400	feminino													
8	1.1-3	1-14 tod	1.57	2		3600	feminir	14	17598.04	159	51.9	106	59.5	84	168	74	79	68	0.5781	0.578
12	6.1-10	n„o	1.65	2	37	2130	feminir	12	20241.78	157.35	60.9	122	77	81	245	97	133	66	0.5781	0.575
7	1.1-3	n„o	1.59	3	38	2770	mascul	25												
18	1.1-3	n„o	1.52	1	41	3200	feminino		24707.18	161.4	58.8	121	81	103	268	72	146	308	0.5821	0.579
7	1.1-3	1-14 tod	1.51	1		3070	feminino		41618.42	157	96	153	102	72	232	65	145	115		
1	3.1-6	n„o	1.62	1	40	3860	feminino													
8	1 ou -	n„o	1.54	1	37	3000	masculin		11169.97	181.35	79.8	122	59.5	92	150	50	84	58	0.5781	0.5781
3	1.1-3	n„o	1.57	2	39	3630	feminir	117												
11	6.1-10	n„o	1.61	2	40	3960	mascul	84	26797.69	175.85	96.3	127	74.5	180	271	31	100	528	0.7144	0.627
11	1 ou -	n„o	1.7	1		3360	masculin		35128.56	185.2	104.9	140.5	91	84	226	47	155	114	0.5735	0.5768
11	1.1-3	n„o	1.62	2		3800	masculin		18857.01	174.25	80.6	109	67	99	204	51	137	81	0.5641	0.5721
4	1 ou -	1-14 tod	1.52	2	41	3300	mascul	23	12696.01	176.65	71.3	134.5	78.5	73	132	55	70	40	0.5508	0.5745
4	3.1-6	n„o	1.56	2	38	3100	feminir	25												
0	1 ou -	n„o	1.61	2	39	2820	feminir	75	20241.47	160.1	57.9	118.5	74	98	202	49	144	55	0.578	0.5786
6	1.1-3	n„o	1.55	2	38	3150	feminir	60	24278.54	153.9	57.4	108	68	106	265	83	162	113	0.5782	0.5777
9	1.1-3	1-14 tod	1.55	3	32	1170	feminir	12												
5	1.1-3	n„o	1.53	4	38	3250	mascul	84	7034.887	182.9	67.1	128	74.5	71	140	43	83	56	0.5802	0.5696
11	1 ou -	1-14 par	1.52	4	30	1470	mascul	28												
5	1.1-3	1-14 tod	1.65	1		1100	masculin													
4	3.1-6	1-14 tod	1.57	2	37	2550	feminir	14	25972.72	159.8	64.8	126.5	79.5	98	227	76	129	82	0.5793	0.5762
1	3.1-6	1-14 tod	1.6	2	40	3600	mascul	29												
7	3.1-6	1-14 tod	1.67	1	43	3900	feminino		32235.65	159.55	69.2	101.5	63.5	88	158	60	86	63	0.5761	0.578
16	10	n„o	1.63	1	40	3220	masculin		22851	175.75	78.2	134	84.5	96	190	63	97	144	0.5757	
5	1.1-3	n„o	1.58	5	41	4750	masculin		4165.359	179.2	64.4	131.5	86	79	148	70	66	48	0.5789	0.5784
0	1 ou -	1-14 tod	1.5	8	37	2640	mascul	48												
7	3.1-6	n„o	1.54	1	43	2500	feminino													
4	3.1-6	1-14 tod	1.43	1	38	2600	masculin		11901.48	161.4	57.7	104	62.5	93	228	68	134	124	0.5763	0.5746
6	1.1-3	n„o	1.51	1	40	3700	feminino													
2	1 ou -	n„o	1.41	5	40	3430	mascul	21												
3	1.1-3	1-14 tod	1.51	1		2500	feminino		28822.14	150.6	65.6	121.5	88.5	88	161	50	93	89		
8	3.1-6	n„o	1.57	1	36	2820	masculin		31645.32	167.15	83.7	135	93.5	98	189	45	109	235	0.5967	0.5836
6	1.1-3	n„o	1.54	2	38	3400	mascul	41		175.05	58.3	130.5	78							0.5761
1	1 ou -	1-14 tod	1.65	3		4000	mascul	24	8982.853	168.9	63.2	118.5	70	117	206	65	127	59	0.5782	0.5784
2	1.1-3	n„o	1.58	3	39	3000	feminir	55	33300.99	162.55	73.9	110	72	84	130	52	56	100		

pescmae	prenda	pfumomae	paltmae	pgesta	pidgest	ppn	psex	pint	dmgtotdxa	daltura2012	dpeso	dsysmed	ddiamed	dglicose	dcolesterol	dhdl	dldl	dtrig	dECMICE	dECMICD
9	1.1-3	n„o	1.67	1	40	2670	feminino		16832.75	158.95	52.8	97.5	62.5	93	200	47	135	75	0.5787	0.5795
5	1 ou -	n„o	1.5	1	39	3070	feminino		41097.17	155.55	86.9	142	91.5	80	233	75	141	87	0.578	0.5821
0	1.1-3	n„o	1.5	2	39	3200	feminir	11	11762.45	161.65	53.2	112.5	66	74	167	62	87	61	0.5778	0.5717
9	6.1-10	n„o	1.5	3	38	2880	mascul	13	17721.32	172.15	67.5	117	71.5	62	210	70	120	74		
7	1.1-3	n„o	1.6	1	41	2730	masculin		13803.07	173.1	77.7	108	64	71	170	52	106	85	0.5763	0.5751
7	3.1-6	n„o	1.6	1	41	2920	feminino		21373.1	155.35	57.3	114.5	68.5	103	207	65	120	107	0.5803	0.5746
3	1 ou -	n„o	1.57	4	42	2990	feminir	36												
3	1.1-3	n„o	1.56	2	37	2200	feminir	13	17721.34	154.85	54.8	136	92	83	202	50	134	75	0.5785	0.578
3	1.1-3	15 + tod	1.53	3	41	3100	feminir	96	46737.97	151.55	93.5	129	79	80	235	57	148	182		
3	1 ou -	1-14 tod	1.59	1	38	3230	feminino		28261.66	155.7	61.8	113	70.5	101	202	62	133	67	0.5693	0.5785
6	1.1-3	1-14 tod	1.52	6	40	3040	feminir	47	8375.75	148.65	44.2	96	63.5	87	153	31	70	175	0.5805	0.5811
16	10	n„o	1.57	2	41	3360	feminir	39	19715.12	156.55	56.7	111	70.5	68	248	79	145	114	0.578	0.578
12	3.1-6	1-14 tod	1.57	1	38	4200	feminino													
4	1 ou -	n„o	1.57	2		3300	feminir	13		163.8	128.5	139.5	79	96	127	36	61	144		0.6239
9	1 ou -	1-14 tod	1.73	3	41	4090	mascul	26	5162.979	182.3	69.9	146	76	86	131	37	80	49	0.5793	0.5656
3	1.1-3	n„o	1.55	4	39	3880	mascul	12	17471.13	178.3	81	114.5	71	99	207	44	129	232	0.5988	0.6017
3	1 ou -	n„o	1.52	4	39	3500	mascul	118												
5	1 ou -	15 + tod	1.51	1		2000	feminino		24056.42	165.3	61.6	117.5	71	76	189	71	109	59		0.577
2	1.1-3	1-14 par	1.65	2	41	3540	mascul	75	32126.91	178.6	106.4	138.5	68.5	83	194	45	132	124	0.5777	0.576
8	1.1-3	1-14 par	1.67	1	38	2890	masculin		8651.815	181	75.1	120	71.5	88	183	63	107	40	0.578	0.5769
11	1.1-3	n„o	1.65	2		3150	feminir	49												
1	1.1-3	n„o	1.53	8	40	3900	mascul	15	5345.603	174.45	66.3	110	59.5	88	170	56	90	83	0.5823	0.5785
7	3.1-6	n„o	1.56	2	41	2850	feminir	14	64228.45	161.8	109.4	140.5	99.5	81	162	47	99	89	0.5879	0.5977
10	1.1-3	n„o	1.54	1		3400	feminino													
4	3.1-6	1-14 par	1.54	4	38	3000	feminir	94												
5	1.1-3	n„o	1.62	6	39	3640	feminir	87		164.55	94.8	140	83.5	89	166	58	81	152		
16	3.1-6	n„o	1.6	1	39	3700	masculin													
15	6.1-10	1-14 tod	1.59	1	40	3100	masculin		32194.26	176.45	91.4	138	88						0.5787	0.5779
4	1.1-3	n„o	1.61	2	38	3700	feminir	62	44818.71	167.8	98.1	116	75.5	45	163	42	86	125	0.5791	
7	1.1-3	1-14 tod	1.58	1	40	2930	masculin													
7	1.1-3	1-14 tod	1.52	4	40	3100	feminir	35	42394.08	155	89.6	131.5	81.5	93	189	56	112	116	0.5801	0.6253
5	1 ou -	n„o	1.49	3	39	2740	feminir	28	15941.26	163.2	61.4	104	66.5	74	179	62	103	57	0.5781	0.5745
3	1 ou -	n„o	1.47	2	39	3220	feminir	23	30322.78	159.65	72.5	110.5	77	63	190	63	112	70	0.5779	0.5778
4	1.1-3	n„o	1.57	2	41	2970	feminir	30												
4	1.1-3	n„o	1.55	4		4160	mascul	29												
5	1.1-3	n„o	1.53	3	42	2850	mascul	10	18008.22	163.3	75.9	129	76	96	249	50	134	289	0.5794	0.5771
0	1.1-3	15 + tod	1.53	3		3150	mascul	54	28549.33	179.2	98.8	135	88	101	255	48	135	430	0.5772	0.579
7	1 ou -	n„o	1.54	2	37	3400	mascul	16	15927.19	178.4	76.3	129.5	85	83	154	39	100	102	0.5527	0.5818
4	1.1-3	15 + tod	1.6	2		2550	mascul	14	25294.18	171.3	84.2	121.5	79.5	73	209	49	137	108	0.573	0.5781
7	3.1-6	n„o	1.64	2	42	4050	mascul	31		192.2	117	150.5	87	95	153	41	86	117	0.578	0.5904
15	10	n„o	1.55	3	38	3340	mascul	84	34469.56	176.35	108.2	147	91	114	240	63	142	206	0.5812	0.578
6	1 ou -	n„o	1.55	1	41	3200	feminino		55690.61	165.6	115.9	126.5	84	92	162	54	86	138	0.5785	0.5717
7	1.1-3	1-14 par	1.54	1	38	2910	feminino		24429.01	158.95	64.2	111	68.5	82	205	53	135	80	0.5826	
4	1.1-3	n„o	1.5	2	41	3900	mascul	61	30442.33	171.55	88.9	128	89.5	96	251	50	139	365	0.5927	0.6025
3	1.1-3	n„o	1.54	7	40	3520	mascul	103												
9	1.1-3	n„o	1.5	1	41	3200	masculin													
3		n„o	1.51	1		3150	feminino			161.15		96	60.5						0.5772	0.578
0	1 ou -	15 + tod	1.64	8		3520	mascul	46	36011.37	175.75	97	116	74.5	87	247	64	164	93	0.5778	0.5771

pescmae	prenda	pfumomae	paltmae	pgesta	pidgest	ppn	psex	pint	dmgtotdxa	daltura2012	dpeso	dsysmed	ddiamed	dglicose	dcolesterol	dhdl	dldl	dtrig	dECMICE	dECMICD
10	6.1-10	n„o	1.57	3	40	3100	mascul	22	20651.32	170.3	81.7	125.5	86	97	265	82	162	93	0.5857	0.5813
6	3.1-6	15 + tod	1.47	2	38	2800	feminir	73	18833.94	156.5	60.5	101	63	86	183	58	110	86	0.5783	0.5773
3	1 ou -	n„o	1.52	2	39	3950	feminir	15												
4	1.1-3	1-14 tod	1.61	3		3200	mascul	38	21131.56	177	95	133	77						0.6365	0.7289
5	1.1-3	n„o	1.58	3	39	3350	mascul	33	8768.22	175.3	66	126.5	70	64	152	53	89	58		
5	1.1-3	15 + tod	1.64	8	36	3530	mascul	98	20763.04	184.1	84.3	139	89.5	89	150	52	87	54	0.5743	0.561
5	1.1-3	n„o	1.52	1	38	2950	masculin		27786.35	169.6	84	120.5	70	90	164	63	79	87	0.5846	0.5774
5	1.1-3	1-14 tod	1.57	6	39	2820	mascul	11												
3	1.1-3	n„o	1.69	5	42	3550	feminir	12												
12	3.1-6	n„o	1.59	1	40	3650	masculin		24208.49	182.5	83.8	111.5	65.5	97	200	70	113	77	0.5765	0.5793
6	1.1-3	n„o	1.58	1		3650	feminino													
12	3.1-6	n„o	1.54	1	39	3500	feminino		20009.97	163.35	56	113.5	73.5	80	215	75	108	175	0.5782	0.5784
7	1 ou -	n„o	1.5	1	39	2310	feminino													
2	1.1-3	n„o	1.59	2	38	3950	mascul	26												
3	1.1-3	n„o	1.56	2	38	3700	mascul	25	29687.22	181.1	103	131.5	82	93	220	45	133	170	0.5799	0.6018
5	1.1-3	n„o	1.46	1	41	3050	feminino		26124.33	158.7	69.7	114	76	69	138	35	91	82	0.5784	0.5781
1	1.1-3	n„o	1.6	10	39	4300	mascul	43	23468.73	179	81.4	134	77.5	80	193	53	119	101	0.5686	0.5745
12	10	n„o	1.68	1	41	3550	masculin													
8	3.1-6	1-14 tod	1.57	3		2790	mascul	26	19185.09	180.4	79.7	124.5	76	96	224	65	132	144		
8	3.1-6	1-14 par	1.68	2	41	3100	feminir	57												
3	6.1-10	n„o	1.55	3	40	3840	mascul	36												
8	3.1-6	15 + tod	1.53	3	41	3360	feminir	14	10245.14	162.8	48.2	98.5	68.5	85	191	84	89	41	0.5731	0.5711
8	1 ou -	n„o	1.64	1	40	3760	masculin		24810.46	175.6	85.7	138.5	86.5	107	200	51	116	163	0.5781	
0	1 ou -	15 + par	1.6	10		3680	feminir	13	20494.98	157.65	55.7	94	61	88	183	65	85	169	0.5772	0.5783
4	1.1-3	15 + tod	1.58	1		2840	masculin													
3	1 ou -	n„o	1.69	1		3550	feminino													
6	3.1-6	15 + tod	1.7	2	38	2730	feminir	36	26416.66	161.4	67.6	118.5	84.5	81	220	60	153	55	0.5809	0.5758
2	1 ou -	1-14 tod	1.46	1	42	3610	masculin		21100.18	170.2	76.7	114.5	72.5	101	208	56	134	99	0.6008	0.5821
10	1.1-3	n„o	1.53	1		3400	feminino		17396.97	160.75	57.9	125	86	72	200	68	120	77		
3	1.1-3	n„o	1.54	2	40	3140	feminir	93												
9	6.1-10	n„o	1.48	2		3040	feminir	67												
18	10	n„o	1.62	2	35	3560	feminir	54	14546.43	162.7	54.1	98	66	99	188	65	105	92	0.5742	0.5725
4	1 ou -	15 + tod	1.5	1	40	1960	masculin													
5	1.1-3	1-14 tod	1.49	1	42	3050	feminino		40815.18	159.25	85.6	118	77.5	88	190	60	106	155	0.5785	0.5791
5	1.1-3	1-14 tod	1.57	2	38	3040	feminir	17	20219.29	152.2	58	116.5	74.5	91	165	36	98	186	0.5781	0.578
4	1.1-3	n„o	1.57	1	34	2100	masculin													
4	1.1-3	n„o	1.55	3	40	3200	feminir	17												
10	10	n„o	1.6	5	39	3550	mascul	98	33284.35	167.65	93.6	116	80.5	81	236	43	152	219	0.5742	0.5781
6	3.1-6	1-14 tod	1.5	2		2100	mascul	32	29383.56	171.4	86.9	124.5	75	76	220	62	134	147	0.6142	0.5781
1	1.1-3	1-14 par	1.56	2	41	3710	mascul	25	6068.579	173.6	55	129	78.5	92	157	56	88	68	0.5792	0.5746
4	1.1-3	1-14 tod	1.65	2	40	2800	feminir	18		164.95		112.5	69.5						0.5772	0.5816
17	3.1-6	n„o	1.5	1		2700	masculin													
11	1.1-3	n„o	1.56	2		2780	masculin													
5	1 ou -	1-14 tod	1.51	1	41	2810	masculin		36536.62	176.55	92.6	138	92	109	251	46	156	234	0.5782	0.5815
12	6.1-10	n„o		4	39	3070	feminino													
0	1 ou -	15 + tod	1.52	2		2100	feminir	28												
4	1.1-3	n„o	1.49	2	42	3400	feminir	74	17530.19	158	56.2	109	73.5	64	200	58	121	80	0.5791	0.5783
1	1 ou -	n„o	1.55	6	41	3070	feminir	24	12100.11	154.3	47.6	133	89.5	74	158	60	86	42		0.578

pescmae	prenda	pfumomae	paltmae	pgesta	pidgest	ppn	psex	pint	dmgtotdxa	daltura2012	dpeso	dsysmed	ddiamed	dglicose	dcolesterol	dhdl	dldl	dtrig	dECMICE	dECMICD
5	1.1-3	1-14 tod	1.61	2	38	2360	feminir	22												
10	3.1-6	n„o	1.55	1		3950	masculin		18602.69	172	75.2	128.5	76	65	159	55	95	53	0.5782	0.5792
5	1 ou -	1-14 par	1.57	1	38	3050	feminino													
6	3.1-6	1-14 tod	1.5	3	41	3400	mascul	29												
4	1 ou -	1-14 tod	1.51	2	37	2680	mascul	16	20031.51	165.4	72.4	125.5	78	97	234	44	153	190	0.5706	0.5774
4	1.1-3	n„o	1.59	1		3200	feminino		33744.1	163.15	74.2	110.5	65	81	189	62	112	77		0.5742
7	1.1-3	n„o	1.56	2	36	3800	mascul	50	17944.64	175.35	82	114.5	64.5	73	170	50	99	114	0.5776	0.5786
8	1.1-3	n„o	1.55	2		4300	mascul	24	19404.44	170.35	74.3	116.5	68.5	67	175	58	93	122		
4	3.1-6	n„o	1.64	1	38	2680	masculin													
12	3.1-6	1-14 tod	1.58	2	40	3800	mascul	41	15458.29	175.25	81.6	133.5	72.5	84	165	32	106	179	0.5907	0.5841
2	1 ou -	15 + tod	1.49	1	40	2950	feminino		41311.47	154.9	85.4	115.5	79.5	82	168	47	106	115	0.5688	
0	1.1-3	n„o	1.5	9		3320	mascul	73	12101.92	167.1	69.1	124.5	72	96	190	44	115	135	0.6155	0.5794
15	1.1-3	1-14 par	1.63	1	38	2550	feminino		17032.27	166.5	53.2	109.5	66.5	93	204	78	116	45	0.5772	0.5785
4	1.1-3	1-14 par	1.56	2	41	2700	mascul	41	17567.71	176.4	77.3	134	87	86	223	56	125	277	0.5804	0.5783
5		15 + par	1.7	1	38	2890	feminino		29114.12	165.65	69.7	124	86	87	170	53	106	102	0.5878	0.578
6	1.1-3	n„o	1.56	1	37	3500	feminino		12827.7	165.3	59.2	146.5	82.5	99	189	80	100	48	0.5777	0.5792
5	1.1-3	1-14 tod	1.6	1	38	2800	feminino		21704.3	159.5	58.5	124	80	89	191	66	112	52	0.5833	0.6635
3	1.1-3	n„o	1.52	2	43	3000	mascul	36	17062.26	161.65	69.7	115	77	90	152	36	79	207	0.5738	0.5781
5	1 ou -	1-14 tod	1.55	6	38	3300	feminir	43	41998.04	166.4	80.8	103.5	71.5	94	170	70	88	77	0.56	0.58
3	1.1-3	n„o	1.44	8	40	3670	mascul	16	21569.19	161.7	74.6	123	59.5	91	159	36	97	126		0.5664
6	1 ou -	1-14 tod	1.69	1	42	3380	feminino													
5	1.1-3	n„o	1.58	2	39	3350	mascul	122	39538.58	169	97.4	124	77.5	96	148	61	74	57	0.5775	0.5782
5	3.1-6	n„o	1.62	4		3640	mascul	20												
6	1.1-3	1-14 tod	1.29	1		2850	feminino		45858.44	145.8	90.4	123	80	93	277	54	172	243		
9	1.1-3	n„o	1.55	3		2850	feminir	33												
5	1.1-3	15 + tod	1.63	2	41	3650	feminir	23	16319.67	161.4	57.5	103.5	63	123	213	65	138	66		0.5682
7	1.1-3	n„o	1.54	2	39	3100	feminir	62												
8	6.1-10	n„o	1.55	1	39	3080	feminino		24078.08	159.8	60.9	111	70	82	192	79	93	110	0.5502	0.5696
7	1.1-3	1-14 tod	1.6	1	43	3600	feminino		18822.11	164.2	54.1	107.5	68.5	116	289	79	186	125	0.5772	0.5812
8	3.1-6	n„o	1.57	5		3380	feminir	81	34484.03	170	77	135	91.5	103	295	92	161	253	0.5814	
8	1.1-3	n„o	1.45	1	28	700	masculin													
5	1.1-3	1-14 tod		1	38	2930	feminino		52986.69	168.85	115.2	139.5	81.5	286	230	54	162	95		
3	1 ou -	1-14 par	1.56	5		4200	mascul	41	31452.89	178.8	99.2	132.5	81.5	90	132	40	76	84		
11	1.1-3	n„o	1.62	1	41	4160	masculin		42885.47	179.75	117.4	141	77	99	188	36	94	287	0.5839	0.5801
5	1.1-3	15 + tod	1.51	1	39	2750	masculin													
11	1.1-3	n„o	1.65	1	42	3700	masculin		16252.37	174	73.5	117	68	90	192	67	112	61	0.5957	0.5736
4	3.1-6	n„o	1.58	2		3750	mascul	33	23492.23	171.8	74.1	116	75	102	282	65	173	238	0.5745	0.5778
5	1.1-3	n„o	1.58	1	40	3500	masculin		9028.374	168.3	55.3	146	89	95	190	75	102	70	0.628	0.5799
16	6.1-10	n„o	1.5	3	37	2400	feminir	62	23678.95	150.95	59.5	117.5	76.5	88	200	62	107	140	0.5826	0.5786
16	3.1-6	n„o	1.64	1	39	4080	feminino		39807.09	158.9	79	109.5	71	85	177	44	116	83		
6	1.1-3	n„o	1.56	2	38	3200	feminir	56	41150.69	162.25	84.3	119.5	76	90	202	74	105	152	0.5847	0.5798
0	1 ou -	n„o	1.63	1	41	2850	feminino													
4	1.1-3	n„o	1.57	3		3600	mascul	76												
2	1 ou -	n„o	1.5	4	39	3300	feminir	55												
8	3.1-6	n„o	1.46	1	40	3360	masculin		43918.44	176.75	101.9	115	79	89	201	60	121	99	0.5792	
5	6.1-10	n„o	1.61	3		3900	feminir	72	24739.87	159.85	66.6	111	66.5	128	165	50	106	53	0.6166	0.5843
2	1 ou -	15 + tod	1.59	3		3450	mascul	19												
11	10	n„o	1.59	3	38	3350	feminir	59	38702.87	166.8	87.2	126	87	82	157	66	71	82	0.5605	0.5774

pescmae	prenda	pfumomae	paltmae	pgesta	pidgest	ppn	psex	pint	dmgtotdxa	daltura2012	dpeso	dsysmed	ddiamed	dglicose	dcolesterol	dhdl	dldl	dtrig	dECMICE	dECMICD
4	1.1-3	1-14 par	1.54	2	28	1170	mascul	14												
6	1.1-3	n„o	1.61	4	38	4100	feminir	30	33053.3	169.85	75.7	113.5	67.5	83	163	58	97	38	0.5862	0.6187
7	3.1-6	n„o	1.65	2	39	4050	mascul	38		179.6	127.3	141	85	99	157	62	89	66	0.6004	0.661
6	3.1-6	n„o	1.55	1		3150	masculin		41941.86	181.65	110.2	153	88.5	116	183	51	97	196	0.6146	
4	1.1-3	n„o	1.58	1	39	4200	masculin		33571.16	185.05	98.2	136.5	85	75	146	60	78	76	0.5737	
5	1.1-3	1-14 tod	1.56	1	36	2660	masculin		22254.31	174.4	85.3	149	82	103	183	39	106	167	0.5797	0.5835
4	1 ou -	15 + tod	1.55	1	41	2500	feminino		28873.23	150.2	64.5	116.5	82	94	169	57	102	53	0.5892	0.5784
21	3.1-6	n„o	1.54	1	39	3930	feminino		54257.34	156.5	108.9	151	98	85	220	57	138	197		0.5844
7	1.1-3	n„o	1.61	2	37	3180	mascul	14	7380.392	171.75	67.1	127	61	81	200	48	123	137	0.578	0.5777
5	3.1-6	n„o	1.56	8	40	3850	mascul	42	15121.39	167.85	67.1	112	72.5	80	183	67	94	96	0.5652	0.5778
7	1.1-3	n„o	1.52	3		2800	feminir	41	28279.17	156.1	61.1	121.5	79	84	176	55	110	63		0.5698
7	1.1-3	n„o	1.59	1	42	3590	feminino													
16	3.1-6	n„o	1.63	1	40	2980	feminino		22391.2	160.55	63.1	124	83	84	189	69	110	42	0.5757	0.5792
5	1 ou -	n„o	1.56	5	40	2850	mascul	41	13036.01	166.85	72.5	126.5	73.5	132	167	46	80	169	0.578	0.5781
4	3.1-6	1-14 tod	1.62	2	41	3650	feminir	30	14957.79	162.25	60.8	110.5	60.5	79	172	72	94	45	0.5701	0.5801
4	1.1-3	1-14 tod	1.6	5	43	3680	feminir	72	37386.68	162.8	81.8	107	76.5	81	163	83	64	75	0.5782	0.5809
10	1 ou -	n„o	1.49	1	37	3250	feminino		20234.51	156.7	55.7	103.5	70.5	80	147	55	75	79	0.557	0.5629
8	3.1-6	15 + tod	1.6	4	38	3150	mascul	177												
13	3.1-6	n„o	1.62	2	36	2870	feminir	13		170.8		124	76.5						0.5817	0.6311
1	1.1-3	n„o	1.5	2		3050	mascul	59												
6	3.1-6	n„o	1.44	1	39	3370	feminino		15004.15	153.3	48.3	104.5	67	80	150	65	72	49		
0	1.1-3	n„o	1.51	8	38	3130	feminir	59		156.75	63.3	87	55	82	244	48	175	94	0.5786	0.5785
9	1 ou -	1-14 par	1.53	4	43	2530	feminir	26												
13	3.1-6	n„o	1.5	2	40	3540	mascul	30												
3	1 ou -	1-14 tod	1.51	10	39	2850	mascul	189	34068.04	161.8	88.6	127.5	85	114	217	43	149	151	0.5879	
3	1 ou -	15 + tod	1.59	3	39	3030	feminir	47	18593.3	153.25	58.5	116.5	69	87	139	53	76	66	0.5781	0.5753
3	1.1-3	15 + tod	1.62	2	39	3450	mascul	48												
5	1 ou -	1-14 tod	1.58	1	39	2570	masculin													
7	3.1-6	15 + tod	1.53	1	35	2160	masculin		4238.699	165.25	51.2	115.5	69	91	136	57	63	90		0.58
5	3.1-6	n„o	1.57	8	37	3500	feminir	137	18796.29	169.55	56.6	123.5	76.5	85	215	84	104	100	0.5787	0.5592
17	6.1-10	n„o	1.57	2	40	3300	mascul	21	19458.94	175.9	74.7	118.5	69.5	83	173	43	115	65	0.5775	
16	1.1-3	n„o	1.64	1	42	4300	masculin													
4	1.1-3	n„o	1.59	1		2970	masculin		37155.7	173.45	91.2	117	70.5	96	272	53	185	154	0.5793	0.5797
4	1.1-3	n„o	1.69	1		2700	feminino		39962.8	176.3	88.1	118	68.5	96	149	58	82	52	0.5775	0.5752
4	1 ou -	1-14 tod	1.53	1		2150	masculin													
12	3.1-6	n„o	1.51	2	38	3100	feminir	28	23577.63	161	61.5	126.5	79	110	236	80	135	147	0.5594	0.5781
8	1.1-3	n„o	1.52	2	40	3320	mascul	36	19805.43	171.95	74.6	108	66.5						0.5745	0.5974
11	1 ou -	n„o	1.53	1	39	4000	masculin		25088.96	169	81.7	108	71	92	174	36	95	214	0.5945	0.5718
1	1 ou -	n„o	1.51	3	36	2150	mascul	74												
13	1.1-3	15 + tod	1.59	1	38	3270	feminino		17194.03	160.15	54.9	102.5	74	79	189	65	110	56	0.5728	0.6142
5	1.1-3	n„o	1.54	5	37	2520	feminir	34	23744.5	158.8	62.7	119.5	71	83	210	46	139	121	0.6003	0.5813
5	1.1-3	n„o	1.69	2	40	2530	feminir	39												
8	1.1-3	1-14 tod	1.54	1	40	3250	masculin													
6	1.1-3	1-14 par	1.62	1	40	2310	feminino			160.15	81.4	138	78	84	178	56	107	92	0.5853	0.5925
7	1.1-3	1-14 tod	1.53	2	38	2930	feminir	31	19161	162.9	56.4	103	59	65	155	64	74	65	0.5816	0.5782
1	3.1-6	n„o	1.51	4		2850	feminir	45	13953.05	163.9	50.9	114.5	72.5	73	143	88	40	58	0.5774	0.577
0	1 ou -	n„o	1.51	4		3300	feminino		17204.43	159.1	55.2	118.5	75	80	161	60	86	50	0.5839	0.578
4	1 ou -	1-14 tod	1.5	3		2650	mascul	83												

pescmae	prenda	pfumomae	paltmae	pgesta	pidgest	ppn	psex	pint	dmgtotdxa	daltura2012	dpeso	dsysmed	ddiamed	dglicose	dcolesterol	dhdl	dldl	dtrig	dECMICE	dECMICD
4	1.1-3	n,,o	1.61	1	39	3220	masculin		25579.86	172.35	79.4	111	72	73	155	45	86	122		0.5771
12	10	n,,o	1.56	3	38	3200	mascul	63												
7	1.1-3	n,,o	1.6	2	40	4100	feminir	68												
18	10	15 + tod	1.57	2	39	2810	feminir	47		160									0.5781	
3	1 ou -	1-14 tod	1.56	1	36	2760	feminino													
11	1.1-3	n,,o	1.55	1	41	3000	feminino		24139.78	153.35	62.1	115	71.5	88	162	42	102	76	0.5778	0.5715
9	3.1-6	n,,o	1.59	1	39	3430	feminino			169.7	150.1			115	202	70	115	99	0.5913	0.5941
16	1.1-3	n,,o	1.61	1	40	3820	feminino													
16	10	n,,o	1.68	4	39	3000	feminir	15	29681.79	173.6	66.4	101	67.5	95	154	56	88	77		0.5767
4	1 ou -	15 + tod	1.5	4	40	3400	feminir	30												
4	1 ou -	1-14 par	1.55	3	38	3140	feminir	32	12855.12	162.7	51.6	120	73	88	169	47	106	77	0.5782	0.5765
5	1.1-3	n,,o	1.54	4	39	3650	mascul	47												
4	1.1-3	n,,o	1.66	4	40	3420	mascul	18	18704.95	179.65	79.1	119	72	82	197	49	113	254	0.5792	0.5786
1	1 ou -	1-14 tod	1.55	2		2600	feminino													
3	1.1-3	n,,o	1.52	2	39	3720	mascul	84												
10	1.1-3	n,,o	1.55	1		2100	feminino		29597.8	161.9	65.5	110	69.5	85	202	53	137	66		
9	1.1-3	1-14 tod	1.6	4	41	3050	mascul	11												
4	1.1-3	n,,o	1.62	6	42	4920	feminir	20												
12	6.1-10	1-14 tod	1.55	1	41	3380	masculin		22917.78	175.9	80.5	129.5	83	80	190	47	102	200	0.5797	0.5793
4	3.1-6	1-14 tod	1.63	1	39	3760	masculin		24065.34	175.25	83.4	129	74.5	72	186	85	88	58	0.5781	0.5773
16	6.1-10	n,,o	1.72	4		4350	feminir	15												
4	1.1-3	1-14 tod	1.53	11	41	3450	feminir	191	29041.5	162.75	77	99.5	60.5	94	213	76	118	90	0.5783	0.5783
5	1 ou -	1-14 tod	1.54	1	36	3400	masculin													
6	1 ou -	n,,o	1.51	1	42	3700	masculin													
8	1 ou -	n,,o	1.53	1	40	3960	masculin													
10	6.1-10	n,,o	1.67	1	39	3920	masculin		11818.03	172.5	65.6	134	60.5	88	170	55	101	59	0.6862	0.7069
4	1 ou -	1-14 tod	1.56	4		3990	feminir	44												
4	1.1-3	1-14 par	1.55	2		3000	mascul	12												
6		n,,o	1.6	1		1170	feminino		28107.18	156.55	66.4	100.5	67.5	84	155	49	94	43		
7	1.1-3	n,,o	1.51	5		3330	mascul	12												
1	1 ou -	n,,o	1.5	2		2640	feminir	14												
6	1.1-3	1-14 par	1.57	6	39	4000	mascul	36												
16	10	n,,o	1.53	3	39	3630	mascul	88	14240.45	181.35	80.2	127	84	72	192	64	113	85	0.5797	0.578
4	1.1-3	n,,o	1.49	2		3500	mascul	40	23823.8	179	85.8	135	76.5	95	228	74	127	110	0.5757	0.5801
0	1.1-3	n,,o	1.54	8	40	2620	mascul	66	14898.6	175.85	72.7	123.5	74.5	94	190	75	104	68	0.5795	0.578
4	1.1-3	1-14 tod	1.53	3	41	3400	feminir	92												
7	1.1-3	n,,o	1.54	1	37	2680	masculin													
5	1.1-3	15 + tod	1.58	6	37	3200	mascul	18												
5	1.1-3	n,,o	1.49	1	40	2900	feminino													
7	1.1-3	n,,o	1.53	1		2630	feminino		22741.32	154.1	56.6	106	70	86	218	73	130	118	0.5761	0.5782
5	1.1-3	n,,o	1.6	2	40	2900	feminir	86												
5	1.1-3	n,,o	1.49	1	38	3150	masculin		17600.87	166.3	64.7	125.5	80.5	79	279	80	161	195	0.5888	0.5794
5	1 ou -	n,,o	1.47	1	41	3240	masculin		25350.48	172.65	75	117	79.5	85	208	53	133	98	0.5738	0.5801
16	10	n,,o	1.61	2	38	3200	mascul	24												
7	1.1-3	1-14 tod	1.52	4	42	3180	mascul	48	9139.607	170.65	74.3	114	61.5	88	172	78	69	131	0.5783	0.5786
2	1 ou -	1-14 tod	1.52	3	40	2750	mascul	41												
5	1 ou -	n,,o	1.56	2	41	3000	mascul	104												
6	1.1-3	n,,o	1.49	5	42	3730	feminir	24	27249.08	151.3	64.4	118	80	78	216	82	114	71	0.5479	0.5752

pescmae	prenda	pfumomae	paltmae	pgesta	pidgest	ppn	psex	pint	dmgtotdxa	daltura2012	dpeso	dsysmed	ddiamed	dglicose	dcolesterol	dhdl	dldl	dtrig	dECMICE	dECMICD
3	1.1-3	1-14 tod	1.61	4	39	2860	feminir	39	28415.03	161.7	65.7	100	59.5	91	165	58	74	65	0.5776	0.5764
19	10	n„o	1.54	1	42	3460	feminino													
14	3.1-6	n„o	1.58	5	40	3360	mascul	20												
10	1.1-3	n„o	1.61	2		3800	mascul	27	4927.056	184.35	69.4	145	77.5	91	161	61	87	60	0.5701	0.5771
3	1.1-3	n„o	1.44	2	38	2750	feminir	50	25539.03	160.5	69.7	100	57	66	174	79	86	62	0.578	0.5777
5	1.1-3	n„o	1.47	3	41	4160	mascul	85	22464.48	168.4	72.8	128	78	80	219	57	144	91	0.5648	
3	3.1-6	15 + tod	1.55	2		3750	masculin													
12	10	n„o	1.64	2		3780	mascul	18	21748.75	176.55	86.9	143.5	93.5	101	144	33	59	293	0.6388	0.5796
13	3.1-6	n„o	1.6	2	42	3180	mascul	27												
17	10	1-14 tod	1.56	1	36	1700	masculin		14928.14	168.3	76.3	113.5	58.5	79	156	61	81	87	0.5781	0.5777
2	1.1-3	n„o	1.54	4	40	3200	mascul	11												
5	1 ou -	1-14 tod	1.62	2	40	2830	feminino													
9	10	n„o	1.59	2	40	2850	feminir	15												
17	10	n„o	1.55	2	38	2150	feminir	15	17149.02	156.85	51	125.5	71	108	206	62	115	179	0.5814	0.6123
2	1 ou -	n„o	1.61	2	39	3050	feminir	31												
3	1 ou -	15 + tod	1.58	4	39	3950	feminir	15												
12	6.1-10	n„o	1.62	2	41	3320	feminir	28		167.45		98	65.5						0.578	0.5924
16	10	n„o	1.49	3	39	3580	mascul	78	13043.44	168.05	67.7	116.5	62	79	181	49	118	87	0.5708	0.5779
5	1 ou -	n„o	1.42	1		3360	masculin													
17	3.1-6	n„o	1.67	1	41	3300	feminino		46952.73	173.2	97.5	113	76	86	165	51	85	156	0.577	0.5767
0	1.1-3	1-14 tod	1.63	3		3580	feminir	12	23861.7	152.95	62.2	118	76	76	165	65	84	66		
5	1.1-3	1-14 tod	1.55	3	43	3540	mascul	26												
5	1.1-3	n„o	1.66	2	38	3560	feminir	51												
13	3.1-6	n„o	1.65	3		3650	feminir	49	23582.3	174.4	67.1	99	64	64	176	60	103	112	0.5793	0.5824
4	1 ou -	n„o	1.6	1	36	3220	masculin													
5	1 ou -	1-14 tod	1.54	3		3800	mascul	32												
4	1 ou -	1-14 tod	1.56	1		2930	masculin		26701.9	161.6	99.1	131.5	76	102	286	70	185	120		
15	10	n„o	1.55	1	41	3820	masculin		19254.45	176.6	78.2	118	73.5						0.5465	0.5964
3	1.1-3	15 + tod	1.46	2		3380	feminir	46		158.6		98.5	60						0.5723	0.5677
5	1 ou -	n„o	1.56	3	40	3280	feminir	11	36517.09	170.2	88.4	112	59.5	85	143	72	58	47	0.5972	0.5787
6	1.1-3	1-14 tod	1.56	6		2500	mascul	14												
16	6.1-10	1-14 par	1.59	3	39	3900	feminir	18												
3	1.1-3	n„o	1.63	3		3770	feminir	73	42827.24	169.3	88.4	108.5	67	70	204	64	127	74	0.5786	0.5871
15	3.1-6	n„o	1.74	1	39	3680	feminino		27557.66	175.5	75	122.5	84.5	83	144	53	75	80	0.5751	0.5757
5	3.1-6	n„o	1.63	8	42	3220	mascul	169	21453.16	175.8	83.1	129	82.5	95	232	65	157	69	0.565	0.5779
7	1.1-3	1-14 tod	1.5	2	39	2540	mascul	31	10504.42	165.5	71.2	119.5	71.5	75	139	59	64	89	0.6053	0.5783
7	1.1-3	15 + tod	1.58	5	37	2840	mascul	44												
16	6.1-10	n„o	1.58	1	39	3210	feminino													
9	6.1-10	n„o	1.68	3	38	2900	mascul	23												
0	1 ou -	1-14 par	1.51	3	40	4070	mascul	26												
0	1 ou -	n„o	1.59	8	38	3380	feminir	174	13166.66	165	51	119.5	86.5	81	164	52	102	41	0.578	0.5802
10	3.1-6	1-14 tod	1.53	2	41	2850	feminir	16												
2	1.1-3	n„o	1.53	1	41	2500	masculin													
9	6.1-10	n„o	1.66	4	40	4610	mascul	42												
5	1.1-3	n„o	1.6	8	41	3050	mascul	13	16926.72	164.8	75.9	124.5	63.5	81	294	63	202	133	0.6671	0.5812
16	6.1-10	n„o	1.51	3	40	3150	mascul	66	37643.93	168.9	107.3	133.5	80	129	197	63	102	445		
8	3.1-6	n„o	1.61	2	40	3560	mascul	51	18121.54	160.85	65.7	120	70.5	96	155	53	90	50	0.5782	0.5762
5	1.1-3	1-14 tod	1.54	1	42	3630	feminino													

pescmae	prenda	pfumomae	paltmae	pgesta	pidgest	ppn	psex	pint	dmgtotdxa	daltura2012	dpeso	dsysmed	ddiamed	dglicose	dcolesterol	dhdl	dldl	dtrig	dECMICE	dECMICD
0	1 ou -	15 + tod	1.57	5	39	3180	feminir	74	33968.09	157.1	86.3	130	86	128	272	45	178	333		
12	1.1-3	n„o	1.62	1	39	3100	masculin		27164.98	178.15	89.2	150.5	93	102	211	40	112	339	0.5836	0.6387
7	1.1-3	1-14 tod	1.67	2	38	2140	feminir	59												
7	10	n„o	1.59	5	40	4100	feminir	44												
6	1 ou -	1-14 tod	1.5	1	40	2640	masculin		14702.78	159.35	63	111	72	263	235	37	156	202	0.5781	0.5809
16	10	1-14 tod	1.62	3	39	3200	mascul	12												
0	1.1-3	1-14 par	1.68	3	42	3510	mascul	16	31049.92	182.55	105.6	122.5	77	87	179	47	112	105		
2	1 ou -	1-14 tod	1.48	1	37	2700	feminino		13033.32	159.75	55.6	134.5	87.5	69	240	74	150	73		
2	1.1-3	n„o	1.52	7	41	3550	feminir	142	7051.363	159.95	43.3	114.5	72	76	130	62	60	51	0.5738	0.5773
5	3.1-6	15 + tod	1.52	2		3250	mascul	19	26203.81	176.1	81	124.5	75	108	199	53	112	186	0.554	0.5768
7	1 ou -	1-14 par	1.54	1	39	3500	masculin		27245.14	180.95	98.3	131	80	91	192	38	114	193		
6	1.1-3	n„o	1.58	1		3630	feminino		52731.34	161.7	107	114	81.5	111	128	44	54	167		
1	1.1-3	n„o	1.49	1	42	3050	masculin		15768.16	178.55	66.6	133.5	81	96	153	57	76	51	0.5772	0.5737
5	1 ou -	1-14 tod	1.55	1	40	3470	masculin		17382.98	177	80.4	117.5	70.5	88	208	47	134	237	0.5799	0.5747
5	1.1-3	n„o	1.5	1	40	3300	feminino													
11	10	n„o	1.62	2	42	3450	mascul	25												
6	1 ou -	n„o	1.56	3	40	2700	feminir	12												
5	1.1-3	15 + tod	1.5	2	39	2950	mascul	49												
3	1.1-3	n„o	1.52	1	40	2750	masculin		23242.19	165.9	78.2	118.5	78.5	95	216	42	115	389	0.578	0.579
12	3.1-6	n„o	1.51	1	39	2700	masculin													
5	1 ou -	1-14 tod	1.46	1		2320	masculin		10428.65	162.35	64.2	123	68	81	135	52	70	49	0.5774	0.584
0	1.1-3	n„o	1.48	10		3360	mascul	41												
7	1.1-3	1-14 tod	1.53	3	37	3200	feminir	31	25269.73	160.1	66.2	116.5	79	66	185	63	115	58	0.5794	0.5961
5	1 ou -	n„o	1.51	2	41	2970	feminir	15	19222.04	157.3	57	112.5	76.5	109	208	57	121	176	0.5735	0.5826
12	3.1-6	n„o	1.61	2	37	3115	mascul	21	23585.04	177.1	92.3	127	80.5	83	168	50	108	64	0.5603	0.5849
4	1.1-3	n„o	1.54	2		3680	mascul	35												
4	1 ou -	n„o	1.5	1		3400	feminino													
5	1 ou -	1-14 tod	1.65	1	43	3570	masculin													
16	10	1-14 par	1.6	1		3000	masculin		28593.6	175.7	85.4	123.5	60	81	209	49	126	181	0.5811	0.5775
5	3.1-6	1-14 tod	1.6	5	38	2600	feminir	180												
4	1 ou -	1-14 tod	1.55	3	41	3720	mascul	62	27419.69	173.05	92.3	125	91.5	80	179	63	100	69	0.7662	0.6098
4	1 ou -	n„o	1.46	4	32	1550	feminir	56	18200.75	155.5	53.3	102.5	61	84	168	63	97	43	0.5579	
4	1.1-3	n„o	1.54	5	41	3660	feminir	15	13557.27	155.8	47.5	99	64	54	208	78	120	75		
5	1 ou -	n„o	1.51	1		2450	masculin		22764.61	185.2	90.4	130	76.5	67	182	46	107	169		
7	1 ou -	n„o	1.58	2	41	2830	feminir	33												
9	1.1-3	15 + tod	1.58	1	40	3650	masculin			181	120.4	144.5	89	101	128	34	67	128		0.5701
7	3.1-6	1-14 par		1	42	3400	feminino													
15	6.1-10	n„o	1.56	2	38	3100	mascul	47	22428.16	174.35	79.7	117	69.5	78	151	53	86	50	0.5797	0.5791
19	3.1-6	n„o	1.57	3	35	2160	feminir	17	41166.69	163.5	86.8	116.5	71.5	82	176	50	102	140		
5	1.1-3	1-14 tod	1.64	2		2800	mascul	36	33731.45	186.8	99.3	124.5	74.5	207	271	32	110	665	0.5785	0.5777
4	1 ou -	n„o	1.56	1	38	2800	feminino		17345.37	157.8	52.6	109.5	70	96	150	44	80	107	0.5591	
5	1.1-3	1-14 par	1.51	2		2850	masculin		20775.86	172.9	78.9	112.5	74.5	92	193	46	93	250	0.5774	
9	1.1-3	n„o	1.65	1	41	3900	masculin													
5	1 ou -	15 + tod	1.59	3	37	3450	mascul	28	29120.4	180.65	91.8	128.5	83.5	73	170	51	112	38	0.5816	0.5775
5	1.1-3	1-14 par	1.61	4	42	3200	mascul	38	9052.713	181.15	66.1	127	67.5	78	153	51	75	132	0.5679	0.5779
5	3.1-6	n„o	1.61	5	40	4220	mascul	18	23219.56	181.15	92.9	136	86	108	238	57	144	188	0.5898	0.6324
15	3.1-6	n„o	1.53	2	38	3450	mascul	98												
8	3.1-6	1-14 par	1.74	1	42	3670	feminino			173.9	126.1	129	84	77	215	58	140	71		

pescmae	prenda	pfumomae	paltmae	pgesta	pidgest	ppn	psex	pint	dmgtotdxa	daltura2012	dpeso	dsysmed	ddiamed	dglicose	dcolesterol	dhdl	dldl	dtrig	dECMICE	dECMICD
0	1.1-3	n„o	1.5	2	42	2950	mascul	45	8438.493	164.8	62.7	123.5	64.5	80	160	56	78	115	0.5785	0.5923
8	1.1-3	n„o	1.55	2	41	2650	mascul	23				123.5	72	80	150	53	79	49		
9	1.1-3	1-14 tod	1.54	5	40	2990	feminir	31	18925.33	165.1	63.6	114	74	81	240	64	148	126	0.6098	0.5815
0	1 ou -	n„o	1.56	15	38	2740	feminir	22	19512.01	167.9	64.3	106.5	66.5	80	182	66	100	81	0.5782	0.5804
5	1 ou -	15 + par	1.71	2	40	3860	mascul	28												
5	1 ou -	1-14 tod	1.55	6	40	3230	mascul	63	14909.97	169.7	70.6	136	81	87	161	65	79	72	0.5784	0.5774
11	1.1-3	1-14 par	1.59	1	38	3200	masculin													
6	1.1-3	1-14 par	1.59	2	38	3250	mascul	34												
2	1.1-3	1-14 tod	1.52	3	38	3250	mascul	36	3557.773	174.1	53.3	111	72	90	168	41	110	40	0.5573	0.5652
8	1 ou -	n„o	1.55	1		3400	masculin		13264.48	174	75.1	118	73.5	91	253	97	133	96	0.5864	0.5841
6	3.1-6	1-14 tod	1.52	4	38	3350	feminir	89	39909.96	159.7	80	120	86.5	107	287	90	173	161	0.5801	0.5849
7	1.1-3	n„o	1.45	2	39	3680	feminir	30												
13	3.1-6	n„o	1.63	1		3380	masculin		22905.03	170.2	71.4	137	85	92	178	56	97	152	0.5728	0.5777
5	1.1-3	1-14 tod	1.56	1	36	3000	feminino		28049.97	166.1	72.4	128	86.5	75	166	65	89	71	0.5737	0.5804
7	1.1-3	n„o	1.59	3	43	2330	feminir	30	52580.29	163.05	116.7	117	74	93	161	41	85	199	0.5992	
5	1 ou -	n„o	1.51	4	40	2800	mascul	63	10593.57	176.2	74	124.5	71.5	87	179	62	101	153	0.577	0.5767
3	1.1-3	n„o	1.59	3	34	1700	feminir	123	62129.69	155.55	119.1	148.5	102	97	182	72	86	167	0.5717	0.5665
6	1.1-3	n„o	1.59	2	42	3550	feminir	46	26746.3	165.45	72.3	115	82.5	98	248	82	147	96		
6	1 ou -	1-14 tod	1.58	2	39	2570	mascul	12												
5	3.1-6	n„o	1.43	1	37	2680	masculin													
8	3.1-6	1-14 par	1.61	1	41	2720	masculin			170.75	109.2									
16	6.1-10	15 + tod	1.64	4	38	2860	mascul	42	32503.91	182.3	103.2	134.5	71	95	213	64	127	188	0.5782	0.6727
7	1.1-3	n„o	1.57	5		3550	mascul	19												
4	1.1-3	n„o	1.53	3		2960	mascul	24												
4	10	n„o	1.65	6	39	3580	mascul	48	23540.43	183.15	85.3	120	69	80	217	42	152	121	0.5696	
0	1.1-3	15 + tod	1.52	1		3040	masculin													
8	6.1-10	n„o	1.59	1	41	3700	feminino		28285.24	169.3	71.1	98.5	63	75	198	63	114	124	0.5597	0.5728
5	1.1-3	1-14 tod	1.57	2		3200	masculin													
9	6.1-10	n„o	1.6	2	38	2760	feminir	61												
8	1.1-3	n„o	1.56	2		1100	feminir	12												
5	1 ou -	n„o	1.58	1	41	3200	masculin													
10	6.1-10	n„o	1.59	1	39	3700	feminino			166.7	67.1	129.5	82.5							
2	1 ou -	1-14 par	1.5	4	38	3130	mascul	21												
9	1.1-3	1-14 par	1.58	2		2860	mascul	119												
12	3.1-6	n„o		1	40	3680	masculin		19812.74	171.75	77.6	114.5	65	79	217	78	125	51	0.5743	0.5786
12	6.1-10	n„o	1.62	3	41	2400	feminir	96												
7	1 ou -	n„o	1.58	2	44	3210	feminir	53	37995.23	161.55	75.6	100.5	64	73	175	70	86	100	0.5785	0.5789
8	3.1-6	1-14 par	1.56	2	38	2750	feminir	49	41644.2	170.1	91.7	124	70.5	90	206	78	114	83	0.5758	0.578
11	1.1-3	15 + tod	1.69	2	41	3400	mascul	33												
7	1.1-3	n„o	1.5	1	42	3580	feminino		39417.7	153.9	78.5	106.5	69	96	199	60	126	70	0.5765	0.5771
3	1.1-3	n„o	1.51	5	39	3800	feminir	47	36412.72	164.7	93.3	118.5	73.5	112	186	45	113	185	0.6358	0.6043
8	1 ou -	n„o	1.54	1	39	3270	feminino		10594	155.7	42.2	98.5	54.5	179	140	83	46	45	0.5782	0.5788
5	3.1-6	15 + tod	1.5	4	39	2500	feminir	88	40533.29	158.8	79.7	117.5	78	101	228	66	145	89	0.5783	0.5781
5	1.1-3	n„o	1.5	2	41	3500	feminir	55	30847.74	156.1	69	117.5	77.5	101	172	66	86	147	0.578	0.5799
3	1.1-3	n„o	1.54	3	38	3920	feminir	28												
5	1.1-3	n„o	1.55	3	39	3450	feminir	111	56942.27	164.8	107.2	115.5	71	97	170	43	98	225		
5	1.1-3	n„o	1.52	2		3330	feminir	61												
7	3.1-6	1-14 tod	1.67	1	39	2900	masculin													

pescmae	prenda	pfumomae	paltmae	pgesta	pidgest	ppn	psex	pint	dmgtotdxa	daltura2012	dpeso	dsysmed	ddiamed	dglicose	dcolesterol	dhdl	dldl	dtrig	dECMICE	dECMICD
8	1.1-3	n„o	1.63	1	41	2840	feminino		18869.6	161.7	56.1	112	69	135	189	63	106	101	0.5783	0.5418
3	1.1-3	n„o	1.55	3	39	3170	feminir	26												
12	6.1-10	n„o	1.67	1	39	3000	masculin													
3	1 ou -	1-14 tod	1.56	8	37	2840	feminir	17	15184.94	162.8	49	106	73	81	220	88	119	58	0.5758	0.5801
11	10	1-14 par	1.59	1	39	3060	feminino													
13	1.1-3	n„o	1.59	4	39	3300	feminir	23												
5	3.1-6	n„o	1.6	2	40	2950	feminir	34	25522.96	167.85	64.5	112.5	72.5	69	169	57	100	67	0.5727	0.5757
5	3.1-6	n„o	1.61	2	38	3220	mascul	59	14796.15	170.15	63	116	73	81	177	47	111	83	0.5902	0.5863
18	6.1-10	n„o	1.54	1	40	3230	masculin		22468.12	171.7	78.9	160.5	102.5	85	187	45	113	144	0.5787	0.5789
0	1.1-3	1-14 tod	1.71	2	40	3240	mascul	129												
2	1.1-3	n„o	1.48	4		2410	feminir	13												
8	1.1-3	n„o	1.63	1		3680	feminino		61281.13	170.3	109.5	123	80.5	72	230	74	114	258	0.5789	0.5771
1	1 ou -	n„o	1.45	1		2650	masculin													
8	1.1-3	n„o	1.54	2	39	3350	mascul	46	37318.7	168	106.3	129.5	83.5	86	142	36	79	149		0.577
1	1.1-3	n„o	1.52	1	37	2480	masculin			178	123.7	125	77	93	173	43	102	146	0.5852	
6	1 ou -	n„o	1.47	2	40	3690	feminir	14	58759	151.3	111.5	153.5	100	94	175	72	95	50		
0	1.1-3	n„o	1.54	2	38	3210	feminir	13	20257.43	161.35	54.1	121	81	80	173	74	87	51	0.5692	0.5537
5	1.1-3	1-14 tod	1.44	1	39	2700	masculin		11834.15	157.75	63.9	127	62.5	86	197	74	115	51	0.5421	0.5729
4	1.1-3	n„o		2	39	3570	mascul	62												
9	3.1-6	n„o	1.55	3	39	3320	feminir	29	33264.04	170.3	71.6	110.5	75	82	160	54	89	65	0.5785	0.5786
3	1.1-3	1-14 tod	1.52	2	40	2900	mascul	24	17249.05	165.5	60.9	105	58.5	84	191	48	114	115	0.5783	0.576
1	1.1-3	15 + tod	1.45	1	42	2940	feminino		25325.63	147.8	60.3	107.5	70.5	79	168	51	102	127	0.5838	0.612
0	1.1-3	n„o	1.67	2	40	3400	mascul	132	22066.49	167.3	78.5	121	79.5	95	178	52	115	76	0.5784	0.5821
17	1.1-3	n„o	1.57	3	37	3500	mascul	37												
3	6.1-10	n„o	1.61	2	40	2950	mascul	17	28642.32	183.5	101.4	132	67.5	84	155	39	94	112	0.5782	0.5783
4	1 ou -	n„o	1.51	4	41	3350	mascul	69												
2	1 ou -	1-14 tod	1.47	1		3020	masculin			174.35	125.3	151	91	136	216	32	122	316	0.6182	0.6207
5	1.1-3	1-14 tod	1.57	4		3450	mascul	11	9218.687	182.75	75.3	116.5	64	86	150	74	65	46	0.5775	0.5717
8		n„o	1.51	2		2690	mascul	27	17097.05	163.6	70.7	130	86	97	214	41	133	209		
18	10	15 + tod	1.63	1	38	3340	feminino													
9	3.1-6	n„o	1.64	1		2300	masculin		33363.13	176.45	92.7	127.5	78.5	82	149	56	83	48	0.5788	0.5781
7	1 ou -	1-14 tod	1.58	1		3070	masculin		12001.7	178.5	67.9	118	65.5	80	180	51	117	45	0.5782	0.5689
3	1.1-3	1-14 tod	1.55	1	41	2570	masculin		44641.39	175.55	104.9	148	104	100	269	55	184	92	0.5808	0.5793
7	1 ou -	n„o	1.58	1		3480	feminino		40816.89	165.5	87.8	107	70.5	76	150	60	81	48	0.5808	
1	1.1-3	n„o	1.57	5	43	3770	mascul	15												
16	3.1-6	n„o	1.68	2	42	3160	feminir	35	18034.91	168.2	59.8	129	81	115	195	61	104	171	0.5782	0.577
8	6.1-10	1-14 par	1.52	4		3200	mascul	118	48426.05	179.15	112.3	122	72.5	79	152	48	87	71	0.5867	0.5808
6	1 ou -	n„o	1.57	1	39	2820	feminino		18347	162.6	61.9	134.5	87	77	187	59	108	99	0.5808	0.5787
3	1 ou -	1-14 tod	1.5	4	40	3710	mascul	33	39265.15	170.9	103.6	127	76	77	170	52	102	48	0.5853	0.5814
12	1.1-3	n„o	1.65	1	37	3000	feminino		17863.87	166.6	60.6	99.5	56.5	85	207	78	107	115	0.5791	0.6026
3	1 ou -	1-14 tod	1.54	2		3020	mascul	58	33927.38	162.6	97.8	129.5	78.5	79	150	38	90	64	0.6426	0.6763
16	10	n„o	1.65	2	36	3400	feminir	23	18768.27	154.1	54.1	103.5	57.5	84	216	72	133	75	0.5733	0.5728
16	1.1-3	n„o	1.69	2	40	3050	mascul	18												
15	10	n„o	1.66	3	39	3340	feminir	95	19305.04	168.55	61.4	121.5	79	78	256	110	135	74	0.5747	0.5818
4	1.1-3	n„o	1.56	2	40	3470	mascul	35	28711.69	174.2	88.1	145.5	91.5	124	206	38	106	272	0.622	0.5922
12	3.1-6	n„o	1.5	2	42	2860	feminir	19	24710.19	156.8	63.2	121.5	78	87	207	80	115	52	0.5684	
7	3.1-6	n„o	1.59	2	39	3350	mascul	14	18155.58	186.45	77.3	124.5	78	109	164	36	87	234	0.5766	0.5767
5	1.1-3	1-14 tod	1.59	1	38	2900	masculin		22601.86	173.8	84.8	129.5	71.5	76	130	48	72	63	0.595	0.6003

pescmae	prenda	pfumomae	paltmae	pgesta	pidgest	ppn	psex	pint	dmgtotdxa	daltura2012	dpeso	dsysmed	ddiamed	dglicose	dcolesterol	dhdl	dldl	dtrig	dECMICE	dECMICD
5		n„o	1.56	5	41	3360	mascul	16	43919.8	177.45	117.4	145.5	87	90	225	62	148	77	0.6061	0.5894
7	3.1-6	n„o	1.57	3		2370	mascul	71												
8	1.1-3	1-14 par	1.64	2	40	4350	mascul	57	19806.22	181.15	85.1	115	68	117	217	35	108	257	0.5651	0.5569
2	1 ou -	n„o	1.47	3	38	3480	masculin													
0	1 ou -	n„o	1.52	6		3610	mascul	62												
6	1 ou -	n„o	1.51	1		2700	masculin													
7	1.1-3	n„o	1.66	1	41	3880	masculin													
12	3.1-6	1-14 tod	1.64	1	38	2800	feminino		21162.61	160.25	52.3	123	82.5	76	160	60	85	79	0.5703	0.5638
3	1.1-3	n„o	1.65	4		2120	feminir	19	15410.01	161.4	55.6	119	79	75	195	70	115	46	0.5784	0.5779
4	1 ou -	n„o	1.62	1	40	2500	feminino													
8	3.1-6	1-14 par	1.56	2	38	2500	mascul	49	3449.839	167.4	71.9	121	60.5	105	166	62	88	69	0.5849	0.6016
16	10	n„o	1.6	3	38	3100	mascul	13	17026.38	182.1	82	135	77	94	241	95	132	74	0.5656	0.5791
6	1.1-3	n„o	1.52	1	41	3360	masculin		15442.1	165.85	71.6	111	66	73	135	48	78	44	0.578	0.5674
2	1.1-3	n„o	1.5	5	38	2780	mascul	43	20238.13	158.5	69.8	121.5	64	56	188	36	128	113	0.5624	0.5804
7	6.1-10	n„o	1.61	2	39	3300	feminino		25646.79	157.7	62.4	105.5	67.5	96	227	96	110	131	0.5676	0.5781
7	1.1-3	1-14 tod	1.57	2	41	2980	feminir	13	23523.15	157.95	62.7	100	67.5	92	206	73	121	60	0.5787	0.5811
1	1.1-3	1-14 par	1.61	1	38	2850	masculin		27939.64	173.6	89.4	128.5	78	103	208	54	126	145	0.594	0.6729
17	10	n„o		3		770	mascul	13												
5	1 ou -	n„o	1.51	3	40	2400	feminir	23	33304.32	157.5	73.6	100	65.5	84	210	52	133	123	0.5633	0.569
8	1.1-3	n„o	1.5	1	40	3950	feminino		18638.46	157.3	53.8	106.5	63	62	176	79	85	64	0.5796	0.528
12	3.1-6	n„o	1.53	1	41	3400	masculin		38360.53	178.55	94.3	123	81.5	97	207	56	140	80	0.5787	0.5676
7	1.1-3	n„o	1.67	4	41	2960	mascul	125		188.75	65.5	138.5	91.5	85	183	67	94	95	0.587	0.5807
1	1.1-3	n„o	1.52	6	41	3170	mascul	72	5530.278	168.4	59.3	114	67.5	72	177	53	111	58	0.5788	0.5792
1	1.1-3	15 + tod	1.53	1	40	3100	feminino													
11	3.1-6	1-14 par	1.61	1	39	2550	masculin													
9	3.1-6	n„o	1.67	2	38	3500	mascul	46	26017.29	175.2	88.2	126	68.5	80	186	73	101	47	0.583	
7	1.1-3	n„o	1.64	4	40	3250	feminir	35	26274.9	168	65.9	101.5	59	81	149	56	76	90	0.5751	0.5666
2	1.1-3	n„o	1.59	8	40	3930	mascul	33	32283.18	186.5	105.5	149.5	84.5	95	236	52	136	249	0.5929	0.5818
6	1 ou -	n„o	1.55	1		2650	feminino													
2	1 ou -	n„o	1.6	1	40	3780	masculin													
5	1.1-3	15 + tod	1.62	6	40	3390	feminir	25												
9	1.1-3	n„o	1.7	2	41	3550	feminir	12												
3	1 ou -	1-14 tod	1.63	2	41	3300	mascul	51												
3	1.1-3	1-14 tod	1.47	2	37	2530	mascul	74												
12	3.1-6	n„o	1.6	1	40	3500	masculin		12870.74	169.35	67	141.5	75.5	102	205	59	103	221	0.57	0.5754
12	3.1-6	1-14 tod	1.46	1	41	3030	feminino													
12	10	n„o	1.6	3	38	3150	feminir	31	13016.81	160.65	48.3	109	74.5	77	175	71	80	105		
4	1 ou -	n„o	1.54	2		2220	feminir	89												
5	3.1-6	1-14 tod	1.63	1		3280	masculin													
6	1.1-3	n„o	1.53	2	41	3280	mascul	22												
6	1 ou -	n„o	1.57	1	39	2890	masculin		25958.16	172.5	79.2	122	79	84	191	46	137	65	0.5821	0.5523
3	1.1-3	n„o	1.61	1	36	3490	masculin		24306.93	179.2	90.2	122	73	96	159	37	76	227	0.5943	0.5925
4	3.1-6	n„o	1.55	1	38	2500	masculin		31686.9	176.05	99.3	123	59	101	171	35	82	317	0.5865	
6	1 ou -	1-14 par	1.62	2		3210	mascul	33												
4	1.1-3	n„o	1.53	3		2930	mascul	56												
3	1 ou -	n„o	1.52	1	40	3500	masculin													
2	1.1-3	1-14 tod	1.53	2		3620	feminir	15	37278.41	165	79.3	123	81.5	78	178	67	98	49	0.5831	0.5806
9	1.1-3	n„o	1.57	2	39	2670	feminir	34	18590.82	163.85	54.9	116	76.5	90	196	56	119	109	0.5747	0.5759

pescmae	prenda	pfumomae	paltmae	pgesta	pidgest	ppn	psex	pint	dmgtotdxa	daltura2012	dpeso	dsysmed	ddiamed	dglicose	dcolesterol	dhdl	dldl	dtrig	dECMICE	dECMICD
0	3.1-6	1-14 tod	1.51	3		4020	mascul	33	17770.83	178.3	76.5	117.5	72.5	132	182	53	109	73	0.5794	0.5665
5	1 ou -	n„o	1.67	7		4230	mascul	40												
6	1.1-3	n„o	1.54	1	39	3060	feminino		40638.95	169	89.4	170	117.5	105	164	47	88	222	0.5949	
4	1.1-3	n„o	1.5	7	39	3650	mascul	53	13523.95	176.75	72.4	126	70	82	195	74	103	78	0.5622	0.5614
0		1-14 tod	1.62	2		3000	mascul	11												
7	1.1-3	1-14 tod	1.53	1		3550	feminino		17923.99	160.9	57.4	105.5	73.5	78	148	55	84	38	0.5692	0.578
0	1 ou -	1-14 par	1.53	4		2600	feminir	89												
7	1.1-3	n„o	1.59	1	38	3000	masculin		25491.88	183.3	86.7	122	76.5	81	199	39	145	63	0.5732	0.5804
9	1.1-3	n„o	1.62	2	39	3400	mascul	47	20766.29	183.05	87.2	114	69.5	87	180	57	109	66	0.5798	0.5743
4	1 ou -	1-14 par		5		3200	feminir	32	23203.29	162.45	60.3	99.5	69.5	91	165	59	92	52	0.5793	0.5789
0	1 ou -	n„o	1.59	5		3540	masculin													
12	3.1-6	n„o	1.65	1	39	3200	feminino													
6	1.1-3	n„o	1.5	1	38	2930	feminino													
4	3.1-6	n„o	1.62	4	39	3600	mascul	19		183.65	149.1	164.5	96.5	96	208	48	117	224		
8	1.1-3	n„o	1.55	6	39	3700	feminir	57	17374.03	158.9	58.5	102.5	53	77	167	45	94	159	0.5777	0.5725
7	3.1-6	15 + tod	1.66	2		3400	feminir	24	41000.49	172.25	90.9	118.5	79.5	97	215	61	131	133	0.5772	0.5773
4	1 ou -	1-14 par	1.53	1	40	2700	masculin													
4	1 ou -	1-14 tod	1.62	1	39	2770	feminino		38688.53	162.95	77.6	130.5	84.5	79	150	74	66	59	0.5828	0.5795
8	1 ou -	1-14 tod	1.63	2	41	3570	mascul	61	15927.77	179.25	77	118.5	60	95	151	54	89	66	0.5672	0.584
3	1 ou -	1-14 tod	1.57	2		3150	mascul	14	22897.81	175.45	87.1	138	83	97	263	51	177	159	0.5842	0.5786
6	1.1-3	n„o	1.5	2		2860	mascul	18												
0	1 ou -	n„o	1.54	4		3200	feminir	142	16881.19	163.4	55.1	113.5	70.5	90	180	59	111	55		0.5741
12	6.1-10	n„o	1.58	2	39	3450	feminir	18	11355.31	162.4	51.6	119	80.5	72	140	63	70	41	0.5648	0.5778
3	1 ou -	15 + tod	1.63	6		2350	feminir	11												
3	3.1-6	1-14 tod	1.67	1	38	3370	feminino		29272.15	180.7	76.6	118	58.5	88	170	75	79	67	0.5785	0.5892
11	1.1-3	n„o	1.6	1	39	3360	masculin		34235.54	174.4	101	123.5	79	87	215	50	134	173	0.5841	0.5762
7	1 ou -	n„o	1.6	1	37	3500	masculin		26544.26	180.6	81.4	117.5	81.5	107	240	40	143	355	0.5781	0.5954
6	1.1-3	n„o	1.59	2	40	3300	feminir	76	48965.23	153.15	95.9	116.5	74	115	306	73	207	217		
11	3.1-6	15 + tod	1.46	2		3070	feminir	32	10736.81	152.6	43.1	97	62.5	80	184	80	93	45	0.5774	0.578
10	1.1-3	n„o	1.53	1	40	3600	feminino		20399.36	164.45	59.8	112.5	73	60	195	95	77	123	0.5789	0.5722
5	1 ou -	n„o	1.58	1	38	2880	masculin		13518.21	166.15	67.9	127.5	81	95	150	54	85	54		
3	3.1-6	n„o	1.64	4	39	3330	feminir	127	18301.66	154.1	54.1	115.5	75.5	92	181	70	100	40		
4	1.1-3	1-14 par	1.6	1	39	3850	masculin		27563.32	185.05	102.5	148.5	86	83	205	45	128	184	0.5781	0.5623
4	1.1-3	1-14 tod	1.52	1	38	3060	feminino		21371.89	158.3	58.4	103.5	71.5	103	214	51	151	93	0.5645	
0	1.1-3	n„o	1.56	20		3360	feminir	20	24317.43	152.6	60.5	101.5	63.5	77	200	79	99	74		
12	1.1-3	n„o	1.65	4	39	2750	feminir	29												
6	3.1-6	n„o	1.58	2	39	3400	feminir	69	46757.66	170.7	90.3	108	71.5	150	283	90	170	137	0.58	0.5616
12	3.1-6	n„o	1.56	2	40	2580	mascul	70	7399.438	170.85	58.3	102	64.5	86	124	60	46	139	0.5831	0.6514
0	1 ou -	n„o	1.51	4		1760	mascul	41												
17	10	n„o	1.61	1	40	3400	masculin													
7	1.1-3	1-14 tod	1.6	2		2450	feminir	11	27379.67	164.3	68.7	105.5	67	87	150	45	88	79	0.5798	0.5708
3	1 ou -	1-14 tod	1.6	2	40	3780	feminir	30	30143.89	168.25	73.1	129	91.5	110	240	75	143	126		
5	1.1-3	1-14 par	1.58	1	41	2970	masculin		34046.63	177.8	93.1	121	82	95	158	51	91	70	0.5781	0.5783
6	3.1-6	1-14 par	1.6	1	33	2200	feminino													
12	6.1-10	n„o	1.57	2	40	3400	feminir	56	55756.3	157.55	113.3	132.5	78.5	77	227	60	146	98	0.5798	
7	3.1-6	n„o	1.6	2	38	3400	mascul	35												
9	1.1-3	n„o	1.64	1	41	4060	masculin													
5	1 ou -	n„o	1.54	3		3440	feminir	10	33882.76	158.65	78.7	116	67	84	190	45	123	110	0.5776	0.579

pescmae	prenda	pfumomae	paltmae	pgesta	pidgest	ppn	psex	pint	dmgtotdxa	daltura2012	dpeso	dsysmed	ddiamed	dglicose	dcolesterol	dhdl	dldl	dtrig	dECMICE	dECMICD
8	1.1-3	n„o	1.55	1		2500	feminino													
3	1.1-3	1-14 tod	1.6	4		3850	feminir	28	23879.98	156.2	61.3	108	62.5	96	189	60	115	77	0.579	0.5737
4	1 ou -	n„o	1.61	2	40	3000	mascul	20												
1	1 ou -	n„o	1.47	3		3320	feminir	59												
16	3.1-6	n„o	1.55	1	40	3430	feminino		17625.34	155.3	58.7	104.5	62.5	98	179	83	76	107	0.5822	0.5772
16	10	n„o	1.7	1	39	3680	masculin													
4	1 ou -	n„o	1.46	4		3100	feminir	61	48689.17	156.3	93.9	128.5	85	134	183	56	92	343	0.58	0.57
16	3.1-6	1-14 par	1.7	1	40	3150	masculin		12710.06	188.75	84.9	127.5	72	87	154	43	104	48	0.5854	0.5787
3	1.1-3	n„o	1.6	2	41	3020	mascul	49	7572.913	173.2	68.3	116	67.5	87	178	52	115	97	0.5814	0.5819
11	6.1-10	1-14 par	1.57	2	41	2970	feminir	67												
5	1.1-3	1-14 tod	1.62	2	38	3200	mascul	30	22700.82	170.75	84.8	152.5	96	134	200	46	80	372	0.5789	0.5807
16	6.1-10	n„o	1.55	3	38	3450	mascul	40	28174.63	176.85	90.4	128	77.5	92	207	48	149	49		
5	1.1-3	n„o		1	39	2670	masculin		19181.01	171.6	89.8	130	81.5	98	175	46	102	174	0.5781	0.6028
6	1 ou -	n„o	1.5	1	39	3470	masculin		57048.01	159.9	116.5	108.5	73	83	229	56	149	124		
5	1 ou -	15 + par	1.56	2	37	2900	mascul	33		177.6	128.5	151.5	94.5	366	491	36	118	809	0.6775	0.708
3	1 ou -	15 + tod	1.5	2		3400	feminir	33	34368.71	166.7	72.7	102.5	71.5	68	160	74	78	39		0.5663
5	1.1-3	15 + tod	1.52	1		3400	masculin													
12	3.1-6	n„o	1.59	2	40	5050	mascul	45	22703.33	183.25	88.2	126	74.5	86	165	50	97	77	0.6186	0.5784
12	3.1-6	1-14 par	1.53	1	28	1430	masculin													
9	3.1-6	n„o	1.64	3		3430	mascul	16	20724.09	177	82.1	128.5	93	90	184	40	109	129	0.5809	0.5782
5	3.1-6	n„o	1.48	4	39	3000	feminir	40												
4	1.1-3	n„o	1.61	2		2000	mascul	35												
8	1.1-3	1-14 tod	1.66	1	41	3600	feminino		37748.89	164.45	81.3	128.5	89	81	238	77	140	110	0.5779	0.5777
4	1 ou -	n„o	1.47	1	40	2600	feminino		12130.06	155.05	48.6	115.5	66.5	81	203	52	132	106	0.5786	0.5788
16	10	n„o	1.55	2	37	3220	mascul	78	44537.69	178.25	94.7	129.5	72	88	232	63	150	87		
1	1.1-3	1-14 tod	1.46	2	35	1900	mascul	64												
6	1.1-3	1-14 par	1.52	9		4000	mascul	11	15012.45	171.3	81.6	122.5	85	83	150	40	77	132	0.5752	0.5653
5	1 ou -	n„o	1.51	5		3630	mascul	17	30442.12	166	83.7	106	74.5	92	170	56	81	167	0.5961	0.5827
5	1 ou -	n„o	1.55	4		3700	feminir	14												
13	10	1-14 tod	1.63	2	38	3300	feminir	141	26700.48	161.6	67.1	117.5	75	88	180	54	110	68	0.5797	0.5803
12	3.1-6	n„o	1.69	1	40	3200	masculin		31601.31	189.45	102.3	138.5	92.5	117	211	46	125	226	0.6106	0.6031
1	1 ou -	1-14 tod	1.56	5	37	2340	mascul	20												
3	1.1-3	15 + tod	1.62	2	39	2800	mascul	68												
4	1 ou -	n„o	1.57	1	39	3130	feminino													
6	1.1-3	1-14 tod	1.64	3	40	3060	mascul	39												
4	1 ou -	n„o	1.56	1	39	2900	masculin													
2	1.1-3	n„o	1.65	2	40	3720	mascul	169												
15	6.1-10	n„o	1.65	1	38	3850	masculin													
1	1.1-3	n„o	1.52	2	42	3490	feminir	47	13411.75	163.6	53.7	119	70	89	132	57	62	53	0.5782	0.5784
0	1.1-3	15 + tod	1.62	9		4000	mascul	23												
3	1 ou -	n„o	1.52	2	41	3400	feminir	27												
2	1.1-3	1-14 tod	1.45	1	41	2550	feminino		30197.63	157.35	70.7	102	68.5	86	247	52	166	116	0.5829	0.5728
8	6.1-10	1-14 tod	1.67	3	40	3760	feminir	21												
8	3.1-6	1-14 tod	1.6	3	40	3600	mascul	73	29969.9	180.3	94.6	115.5	71	101	167	42	104	102	0.5725	0.5809
14	1.1-3	1-14 par	1.55	1	35	2230	masculin													
8	1.1-3	1-14 par	1.56	2	38	3000	mascul	55	31407.95	171.5	83.2	148.5	99.5	85	155	43	90	102	0.6538	0.5897
2	1 ou -	n„o	1.48	1		3300	masculin		3137.688	164.25	56.5	108	61.5	114	200	79	99	84	0.5776	0.5781
6	1.1-3	n„o	1.57	1	40	2950	masculin													

pescmae	prenda	pfumomae	paltmae	pgesta	pidgest	ppn	psex	pint	dmgtotdxa	daltura2012	dpeso	dsysmed	ddiamed	dglicose	dcolesterol	dhdl	dldl	dtrig	dECMICE	dECMICD
5	3.1-6	1-14 tod	1.6	5	40	2100	feminir	119												
4	1.1-3	n„o	1.57	1	41	3300	masculin													
4	1.1-3	1-14 tod	1.5	2	40	2950	feminir	26												
0	1 ou -	1-14 tod	1.48	6	42	2940	feminir	49	34372.41	154	76.2	139	94.5	75	148	57	84	39	0.5758	
5	3.1-6	1-14 tod	1.62	3		3560	feminir	33	42631.79	153.4	80.3	108	73.5	103	242	74	119	270	0.5719	
4	1.1-3	n„o	1.62	4		4500	mascul	127		187.85	104.4	115	74	79	150	35	92	102		
1	1.1-3	n„o	1.63	1	40	3240	masculin													
8	1.1-3	n„o	1.65	1	39	3400	feminino		29486.98	167.2	78	115	78	94	166	53	100	58	0.5866	0.58
2	1 ou -	n„o	1.53	1	39	3000	feminino		20831.15	159.25	63.5	116.5	80.5	68	190	61	106	145	0.537	0.5483
9	1.1-3	n„o	1.61	3	41	3000	feminir	94	36120.71	163.8	88	118.5	69	64	98	22	55	97	0.5796	
11	6.1-10	n„o	1.5	1	41	3140	feminino													
3	1 ou -	n„o	1.58	1	39	2410	feminino		22290.53	156.55	56.1	102	70.5	84	153	55	83	80	0.5787	0.5786
5	3.1-6	n„o	1.5	2	41	3360	mascul	23	22634.93	175.4	82.7	128	80.5	68	140	36	74	146	0.5762	0.5764
4	1.1-3	1-14 par	1.5	4	40	2800	feminir	47	23016.85	156.2	62	110	72	92	218	63	139	89		
8	1.1-3	n„o	1.63	2	40	3080	feminir	63												
9	1.1-3	1-14 par	1.61	1		2080	masculin		19716.32	174.2	90.4	121	68	77	128	32	77	100	0.5785	0.5762
6	1.1-3	1-14 tod	1.56	2	40	3550	mascul	55	18287.89	177.45	78.9	125.5	71	100	205	53	111	252	0.5972	0.5776
8	3.1-6	n„o	1.61	1	40	3490	masculin													
8	1.1-3	15 + tod	1.52	1	41	2900	feminino		39752.04	156.25	85.5	121.5	79.5	96	229	71	113	300	0.5995	0.5797
4	1.1-3	n„o	1.6	1		3870	masculin													
2	1 ou -	n„o	1.57	1		3730	feminino		56409.21	166.45	119.5	215.5	124.5	169	317	51	201	467	0.7033	0.7147
0	1.1-3	1-14 tod	1.59	1		3100	masculin													
4	1.1-3	n„o	1.55	1	39	3310	masculin		32174.74	174.8	99.5	133	71	80	165	45	98	119	0.5696	
12	1.1-3	n„o	1.56	1	38	3150	feminino													
8	1 ou -	n„o	1.62	3	39	3610	feminir	15	16160.48	170.5	61.3	136	87.5	76	162	49	100	71	0.5808	0.5799
0	1 ou -	n„o	1.54	5	38	3320	mascul	67	25612.71	170	80.7	133.5	74.5	109	263	63	168	173	0.5838	0.6036
15	10	n„o	1.46	2	37	3400	mascul	38	15935.87	156.55	61.8	120.5	72.5	75	212	57	139	99	0.6184	0.597
11	3.1-6	1-14 tod	1.58	2	41	3700	feminir	42	54109.3	172.2	106.1	105	72.5	93	260	59	156	213	0.5844	0.578
15	1.1-3	n„o	1.58	1	39	3450	feminino													
7	3.1-6	n„o	1.46	3	39	3200	feminir	27	31934.16	155.45	66.8	100.5	65	75	190	63	111	83	0.5792	0.5818
16	6.1-10	n„o	1.62	2	34	2370	feminir	42												
5	1.1-3	n„o	1.52	3	36	2400	mascul	99	32254.84	173.5	95.5	130	71.5	86	218	51	139	125	0.6167	0.5783
5	1.1-3	n„o	1.7	3	37	3750	mascul	104	37376.04	186.2	99.9	131.5	79	133	327	56	222	424	0.6108	0.6236
0	1 ou -	n„o	1.53	2		3300	mascul	11	24750.15	171.4	85.2	135	84	73	132	38	64	129	0.5732	0.5781
16	6.1-10	n„o	1.57	3	39	3860	mascul	52												
6	1.1-3	n„o	1.56	2		3820	feminir	18	28731.7	167.2	72.5	99.5	60	93	128	38	62	122	0.5756	0.5774
16	6.1-10	n„o	1.56	4	41	3550	mascul	36	29890.97	174.1	84.3	131	82	87	225	58	132	211		0.5811
0	1.1-3	n„o		2	40	3600	mascul	12												
3	3.1-6	n„o	1.67	3	39	3400	mascul	31												
3	1 ou -	1-14 tod	1.45	1	43	3700	masculin													
6	1.1-3	1-14 par	1.6	2	39	3280	masculin													
9	3.1-6	1-14 tod	1.6	1	38	3350	feminino		25159.82	159.5	59.9	132.5	91	83	263	103	88	437	0.5886	0.592
12	6.1-10	n„o	1.47	3	37	2960	mascul	34	13892.12	172.9	65.8	108.5	66	89	178	58	105	71	0.5786	0.578
4	3.1-6	n„o	1.53	2	40	2670	feminir	24												
7	1.1-3	n„o	1.41	5		3380	mascul	24	6699.795	163.25	65.5	107.5	51.5	95	132	64	62	40	0.5783	0.5738
5	1 ou -	1-14 tod	1.59	4	38	2420	feminir	91	38573.81	172.8	84.1	112	76	157	188	58	106	139	0.5775	0.5767
5	1.1-3	n„o	1.51	1	38	2970	masculin													
6	1.1-3	n„o	1.52	1	30	1300	masculin													

pescmae	prenda	pfumomae	paltmae	pgesta	pidgest	ppn	psex	pint	dmgtotdxa	daltura2012	dpeso	dsysmed	ddiamed	dglicose	dcolesterol	dhdl	dldl	dtrig	dECMICE	dECMICD
9	1.1-3	n,,o	1.6	1	41	3830	masculin		14106.82	172.55	70.2	106.5	63	77	159	63	79	75	0.57	0.5788
0	1.1-3	n,,o	1.51	1		3400	feminino													
0	1.1-3	n,,o	1.53	2	38	4220	mascul	18												
7	3.1-6	n,,o	1.64	1	40	2680	masculin		6485.827	181.65	69.9	117.5	73	89	213	61	135	74	0.6394	0.5783
7	3.1-6	n,,o	1.61	1	43	2870	feminino		46839.11	164.9	90.2	123.5	96.5	92	200	70	117	81	0.5857	
13	1.1-3	n,,o	1.44	1		2650	masculin		10638.43	166.45	71.9	136	78.5	108	189	70	91	146	0.5787	0.5774
14	1.1-3	n,,o	1.62	1		4000	feminino		33998.05	165.1	74.1	107	72	82	198	81	99	73	0.5781	0.5778
4	1.1-3	n,,o	1.57	2	39	2580	feminir	15												
6	1.1-3	1-14 par	1.63	1	40	3150	masculin													
2	1 ou -	n,,o	1.37	3	36	2670	feminir	35												
14	3.1-6	n,,o	1.64	2	38	3450	feminir	32	20787.11	166.35	64	107.5	66.5	86	205	76	120	40		0.578
7	1.1-3	15 + tod	1.61	2	40	3800	mascul	40	26880.56	187.05	92.8	118.5	74.5	84	175	50	111	63	0.5804	0.5792
6	3.1-6	n,,o	1.54	1	40	2670	feminino		24673.5	152.8	61.3	104	67	88	260	89	150	115	0.5896	0.5908
15	6.1-10	n,,o	1.63	1	40	3320	masculin		37507.14	183.15	108.2	142	84	77	180	53	114	51	0.5783	0.7157
5	1.1-3	1-14 par	1.57	1		2600	masculin		9496.102	177.15	72.7	121	76.5	84	135	47	78	42	0.5749	0.578
3	1.1-3	n,,o	1.49	1	38	2500	feminino													
5	1.1-3	1-14 tod	1.61	4	38	3800	feminir	39	30472.27	160.65	71.6	131	79	77	211	48	134	130	0.5792	0.5786
2	1.1-3	n,,o	1.49	2	41	3670	masculin		27507.04	173.7	106.2	172.5	107.5	134	192	35	81	446	0.6032	
3	1.1-3	15 + tod	1.5	3	38	3100	mascul	35	23296.73	180.5	88	143	80	101	163	48	102	51	0.6136	0.6034
4	1.1-3	n,,o	1.48	1	38	2100	masculin		24922.24	159.2	72.7	134.5	88	110	180	40	98	255	0.5846	0.578
5	1.1-3	n,,o	1.58	2	40	2760	mascul	70	12830.12	164.8	51.9	109.5	79.5	138	201	25	75	434	0.5783	0.5746
5	3.1-6	1-14 par	1.6	1	40	3340	feminino			165.85		106	60.5						0.5776	0.5787
12	6.1-10	n,,o	1.58	2	40	3150	feminir	37	18208.49	159.9	56.3	106.5	72	92	199	78	112	40	0.5789	0.5792
11	1.1-3	n,,o	1.55	1	38	2830	masculin		31065.56	178.35	90.7	140	89	91	222	64	149	75		0.6155
17	10	n,,o	1.58	3	38	2720	feminir	12												
2	1.1-3	1-14 par	1.52	1		2800	feminino			166.3	100.3	161.5	91						0.5766	0.5709
7	1.1-3	n,,o	1.47	1	42	2700	masculin		17116.97	166.5	62.3	142	93.5	89	133	49	72	48	0.5747	0.5695
1	1 ou -	n,,o	1.54	4	39	3150	mascul	60	10379.41	176.1	76.1	117	65.5	90	145	43	87	73	0.6194	0.5789
3	1.1-3	n,,o	1.54	3	38	2900	feminir	47	11764.38	168.5	48.9	151	98	69	192	88	79	110		
6	1.1-3	1-14 par	1.59	1	41	3250	feminino													
5	6.1-10	n,,o	1.6	3	38	3500	feminir	32												
4	1.1-3	n,,o	1.57	3	40	3020	feminir	11	21109.05	160.3	65.5	105	69.5	81	160	51	98	60	0.5885	0.6174
0	1.1-3	n,,o	1.58	4	42	4100	feminir	66	35579.94	159.6	79.4	115	69.5	102	210	65	93	290	0.5782	0.5744
6	1.1-3	n,,o	1.61	1	39	3300	feminino		20364.37	170.1	57.8	135	89	77	174	65	86	101	0.5807	0.5783
5	1.1-3	n,,o	1.57	1	41	3050	feminino													
10	3.1-6	n,,o	1.53	1	41	3500	masculin		33098.95	170.75	92	128.5	78.5	89	159	55	84	105	0.6245	0.578
9	3.1-6	1-14 tod	1.55	2	40	3330	mascul	37												
15	6.1-10	n,,o	1.63	2	39	3670	feminir	47												
12	3.1-6	1-14 par	1.5	2	38	3190	feminir	14	10912.77	156.45	50.3	106.5	67	97	150	83	58	40	0.5797	0.5776
15	10	n,,o	1.5	1	39	3510	masculin		14716.54	179.1	85.6	156.5	85.5	97	175	43	92	184	0.5717	0.5634
5	1 ou -	n,,o	1.56	3		3820	mascul	19												
5	1.1-3	n,,o	1.51	3	40	3540	feminir	61												
12	10	n,,o	1.58	2	41	3400	feminir	69												
7	1.1-3	n,,o	1.47	1	27	1090	masculin													
6	1 ou -	n,,o	1.54	1		3500	feminino		51580.08	162.9	99.7	106	75	92	162	40	85	189		
3	1.1-3	n,,o	1.57	3		4050	mascul	15												
7	1.1-3	n,,o	1.69	4	38	3550	mascul	46	7803.917	179.45	69.4	114	66.5	62	100	46	38	77	0.5868	0.5783
5	1 ou -	n,,o	1.58	2	37	3300	feminir	21	37557.5	156.55	82.4	118.5	71.5	93	200	58	126	76		

pescmae	prenda	pfumomae	paltmae	pgesta	pidgest	ppn	psex	pint	dmgtotdxa	daltura2012	dpeso	dsysmed	ddiamed	dglicose	dcolesterol	dhdl	dldl	dtrig	dECMICE	dECMICD
0	3.1-6	15 + tod	1.61	4		3060	masculin		9334.46	173.7	78.5	119	53.5	78	142	46	80	83	0.5796	0.5805
4	1.1-3	1-14 tod	1.48	2	40	2570	feminir	11	14730.07	155.65	48.9	108.5	73.5	82	252	74	166	46	0.5549	0.5677
8	3.1-6	n„o	1.44	1	39	2940	feminino		10752.69	158.1	45.4	109.5	74	68	183	70	102	45	0.5669	0.577
16	10	n„o	1.56	5	37	2350	feminir	48												
6	3.1-6	15 + tod	1.46	3	40	2980	mascul	78	42397.46	170.45	108.9	119	73.5	88	210	49	142	88		0.58
8	3.1-6	15 + tod	1.55	6	35	2160	mascul	98												
1	1.1-3	1-14 tod	1.57	1	38	2850	masculin													
3	1 ou -	n„o	1.56	2		3360	feminir	14	20102.38	170.85	60.4	106	67	68	172	39	103	142	0.5821	0.6084
5	1.1-3	n„o	1.68	6	39	3750	feminir	22	16774.26	166.1	62.5	119.5	81	74	206	63	121	113	0.5788	0.5772
3	1.1-3	n„o	1.48	2		2700	mascul	24	22420.79	163.45	74.4	119	84.5	84	209	74	127	45		0.5798
8	3.1-6	1-14 tod	1.56	1		3250	masculin													
0	1 ou -	n„o	1.56	1		2300	feminino													
16	6.1-10	1-14 par	1.58	2	39	3950	feminino													
7	1.1-3	n„o	1.58	1	41	3400	masculin													
11	10	n„o	1.5	2	38	3400	feminir	82	26453.39	153.1	54.9	125.5	86.5	80	290	105	171	84	0.5781	0.5788
7	1.1-3	1-14 par	1.61	3	40	3320	mascul	18												
6	1.1-3	n„o	1.63	1	39	2910	masculin			174.6	132.4	146.5	80.5	117	232	45	156	158	0.6109	0.6029
6	1.1-3	1-14 tod	1.59	1	41	3260	feminino		16728.7	158.95	58.2	112.5	71.5	93	143	45	88	49		0.5777
11	3.1-6	n„o	1.6	1	35	1980	masculin		25027.47	184.95	81.5	125	84	96	180	51	109	90	0.579	0.5826
3	3.1-6	1-14 tod	1.59	3	39	3000	feminir	25	35078.62	157.7	73.7	114	77.5	80	204	79	110	91		
4	1 ou -	n„o	1.58	1	34	1820	feminino													
12	3.1-6	n„o	1.6	1	38	2900	feminino													
3	6.1-10	n„o	1.49	2		3070	mascul	33												
5	1.1-3	15 + tod	1.51	4	31	1600	feminir	29	25130.91	151.5	60.1	128	77.5	84	215	65	140	38	0.5759	0.5748
15	3.1-6	1-14 par	1.63	5	38	2900	mascul	60												
3	1.1-3	n„o	1.42	3	42	3100	feminir	31												
5	6.1-10	n„o	1.61	5	38	5850	mascul	134		190.6	130.3	130	83.5	62.825	165.015	39.79	104	159.1		
6	1.1-3	n„o	1.59	2	38	2550	feminir	14												
5	1.1-3	1-14 tod	1.6	2	39	2500	mascul	15												
3	1.1-3	n„o	1.54	2	40	3550	mascul	34	35566.77	174.5	97.5	127	67	114	180	68	99	59	0.5781	
17	10	n„o	1.61	2	39	3640	feminir	44	33206.59	158.5	73.6	113.5	80	91	193	61	114	447	0.5788	0.578
2	1.1-3	n„o	1.49	2	39	3800	mascul	27	13154.15	160.9	71.4	151	98.5	89	201	59	127	90	0.584	0.697
2	1.1-3	n„o	1.6	1	42	3280	masculin													
12	3.1-6	n„o	1.5	3	41	3310	masculin		40507.04	172.7	96.6	145	88.5	99	201	42	127	154	0.5785	0.5758
9	3.1-6	n„o	1.62	1	39	4720	masculin													
16	10	n„o	1.56	2	39	3750	mascul	48	33060.96	165.7	86.1	115	77	79	198	55	120	105	0.6078	0.5798
7	1 ou -	1-14 tod	1.52	5	36	3600	mascul	33												
3	1 ou -	1-14 tod	1.5	3	34	1950	mascul	24												
7	1.1-3	n„o	1.56	2	37	3560	feminir	15	23709.45	158.6	65.1	106	74.5	80	151	48	93	67	0.5772	0.5613
13	1.1-3	n„o	1.6	2	37	3820	feminir	14	23624.77	163	63.9	123	86	78	207	91	100	77	0.578	0.5785
6	3.1-6	n„o	1.58	2	37	2800	feminir	14	20727.36	167.4	61.4	111.5	70	78	200	80	99	74	0.5788	0.5784
9	3.1-6	n„o	1.52	1		2920	feminino		37442.49	152.45	76.4	143.5	102	78	197	58	123	68		0.5779
6	1 ou -	15 + par	1.53	2	39	2670	mascul	13												
12	10	n„o	1.56	3	38	2950	mascul	63												
5	3.1-6	1-14 tod	1.62	3	41	3000	feminir	17	18693.61	160	59.5	108.5	70.5	96	188	94	87	41		
5	1.1-3	n„o	1.48	1	36	2850	feminino			157.9		106.5	63.5						0.5714	0.5769
12	6.1-10	n„o	1.6	1	41	3300	feminino		35552.21	160.9	76.6	122.5	80	78	186	75	93	102	0.5841	0.5917
3	1.1-3	n„o	1.42	6		3030	mascul	142	28413.16	171.3	92.3	144.5	71.5	74	248	58	157	151	0.6093	0.6045

pescmae	prenda	pfumomae	paltmae	pgesta	pidgest	ppn	psex	pint	dmgtotdxa	daltura2012	dpeso	dsysmed	ddiamed	dglicose	dcolesterol	dhdl	dldl	dtrig	dECMICE	dECMICD
1	1.1-3	n„o	1.64	1	39	2650	feminino		23386.63	170.25	66.8	115.5	81	73	178	74	99	39	0.5781	0.5749
4	1 ou -	n„o	1.69	3		4080	mascul	17												
3	1 ou -	1-14 par		2		3400	feminir	40												
7	1.1-3	1-14 tod	1.61	2	39	3650	feminino					101	77	90	184	54	114	55		
5	1 ou -	n„o	1.55	1	37	2800	feminino		13529.59	160	56.2	121	76.5	84	198	67	109	77	0.5756	
2	1 ou -	n„o	1.51	4		2950	feminir	23												
5	1.1-3	n„o	1.64	4	39	3360	masculin		4448.93	168.55	54.3	134	65	91	189	74	104	48	0.578	0.5883
8	1.1-3	1-14 tod	1.66	1	41	2970	feminino		49035.18	168.1	96.7	112	74	80	113	55	40	83	0.5785	0.5803
9	3.1-6	1-14 par	1.56	1	38	2880	feminino		56293.47	171.05	110.9	117	76	97	258	48	180	159	0.5826	0.5799
4	1.1-3	n„o	1.51	1	39	2980	masculin							85	201	51	122	136		
12	1.1-3	n„o	1.55	1	39	3930	feminino		29949.65	158.5	72.1	140.5	96	71	212	63	121	159	0.5768	
8	1.1-3	n„o	1.53	2	39	3500	mascul	52	5458.063	180.1	58.1	132.5	78.5							
8	1.1-3	1-14 par	1.54	2	40	3050	masculin													
5	1 ou -	1-14 tod	1.58	2	39	3870	mascul	36	17715.94	172.6	78.3	146.5	96	82	142	47	81	97	0.5864	0.5878
11	1.1-3	n„o	1.5	1	36	2450	feminino													
3	1.1-3	n„o	1.52	8	37	3850	feminir	65	50013.82	156.1	100.3	135.5	91.5	74	193	43	130	85	0.5801	0.5722
13	6.1-10	n„o	1.6	1	39	2900	masculin													
5	1.1-3	1-14 tod	1.53	2	40	1950	feminir	43	21284.13	158	59.5	130.5	77.5	83	174	78	81	68	0.5819	0.578
6	1 ou -	n„o	1.57	1	36	3020	masculin		24884.26	169.1	80.6	121	80	83	287	52	221	102	0.5695	
1	1.1-3	n„o	1.5	5	43	3350	mascul	21	27037.43	171.1	94.1	127.5	69.5	107	249	55	137	294	0.587	0.5826
10	1.1-3	n„o	1.55	2		3380	mascul	12	19365.31	176.1	77.6	111.5	61.5	134	151	49	89	64		0.6053
6	1.1-3	1-14 par	1.58	1	40	2650	masculin		24418.37	171.45	80.2	137.5	76.5	91	250	48	148	243	0.5832	0.5863
5	1.1-3	15 + tod	1.58	2	40	2920	feminir	93	14876.34	161.1	52.3	120.5	70	82	190	56	119	71	0.5784	0.5785
3	1 ou -	1-14 tod	1.56	3	41	2930	feminir	24												
15	3.1-6	n„o	1.7	3	41	4250	mascul	30		187.3	104	129.5	71.5	78	165	42	98	118		
2	1 ou -	n„o	1.62	1		3720	feminino													
9	1.1-3	15 + tod	1.7	2	35	2480	mascul	25	20687.71	183.9	103.2	144.5	92.5	55	197	47	124	82	0.5793	0.5813
6	1.1-3	1-14 tod	1.54	3	43	2740	feminir	31	41881.89	160.45	89.5	123.5	77	87	133	51	66	59	0.5853	0.5879
5	1.1-3	n„o	1.66	7	34	2550	feminir	164	37345.66	180.7	86.8	125	77	87	265	73	149	310	0.577	0.5772
6	3.1-6	n„o		5	39	2150	feminir	33	56985.75	160.3	116.7	108	71.5	87	144	40	75	127	0.6003	0.5825
6	1.1-3	1-14 tod	1.48	1	41	2680	feminino		40603.18	161	82.8	111	68	88	106	38	60	39		
6	1.1-3	n„o	1.51	3	39	3150	feminir	26	35762.81	156.25	76.5	105	66.5	74	164	58	90	86	0.5824	0.576
3	1.1-3	n„o	1.57	5	38	4350	mascul	39	15608.78	169.3	68.3	127	76	76	240	57	161	95	0.5772	0.5782
0	1 ou -	n„o	1.51	2	39	3370	feminir	21	24657.57	158.6	68.5	113.5	69.5	87	175	51	110	131	0.5801	
2	1 ou -	n„o	1.51	6	39	2250	mascul	12												
2	1 ou -	n„o	1.49	3	40	3470	mascul	84	18397.24	172.25	73.1	159	86	99	204	74	120	46		
3	1 ou -	1-14 tod	1.59	2	39	3650	mascul	17												
0	1 ou -	n„o	1.5	4	37	1930	masculin													
9	1.1-3	1-14 par	1.52	2	40	3360	mascul	80	7661.578	171.9	71.6	133.5	69	81	168	70	82	93	0.5713	0.5783
7	1.1-3	n„o	1.65	3	39	4250	mascul	69	22338.59	186.25	85.3	156	94	86	149	99	48	45	0.5744	
4	1.1-3	n„o	1.46	1	30	1200	feminino													
5	1.1-3	n„o	1.53	2	42	3980	mascul	99												
1	1 ou -	n„o	1.54	8		3250	feminir	60	32510.46	167.15	74.9	117	71	98	187	66	102	107	0.5828	0.575
3	3.1-6	n„o	1.52	1	42	3470	feminino													
8	1.1-3	n„o	1.56	5	39	2700	feminir	21												
5	1.1-3	1-14 tod	1.66	1		2550	feminino		43707.37	158.25	101.4	140.5	90	95	199	56	131	80		
8	1.1-3	1-14 tod	1.51	3		3620	mascul	14												
7	1 ou -	15 + tod	1.65	3	40	2880	feminir	16	19042.15	168.05	55.6	128.5	87.5	85	208	51	126	149		

pescmae	prenda	pfumomae	paltmae	pgesta	pidgest	ppn	psex	pint	dmgtotdxa	daltura2012	dpeso	dsysmed	ddiamed	dglicose	dcolesterol	dhdl	dldl	dtrig	dECMICE	dECMICD
13	3.1-6	n„o	1.55	2		3650	mascul	16												
9	1.1-3	n„o	1.57	2	36	3460	feminir	32	18336.55	168.45	58.3	112.5	62.5	95	203	79	100	142	0.5662	0.5718
16	10	n„o	1.56	2	36	2650	feminir	42												
5	1.1-3	n„o	1.58	3	40	3800	mascul	16	32416.26	183.3	104.7	137	88	97	176	38	82	431	0.5808	0.5803
5	1.1-3	1-14 tod	1.53	1	39	3100	masculin		13550.11	173	66.8	111	72	81	162	52	86	186		0.5734
5	3.1-6	n„o	1.66	3	39	3750	mascul	81	16576.78	184.85	79.5	121.5	70	58	125	43	66	83		
8	1 ou -	n„o	1.54	2	39	3350	masculin													
4	1.1-3	1-14 tod	1.58	2	41	3080	mascul	50	21096.18	186.8	83.6	126	78.5	94	204	46	136	81		
7	10	n„o	1.64	4	39	3580	mascul	46												
8	1.1-3	n„o	1.6	1	34	1530	masculin		20990.82	174.95	74.8	130.5	79.5	79	250	55	167	117	0.5775	0.5825
4	1.1-3	1-14 par	1.5	7	40	3170	feminir	15	16607.56	160.6	57.3	98.5	69	69	228	55	150	168	0.5706	0.5763
3	1 ou -	1-14 tod	1.53	4	39	2600	mascul	26												
12	1.1-3	n„o	1.54	3	40	3100	feminir	18	28794.49	164.6	65.8	121	90.5	82	183	89	76	60	0.5784	0.5767
0	1.1-3	n„o	1.61	7	41	3630	mascul	24	22465.87	174.9	80.4	121.5	82	95	184	40	116	130	0.5795	0.5809
3	1 ou -	15 + tod	1.62	7	40	4050	feminir	15	6526.803	160.4	51	109.5	65	71	128	51	64	60	0.578	0.5789
9	1.1-3	1-14 tod	1.56	2	37	3330	feminir	40	31194.16	169.55	80.6	113.5	66.5	89	188	62	115	47	0.5915	0.653
6	1.1-3	n„o	1.57	2	40	3260	feminir	46	29557.2	160.65	67.2	121	73.5	89	139	37	82	76		
5	1 ou -	1-14 tod	1.62	4	42	2950	mascul	43												
5	1.1-3	15 + tod	1.51	7	42	3500	mascul	36	25977.28	172.9	88	123.5	74	130	168	41	90	181	0.6109	0.5857
4	1.1-3	n„o	1.57	1	40	3580	masculin		27535.66	171.9	83.1	110.5	73	108	305	56	178	371	0.5797	0.6006
5	1 ou -	n„o	1.57	4		3980	mascul	15	19725.05	173.8	80.9	140	83.5	100	198	44	112	221	0.6129	0.6374
5	1 ou -	n„o	1.51	1	41	3400	feminino		19062.1	155.6	57.8	130	78	74	197	66	102	143	0.5782	0.5807
5	1 ou -	n„o	1.56	3	38	2460	mascul	16	5474.412	172.45	75.4	141.5	58.5	71	243	65	162	66	0.5782	0.632
5	1.1-3	n„o	1.53	1	42	3430	masculin		16362.57	178.65	71.5	120	81.5	87	191	56	124	76	0.5756	
8	1.1-3	n„o	1.56	2	39	3500	feminir	56	30407.81	162.8	73	125.5	79	99	162	45	102	105	0.5947	0.578
0	1.1-3	1-14 par	1.64	2	34	2200	mascul	48												
4	3.1-6	1-14 tod	1.59	4	38	2900	mascul	67	28763.94	166.3	88.5	137	86.5	97	235	54	133	312		
18	10	1-14 par	1.64	3	40	2940	mascul	24												
13	1.1-3	n„o	1.54	1	39	3700	feminino													
6	1.1-3	1-14 tod	1.6	2	36	2420	feminino													
7	1 ou -	n„o	1.47	1	39	3500	masculin		27202.48	170.3	84.8	131	77	68	214	58	139	76	0.5598	
12	6.1-10	n„o	1.65	1	38	3150	masculin		40418.71	183.7	114.6	150.5	83	89	168	43	107	66	0.6271	0.6036
2	1 ou -	n„o	1.43	3	43	2220	feminir	13												
7	1.1-3	n„o	1.57	2		3600	mascul	60	29729.53	175.8	87.8	135.5	88.5	96	175	50	94	177		
15	1.1-3	n„o	1.61	2	41	3500	mascul	15	14641.18	179.3	73.8	120.5	75	85	184	52	114	93		
6	1.1-3	n„o	1.55	2	41	4180	mascul	57												
10	1.1-3	15 + tod	1.65	5	39	3440	mascul	127												
5	3.1-6	n„o	1.53	2	39	3250	mascul	67												
5	10	n„o	1.57	3	36	4020	feminir	151												
0	1.1-3	1-14 tod	1.57	2	42	3600	feminir	37	16322.05	171.65	61	111	71	88	165	76	74	57	0.5617	0.5639
15	10	n„o	1.64	2	39	3970	feminir	53												
5	1.1-3	n„o	1.67	5	40	3200	feminir	17												
5	1 ou -	n„o	1.53	2	36	2600	mascul	18	20013.51	165	72.9	123.5	80	71	175	59	102	81	0.6075	0.5749
4	1.1-3	n„o	1.5	3		4150	masculin		18049.86	183.45	80.6	125	68.5	72	232	62	154	58	0.5799	0.6273
11	1.1-3	n„o	1.53	2	41	3220	mascul	70	15435.67	171.5	81.4	118.5	73.5	80	187	42	126	100		
1	1 ou -	1-14 par	1.53	3	40	3280	feminir	95												
9	3.1-6	1-14 par	1.51	2	34	2200	feminir	120	20105.15	165.4	60.8	123	74	82	166	57	101	50	0.577	0.5843
7	10	n„o	1.54	1	37	3450	feminino		14178.81	160.6	56.9	109	72.5	80	195	77	93	110	0.5783	0.5781

pescmae	prenda	pfumomae	paltmae	pgesta	pidgest	ppn	psex	pint	dmgtotdxa	daltura2012	dpeso	dsysmed	ddiamed	dglicose	dcolesterol	dhdl	dldl	dtrig	dECMICE	dECMICD
3	1.1-3	1-14 tod	1.5	2	40	3250	mascul	31	23181.18	181.3	93.7	115	65	81	175	56	103	88	0.5809	0.6002
5	1.1-3	n„o	1.47	2	39	4040	mascul	65	12509.11	174.3	71.6	124	62	100	189	34	97	288	0.5778	0.5829
5	1 ou -	1-14 tod	1.53	4	40	3450	feminir	10												
12	3.1-6	1-14 par	1.58	2	40	3820	mascul	60	13266.73	171.65	70.7	135	76.5	79	155	70	73	51		
10	6.1-10	1-14 par	1.59	1	39	3450	feminino		16367.36	164.6	54.6	112.5	68.5	73	207	84	116	52	0.5682	0.5783
2	1 ou -	n„o	1.52	1		3140	feminino		22628.74	166.1	69.3	101.5	64	108	207	79	116	49	0.5812	0.5826
5	1.1-3	n„o	1.56	5	39	3500	feminir	59	28552.32	168.2	72.5	104.5	72	78	182	53	107	100	0.583	0.5665
7	1 ou -	n„o	1.53	2		3400	feminir	93	15910.84	158.95	56.5	114.5	74.5	73	200	63	120	61	0.5778	0.585
16	3.1-6	n„o	1.66	2	40	3590	feminino		20757.89	175.15	69.3	97.5	64	107	222	98	107	78	0.5795	0.5747
11	1.1-3	n„o		5	39	4370	mascul	17												
3	1.1-3	1-14 tod	1.57	2	41	3180	mascul	30												
13	10	n„o	1.56	3	39	3100	mascul	46	32978.79	174.2	97.5	137.5	82	86	135	30	86	85	0.5945	0.6111
19	3.1-6	n„o	1.7	2	39	3400	feminir	48												
5	1.1-3	n„o	1.46	1	41	3080	feminino		16756.08	150.1	50.2	105.5	74	80	149	76	66	40	0.5738	0.5779
5	1.1-3	1-14 tod	1.58	4	39	2450	feminir	133	27737.09	152.55	64	97.5	60	74	172	52	107	48		
3	1.1-3	n„o	1.57	1	39	3150	masculin		10736.39	180.05	63.9	116.5	72	94	162	55	87	94	0.5804	0.5784
6	1.1-3	n„o	1.55	1	37	2600	masculin		3765.924	168.25	56.7	124	78	103	170	65	90	66	0.5777	0.5778
8	1.1-3	n„o	1.52	4		3150	feminir	18	11036.84	154.4	50	100.5	64	69	155	64	80	49	0.5787	
5	1.1-3	15 + tod	1.49	2		1900	mascul	79												
2	3.1-6	15 + tod	1.53	4	36	2800	mascul	50												
7	1.1-3	1-14 tod	1.59	1	40	2970	feminino		31790	155.6	71.7	114	71.5	80	228	61	141	140	0.6528	0.5908
17	10	n„o	1.58	3	38	2320	mascul	12												
5	3.1-6	1-14 par	1.58	4	39	4070	mascul	42		181.1	126.4	145.5	92.5	99	315	79	199	200	0.5803	0.5793
18	6.1-10	n„o	1.57	3	41	3000	feminir	16	15894.63	163.6	54.3	112.5	66	78	133	61	62	51	0.547	0.5787
0	1 ou -	1-14 tod	1.58	7	42	3180	feminir	18												
5	1 ou -	1-14 par	1.65	1		3400	masculin		28175.38	180.95	90.3	135.5	70.5	91	206	50	145	62	0.5818	0.5917
7	6.1-10	15 + tod	1.63	2	40	2880	feminir	24												
14	6.1-10	n„o	1.64	1	41	3650	feminino			167.25		110.5	73						0.5768	0.5819
5	1.1-3	n„o	1.57	2	43	3000	mascul	12												
9	3.1-6	n„o	1.58	5	39	3300	feminir	46	25672.91	167.1	64.6	109	59.5	103	178	60	110	64	0.5799	0.5795
3	1.1-3	n„o	1.54	1		2640	feminino		14738.1	155.6	51.2	111	71.5	94	248	74	143	150		
5	1.1-3	n„o	1.51	3	43	3100	feminir	25	51712.59	164.35	99.5	118.5	72	86	248	51	165	133	0.5468	0.5857
5	1 ou -	n„o	1.58	4		1420	feminir	34												
3	1 ou -	1-14 tod	1.57	2	42	2900	mascul	29												
12	3.1-6	15 + tod	1.73	2		3100	mascul	13	18200.4	177.05	71	126	73	98	255	87	152	105	0.581	0.5923
6	1.1-3	n„o	1.63	1		3700	feminino		39366.66	165.5	90.3	133	89	88	216	63	134	124	0.5786	0.5738
6	1.1-3	n„o	1.56	1	41	3350	masculin			189.6	86.4	131.5	78.5	112	200	51	122	163		
7	3.1-6	n„o	1.56	2	39	4000	mascul	49	21622.79	170.9	87.4	133	78.5	102	216	70	123	132	0.573	
6	3.1-6	n„o	1.57	3		3070	mascul	63				134	86							
5	3.1-6	15 + par	1.55	2	39	3650	mascul	48	33135.24	164.25	89.1	139	94	106	302	52	208	228	0.5747	0.5744
4	1 ou -	n„o	1.5	7	40	3170	mascul	19												
0	1 ou -	15 + par	1.56	7	40	3650	mascul	90	6522.904	165.95	55.7	133.5	99.5	80	236	79	137	122	0.59	0.58
8	6.1-10	n„o	1.58	1		4200	masculin		46184.89	170.05	107.9	131	84.5	74	256	56	170	182		
5	1.1-3	n„o	1.5	5	40	3620	mascul	99												
8	1.1-3	n„o	1.52	2		2900	feminir	15	49338.38	153.55	97.7	135.5	90.5	79	192	57	114	152	0.5744	0.5889
12	10	n„o	1.6	2	37	2700	feminir	78	31516.22	160.2	68.6	107	70	89	250	65	152	106	0.5791	0.5786
13	1.1-3	n„o	1.59	3	37	3450	mascul	21	27998.29	173.7	87.4	126.5	75.5	85	145	40	88	80	0.5802	0.5746
0	3.1-6	15 + tod	1.58	8	43	2940	mascul	20												

pescmae	prenda	pfumomae	paltmae	pgesta	pidgest	ppn	psex	pint	dmgtotdxa	daltura2012	dpeso	dsysmed	ddiamed	dglicose	dcolesterol	dhdl	dldl	dtrig	dECMICE	dECMICD
14	10	n„o	1.61	3	38	3980	feminir	20												
16	6.1-10	n„o	1.71	2		3800	mascul	27												
1	1.1-3	n„o	1.48	2	39	3280	mascul	18	28715.42	172.3	92	136.5	92.5	90	242	57	160	126	0.7368	0.59
17	10	n„o	1.67	2	40	4050	mascul	28	16525.99	179.8	76.1	119.5	66	91	158	49	90	130	0.5726	0.5778
5	1.1-3	n„o	1.52	2	40	4000	feminir	16	25206.47	157.4	65.1	102	59.5	68	170	51	98	102	0.5771	0.5783
12	6.1-10	1-14 par	1.63	1	39	3930	masculin													
12	6.1-10	n„o	1.53	2	39	3550	feminir	29	22112.78	155.85	59.1	97.5	60	95	260	56	134	371	0.5717	0.5676
2	3.1-6	n„o	1.59	1	39	4040	masculin													
11	1.1-3	n„o	1.52	1		3260	feminino													
5	1.1-3	n„o	1.5	2		3450	feminir	49												
8	1.1-3	15 + tod	1.49	1		2520	feminino		23130.88	164.2	60.6	106.5	65.5	68	174	44	116	75		
9	1.1-3	n„o	1.6	7	39	5700	masculin		12072.84	191.85	86.1	125	66.5	55	157	54	93	64	0.5507	0.5807
14	3.1-6	1-14 tod	1.48	3	40	2460	feminir	33												
10	3.1-6	1-14 tod	1.57	5		3380	feminir	101	29192.45	163.05	71.2	129	88	71	222	63	128	117	0.5609	0.5725
4	1.1-3	n„o	1.55	6	42	3290	mascul	150	28634.75	179.05	88.3	124.5	73	87	169	55	96	102	0.5891	0.5971
5	1 ou -	n„o	1.52	3	38	3580	feminir	192												
8	3.1-6	n„o	1.48	2	43	3000	feminino													
6	3.1-6	n„o	1.54	7	39	2150	feminir	17	22082.87	155.2	65.4	113.5	76	98	198	46	111	225	0.5789	0.583
5	3.1-6	1-14 par	1.61	1	40	3540	feminino		31621.17	171.9	73.9	105.5	68	80	128	44	75	62	0.5764	0.5817
18	3.1-6	n„o	1.55	1	39	3050	feminino		20389.14	158.2	53.7	118.5	69	97	244	82	143	105		
7	1.1-3	n„o	1.64	3	40	4100	feminir	29	42102.71	170.9	91.2	128	76	136	193	57	122	62	0.5784	0.5816
0	1 ou -	1-14 par	1.5	8	38	2700	feminir	121		161.15	123	133	89.5	88	174	51	100	117		
7	1.1-3	n„o	1.62	4	38	2690	feminir	77	21767.34	159	56.6	110	73	84	220	65	131	124	0.5793	0.5786
13	6.1-10	n„o	1.6	3	39	3240	feminir	57	20734.15	167.1	60.6	105	58.5	73	140	47	75	72	0.5464	0.5738
6	1.1-3	n„o	1.55	1	40	2900	masculin		31146.6	165.25	88.4	159	81.5	81	199	59	111	135	0.6126	0.5723
3	1.1-3	n„o	1.53	2	41	3310	masculin		12787.35	187.3	77.5	124	72.5	67	184	58	107	116	0.581	0.6281
8	1.1-3	n„o	1.55	1	38	3700	feminino		24957.01	159.65	67.9	107.5	70.5	76	170	66	81	107	0.5785	0.6243
6	1.1-3	1-14 par	1.6	1	38	3300	feminino		27749.09	157.8	62.7	121	82.5	91	296	96	178	69		
8	3.1-6	n„o	1.66	1	40	4950	feminino		40860.37	165.4	87.3	110	72.5	77	157	44	93	96	0.5851	
8	1.1-3	1-14 tod	1.57	6	42	3640	feminir	75												
3	1.1-3	n„o	1.5	4	35	3530	feminir	26	24410.87	159.3	62.1	111	75.5	105	199	63	110	102	0.5711	0.5776
11	1.1-3	n„o	1.58	1	41	3430	masculin		38118.04	179.8	102	138.5	90	100	233	52	157	112	0.5759	0.6293
11	3.1-6	n„o	1.68	1	43	3650	masculin		19452.06	187	83.5	125.5	79	79	161	59	92	71	0.5835	0.5903
5	1.1-3	n„o	1.65	4	40	3100	feminir	63	23167.36	162.45	58	105.5	65.5	100	151	70	72	51	0.5743	0.5875
1	1 ou -	n„o	1.6	6	41	3900	feminir	44	23334.25	168.7	65.5	122	69.5	76	186	56	118	50	0.5783	0.5765
5	1 ou -	n„o	1.58	1	40	2550	masculin		13165.11	166.35	65.8	117	74	78	233	81	137	89	0.5774	0.5859
5	1.1-3	1-14 tod		2		3180	mascul	29												
8	1.1-3	1-14 tod	1.52	2		3330	mascul	36												
10	1.1-3	1-14 tod	1.53	2	39	3400	mascul	63	18127.72	172.8	75.5	135.5	86.5	81	201	43	120	148	0.557	0.5721
1	1 ou -	n„o	1.56	2		2400	mascul	20	23454.6	174.6	81.5	132.5	69.5	83	169	39	103	140	0.6935	0.5866
17	10	n„o	1.68	1	43	3100	feminino		13381.15	159.5	50.1	109.5	67.5	74	249	82	138	136		0.5767
16	6.1-10	n„o	1.66	2	40	3180	feminir	16	25475.43	173.2	64.6	127	83	82	197	64	119	88	0.5797	0.5781
6	1.1-3	1-14 tod	1.46	1	40	2970	masculin		17065.7	163.6	67.4	143	92.5	96	187	44	121	81	0.5826	0.5859
12	3.1-6	1-14 tod	1.66	2	37	4430	mascul	69	33280.99	184.4	106.2	148	92.5	97	200	64	127	51	0.5995	0.5872
6	3.1-6	n„o	1.53	1		2980	feminino		21925.83	159.55	59.1	121	69	86	172	50	85	140	0.5715	0.5657
12	10	n„o	1.55	1	39	3000	masculin		24268.05	175.7	84	136.5	79	82	225	75	125	146		
16	3.1-6	n„o	1.55	1	40	3950	masculin		35133.58	171.6	90.4	123	79	93	252	60	175	66	0.5876	0.6036
12	1.1-3	n„o	1.61	3		3600	mascul	33	16417.46	170	79.7	121	71.5	85	189	46	98	224	0.632	0.5859

pescmae	prenda	pfumomae	paltmae	pgesta	pidgest	ppn	psex	pint	dmgtotdxa	daltura2012	dpeso	dsysmed	ddiamed	dglicose	dcolesterol	dhdl	dldl	dtrig	dECMICE	dECMICD
3	1 ou -	n„o	1.54	1	38	2800	masculin													
5	3.1-6	15 + tod	1.55	4	39	2700	feminir	122	16274.2	157.1	55.3	111.5	74.5	89	162	51	85	206	0.5783	0.5786
4	1 ou -	n„o		4	40		mascul	47												
5	3.1-6	n„o	1.6	2	38	3500	mascul	71	36597.6	169.6	107.7	122	74						0.5767	0.5814
5	1.1-3	n„o	1.57	2	39	3900	feminir	40												
5	1 ou -	15 + tod	1.61	3		3000	feminir	77												
9	1.1-3	n„o	1.47	3	39	3100	mascul	102												
2	1.1-3	n„o	1.59	6	39	3400	feminir	92	25431.9	172.55	66.2	119.5	79	90	178	62	106	61	0.5844	0.578
0	1.1-3	n„o	1.54	17	40	2710	feminir	57	11111.48	155.6	41.9	116.5	76	90	168	68	91	41	0.574	0.5799
9	1.1-3	n„o	1.66	1	38	3450	masculin			184.55	125.1	129	88.5	122	175	31	82	321	0.6589	
3	3.1-6	n„o	1.56	3	40	3120	mascul	20	9561.185	179.5	68.8	119.5	76	61	201	53	130	79		
3	6.1-10	1-14 tod	1.55	6	37	2960	feminir	65												
5	1 ou -	n„o	1.61	4		2080	feminir	79												
2	3.1-6	1-14 tod	1.51	2	39	3150	feminir	16												
8	1.1-3	1-14 par	1.54	1	39	2700	feminino		19481.69	158.65	62.6	106	71.5	176	229	75	122	146	0.589	0.5898
4	1.1-3	n„o	1.62	2		4850	mascul	17												
7	1.1-3	15 + par	1.56	4	40	3050	feminir	96	29900.97	163.25	69.1	116	71.5	94	205	62	130	48	0.5789	0.5816
3	3.1-6	n„o	1.52	2	39	1750	mascul	53	9547.524	164.2	58.8	118.5	73	51	154	56	79	137	0.5769	0.5526
12	3.1-6	n„o	1.55	1	43	3600	masculin													
12	6.1-10	n„o	1.67	4	38	4150	mascul	26												
5	3.1-6	15 + tod	1.64	4	37	3400	mascul	92												
4	1 ou -	n„o	1.54	4		2230	feminir	39												
10	3.1-6	n„o	1.65	2	40	3800	mascul	17												
6	1.1-3	n„o	1.63	1	38	3350	feminino		17868.56	159.95	54.1	90	56.5	79	255	99	130	136	0.5527	
6	1.1-3	n„o	1.53	3		3650	mascul	48	26671.17	172.4	81.2	110.5	73	91	241	53	178	69	0.5781	0.5859
2	1 ou -	1-14 tod	1.5	2	40	2940	feminir	19												
5	1.1-3	n„o	1.56	1	41	4510	masculin		15750.8	178.3	75.5	126	66						0.5773	0.6228
4	1.1-3	n„o	1.51	3		2700	feminir	29	27671.91	159.9	67.9	112.5	75.5	83	199	49	138	98	0.5603	
16	6.1-10	n„o	1.71	3	40	3730	feminir	22	30007.36	170.9	71.8	107	64	73	153	58	85	55	0.5772	0.5765
6	1 ou -	n„o	1.63	1	41	3720	masculin													
9	3.1-6	15 + tod	1.56	1	38	3150	masculin													
5	1.1-3	1-14 par	1.54	4	38	2880	mascul	41	28637.33	170.65	99.2	144.5	78	85	206	42	129	181	0.6515	0.6381
0	1.1-3	n„o	1.48	9	40	3950	feminir	152												
2	1.1-3	n„o	1.63	2	42	3500	mascul	12												
16	10	n„o	1.61	2	39	3780	mascul	39												
0	1.1-3	15 + tod	1.53	11		3630	mascul	99						80	159	58	79	128		
4	1.1-3	n„o	1.55	3	38	2420	feminir	100												
3	1 ou -	n„o	1.51	9	39	3070	feminir	18												
2	1 ou -	n„o	1.53	1		2130	feminino													
9	3.1-6	n„o	1.65	1	40	3160	masculin		28061.8	178.65	89.4	132.5	87	89	230	67	143	82	0.5767	0.5781
5	3.1-6	n„o	1.68	3	42	3350	mascul	72	4772.447	170.35	49.9	123.5	77.5	81	152	48	91	76		0.5698
0	1.1-3	n„o	1.59	3		4520	mascul	32	3913.236	177.95	58.1	149.5	97	75	145	55	75	77	0.5788	0.5763
12	10	n„o	1.6	1	39	3300	feminino		19117.53	176.35	58	105.5	69.5	81	197	67	115	50	0.5478	0.5856
13	3.1-6	n„o	1.59	1	41	3550	feminino		36167.75	163	77.3	114	71	87	180	70	94	103	0.5783	0.5781
3	1 ou -	1-14 tod	1.61	4		3850	feminir	22												
4	1.1-3	1-14 tod	1.6	3	39	3350	mascul	16												
9	6.1-10	n„o	1.59	1	42	3250	feminino													
18	1.1-3	n„o	1.58	1	40	3300	masculin		13790.82	175.35	75.8	135.5	77	60	182	63	110	47	0.5782	0.5902

pescmae	prenda	pfumomae	paltmae	pgesta	pidgest	ppn	psex	pint	dmgtotdxa	daltura2012	dpeso	dsysmed	ddiamed	dglicose	dcolesterol	dhdl	dldl	dtrig	dECMICE	dECMICD
5	1.1-3	1-14 tod	1.5	2	39	2640	mascul	59	17807.93	165.25	71.9	112.5	74	94	153	47	84	66	0.5963	0.5604
12	6.1-10	n„o	1.6	4		3520	feminir	11												
13	10	n„o	1.66	2	39	3140	mascul	31												
5	1.1-3	15 + tod	1.49	2	37	3100	feminir	32	34611.83	160.5	74.2	96	76	79	215	64	125	140	0.5768	0.5779
15	10	n„o	1.68	1	39	3900	masculin													
6	1.1-3	n„o	1.46	3	36	2670	mascul	20												
0	1.1-3	1-14 tod	1.48	11	39	4250	mascul	40	4834.622	162.8	54	136.5	86.5	112	155	60	84	57	0.5781	0.5777
5	3.1-6	n„o	1.53	1	43	3900	feminino													
0	1 ou -	n„o	1.52	4	37	2890	mascul	34	13756.51	174.9	73.6	116.5	71.5	93	172	72	81	59	0.579	0.5583
17	6.1-10	n„o	1.65	4	37	2620	feminir	21	50776.31	168	104	141.5	78	89	145	53	83	65	0.5784	
12	1.1-3	15 + tod	1.53	3	32	1500	mascul	27	35898.2	167.6	89.9	152.5	85	93	160	46	92	157	0.5814	
3	1.1-3	1-14 tod	1.54	1	40	3800	masculin		29950.32	184	103.4	135.5	65.5	98	161	45	78	166	0.5779	0.5781
4	1.1-3	n„o	1.67	2		3850	mascul	15												
8	3.1-6	1-14 par	1.53	2	41	1630	masculin													
3	1.1-3	n„o	1.49	3	41	3140	feminir	63	27438	157.6	63.9	114.5	75.5	102	215	53	118	248	0.5774	0.5774
4	1.1-3	1-14 tod	1.61	1	39	2880	feminino		14337.18	168.5	56.4	116.5	69.5	57	163	70	83	76	0.5781	0.5784
7	1.1-3	n„o	1.55	1	36	2750	masculin													
4	1.1-3	1-14 tod	1.49	4	40	2650	mascul	35												
4	1.1-3	15 + tod	1.52	2	41	3750	mascul	156	37860.93	179.9	105.9	125.5	72	101	237	52	129	272	0.6314	0.5792
4	1.1-3	15 + tod	1.55	3	42	3330	feminir	117	42517.46	159.45	93.1	114	68.5	81	232	63	139	184	0.5693	
8	1.1-3	1-14 tod	1.57	2	39	2850	feminir	52												
8	10	n„o	1.55	1	42	3500	masculin		15414.62	165.5	66.1	115	65.5	73	159	41	99	98	0.5853	0.5849
5	1 ou -	1-14 tod	1.57	1	39	3200	masculin													
9	1.1-3	n„o	1.52	2	37	3800	mascul	69	3076.803	171.2	54.2	123	68	93	134	56	65	49		
5	1.1-3	1-14 tod	1.54	1	40	2820	feminino													
8	1.1-3	n„o	1.65	1	41	3070	feminino													
5	3.1-6	n„o	1.52	3	37	3250	feminir	16												
5	1 ou -	n„o	1.36	1	38	3110	masculin		8156.742	170.35	60.5	127.5	79	85	165	79	73	44	0.5777	0.5774
5	1.1-3	n„o	1.63	2	38	4200	feminir	14	39105.91	170.1	95.6	117.5	80	83	199	70	110	89	0.5848	0.5799
8	3.1-6	n„o	1.58	1	38	2780	feminino													
5	3.1-6	n„o	1.53	4	38	3360	mascul	37	31331.05	164.75	96.5	145	84.5	111	245	58	160	152	0.6422	0.5757
7	1.1-3	1-14 tod	1.65	1	42	3130	feminino		39354.94	153.75	86.9	129.5	82.5	87	224	55	155	80	0.595	0.5893
8	1 ou -	1-14 tod	1.5	8		3870	mascul	30												
12	1.1-3	1-14 tod	1.59	3	39	3650	feminir	109												
4	1.1-3	n„o	1.54	2		3500	mascul	62	10946.79	168.7	64	111	72	76	180	64	108	44	0.5734	0.5722
7	3.1-6	n„o	1.52	2		3810	mascul	20	18265.14	171.25	71.6	113	70.5	74	172	60	100	54	0.5801	0.5796
6	1.1-3	1-14 tod	1.49	3	40	3650	mascul	47												
1	1 ou -	n„o	1.5	1	40	3310	masculin													
8	1.1-3	1-14 par	1.5	4	37	3060	mascul	23	23894.21	168.95	79.7	122.5	82.5	87	159	37	107	83	0.5725	0.5556
6	6.1-10	n„o	1.55	2	37	3900	feminir	49	38241.49	166.55	84.7	115.5	74.5	74	227	75	126	157	0.5833	0.5685
7	1.1-3	n„o	1.63	1	40	3700	masculin		21594.56	173.95	73	145	91.5	97	142	44	82	56	0.5785	0.5778
16	10	n„o	1.61	2	39	3850	mascul	52												
4	1.1-3	n„o	1.54	1	40	3100	masculin		8030.063	173	68.2	118	78	38	150	60	76	58	0.5782	0.5758
4	1 ou -	n„o	1.62	1	38	2810	feminino			162.25	50.7	125	79.5	85	136	65	61	45	0.5741	
12	10	15 + tod	1.68	2	38	2940	mascul	17												
5	6.1-10	1-14 tod	1.43	10	38	2420	feminir	48	27055.01	148.45	62.1	116.5	82	75	180	47	118	66	0.5769	0.5693
5	1.1-3	n„o	1.65	2		3300	mascul	123	16764.58	178.2	81.3	119.5	75	96	141	47	77	85	0.5653	0.5759
3	1 ou -	n„o	1.44	1		2750	feminino													

pescmae	prenda	pfumomae	paltmae	pgesta	pidgest	ppn	psex	pint	dmgtotdxa	daltura2012	dpeso	dsysmed	ddiamed	dglicose	dcolesterol	dhdl	dldl	dtrig	dECMICE	dECMICD
1	1.1-3	n„o	1.64	2	37	3310	mascul	53												
12	6.1-10	n„o	1.55	4		2750	mascul	13	9919.273	163.1	65.1	115.5	69	78	204	55	137	70	0.6662	0.579
4	1 ou -	n„o	1.47	5	38	3100	mascul	18	17108.86	166.2	70.3	137.5	81.5	96	231	61	125	269	0.5834	0.5792
19	10	1-14 tod	1.57	2	40	3730	mascul	94												
1	1.1-3	n„o	1.64	5	37	2900	feminir	144												
7	1.1-3	n„o	1.59	1		3600	feminino		22368.91	162.05	64.3	125	81	76	152	54	89	44	0.5782	0.5776
13	6.1-10	n„o	1.56	1	41	3800	masculin													
7	1.1-3	1-14 tod	1.59	1	39	3500	masculin													
5	1.1-3	n„o	1.55	5	39	3400	feminir	80	14293.04	161.75	55.7	115	72	98	165	47	95	140	0.579	0.577
16	10	n„o	1.58	2	40	3350	feminir	28	19280.24	164.75	58.6	107.5	75.5	82	216	89	99	130	0.5794	0.57
5	1.1-3	n„o	1.6	3	36	2500	feminir	27												
6	6.1-10	15 + tod	1.62	3	40	3350	feminir	46												
5	1 ou -	n„o	1.51	1	40	3040	masculin		36249.66	169.5	105.4	142.5	88.5	80	238	52	150	185	0.578	0.583
2	1 ou -	15 + tod	1.67	2		3920	mascul	29	23053.76	181	102.7	139.5	74.5	109	225	56	137	209	0.5734	0.5781
7	3.1-6	n„o	1.57	3		2550	mascul	71				140.5	83.5	97	217	55	116	339		
9	3.1-6	1-14 par	1.63	1	37	2880	masculin													
0	1.1-3	1-14 tod	1.55	12		3300	mascul	12												
6	1.1-3	n„o	1.56	2	36	2900	mascul	24	12125.44	174.1	72.9	151.5	83	71	229	55	158	112	0.6263	0.6374
7	1.1-3	n„o	1.52	2		3900	feminir	97												
4	1.1-3	n„o	1.6	6	38	3500	mascul	27												
4	1.1-3	1-14 tod	1.55	6		3430	mascul	40												
6	1.1-3	1-14 tod	1.58	2	38	2730	mascul	32												
9	1.1-3	n„o	1.63	1	38	3050	masculin		30208.13	175.1	88.2	130	79	83	190	41	124	115	0.5821	0.6214
9	3.1-6	n„o	1.53	2	38	3200	feminir	66	32238.06	158	67.4	131.5	81	85	139	52	71	77		0.5719
16	10	15 + tod	1.56	3	40	3100	mascul	70	20788.21	167.65	74.4	133.5	83	77	184	57	108	106	0.5777	
17	10	n„o	1.6	1	40	3700	masculin		25913.03	182.95	87.9	126.5	75	95	249	45	164	212	0.6703	0.5795
16	10	n„o	1.66	1	41	3710	masculin													
9	1.1-3	n„o	1.57	1	40	3300	feminino													
12	3.1-6	n„o	1.54	1	35	2130	masculin		16514.37	169.95	71	146.5	95.5	102	222	41	130	246	0.5958	
8	6.1-10	n„o	1.56	2	41	3870	feminir	57	44051.66	168.85	86.3	120	72.5	74	173	49	109	91	0.5794	0.5913
0	1.1-3	n„o	1.5	1		2700	masculin													
6	3.1-6	1-14 tod	1.59	2	40	3000	feminir	87												
4	1.1-3	n„o	1.6	6		4380	mascul	22	21186.13	176.05	82	113.5	71	86	218	84	111	97	0.5793	0.5792
5	6.1-10	1-14 par	1.58	1	41	3300	masculin													
0	1 ou -	n„o	1.51	9		2660	mascul	17												
7	1.1-3	1-14 tod	1.51	2	39	3500	mascul	78												
12	3.1-6	n„o	1.6	3	39	3380	feminir	21	55804.66	166.05	104.8	133	86	56	228	71	135	176	0.5896	
7	1.1-3	1-14 par	1.45	1	41	3290	masculin													
0	1.1-3	n„o	1.54	4		3720	mascul	12	11693.27	167.5	75.1	131	76.5	82	160	51	92	81	0.5791	0.5775
12	1 ou -	n„o	1.59	1	38	3570	masculin		40458.74	173.65	103.9	136	89.5	129	173	38	93	276	0.6318	0.5828
3	1.1-3	n„o	1.45	3	41	2930	feminir	63	12606.01	147.5	43.3	97	60	85	167	58	98	70	0.5674	
14	3.1-6	15 + tod	1.61	1	40	3250	masculin													
3	1.1-3	n„o	1.6	9		1360	feminir	16	36935.07	163.3	78.3	112.5	78.5	92	152	54	88	47	0.5792	0.5759
10	3.1-6	n„o	1.63	2	39	3650	feminir	78	25308.42	169.45	66.6	112.5	76	79	200	63	117	125	0.5809	0.5804
5	1.1-3	1-14 tod	1.41	2	40	2890	feminir	50	35863.64	154.15	75.1	122.5	77.5	70	185	71	101	50		0.5784
16	6.1-10	n„o	1.57	1	39	3840	masculin													
7	3.1-6	n„o	1.68	1	39	3050	masculin													
6	1 ou -	n„o	1.65	1	42	3900	masculin													

pescmae	prenda	pfumomae	paltmae	pgesta	pidgest	ppn	psex	pint	dmgtotdxa	daltura2012	dpeso	dsysmed	ddiamed	dglicose	dcolesterol	dhdl	dldl	dtrig	dECMICE	dECMICD
10	3.1-6	n„o	1.53	2		3790	mascul	31	29811.64	180.8	95.1	127	85	85	203	55	128	84	0.6188	0.5835
18	10	n„o	1.66	3	39	3600	feminir	45												
1	3.1-6	n„o	1.58	3	41	3400	mascul	135	44208.07	175.85	112.4	144.5	93	63	119	33	73	59	0.7006	0.586
4	3.1-6	n„o	1.59	4	37	2970	mascul	30	25397.28	178.25	87.6	137	82	118	189	70	91	170	0.576	0.5806
4	1.1-3	15 + tod	1.5	1		2700	feminino		29863.24	159.1	71.9	106	67	78	220	57	123	295	0.6612	0.5827
9	1.1-3	1-14 par	1.7	2	42	3750	mascul	45	33005.82	178	100	121.5	73	92	226	62	113	282	0.6813	0.6677
6	3.1-6	1-14 par	1.58	3	41	3200	feminir	47												
6	1.1-3	n„o	1.54	2		2610	feminir	17	58071.68	157.7	110.2	98.5	64	96	185	59	107	58	0.5878	
6	1 ou -	1-14 tod	1.53	2		3250	feminino		30444.61	161.75	72.2	105	67.5	88	150	46	86	124	0.5773	0.5782
1	1 ou -	1-14 par	1.47	3	40	3010	mascul	23												
7	1.1-3	n„o	1.58	1	40	2850	masculin													
3	1.1-3	15 + par	1.47	2		3400	mascul	15												
9	1.1-3	1-14 tod	1.62	2	43	2800	feminir	37												
1	1.1-3	1-14 tod	1.58	5		3000	mascul	29	8871.069	177	67.9	149	82.5	96	164	57	93	55	0.5853	0.5781
7	3.1-6	1-14 par	1.65	2		3150	mascul	12												
0	1.1-3	n„o	1.42	2		2860	mascul	13	14497.75	162.7	64.2	120.5	74.5	83	202	52	137	88	0.5889	0.6333
17	10	1-14 tod	1.58	5	40	2970	feminir	73		165	47.1	94	66	90	185	78	88	98		
7	1.1-3	n„o	1.51	1	38	3060	masculin													
3	3.1-6	n„o	1.53	3	40	3550	feminir	150	24189.27	161.2	64.4	108	71	86	169	64	83	125	0.5761	0.5783
1	1.1-3	1-14 tod	1.51	4		1900	feminir	37												
12	10	n„o	1.59	1	41	3690	masculin													
5	1.1-3	n„o	1.55	1	42	3350	feminino		25982.4	164	67.2	107	72	92	210	89	100	140		
4	1 ou -	15 + tod	1.6	1		3220	masculin													
4	1 ou -	1-14 tod	1.6	1	42	2950	masculin		3315.503	162.8	53.2	105	71.5	91	190	63	103	117	0.5674	0.5771
6	3.1-6	n„o	1.56	5	42	3250	feminir	29	44268.03	160.15	83.7	114.5	73.5	84	269	73	178	105	0.5869	
5	1.1-3	n„o	1.63	2	42	3300	mascul	12												
7	3.1-6	n„o	1.52	2		3050	mascul	27	32237.23	176.2	91.4	130	81.5	110	184	44	104	197	0.5835	0.5785
10	1.1-3	n„o	1.49	1	37	2620	feminino			156.7		137	90						0.5866	0.583
4	1 ou -	n„o	1.54	1		3200	feminino		30321.74	165.8	77.6	105.5	68	73	223	65	141	82		0.5645
5	3.1-6	n„o	1.56	2	37	3310	feminir	15												
5	1.1-3	n„o	1.61	2	40	3850	mascul	71	28181.29	176.65	87.5	135.5	84.5	91	235	78	136	89	0.5736	0.5864
5	1.1-3	n„o	1.58	1	40	4100	feminino													
15	10	n„o	1.58	3	40	3350	feminir	27	37999.5	162.25	74	113	86.5	95	193	68	101	132		0.5633
5	1.1-3	n„o	1.67	4		3400	mascul	67		191.65	123.1	132.5	85	124	263	47	110	539	0.5886	0.5758
5	3.1-6	15 + tod	1.61	7	41	2480	mascul	26												
0	1.1-3	n„o	1.58	12	39	3500	mascul	94	4361	178.65	55.2	130	78	98	173	68	96	43	0.5914	0.5873
0	1 ou -	1-14 tod	1.53	5		2760	feminir	21												
5	1 ou -	n„o	1.6	5		3360	feminir	21		164.8	62.5	101.5	60	51	151	50	91	48	0.5781	0.5749
7	1.1-3	n„o	1.52	1	37	3000	feminino		12499.5	165.3	47.6	119	73	74	145	62	68	59	0.5786	0.5783
6	1.1-3	1-14 tod	1.56	2	40	3700	mascul	54												
8	1.1-3	1-14 tod	1.59	2		3360	mascul	90	29153.32	173.7	83.8	142.5	88	75	163	53	101	49	0.5745	0.5784
3	1.1-3	1-14 par	1.61	1	39	3160	feminino		50058.8	163.75	93.9	103.5	73	85	177	64	100	73	0.5691	0.5912
5	1.1-3	n„o	1.47	2	41	3710	mascul	13	27277.44	173.1	95.9	132.5	79	95	218	71	133	92		0.5808
2	1.1-3	1-14 tod	1.49	4		3200	mascul	42												
4	3.1-6	n„o	1.56	1		2900	masculin		14567.93	173.4	68.3	136	83	94	189	51	123	85	0.5785	0.5774
15	3.1-6	1-14 par	1.58	1	36	2780	feminino													
3	1 ou -	n„o	1.85	1	40	3230	masculin		15055.86	182.4	78.3	120.5	78.5	84	120	44	64	55	0.5811	0.5783
5	1.1-3	n„o	1.68	2	41	3720	mascul	61	18801.46	168.4	80.9	135.5	79.5	92	189	64	113	62	0.5779	0.5365

pescmae	prenda	pfumomae	paltmae	pgesta	pidgest	ppn	psex	pint	dmgtotdxa	daltura2012	dpeso	dsysmed	ddiamed	dglicose	dcolesterol	dhdl	dldl	dtrig	dECMICE	dECMICD
6	1 ou -	1-14 tod	1.57	3	37	2700	mascul	30												
3	1 ou -	1-14 par	1.53	4		2930	feminir	15	10793.6	150.8	43.7	98.5	65	126	178	67	94	54	0.5772	0.573
3	1 ou -	1-14 par	1.61	1		2790	feminino													
4	1.1-3	15 + par	1.61	2	43	3100	feminir	35												
5	1.1-3	n„o	1.6	1	40	3620	feminino		24001.8	164.85	66.8	104.5	62.5	63	188	65	105	72		
12	1.1-3	15 + tod	1.62	2	40	3600	feminir	54	23129.34	176.8	65.4	99	65	125	222	83	123	65	0.5715	
0	1.1-3	1-14 par	1.64	1	40	3000	feminino		20177.51	166.15	63.6	115.5	75	88	216	67	127	107		
5	1 ou -	n„o	1.53	1	41	3400	masculin		22756.08	172.15	85.1	129	76	108	211	54	121	161	0.5795	0.5941
6	1.1-3	n„o	1.7	1	40	2500	masculin													
7	1.1-3	1-14 tod	1.62	2	41	3260	feminir	14	30002.42	166.25	72.3	103	63.5	82	206	64	134	51	0.5857	0.5842
8	3.1-6	n„o	1.59	3	35	2800	mascul	70	18867.4	183.75	88.4	112.5	61.5	79	159	51	92	81	0.5725	0.5707
15	3.1-6	n„o	1.67	1		3650	feminino		17409.58	167.9	58.9	122.5	75	81	175	64	95	56	0.5636	0.5557
9	1.1-3	n„o	1.54	3	39	3570	mascul	21	13260.28	186.45	67.5	107	68.5	71	190	59	117	63	0.5655	
12	1.1-3	15 + par	1.64	1	39	3790	masculin		37615.81	187.35	99.9	106	68	74	235	45	141	206	0.5804	0.5807
4	1 ou -	n„o	1.6	4	42	4160	feminir	59	56048.75	169.6	112.9	115	77	94	243	61	150	175		
7	1.1-3	1-14 tod	1.52	1	40	3200	masculin		17712.33	178	80.7	127	82	81	215	58	139	94	0.5877	0.5794
4	1 ou -	15 + par	1.52	4	41	3800	mascul	28	3739.715	175.5	62.1	116.5	69	115	170	65	80	132	0.5787	0.5764
9	3.1-6	n„o	1.58	1	40	3570	masculin													
4	1.1-3	15 + tod	1.58	1	39	3400	masculin		14984.96	181.6	76.5	140	85.5	120	224	55	147	128		0.5874
16	10	1-14 par	1.6	4	39	3350	mascul	98	29797.73	179.15	91.9	133.5	91.5	81	250	60	174	96	0.5785	0.5855
5	1.1-3	n„o	1.64	2	39	4390	mascul	35	25124.58	182.4	80.9	128.5	80	73	167	50	97	93		
1	1.1-3	n„o	1.55	9		4000	feminir	80	26154.45	161.3	70.8	99	74.5	70	219	54	151	87	0.6361	0.6306
12	3.1-6	n„o	1.63	3	38	3450	mascul	95												
1	1 ou -	1-14 tod	1.59	1	40	1880	feminino													
1	1.1-3	n„o	1.56	2	39	2560	feminir	41	20886.41	158.3	57.9	109	67	71	178	47	115	74		
0	1 ou -	n„o	1.48	6		3500	mascul	64												
16	10	n„o	1.62	3	39	4700	mascul	21	17527.01	175.9	83.7	111.5	70	82	166	61	98	40	0.578	0.5753
11	3.1-6	1-14 tod	1.59	1	40	3770	masculin			185.05	128.2	142	96	99	229	42	118	356		
12	6.1-10	1-14 tod	1.61	3	38	2900	mascul	43	19189.23	175.15	67.9	129.5	84.5	93	154	49	78	143	0.5786	0.5716
5	3.1-6	n„o	1.55	2	39	2810	feminir	85	43898.41	156.1	85.4	126.5	76	87	260	59	151	291	0.5672	0.5814
2	1 ou -	n„o	1.6	5	41	4100	feminir	18	49227.52	159.9	102.6	111	80	81	152	64	80	36	0.6257	0.5937
12	3.1-6	n„o	1.54	3	41	3430	feminir	84		162.85		120.5	83							0.5697
3	1.1-3	1-14 tod	1.6	3	37	3000	mascul	57												
6	1 ou -	n„o	1.6	4	37	3050	mascul	145	26011.78	177.2	88.1	130.5	71.5	96	160	42	109	70	0.6541	0.5907
2	1.1-3	n„o	1.5	1		3200	feminino		26006.25	155.15	63.5	110.5	68.5	86	207	75	120	64	0.5776	0.5781
5	1.1-3	n„o	1.55	1	40	3260	feminino													
4	1.1-3	n„o	1.55	1	41	4250	masculin		23483.94	188.55	93	132	78.5	107	192	50	108	164		0.5985
14	6.1-10	1-14 tod		2		3710	feminir	36												
0	1 ou -	n„o	1.58	1	37	2600	masculin		32856.64	171.35	92.9	131.5	74						0.6066	0.5833
5	1 ou -	1-14 tod	1.57	1	40	3130	masculin		6234.617	170.2	60.9	109.5	71	129	250	82	143	124	0.5755	0.5385
7	3.1-6	n„o	1.6	2	41	3600	mascul	84												
5	1.1-3	1-14 par	1.61	1	35	2100	feminino		10497.72	166.9	50	117.5	88.5	100	194	75	102	55	0.5788	0.5781
7	1.1-3	n„o	1.58	2	42	3600	mascul	48	39043.75	174.1	101.1	136	84	82	223	52	133	211		
7	1 ou -	n„o	1.44	4	40	3150	feminir	13	28583.07	162.05	68.4	107	72.5	105	178	43	115	94	0.5984	0.6011
4	1.1-3	1-14 tod	1.58	4	29	950	feminir	45												
1	1.1-3	n„o	1.58	3	41	3400	feminir	21	24933.77	169.7	73	116	71.5	87	225	88	113	133	0.5798	0.5748
2	1.1-3	n„o	1.51	5	41	4450	mascul	128	17531.48	177.3	78.2	120	71	71	212	64	128	115		
9	3.1-6	n„o	1.58	2	40	3200	mascul	46												

pescmae	prenda	pfumomae	paltmae	pgesta	pidgest	ppn	psex	pint	dmgtotdxa	daltura2012	dpeso	dsysmed	ddiamed	dglicose	dcolesterol	dhdl	dldl	dtrig	dECMICE	dECMICD
2	1 ou -	n„o	1.59	2	39	3100	feminir	14												
8	1.1-3	n„o	1.5	2	41	3250	feminir	90												
1	1 ou -	n„o	1.5	9		3700	masculin			192.05	103.1	151	79	106	157	38	79	197	0.5762	0.5788
7	1.1-3	15 + tod	1.53	3	39	3350	mascul	66	16594.66	175.05	85.1	128	70	91	183	59	111	77	0.5787	0.5809
7	3.1-6	n„o	1.64	1	41	3050	masculin		36532.61	183.35	117.4	152.5	92.5	113	193	37	96	345	0.628	0.5976
18	6.1-10	n„o	1.52	2	39	3980	mascul	34	36644.66	181	110.4	125	77	84	214	47	129	160	0.5992	0.5811
10	6.1-10	n„o	1.62	2	41	4000	feminir	36	25778.41	173.9	69.5	111	68	70	220	85	125	62	0.5744	
8	1 ou -	1-14 tod	1.56	1	42	2550	feminino		44277.31	158.6	87.9	126.5	90	84	254	61	167	136		
5	3.1-6	n„o	1.6	2	38	2820	masculin			174.8	145.8	151	84.5	109	191	47	105	194	0.5788	
5	3.1-6	n„o	1.62	3	41	3900	mascul	29	12158.2	181.55	67.4	114.5	71.5	82	149	60	81	53	0.578	0.5797
5	1.1-3	n„o	1.55	1	40	2800	feminino													
12	3.1-6	n„o	1.57	2		3150	masculin		11598.54	173.55	66.3	127	74.5	79	173	61	93	103	0.5775	0.5861
18	6.1-10	n„o	1.58	1	38	2950	masculin		24937.02	174.7	87.8	127.5	84.5	98	232	44	140	240	0.5824	0.5939
6	6.1-10	n„o	1.53	4	41	3200	mascul	46												
11	3.1-6	1-14 tod	1.63	1	41	3650	feminino		24600.71	163.65	66.6	104	59.5						0.5575	0.5778
7	3.1-6	1-14 par	1.45	3	40	3300	mascul	53	26215.86	172.2	98	134.5	76	83	197	39	118	216	0.5994	0.6483
5	1.1-3	n„o		2		3350	mascul	13	24776.06	181	88.9	157	75.5	103	203	64	130	54	0.5687	0.5546
4	1 ou -	n„o	1.52	1		2980	feminino		21833.43	158.45	57	115.5	76.5	55	158	54	96	43	0.5759	0.578
3	1 ou -	1-14 tod	1.58	3	36	2200	feminir	23	36612.99	167.4	87	122	79.5	99	198	59	120	121	0.5807	0.5805
4	1.1-3	n„o	1.52	1	42	3580	feminino		34195.83	153.8	74.2	110.5	71	77	206	69	115	117	0.7308	0.5581
7	1.1-3	n„o	1.62	1	37	3000	masculin													
9	3.1-6	1-14 tod	1.49	3	39	3270	feminir	42	27386.07	158.4	67.7	110	70	94	175	60	96	164	0.5792	0.5909
2	1 ou -	1-14 par	1.55	1		2460	feminino		33322.12	163.6	78.9	112.5	73.5	88	250	52	178	121	0.5873	0.578
0	1 ou -	n„o	1.52	4	40	3770	mascul	40	3971.679	177.35	61.5	117	60.5	89	168	51	101	75	0.5781	0.5774
4	1 ou -	n„o	1.52	2	37	2900	feminir	15	14723.38	166.05	59.2	119.5	69	72	217	60	141	121	0.5781	0.5671
1	1 ou -	1-14 par	1.49	2		2900	mascul	15												
5	1 ou -	n„o	1.68	1	41	4000	masculin		26567.03	186.1	89.3	144.5	85.5	94	188	56	122	52	0.5464	0.5796
7	1.1-3	1-14 tod	1.55	1	38	3100	feminino		15513.22	167.8	56.1	104	69.5	87	219	68	139	60	0.5783	0.578
0	1.1-3	1-14 tod	1.55	4		2100	feminir	52												
7	1.1-3	1-14 tod	1.58	2	37	2880	mascul	25												
5	1.1-3	n„o	1.56	1	41	3610	feminino		20240.8	167.1	58.2	102.5	62.5	81	225	77	115	174		0.5737
8	1.1-3	n„o	1.52	1	39	2250	feminino		21585.01	155.5	54.7	116	75	84	215	54	135	125	0.5788	0.593
2	1 ou -	1-14 tod	1.56	7	39	4000	mascul	39	12609.79	163.7	75.4	121	63.5	65	236	64	145	139	0.6171	0.5781
3	1 ou -	n„o	1.44	8	39	2770	feminir	11	10209.17	156.4	46.1	107.5	72	80	165	74	81	39	0.5766	0.5416
7	3.1-6	1-14 tod	1.57	3	39	2750	feminir	31	22571.59	168.25	68.1	97.5	62.5	79	200	61	126	42	0.5776	0.5674
4	3.1-6	n„o	1.52	3	41	3850	mascul	38	11562.34	171.15	68.8	125.5	75	110	208	46	104	300	0.612	0.6329
15	6.1-10	n„o	1.5	2	38	3350	feminir	46												
7	1.1-3	n„o	1.69	1		3500	masculin													
7	1.1-3	1-14 tod	1.62	2	41	3230	feminir	48												
12	3.1-6	n„o	1.53	2	39	4030	mascul	72												
5	1.1-3	n„o	1.52	1	41	2600	masculin		21117.46	172.6	75.5	116	72	85	225	48	162	131	0.5793	0.5827
6	1.1-3	n„o	1.64	1	40	4200	masculin													
3	1.1-3	n„o	1.51	2	40	3550	mascul	14	15527.85	176	69.8	110	66	84	120	41	62	73	0.5752	0.5749
5	1 ou -	n„o	1.5	1		3200	masculin													
14	3.1-6	1-14 tod	1.65	1	40	2790	feminino													
9	3.1-6	1-14 tod	1.57	2	40	3770	feminir	57	20831.26	164.25	55	130.5	78	81	201	87	91	136	0.5812	0.6196
0	1.1-3	n„o	1.56	8	42	3500	feminir	24	25921.55	159.9	62.6	106	74.5	83	166	53	94	90		
1	1 ou -	n„o	1.6	7	40	3900	feminir	24	33907.64	167.85	83.3	115	63.5	80	229	62	157	76	0.5814	0.6217

pescmae	prenda	pfumomae	paltmae	pgesta	pidgest	ppn	psex	pint	dmgtotdxa	daltura2012	dpeso	dsysmed	ddiamed	dglicose	dcolesterol	dhdl	dldl	dtrig	dECMICE	dECMICD
0	1 ou -	n,,o	1.5	2		2800	feminir	59	18613.37	157.5	53.2	115.5	77.5	82	190	78	100	69	0.5784	0.5664
14	10	1-14 par	1.61	3	38	3420	feminir	40												
1	1 ou -	1-14 tod	1.56	5	37	2350	feminir	20	15166.87	156.8	51.9	121.5	79	97	270	68	178	164	0.5781	0.5772
7	1.1-3	n,,o	1.53	6	43	2260	feminir	17		164.5		94	70.5						0.577	0.5615
4	1 ou -	n,,o	1.52	2	40	3130	feminir	116												
4	1.1-3	n,,o	1.5	1		3300	feminino													
5	1.1-3	1-14 tod	1.69	3	37	2920	mascul	39												
4	1 ou -	1-14 tod	1.59	2		2730	feminir	21	17551.08	153.9	56.6	102	58.5	80	175	42	112	108	0.5795	0.5744
9	1.1-3	n,,o	1.61	2	40	3330	feminino													
2	1.1-3	n,,o	1.47	6		1980	feminir	64												
7	1.1-3	1-14 tod	1.55	5	41	3470	feminir	21	22870.88	150.7	59	102	62	243	230	118	82	117	0.5686	0.5807
6	1.1-3	1-14 par	1.53	2	40	3000	feminir	41												
4	1.1-3	n,,o	1.59	2		2900	feminir	57	15045.74	163	50.6	114	76.5	76	190	83	93	54	0.5773	0.6123
16	10	15 + tod	1.71	2	37	2900	mascul	24												
12	10	n,,o	1.58	3	40	3920	feminir	45												
5	1.1-3	n,,o	1.57	1	41	3250	masculin													
5	1.1-3	n,,o	1.54	5	37	2920	feminir	34	18111.04	158.4	56.2	108.5	60.5	98	169	45	100	148	0.5623	0.5714
5	1.1-3	n,,o	1.58	1	42	3400	feminino		16629.46	154.55	54.8	109.5	77	93	215	82	115	99	0.5779	0.5782
11	3.1-6	n,,o	1.57	2	39	3980	mascul	18												
4	3.1-6	15 + tod	1.6	9	40	3530	mascul	14	18221.68	174.85	80.1	138	77	63	190	48	99	210	0.5781	0.7007
4	1 ou -	1-14 tod	1.59	1	42	2900	feminino		3623.319	156.45	33.7	130.5	98	83	184	74	95	67	0.5568	0.5735
6	3.1-6	1-14 tod	1.49	3	38	3250	mascul	46	25075.7	177.35	79.7	110.5	66.5	84	214	63	137	54	0.5864	0.5786
17	10	1-14 tod	1.56	1	36	1950	masculin		14760.28	170.95	78.2	123.5	72	92	177	69	92	75	0.5586	0.5785
18	3.1-6	1-14 tod		1	39	2870	feminino		48212.61	156	96.6	122	78	85	205	66	117	102	0.5574	
3	1.1-3	1-14 tod	1.54	2	37	3250	feminir	19	30484.51	159.5	67.4	132	93.5	80	150	53	88	66	0.5793	0.5792
4	1.1-3	1-14 par	1.61	3	39	3550	feminir	24												
	1 ou -	n,,o	1.5	1	41	3400	masculin		24906.43	180.05	86.6	131.5	77.5	103	191	49	116	111	0.5789	0.5786
2	1.1-3	n,,o	1.52	1	40	3700	masculin		9312.331	178.75	81.4	142.5	77	63	133	31	82	98	0.5788	0.5751
8	1.1-3	n,,o	1.55	1	41	3410	feminino		45305.07	161.3	95	127.5	88	78	185	53	111	103		0.5737
12	10	n,,o	1.66	3	38	3000	feminir	18	19904.71	169.7	64.9	110	64	84	215	87	103	118	0.583	0.5785
7	1.1-3	15 + tod	1.55	3	38	3020	feminir	76												
7	1.1-3	n,,o	1.45	1	40	3320	masculin		25017.46	166.3	88.9	139	91.5	111	234	51	149	176	0.6283	0.5721
13	10	n,,o	1.67	4		3750	feminir	48	28459.32	178.4	74.2	113	73	73	237	77	143	80	0.5783	0.6008
5	6.1-10	n,,o	1.63	3	35	2950	mascul	145	31020.65	185.2	96	149.5	89	96	199	50	116	180	0.5795	0.5816
3	1 ou -	15 + par	1.58	2	37	3200	feminino													
4	1.1-3	n,,o	1.54	3	42	3700	feminir	19	41891.08	161.3	82.2	119.5	71.5	92	150	50	82	105	0.5755	
0	1 ou -	n,,o	1.53	9		3900	mascul	29												
12	3.1-6	1-14 par	1.53	4		3170	mascul	17												
10	1.1-3	n,,o	1.54	1		3350	masculin		18167.18	178.5	77.1	122.5	74	86	239	87	131	130	0.5828	0.5789
5	3.1-6	15 + tod	1.59	7		2400	feminir	120												
7	3.1-6	1-14 par	1.64	4	40	3920	mascul	68												
5	3.1-6	1-14 par	1.64	2	41	3800	mascul	58	15788.03	182.5	77.4	117	76.5	85	187	50	129	40	0.5778	0.578
9	1.1-3	1-14 par	1.64	1	40	2940	masculin													
6	1.1-3	n,,o		1		3250	masculin													
0	1 ou -	n,,o	1.61	7		3050	mascul	12												
10	1.1-3	1-14 tod	1.58	3	40	3400	mascul	32	24559.86	174.25	93.3	123.5	73	90	197	73	107	88	0.5783	0.5782
7	1.1-3	n,,o	1.65	1	40	3100	feminino		38252.09	168.7	81.1	107.5	71.5	74	149	49	71	163	0.5773	0.5782
11	1.1-3	1-14 tod	1.68	2	41	3920	mascul	36	39144.91	180.7	108.8	120	77	91	208	46	132	109	0.5783	0.5793

pescmae	prenda	pfumomae	paltmae	pgesta	pidgest	ppn	psex	pint	dmgtotdxa	daltura2012	dpeso	dsysmed	ddiamed	dglicose	dcolesterol	dhdl	dldl	dtrig	dECMICE	dECMICD
5	1.1-3	n„o	1.48	4	38	3000	mascul	59	15590.06	164.4	73.4	122	76	92	208	53	109	262	0.5797	0.5909
6	1.1-3	1-14 par	1.53	2	37	3250	feminir	12	19740.1	166.2	61.8	106	70.5	60	120	32	75	39	0.5777	0.5438
9	1.1-3	1-14 tod	1.51	2	41	3900	feminir	27												
9	1.1-3	1-14 tod	1.54	3	38	3050	feminir	12												
6	1 ou -	n„o	1.61	2		2930	feminir	25				104.5	74							
7	3.1-6	1-14 tod	1.49	3	36	2300	feminir	32	20836.13	152.05	59	102.5	70.5	116	226	56	140	131		
16	3.1-6	n„o		1	37	3270	masculin		17365.67	185.2	86.9	129.5	73.5	88	260	57	176	140	0.5793	
5	1.1-3	1-14 par	1.61	1		2850	masculin		19313.14	167.6	76.4	137.5	91	145	185	31	89	471		0.5941
5	3.1-6	15 + tod	1.5	1		2960	feminino													
5	6.1-10	n„o	1.43	3	39	2800	feminir	73												
8	1.1-3	n„o	1.59	1	39	2800	masculin		21991.47	180	82.3	135	90	78	220	55	143	99	0.7013	0.614
5	1.1-3	n„o	1.65	4	39	3780	feminir	171	53354.3	167.65	113	123.5	85	93	169	51	102	101	0.5796	0.5779
5	1 ou -	n„o	1.55	3	39	3320	feminir	101												
9	3.1-6	1-14 par	1.61	1	43	3150	feminino		32402.62	171.8	70	115.5	79.5	95	230	95	97	128	0.577	0.5755
9	3.1-6	1-14 tod	1.61	2	37	3780	feminir	25												
0	1 ou -	n„o	1.58	5	42	2690	mascul	22												
5	1 ou -	n„o	1.59	1		2180	feminino		36853.21	154.4	75.7	112	74	122	179	62	98	156	0.5691	0.5843
8	1 ou -	n„o	1.55	2		2650	feminir	24												
8	1.1-3	n„o	1.6	1	41	3500	feminino													
13	3.1-6	1-14 tod	1.5	2	41	3020	mascul	46	18656.68	171.25	79.4	115	69.5	89	237	61	151	100	0.5868	0.576
7	1.1-3	1-14 par	1.52	1		3080	masculin		10027.48	169.2	60.5	129.5	86.5	90	175	48	93	172	0.5758	0.578
5	1.1-3	1-14 tod	1.51	1	39	2950	feminino		53305.55	146.7	116.6	146.5	94.5	86	226	55	121	303		
8	1.1-3	n„o	1.5	2	39	3290	feminir	31	36502.05	165.95	77.1	130	82	91	190	73	96	110	0.5516	
6	1.1-3	1-14 tod	1.53	1		2760	masculin		39609.38	174.15	100.7	128	79.5	86	290	63	175	276		
8	1.1-3	n„o	1.53	1	37	2550	feminino		15142.31	161.8	56	105.5	60.5	78	168	65	84	72		
2	1.1-3	n„o	1.59	4	39	2470	feminir	36	51502.39	165.3	104.7	127	87	84	157	48	92	104	0.5773	0.5783
3	6.1-10	n„o	1.61	2	40	3100	mascul	17												
1	1.1-3	n„o	1.63	6	39	3200	feminir	60	24724.97	171.25	71.4	101.5	58.5	82	120	44	65	45	0.576	0.5646
11	1.1-3	n„o	1.63	1	37	2640	feminino		42859.23	157.8	82.3	136	88	81	165	61	91	79	0.5911	0.6082
8	3.1-6	n„o	1.5	1	39	4000	masculin													
4	1.1-3	1-14 tod	1.66	1		3700	masculin		30390.6	182.25	93.6	122.5	77	96	275	51	144	373	0.6225	0.5915
8	1.1-3	n„o	1.58	1	40	3650	feminino		49240.75	165.6	97.5	114	80	91	260	67	170	118	0.6044	
15	6.1-10	n„o	1.53	1	39	3880	masculin		30918.54	186.5	94.8	164.5	107	114	222	50	150	96		
9	3.1-6	n„o	1.6	1	41	3720	masculin													
4	1 ou -	n„o		1		2660	feminino													
19	10	n„o	1.59	2	41	3150	feminino													
5	1.1-3	n„o	1.53	4	39	3900	mascul	34												
7	3.1-6	1-14 tod	1.63	1	40	3420	feminino		34813.5	157.1	73.7	100	63.5	85	142	46	85	48	0.5618	0.567
4		15 + tod	1.5	1	26	750	masculin													
6	1.1-3	n„o	1.59	1		3200	masculin		27957.08	178.2	86.2	133.5	76	124	195	57	123	112	0.5803	0.5807
7	1.1-3	n„o	1.56	1	38	3060	masculin		43215.29	174.1	117	150	97.5	92	258	80	158	124	0.5791	0.5974
1	1.1-3	n„o		5	41	3600	feminir	86	41605.34	154.25	88.9	117	76.5	84	168	55	101	102		
13	3.1-6	n„o	1.62	2	39	3200	feminir	41	38899.87	169.05	80.4	113	69	80	229	67	141	149		0.6065
16	10	1-14 par	1.57	3	38	3550	feminir	24	14100	156.5	48.2	131	92	82	309	97	188	136	0.5664	0.5769
6	1.1-3	1-14 par	1.49	1	40	3450	feminino		17803.81	152.1	53.4	98	62.5	84	153	53	80	89	0.571	0.5814
5	1.1-3	1-14 tod	1.5	2		2850	mascul	14	25589.73	162.55	69.2	134	84	75	170	36	119	72		
5	1.1-3	15 + tod	1.55	6	37	3300	mascul	54	12079.34	163.3	64.6	111.5	63	87	145	55	65	135	0.5795	0.5816
5	1.1-3	1-14 tod	1.48	2	38	3700	mascul	51	22668.97	178.45	81.8	136	89.5	85	220	48	140	122		0.5829

pescmae	prenda	pfumomae	paltmae	pgesta	pidgest	ppn	psex	pint	dmgtotdxa	daltura2012	dpeso	dsysmed	ddiamed	dglicose	dcolesterol	dhdl	dldl	dtrig	dECMICE	dECMICD
5	1 ou -	n,,o	1.5	4	38	3720	feminir	97	27459.39	159.5	71.5	109	68.5	102	211	56	134	111	0.58	0.58
4	1 ou -	n,,o	1.56	3	42	2300	mascul	31												
6	1.1-3	1-14 tod	1.62	3	38	3090	feminir	97												
7	3.1-6	n,,o	1.62	1	36	2800	feminino													
8	6.1-10	1-14 tod	1.64	2	38	3280	mascul	52	21829.54	177	77.4	136.5	77.5	84	139	51	77	53	0.5776	0.5873
9	3.1-6	1-14 par	1.62	2	38	3100	feminino		47213.17	166.1	90.3	119	82.5	60	219	74	130	89	0.582	
4	1 ou -	n,,o	1.6	3	40	3600	mascul	41	8958.259	169.8	61.5	123	66.5	114	130	51	70	42	0.5774	
4	1.1-3	n,,o	1.56	1	38	2250	feminino		31590.63	161.7	75.5	104	73	87	130	42	80	53	0.5785	0.5782
13	10	n,,o	1.6	2	40	3320	mascul	15												
5	1.1-3	15 + par	1.53	5		2130	mascul	88												
5	1.1-3	1-14 par		2	39	2900	feminir	96												
3	1 ou -	n,,o	1.57	2	41	2750	feminir	27	22689.07	166.2	64.6	97	57.5	72	173	70	90	44	0.5747	0.5696
16	3.1-6	n,,o	1.53	2	40	3610	mascul	41	40343.29	177.05	117.2	144.5	81.5	123	220	57	105	339		
4	1.1-3		1.64	3	41	3150	mascul	34												
19	10	15 + tod	1.71	3	42	4400	mascul	30												
6	1.1-3	n,,o	1.64	1	39	3760	masculin													
4	1.1-3	n,,o	1.61	10	39	2920	mascul	62												
3	1.1-3	n,,o	1.52	4	38	2270	mascul	39												
9	1.1-3	n,,o	1.57	3	40	2800	feminir	19												
1	1.1-3	n,,o	1.48	6	40	3060	mascul	70	26936.29	163.8	84.4	133	80	90	173	38	109	149		
16	1.1-3	n,,o	1.47	2	38	3990	feminir	36												
2	1.1-3	n,,o	1.46	3	39	3810	feminir	48	34981.02	165.65	77.8	100.5	71	60	125	34	64	123	0.5787	
4	1.1-3	n,,o	1.51	2	42	3500	mascul	20												
1	1.1-3	n,,o	1.51	5		3310	mascul	12												
7	1.1-3	1-14 tod	1.57	3	39	3420	feminir	26	18345.06	164.1	58.6	140	88.5	82	230	78	135	66	0.5811	0.5785
5	1.1-3	15 + tod	1.58	1		2250	masculin					156	95							
2	1.1-3	1-14 tod	1.53	3	43	2450	feminir	32	19902.72	158.7	60	120	67	104	148	46	90	73		0.5517
5	1.1-3	n,,o	1.57	5		3000	feminir	48	21102.57	160.25	66	104.5	67.5	89	134	56	69	46	0.5798	0.5699
11	10	n,,o	1.61	3	41	3730	feminir	37	25822.13	163.25	68.3	110	74.5	88	208	64	112	122	0.5725	0.5816
7	1.1-3	1-14 tod	1.52	1		2900	feminino		15264.12	150.35	47.5	117	74.5	47	108	42	55	47	0.5722	0.6049
3	1.1-3	n,,o	1.49	1	38	2600	feminino													
7	1 ou -	1-14 tod	1.6	1		3100	feminino		30646.75	169.7	74.1	101	64	76	200	64	122	91	0.5941	0.5788
9	3.1-6	n,,o	1.55	3		3400	feminir	15												
3	1 ou -	1-14 tod	1.65	6	37	3050	mascul	46												
9	6.1-10	n,,o	1.55	4	39	3800	feminir	83												
15	6.1-10	n,,o	1.68	1	36	3260	masculin					147.5	93.5	103	250	43	117	502		
0	1 ou -	n,,o	1.49	4		3180	feminir	25												
7	1.1-3	15 + par		2	37	3400	mascul	40												
6	1.1-3	1-14 tod	1.55	3		3400	feminir	27												
5	1.1-3	n,,o	1.63	6	41	3830	mascul	20		190.4	130.5	142	83.5	91	184	32	117	215	0.5813	0.5992
7	1.1-3	n,,o	1.59	3		3680	mascul	29												
0	1.1-3	n,,o	1.53	2		2750	feminir	72	36742.92	156.8	73.1	105	68	83	200	60	121	104	0.5782	0.5781
2	1 ou -	1-14 par	1.66	4	39	3000	feminir	35												
4	1.1-3	n,,o	1.61	4	39	3400	mascul	13	24078.76	177.4	84.5	124.5	84	81	215	64	128	125	0.5811	0.5767
3	1 ou -	n,,o	1.61	6		3920	mascul	12												
0	1.1-3	n,,o	1.58	5		3250	feminir	65	30177.42	164.4	73.3	127	83.5	81	147	40	96	63	0.5792	0.582
5	6.1-10	n,,o	1.57	5	38	3300	mascul	64												
18	10	n,,o	1.62	6	38	3400	feminir	21	26346.9	169.45	70.1	137	85.5	89	193	53	125	96	0.5819	0.577

pescmae	prenda	pfumomae	paltmae	pgesta	pidgest	ppn	psex	pint	dmgtotdxa	daltura2012	dpeso	dsysmed	ddiamed	dglicose	dcolesterol	dhdl	dldl	dtrig	dECMICE	dECMICD
8	3.1-6	n„o	1.52	1	41	4200	masculin		26667.97	174.8	87.5	109	71.5	65	168	46	100	116	0.6914	0.6007
0	1 ou -	1-14 tod	1.54	5		4350	mascul	24												
9	1.1-3	n„o		2	39	3500	feminir	33												
11	3.1-6	n„o	1.51	1	42	3320	feminino		26549.42	161.4	60.9	118	76.5	77	176	55	103	81		
10	3.1-6	1-14 tod	1.44	1	37	3370	feminino			157.8		116	81						0.5771	0.5785
8	1.1-3	15 + tod	1.59	1	42	3030	masculin		34709.54	168.6	104.5	154	86.5	98	212	62	135	81	0.6342	0.6031
5	1.1-3	15 + tod	1.52	3	39	3150	feminir	50	19977.3	159.6	59	123.5	70.5	88	196	95	82	113		
16	3.1-6	n„o		2	38	3470	mascul	24	9573.893	179	69.3	104.5	59.5	75	159	51	89	73	0.5781	0.577
1	1 ou -	n„o	1.53	3	40	2560	feminir	28	30647.94			123.5	73	89	185	73	90	100	0.57	0.58
9	1.1-3	n„o	1.55	1	41	3350	feminino		25936.15	163.3	70.9	103	64.5	84	184	65	101	57	0.5809	0.5787
3	1 ou -	1-14 tod	1.62	1	39	2900	feminino													
6	1.1-3	n„o	1.53	1	39	3090	masculin													
6	6.1-10	n„o	1.5	8		3450	mascul	60												
5	1.1-3	n„o	1.53	3	41	3400	feminir	14	8413.535	161.15	47.5	104.5	57.5	71	143	61	72	38		0.5665
4	1 ou -	n„o	1.46	1	43	2050	masculin													
7	3.1-6	n„o	1.52	4	41	3970	mascul	56	42664.38	175.1	111.8	159	93.5	80	219	54	137	150	0.5778	
7	3.1-6	15 + tod	1.61	1	40	2840	masculin													
7	3.1-6	n„o	1.6	1	39	3870	masculin		30466.58	169.95	87.5	146.5	85.5	112	180	52	112	90	0.6698	0.5934
7	3.1-6	n„o	1.63	2	36	3470	mascul	108	23942.53	172.95	87.1	123	66	74	200	60	121	79		
5	1.1-3	n„o	1.62	2	41	3850	mascul	84						86	196	42	138	66		
6	1.1-3	n„o	1.41	1		950	masculin													
6	3.1-6	n„o	1.52	1	40	3120	feminino		22178.55	162.75	63	111.5	74	88	237	85	137	68	0.4872	0.5631
5	1.1-3	n„o		6		1500	feminir	19												
6	1 ou -	n„o	1.6	1	38	3500	feminino		23589.93	161.6	62.1	105.5	67.5	79	168	77	76	105	0.5761	0.5722
7	3.1-6	n„o	1.58	2		2480	mascul	13	29522.22	175.4	88.9	139.5	85	85	238	54	159	140		
5	1.1-3	n„o	1.61	1	39	3200	feminino		27052.33	167.3	73	118	68.5	63	110	41	60	63	0.5783	0.579
13	1.1-3	n„o	1.63	1	40	3370	feminino		31174.09	169.6	70.7	103.5	68	86	180	55	104	129	0.5865	0.5743
4	1.1-3	n„o	1.68	1	41	3430	masculin		18880.92	183.45	82.2	135.5	73.5	105	164	44	106	90	0.5826	
5	1.1-3	n„o	1.45	4	32	1980	mascul	31												
3	1.1-3	n„o	1.6	1	39	3200	feminino			150.6	53.3	122.5	81.5						0.5643	0.5701
18	6.1-10	n„o	1.6	1	38	2800	masculin													
6	1 ou -	n„o	1.48	2		3750	mascul	19	15533.62	169.3	75.7	120	76.5	100	187	75	95	105	0.5853	0.5784
16	10	n„o	1.63	2	41	3330	feminir	58	24230.45	161.45	62.8	106.5	71	91	235	85	122	68	0.5814	0.562
8	1 ou -	n„o	1.52	1	41	3500	masculin		11935.93	179.5	72.3	118	71.5	86	193	49	128	52	0.6052	0.5967
5	1.1-3	n„o	1.6	2	40	3400	feminir	12	35972.86	159.6	77.9	115.5	82	93	194	64	107	106	0.5797	0.6154
0	1.1-3	n„o	1.53	5		2400	feminir	33	24457.74	152.8	59.6	125	75.5	102	235	66	138	159		0.5732
15	3.1-6	n„o	1.58	1	40	3650	masculin		24563.87	181.45	79.8	125	74	81	242	60	138	147		
8	6.1-10	n„o	1.6	4	37		feminir	88												
11	3.1-6	n„o	1.62	1	37	3690	masculin		32370.82	177.8	102.9	135	83.5	82	176	52	110	76	0.5806	
5	3.1-6	n„o	1.35	2	43	3750	mascul	37	13263.68	168.75	72.1	104	68	97	205	54	125	119	0.6107	0.6238
7	1.1-3	n„o	1.53	1	41	3450	masculin		5419.396	164.3	59.9	124	67	86	132	44	76	75	0.5739	0.5781
0	1.1-3	n„o	1.5	3		3140	feminir	28												
5	1.1-3	1-14 tod	1.54	1	39	2820	feminino													
4	1 ou -	n„o	1.54	4		2470	mascul	39												
6	1.1-3	1-14 tod	1.62	1	39	2800	feminino		11761.9	164.8	49.7	113.5	76	66	188	65	109	80		
4	1 ou -	n„o	1.42	5		3350	masculin													
10	1.1-3	n„o	1.62	1	37	2540	feminino		48869.24	167.55	96.4	134.5	92	75	167	71	76	196		0.575
1	1 ou -	15 + tod	1.54	1	38	2270	masculin		13310.77	162.75	54.3	127	85	97	200	47	136	115	0.71	0.63

pescmae	prenda	pfumomae	paltmae	pgesta	pidgest	ppn	psex	pint	dmgtotdxa	daltura2012	dpeso	dsysmed	ddiamed	dglicose	dcolesterol	dhdl	dldl	dtrig	dECMICE	dECMICD
5	1.1-3	n,,o	1.68	2	40	2940	mascul	75	20613.97	174	80.3	146.5	89	105	140	49	81	75	0.5761	0.577
5	1.1-3	1-14 tod	1.48	2	38	3680	feminir	23	15032.24	160	50.7	100	61.5	76	178	71	88	115	0.5718	0.5777
4	1.1-3	n,,o	1.53	1	38	3340	masculin		18748.27	166.2	78.3	129	84	119	197	38	90	330		
10	1.1-3	n,,o	1.64	2	37	2470	feminir	41												
15	1.1-3	n,,o	1.58	1	39	3380	feminino		23241.36	163.5	56.8	104.5	74	90	189	73	105	49	0.5783	0.5706
6	1.1-3	n,,o	1.46	4		3720	mascul	82	27365.63	166.75	86.8	128.5	78.5	106	256	44	161	168	0.6158	0.5789
12	1.1-3	n,,o	1.45	2	40	2560	feminir	11	19017.83	158.25	56.9	124	77.5	80	192	63	110	86	0.5786	0.5713
4	3.1-6	n,,o	1.56	6		3050	feminir	93	22821.45	161.4	57.7	107	73	76	250	74	160	88		0.5793
5	1 ou -	15 + tod	1.52	2	36	2630	feminir	10	14344.73	151	52.2	124.5	79.5	70	129	68	50	61	0.5784	0.5781
5	1.1-3	n,,o	1.56	4	38	3080	mascul	249	32996.68	170.35	93.6	131.5	83	79	147	42	91	84	0.5797	
2	1 ou -	1-14 tod	1.57	1	40	2840	feminino		39835.66	157.6	81.7	126.5	92.5	97	204	68	118	113	0.5773	0.5805
3	1.1-3	n,,o	1.61	2	29	1070	feminir	13												
4	1 ou -	1-14 tod	1.51	2	42	2780	mascul	29												
15	3.1-6	n,,o	1.58	1	39	2900	masculin		29777.72	176	102.6	145	84.5	91	153	45	90	80	0.5794	0.5701
0	1 ou -	1-14 tod	1.58	2		3280	feminino													
4	1 ou -	n,,o	1.47	4		3420	feminir	39	26653.75	161.9	71.3	113.5	72	63	160	57	91	79		
7	1.1-3	1-14 tod	1.46	2	40	3900	mascul	28	23433.15	168.8	80.4	131	78	101	239	59	143	212	0.5793	0.5784
10	6.1-10	n,,o	1.65	1	36	2700	feminino													
9	1.1-3	n,,o	1.63	2	40	3850	feminir	34	30623.28	159.75	70.2	112.5	71.5	77	168	65	85	63	0.579	
5	1.1-3	n,,o	1.62	2	38	3300	masculin		24973.23	177.6	82.7	125	75							
2	3.1-6	1-14 tod	1.58	2	36	3160	feminir	70	15297.52	157.6	53.5	114.5	72.5	73	124	30	80	53		
12	10	n,,o	1.58	3	40	3600	mascul	19												
5	3.1-6	n,,o	1.63	2	42	2680	mascul	11												
5	1.1-3	n,,o	1.59	1	39	3580	masculin													
17	3.1-6	n,,o	1.6	2	41	3570	mascul	44	23673.83	177.9	86.5	126	75.5	95	210	41	142	99	0.5883	0.5667
5	1 ou -	1-14 par	1.53	1		3340	feminino		20706.97	158.2	63.7	105	80.5	86	222	75	129	84	0.5781	0.5831
17	10	n,,o	1.64	1	33	1600	feminino		16647.15	168.65	52.8	111.5	67.5	87	195	81	95	98		0.5775
0	1.1-3	n,,o	1.48	1	40	2300	feminino													
7	1.1-3	n,,o	1.62	2		3700	feminir	29	17711.02	172.5	67.9	107.5	67.5	68	137	60	66	40		0.557
6	3.1-6	n,,o	1.6	4	39	3200	mascul	46	32648.96	169	89.3	123.5	86.5	75	166	79	78	40	0.5779	0.5779
5	1 ou -	n,,o	1.56	3	42	3100	mascul	67	13658.03	176.25	70.1	114	63	71	162	58	91	71	0.5815	0.5742
5	1 ou -	n,,o	1.54	1	42	3300	masculin													
3	1 ou -	n,,o	1.54	1	42	3380	masculin		29042.58	172.8	91.4	135	93.5	131	226	47	116	379	0.5813	0.5825
9	3.1-6	n,,o	1.55	1	33	1360	masculin		26439.1	175.6	82.6	132.5	87.5	77	175	44	103	128	0.627	0.5997
13	3.1-6	n,,o	1.55	1		3160	feminino		13919.19	159.15	46	96.5	63	89	148	49	84	66		0.5784
2	1.1-3	15 + tod	1.69	5	36	2650	mascul	35												
0	1 ou -	n,,o	1.64	6	39	2980	mascul	24												
8	1.1-3	n,,o	1.65	1	38	3160	feminino		19163.49	159.85	58.7	116.5	74	82	202	79	109	64	0.5705	0.5776
5	1.1-3	n,,o	1.56	4	41	3760	mascul	26	28202.98	177.95	92.2	118	74.5	82	173	49	113	87	0.5737	0.582
11	6.1-10	n,,o	1.67	2	39	2870	feminir	18	45271.8	174.2	102.4	131.5	89	80	189	66	105	109	0.5781	0.5758
12	10	n,,o	1.56	3	40	3880	mascul	104												
14	10	n,,o	1.58	2	39	2650	feminir	20	32507.68	152.35	65.8	112	75	96	240	79	137	114	0.5745	0.5751
4	1.1-3	n,,o	1.56	6	39	3100	mascul	147				153.5	101	257	372	20	80	1130		
4	1 ou -	1-14 par	1.67	2		3550	mascul	93		183.35	137.6	162.5	95	97	192	48	93	261	0.5819	0.5813
2	3.1-6	n,,o	1.49	2	38	3750	mascul	17	13562.25	162.2	72.8	116.5	75	85	227	74	138	62	0.6125	0.5666
8	1 ou -	1-14 tod	1.64	2	43	2620	feminir	34	11067.31	162	56	130.5	84	77	120	49	55	77	0.578	0.5796
4	1.1-3	n,,o	1.61	1	40	3250	masculin		2564.922	176.4	58.8	122	72	91	178	46	109	79	0.5783	0.5789
4	1.1-3	1-14 par	1.56	4	39	3320	feminir	23	30233.26	154.6	67.5	119	76.5	92	193	75	93	101		

pescmae	prenda	pfumomae	paltmae	pgesta	pidgest	ppn	psex	pint	dmgtotdxa	daltura2012	dpeso	dsysmed	ddiamed	dglicose	dcolesterol	dhdl	dldl	dtrig	dECMICE	dECMICD
12	1.1-3	n„o	1.6	4	40	2950	mascul	21	21671.52	181.8	84.8	127.5	85	94	210	55	134	113	0.6106	0.5675
2	1.1-3	n„o	1.52	2	40	3400	feminir	19												
9	1.1-3	n„o	1.59	3		1700	feminir	23	17398.73	160.9	53.2	126.5	87.5	108	229	89	129	58	0.5761	0.5709
8	1.1-3	1-14 par	1.51	1	39	3000	masculin		16329.41	180.4	79.6	119	73	80	150	46	94	78	0.5781	0.5783
4	1.1-3	1-14 tod	1.48	4	38	3200	mascul	12												
1	1 ou -	n„o	1.5	1	40	2870	masculin													
5	1.1-3	15 + tod	1.53	2		3100	feminir	28	29034.79	149.6	67.5	112	69.5	81	101	62	25	20	0.5576	0.5782
2	1.1-3	1-14 tod	1.55	3	39	3900	mascul	57												
5	6.1-10	n„o	1.6	1	42	3800	feminino													
3	1.1-3	n„o	1.62	3	40	2900	feminir	57	21388.08	167.25	59.6	110	75	100	174	67	99	36	0.5751	0.5779
3	1.1-3	n„o	1.57	3	38	3400	mascul	21												
12	6.1-10	n„o	1.64	3	40	3400	mascul	18												
7	1.1-3	15 + tod	1.68	3	38	3500	feminir	17												
16	10	15 + tod	1.61	3	37	2720	feminir	50		153.1	54.2									
0	1.1-3	n„o	1.46	2	41	3100	feminir	95	11900.59	152.45	46.9	109	72.5	86	142	70	63	56		
7	3.1-6	n„o	1.65	1	39	3270	masculin													
6	1 ou -	15 + par	1.48	2		3600	mascul	12	13940.89	157.2	61.1	137.5	77.5	101	162	46	96	135	0.5806	0.5922
0		1-14 tod	1.57	3	38	3750	mascul	81	6632.979	177	68.8	113.5	69	81	176	44	117	68	0.5785	0.5859
0	1 ou -	n„o	1.64	3	39	2300	feminir	55												
4	1 ou -	n„o	1.52	4	43	3260	feminir	11	20602.52	164.3	62.5	113.5	68.5	72	186	83	85	51	0.5768	0.5779
8	1.1-3	n„o	1.62	1	40	3200	feminino		28639.89	162.35	64.5	104	61.5	82	142	54	73	77	0.5769	0.5781
11	6.1-10	n„o	1.56	2	38	2650	feminir	33												
4	1.1-3	1-14 tod		6	40	2650	feminir	16	38784.83	166.4	76.6	119.5	72	64	174	75	84	59	0.5794	0.5784
1	1 ou -	15 + tod	1.51	7		2330	mascul	66	19199.6	164.7	67.8	130.5	79	80	165	44	95	141	0.578	0.5518
5	3.1-6	n„o	1.63	1	42	3600	masculin		21663.19	185.55	89.3	135	68	112	264	47	127	355	0.6264	0.5845
11	1.1-3	n„o	1.66	1	41	3920	masculin		26918.85	176.7	91.2	119.5	79.5	84	165	37	90	186	0.5772	
5	1.1-3	1-14 tod	1.55	8	36	2220	mascul	13	19954.93	172.1	75.1	127	80	85	229	61	142	204	0.5813	0.5897
16	10	n„o	1.57	2	38	3200	feminir	18											0.5794	0.5947
3	1.1-3	n„o	1.54	1		3240	masculin													
3	1 ou -	n„o	1.47	2	38	3050	feminir	17												
8	1.1-3	n„o	1.52	1	41	3700	masculin		23064.89	169.85	77.5	131.5	75	95	226	35	161	121	0.5962	0.6014
0	1 ou -	1-14 tod	1.5	8	39	2430	feminir	76												
6	1.1-3	n„o	1.55	5		2550	feminir	22	40689.38	166.15	88.9	123	82.5	105	214	70	120	138		
4	1.1-3	1-14 tod	1.58	4	29	980	mascul	45												
0	1.1-3	15 + tod	1.54	7	38	2850	mascul	18												
5	3.1-6	n„o	1.6	2	39	3750	feminir	49	18254.85	168.7	62.7	113.5	70	69	182	66	101	79	0.5839	0.5785
6	3.1-6	n„o	1.58	3	37	3390	mascul	120	37580.69	178.85	99.9	123	80	91	140	45	79	89	0.5788	0.5812
5	1.1-3	n„o	1.59	4	38	2970	feminir	121	39536.35	166	78.6	110	70.5	73	213	64	118	185		
4	1 ou -	15 + tod	1.49	4		2730	mascul	18												
7	1.1-3	n„o	1.65	1	39	3740	feminino		19928.04	171.1	63.1	139	96	89	249	92	128	199	0.5756	
16	10	n„o	1.56	3	41	3970	mascul	19	11927.02	169.8	71.4	113.5	73	83	213	66	109	245	0.5845	0.5407
4	1 ou -	n„o	1.61	7	38	2960	mascul	13												
7	1.1-3	15 + par	1.55	1	35	2100	feminino													
5	1.1-3	n„o	1.51	1	40	3250	feminino		28968.76	161.95	68	101.5	67.5	72	168	42	102	95	0.5847	0.5803
3	1.1-3	n„o	1.55	1	39	2780	masculin		6137.409	171.1	67.2	141.5	76	82	138	45	66	80	0.567	0.5783
7	1.1-3	n„o	1.5	1	41	3980	feminino		19777.27	159.15	54	115	75.5	70	250	85	130	192	0.5807	0.5762
1	1 ou -	n„o	1.59	3	39	3200	feminir	48												
1	1.1-3	n„o	1.65	1		3510	masculin		40687.2	176.1	105.2	124	68	78	230	33	145	303		

pescmae	prenda	pfumomae	paltmae	pgesta	pidgest	ppn	psex	pint	dmgtotdxa	daltura2012	dpeso	dsysmed	ddiamed	dglicose	dcolesterol	dhdl	dldl	dtrig	dECMICE	dECMICD
10	1.1-3	1-14 tod	1.69	3	36	3400	mascul	21		185.35	124.2	137.5	70.5	81	192	64	110	91	0.5824	0.5835
5	1.1-3	1-14 tod	1.44	1	40	2900	masculin													
0	1.1-3	n„o	1.54	6	38	2530	feminir	56	32418.89	154.4	75.7	123	79	91	226	57	146	178	0.57	0.56
9	3.1-6	1-14 tod	1.68	2	39	3500	feminir	19	53300.53	172.1	108.7	133	85	95	150	62	71	64	0.6182	0.5898
6	3.1-6	n„o	1.64	1		3930	masculin		27979.91	176.25	87.5	119.5	70.5	106	218	67	119	159		
5	1.1-3	n„o	1.51	4	40	3520	mascul	21												
5	1.1-3	n„o	1.52	1	41	2550	feminino		25728.88	150.85	60.7	96	65.5	99	199	78	100	96		
1	1.1-3	n„o	1.49	2	40	4270	mascul	16												
4	1.1-3	n„o	1.53	1	42	3450	feminino		19443.09	164	56	107.5	69	125	172	55	103	99	0.5782	0.5768
0	1.1-3	n„o	1.55	6		2700	feminir	16												
0	1 ou -	1-14 tod	1.55	5	40	3150	feminir	62	23021.77	162.5	66.7	126	78.5	76	186	64	111	57	0.5907	0.5783
5	1 ou -	1-14 tod	1.6	1	41	3950	feminino		27809.93	163.5	64.5	100	68	80	205	58	126	102	0.5555	0.5775
12	3.1-6	n„o	1.59	1	41	3500	feminino		19394.37	164.4	59.2	113	74	100	175	65	82	128	0.578	0.5598
3	1 ou -	n„o	1.56	3		2810	feminir	143												
12	3.1-6	n„o	1.52	2	40	3650	mascul	43	26341.69	163.5	78.5	121	77	65	215	70	115	181	0.5709	
17	10	n„o	1.59	3	40	3000	feminir	15	31957.27	158.8	74.2	115.5	76.5	128	280	100	150	189		
5	1 ou -	n„o	1.48	2		3520	mascul	11	18981.87	170	74.4	120	69.5	84	225	59	153	81	0.5805	0.58
5	1.1-3	n„o	1.52	1	37	2430	feminino		10861.12	153.1	47.9	93	54						0.5785	0.5781
5	1.1-3	1-14 tod	1.59	2	39	2800	feminir	22	14997.15	164.95	52.5	109	73.5	79	165	70	88	43		
6	1.1-3	n„o	1.73	1	40	3350	masculin													
15	3.1-6	n„o	1.63	1	40	3500	feminino		31069.13	175.7	83.3	117	77.5	83	213	46	147	130		0.5932
5	3.1-6	n„o	1.55	2	39	2950	feminir	26	15412.62	163.8	53.4	121	82.5	72	160	80	68	58		
9	1 ou -	n„o	1.6	2	41	3650	mascul	32												
3	1.1-3	n„o	1.51	3	42	2870	mascul	44												
11	6.1-10	n„o	1.55	3	41	3430	feminir	42	14762.61	158.3	57.9	113	69.5	71	205	78	113	67	0.577	0.5774
11	3.1-6	n„o	1.5	1	39	3560	feminino		21945.04	157.65	59.9	103.5	55	90	192	78	101	45	0.5754	0.5761
7	1.1-3	15 + tod	1.6	6	39	3610	mascul	18												
4	1 ou -	n„o	1.56	1	39	3470	masculin													
11	1.1-3	n„o	1.57	3	39	3400	mascul	25	8793.021	176.1	77.5	113	66	82	189	31	140	72	0.635	0.5977
9	3.1-6	n„o	1.56	3	40	3870	mascul	30	28088.41	169.2	92.7	128	78.5	78	178	52	102	80	0.5782	0.5798
3	1.1-3	n„o	1.53	2	37	3050	feminir	13												
5	3.1-6	15 + tod	1.55	2	41	3150	mascul	78	14978.97	176.65	69.1	133.5	81	92	130	48	69	50	0.5783	0.577
3	1.1-3	n„o	1.57	3	40	3530	mascul	30	21039.58	185.4	84.5	136	72	112	226	58	123	313	0.6461	0.5792
12	10	n„o	1.52	2	38	4000	mascul	197												
6	1 ou -	1-14 par	1.53	4	41	3900	feminir	35	12785.4	164.8	59.1	106	65	66	149	58	75	50	0.5781	0.578
9	3.1-6	n„o	1.59	1	37	3600	masculin		35340.29	177.8	89.5	137	91.5	97	178	63	100	94	0.5801	0.7202
8	1 ou -	n„o	1.37	8	40	3740	mascul	29												
3	1 ou -	1-14 tod	1.5	2		2990	feminir	12		165.85		111.5	64							0.5781
2	1 ou -	n„o	1.5	5		3750	mascul	21	2825.637	170.75	55.2	137.5	82	84	149	54	79	72	0.5789	0.5777
8	3.1-6	1-14 tod	1.6	1	37	2750	masculin		19414.22	185.6	85.7	110.5	67	80	185	70	94	127	0.5779	0.5814
10		1-14 tod	1.55	3	40	2910	feminir	60	37807.47	162.75	84.7	119	78.5	97	237	42	101	556	0.6303	0.5942
8	1.1-3	n„o	1.52	2	40	2860	feminir	49	9510.27	161.9	52.4	107.5	64	83	168	58	98	48	0.5781	0.5734
7	1.1-3	1-14 tod	1.55	2	38	3200	mascul	30	18400.67	175.05	76.3	115	74.5	128	219	49	105	369	0.5765	0.5687
10	1.1-3	1-14 tod	1.55	1	39	3200	feminino		44381.51	160.9	92.4	122.5	85.5	112	193	75	100	129	0.574	0.577
12	10	n„o	1.61	2		3350	mascul	60												
5	1.1-3	n„o	1.55	6	39	3220	mascul	74	18997.74	174.8	71.8	108.5	59	85	200	60	129	70	0.574	0.5686
5	1.1-3	1-14 tod	1.65	2	40	3130	feminir	16	19768.33	165	65.9	120.5	79	84	82	34	39	48	0.5781	0.5778
5	3.1-6	n„o	1.64	1	41	3700	masculin		21905.29	177.2	83.2	128	76	115	189	69	88	158	0.5639	0.578

pescmae	prenda	pfumomae	paltmae	pgesta	pidgest	ppn	psex	pint	dmgtotdxa	daltura2012	dpeso	dsysmed	ddiamed	dglicose	dcolesterol	dhdl	dldl	dtrig	dECMICE	dECMICD
10	1.1-3	n„o	1.54	2	40	3000	mascul	24												
5	3.1-6	15 + tod	1.54	3	40	2800	feminir	75												
7	1.1-3	n„o	1.5	3	40	3350	mascul	28	24662.62	170	87.6	126	72	92	208	52	123	147	0.5781	0.5772
12	1.1-3	n„o	1.53	1	40	3450	feminino		21271.28	154	55.1	109.5	64	81	172	64	88	96	0.5794	0.5741
3	1 ou -	1-14 tod	1.69	1		3150	masculin		20448.69	172.4	81.6	134	78	92	171	55	92	124	0.5829	0.6012
16	6.1-10	n„o	1.57	2	40	2950	feminir	72	50234.53	158.7	96.5	126	87.5	107	189	73	106	85		
9	3.1-6	1-14 tod	1.61	2	39	3180	mascul	49	13775.77	170.55	71.3	124	75.5	82	188	46	129	126	0.6368	0.5789
16	10	n„o	1.7	1	39	3450	feminino		32409.76	171.6	74.4	125.5	74.5	76	171	73	84	46	0.5774	0.5801
3	1 ou -	1-14 tod	1.53	4	40	3060	feminir	14												
10	10	n„o	1.64	4	39	3450	feminir	22												
2	1 ou -	1-14 tod	1.5	6	40	3350	feminir	30	20768.57	154.4	62.4	107	68.5	87	158	34	94	132	0.5795	
7	1.1-3	1-14 tod	1.64	2	36	3480	mascul	39												
11	3.1-6	n„o	1.64	1	40	3930	feminino		26880.69	160.1	67.5	121.5	83.5	97	239	71	144	216	0.5803	0.5781
16	10	n„o	1.57	3		3200	feminir	48	40089.3	160.5	79.7	100	63.5	80	202	62	127	72	0.5806	0.5781
8	1.1-3	n„o	1.54	1	39	3400	masculin		24847.2	173.7	76.7	123.5	82	92	215	47	134	162	0.6353	0.5787
5	1.1-3	n„o	1.57	2	39	3650	mascul	68	10620.88	186.1	70.5	116.5	72.5	86	157	42	98	72	0.5796	0.5769
6	1.1-3	n„o	1.65	2	41	3350	mascul	18	31341.14	183	97.4	129.5	85	79	241	63	156	92	0.5772	0.5779
7	1.1-3	1-14 par	1.6	1	40	3210	masculin													
4	6.1-10	n„o	1.53	9	40	2980	feminir	68	44858.25	163.75	93.9	108.5	72	85	189	69	106	56		0.5793
4	1.1-3	1-14 tod	1.57	3	40	2750	mascul	11	11627.49	170.55	72.9	115	65	89	190	49	120	106	0.5799	0.6594
7	3.1-6	1-14 tod	1.61	3		3000	mascul	120	21014.87	174.2	72.6	100.5	66	62	200	64	117	110	0.5717	0.578
6	1 ou -	n„o	1.57	2	41	3600	feminino		21702.96	154.15	57.5	100.5	68.5	83	190	72	97	130	0.571	0.5778
5	1 ou -	n„o	1.53	1	38	3710	masculin		10157.58	169.6	73.4	137	74.5	93	220	52	153	60	0.5916	0.6108
5	3.1-6	n„o	1.52	4	39	2820	mascul	105												
0	1 ou -	n„o	1.56	1		2650	feminino													
0	1 ou -	n„o	1.5	7	43	4250	feminir	36	26467.82	158.6	70.1	118.5	73.5	87	138	60	70	56	0.5813	0.5873
2	1.1-3	n„o	1.57	4	39	4050	feminino		50637.56	161.25	104.3	127	85	105	184	51	120	78	0.5921	0.6053
9	1 ou -	1-14 tod	1.59	3		3440	mascul	18												
5	1.1-3	n„o	1.62	1	39	3600	feminino		27155.63	166.9	67.7	100	62	82	202	70	111	100	0.5792	0.6015
9	1.1-3	15 + tod	1.54	3	42	4350	mascul	12												
6	1.1-3	1-14 tod	1.52	1	39	2820	masculin													
8	1 ou -	n„o	1.57	1	41	3050	masculin		19636.87	177.3	77.9	135	79	86	175	62	97	86	0.5841	0.565
5	3.1-6	n„o	1.54	3	39	2850	mascul	106	32690.4	165	88.2	140	88.5	216	224	44	126	418	0.5784	0.5894
3	3.1-6	n„o	1.52	2	38	3320	feminir	209	25367.24	166.35	63.9	114.5	68	86	272	94	153	152	0.5715	0.5615
3	1 ou -	n„o	1.49	13	40	3650	feminir	48												
12	3.1-6	n„o	1.57	4	36	2370	feminir	16												
5	1.1-3	1-14 par	1.5	1	38	2980	feminino		37413.79	157	82.7	110.5	76.5	82	155	46	86	92		
13	1.1-3	n„o	1.65	1	40	4000	feminino		18750.01	168.9	58.4	106	67	81	199	75	111	51		
8	1 ou -	n„o	1.52	1	39	3300	feminino		24643.69	157.5	64.4	93.5	63.5	70	171	56	102	83	0.5749	
6	1 ou -	n„o	1.55	1	40	2950	feminino			152.85	76	119	77	150	176	50	100	117	0.5474	0.5579
6	1.1-3	15 + tod	1.59	5	41	3780	mascul	80	9183.137	176.1	70.2	115	62.5	110	178	43	86	289	0.5765	0.5783
2	1 ou -	n„o	1.58	1	39	3700	feminino													
4	1 ou -	1-14 tod	1.5	1		3150	feminino		22668.76	148.85	58.3	128	79.5	82	135	46	70	69	0.5758	0.577
4	1.1-3	n„o	1.58	2	38	3200	mascul	27	4065.651	176.95	60.7	117	75	85	195	64	117	62	0.5781	0.577
4	3.1-6	n„o	1.58	4	39	3750	mascul	36												
5	3.1-6	1-14 par	1.61	3	40	3600	feminir	57	20092.12	168.4	60.7	107	67.5	85	224	86	111	123	0.5785	0.5743
0	1.1-3	n„o	1.47	5	42	3600	mascul	30	8278.233	167.2	58.3	129	73.5						0.5769	0.5577
7	1.1-3	n„o	1.63	1		2450	feminino													

pescmae	prenda	pfumomae	paltmae	pgesta	pidgest	ppn	psex	pint	dmgtotdxa	daltura2012	dpeso	dsysmed	ddiamed	dglicose	dcolesterol	dhdl	dldl	dtrig	dECMICE	dECMICD
13	10	n„o	1.8	3	40	3400	mascul	21		201.2	117.9	137.5	93.5	96	209	72	127	59	0.5843	0.5683
3	1 ou -	15 + tod	1.55	5	40	2750	mascul	17	33504.59	177.85	95.1	113.5	69.5	103	225	39	128	269	0.5787	0.5753
3	1.1-3	n„o	1.51	3	39	2970	feminir	138	25155.34	150.5	62.5	122	80	89	169	63	90	95		
4	1.1-3	n„o	1.69	1		3050	masculin		4225.177	178.2	58.3	139.5	90.5	85	174	85	71	77	0.5517	0.5798
6	1.1-3	n„o	1.72	2	39	4100	mascul	21	18745.04	179.95	90.2	135	74.5	65	139	53	70	56	0.5785	0.5578
12	1.1-3	n„o	1.54	1	41	2800	masculin													
3	1.1-3	n„o	1.68	5	39	2950	feminir	58												
4	1.1-3	n„o	1.45	9	39	3430	mascul	13												
5	1 ou -	15 + tod	1.55	3	41	3300	feminir	48												
4	1.1-3	n„o	1.48	3	40	2870	mascul	90	18771.47	169.15	68	131	89	80	202	41	104	267	0.5743	0.5731
4	1.1-3	n„o	1.64	6	38	3050	feminir	67												
0	1.1-3	n„o	1.47	3		2850	mascul	17												
4	1.1-3	15 + tod	1.59	4	40	3100	mascul	37	8364.94	164.6	53.5	121.5	70.5	88	204	49	129	125	0.5699	0.5724
2	1.1-3	n„o	1.63	3	40	2960	mascul	109	6930.587	179.35	59.5	130	76.5	86	190	60	100	129	0.5826	0.585
5	1.1-3	15 + tod	1.61	6	36	2450	feminir	111												
18	10	15 + tod	1.53	3	37	2940	mascul	83												
3	1 ou -	n„o	1.52	4		3020	mascul	12												
8	1.1-3	n„o	1.58	1	39	3070	masculin		21902	169.45	74.1	137.5	90	98	180	58	97	202	0.5786	0.5521
6	1.1-3	n„o	1.55	2	37	2960	mascul	35	4790.36	182.1	70.5	118.5	77.5	82	126	48	60	87	0.5804	0.5787
7	1.1-3	1-14 tod	1.45	2	39	2800	mascul	20	24780.47	168	79	158.5	88	87	195	50	113	157	0.5795	0.5772
5	1.1-3	1-14 tod	1.52	3	40	3000	feminir	17	30477.7	176.35	80.6	113.5	76.5	90	190	63	110	79	0.5781	0.5695
0	1.1-3	1-14 tod	1.58	6	37	2930	mascul	65												
7	1.1-3	n„o	1.61	2		2780	mascul	13												
3	1.1-3	n„o	1.51	5	40	3250	feminir	46	42912.5	163.3	96.4	111	65	76	200	50	132	98	0.5788	0.577
7	10	n„o	1.55	3	39	3230	feminir	16												
5	3.1-6	n„o	1.53	4	43	4600	mascul	74		168.2	124	146	91	108	200	39	119	340	0.5785	0.58
7	1.1-3	1-14 par	1.49	2	40	2800	feminino													
4	1 ou -	n„o	1.67	1	40	3650	feminino		43678.44	169.3	94.9	125	76	93	175	52	109	79	0.5729	0.5802
8	3.1-6	n„o		1	39	3860	masculin													
3	1 ou -	n„o	1.59	4	37	3350	mascul	15	12540.01	170.8	74.8	124.5	72.5	90	216	76	120	99	0.5766	0.5743
8	1.1-3	n„o	1.55	1	37	3720	masculin		34741.99	175.1	95.7	128	88.5	90	203	46	123	192	0.5659	0.5742
8	1.1-3	n„o	1.54	3	40	3550	mascul	57	23014.98	177.55	83.1	141.5	87	90	174	58	95	113	0.6401	0.5807
18	10	n„o	1.61	1	39	2800	masculin		4470.166	168.95	57.2	130.5	77.5	92	204	60	135	71		
5	1.1-3	1-14 tod	1.54	2	38	3350	feminir	48	15189.81	171.4	51.6	136	98.5	107	175	89	69	76	0.578	0.5717
3	1.1-3	n„o	1.53	3	37	3350	mascul	55												
5	1.1-3	n„o	1.43	1	39	2560	masculin													
8	3.1-6	n„o	1.48	2	38	3100	mascul	40												
4	1 ou -	n„o	1.55	5		2700	feminir	20	25126.6	158.65	67	136.5	90	64	220	76	119	143	0.6824	0.5694
2	1.1-3	1-14 tod	1.5	5	39	3800	mascul	23	33593.44	178.6	98.3	106.5	73	79	127	33	70	123	0.5958	
4	1 ou -	n„o	1.29	2	34	2220	feminir	12	15475.25	151.3	52.7	113.5	77.5	83	180	70	104	40	0.5778	0.578
10	10	n„o	1.63	1	40	3780	masculin		20154.92	177.2	85.4	141.5	84	83	200	64	109	141	0.5754	
16	6.1-10	n„o	1.65	1	40	3750	feminino													
9	1.1-3	15 + tod	1.54	3	42	3560	mascul	11	25244.87	175.75	104.5	139	79	74	129	31	60	174	0.5801	0.5856
2	1 ou -	1-14 tod	1.55	1		2900	feminino		5618.376	165.6	46.6	118	72.5	86	192	49	100	192	0.5782	0.5872
0	1.1-3	n„o	1.55	2	39	3580	mascul	30	13469.53	183.6	68.4	135	75	90	195	89	91	61		0.5672
3	1 ou -	1-14 tod	1.45	7	42	3570	mascul	57	15154.39	165.65	67.2	116	77.5	88	265	59	195	79	0.5804	0.5738
10	3.1-6	n„o	1.54	2	39	3700	feminir	79												
16	10	n„o	1.56	4		4100	feminir	87												

pescmae	prenda	pfumomae	paltmae	pgesta	pidgest	ppn	psex	pint	dmgtotdxa	daltura2012	dpeso	dsysmed	ddiamed	dglicose	dcolesterol	dhdl	dldl	dtrig	dECMICE	dECMICD
8	3.1-6	n„o	1.5	2		3300	feminir	11												
12	1.1-3	n„o	1.55	1	40	2850	masculin		6715.246	172.5	56.7	122.5	69.5	84	191	63	91	167	0.5636	0.556
11	6.1-10	n„o	1.56	3	41	3150	mascul	44	15823.27	171.25	75.8	125	82	80	213	55	138	72	0.5862	0.587
11	3.1-6	1-14 tod	1.54	2	38	3400	feminir	16												
12	1.1-3	n„o	1.57	2		2740	mascul	52	15681.02	178	70.6	131.5	78.5	93	185	60	111	93	0.5632	0.5786
4	1.1-3	n„o	1.52	2	41	3830	feminir	47	12280.18	166	54.4	103	59	80	165	63	90	50	0.5714	0.578
7	6.1-10	n„o	1.59	2	31	1700	feminir	45												
5	3.1-6	n„o	1.56	2	43	3350	mascul	113												
7	3.1-6	n„o	1.53	3	41	2920	feminir	91												
5	1.1-3	n„o	1.54	4	41	3610	mascul	17	20370.78	169.35	79.8	119	76.5	81	183	41	103	292	0.5878	0.6123
4	1.1-3	n„o	1.65	2		1980	feminir	9												
3	1.1-3	n„o	1.58	2	41	3430	feminir	19	28142.76	171.9	74	106.5	65.5	77	183	44	106	197	0.5914	0.5938
5	3.1-6	n„o	1.55	2	41	2830	mascul	43	10966.42	172.55	63.1	110	58.5	96	185	42	112	157		0.5349
3	1.1-3	1-14 tod	1.49	3		1050	mascul	14												
12	10	n„o	1.56	1	41	3870	masculin													
17	3.1-6	15 + tod	1.68	1	41	3800	feminino		32406.3	166.5	79	117.5	80	70	187	72	99	62	0.5794	0.5888
0	1 ou -	1-14 tod	1.55	2		750	feminino													
12	3.1-6	n„o	1.51	2	41	2710	feminir	46	19874.27	154.3	57.1	125	77	82	158	60	64	177		
5	6.1-10	n„o	1.53	3		2550	feminir	25												
2	1 ou -	15 + tod	1.65	1	39	2630	masculin					151.5	88.5							
5	1 ou -	n„o	1.46	1		3110	masculin													
9	6.1-10	1-14 tod	1.61	1	41	3700	feminino		17842.92	161	56.6	94	63.5	77	215	79	109	138	0.5722	0.5645
2	1 ou -	1-14 tod	1.54	3		2400	mascul	57	5076.367	167.8	59.6	136	73.5	92	201	48	117	172	0.5782	0.5797
7	1.1-3	n„o	1.7	2		2650	feminir	59		159.05	48.2	116	78	97	159	68	77	65		
3	1 ou -	n„o	1.58	3		4800	feminir	65	25854.41	174.5	72.9	118	80	78	211	71	124	83		
5	3.1-6	n„o	1.59	1	36	2900	masculin		12236.4	168.4	62.5	117	78	88	234	62	154	86		0.5745
5	1.1-3	n„o	1.63	4		3150	mascul	141	33296.72	171.65	99.2	143	96.5	95	195	57	113	111	0.5797	0.5781
5	1 ou -	n„o	1.55	1		3600	feminino		28770.62	166.05	68.7	118	78.5	72	195	59	111	139	0.5789	0.5794
5	1.1-3	1-14 tod	1.58	2	41	2900	feminino		21705.31	160.8	63.2	116	66	87	172	52	94	93		0.5699
17	10	1-14 par	1.68	2		3150	mascul	23	10512.35	175.9	68.7	127.5	65.5	84	168	70	82	87	0.5781	0.5694
4	1 ou -	n„o	1.48	1		3560	feminino		27388.66	158.3	67.5	98	64.5	94	190	65	106	78	0.5717	
18	3.1-6	n„o	1.63	3	41	3430	mascul	24												
8	1 ou -	1-14 tod	1.55	1		2300	feminino													
4	1 ou -	n„o	1.59	4	42	3470	feminir	53	30451.88	167.05	69	122	79.5	79	167	62	87	79	0.6062	0.5866
8	1.1-3	n„o	1.67	3	39	2800	feminir	16	30000.68	173.4	72.1	106	71	107	238	75	150	79	0.5733	0.5735
16	10	n„o	1.75	1	38	3370	masculin		32214.25	187.05	101.2	141.5	84.5	100	199	63	113	123		0.5762
5	1.1-3	1-14 tod	1.5	3	41	3800	mascul	25	17491.6	175.1	84.4	141	79	96	281	54	193	91	0.5982	0.5783
4	1.1-3	n„o	1.5	2	38	3280	feminir	53	37995.54	156.5	77.7	123.5	88	98	200	74	103	137		
9	3.1-6	n„o	1.65	2	40	2780	feminir	43												
3	1.1-3	n„o		4	39	3750	masculin													
5	3.1-6	1-14 tod	1.58	2	39	3250	feminir	55												
2	1 ou -	n„o	1.52	2	38	3300	mascul	12		175.15	120.1	141.5	88.5	79	131	36	75	130	0.599	0.5966
9	6.1-10	1-14 tod	1.63	1	41	3420	masculin			176.85	123.9	147.5	93	81	135	47	71	81	0.5809	0.5811
0	1.1-3	1-14 tod	1.57	6		3900	feminir	36	20741.66	162.65	60.9	101.5	62.5	82	215	78	119	87	0.578	0.5786
7	6.1-10	n„o	1.57	4		3750	feminir	202	18387.97	170	57.5	113.5	69.5	75	172	80	78	76	0.5755	0.5781
12	3.1-6	n„o	1.58	1	39	3650	feminino													
9	1.1-3	1-14 tod	1.59	5	40	2820	masculin													
6	1.1-3	1-14 tod	1.55	1	39	1640	masculin		17246.59	171.35	82.3	155.5	87	101	148	34	88	160	0.5943	0.5823

pescmae	prenda	pfumomae	paltmae	pgesta	pidgest	ppn	psex	pint	dmgtotdxa	daltura2012	dpeso	dsysmed	ddiamed	dglicose	dcolesterol	dhdl	dldl	dtrig	dECMICE	dECMICD
9	3.1-6	n„o	1.62	4	39	3000	feminir	18												
6	1.1-3	n„o	1.59	4	39	3850	mascul	58	17635.27	174.95	76.9	129.5	89	79	206	42	111	298		
11	1.1-3	n„o	1.55	1	39	3850	feminino		29108.01	153.5	67.6	100	60.5	102	209	51	140	99		
16	10	n„o	1.65	2	38	2900	feminir	56	43498.57	166.2	84.6	125.5	89.5	78	206	69	116	161	0.5786	0.5781
7	10	n„o	1.55	3	41	3260	mascul	54	21955.32	175.35	81.4	132	75.5	101	208	53	127	238	0.5807	0.5771
0	1 ou -	n„o	1.56	7	42	4520	mascul	120												
5	1 ou -	1-14 tod	1.53	5	39	2840	mascul	33												
6	1.1-3	1-14 tod	1.62	3		3300	feminir	14	23356.11	158.05	59.6	125.5	79.5	88	182	52	101	89	0.5781	0.5791
8	1.1-3	n„o	1.47	4	39	3650	mascul	14	24998.54	164.15	82.5	128	73.5	65	194	38	129	127	0.6575	
15	10	n„o	1.57	4	39	3460	mascul	30	5255.369	175.1	64.2	128.5	77	87	146	46	90	73	0.5825	0.5744
4	1.1-3	n„o	1.61	2	37	3200	mascul	72	39153.3	179.6	102.8	126	79	70	228	53	145	153	0.5808	0.5752
2	1.1-3	n„o	1.51	4	37	3900	feminir	18	27570.41			103	72	95	178	60	92	123		0.5762
6	3.1-6	15 + tod	1.66	4		1900	mascul	78	26472.22	183.3	91.8	121.5	78	75	180	40	114	130	0.573	0.578
11	10	n„o	1.6	1	39	3650	feminino			167.4		106	68.5						0.5954	0.5773
8	1.1-3	n„o	1.5	3	38	3630	mascul	33	17629.9	180.1	83.7	110	65	127	180	61	106	74	0.5344	0.5739
5	3.1-6	1-14 tod	1.51	3	39	3100	mascul	72	15036.12	176.65	79.2	124.5	65.5	96	158	59	80	101	0.578	0.5701
3	1.1-3	n„o	1.59	4	40	2940	mascul	72	12069.19	165.1	55.5	120.5	81	86	228	72	135	125	0.5816	0.5959
6	1.1-3	1-14 tod	1.53	2	40	3400	mascul	19	29920.91	179.2	99.6	120	78.5	97	195	46	108	211	0.6339	0.5974
7	1.1-3	n„o	1.58	1	38	3300	feminino		35855.39	163.7	83.1	132	82.5	69	138	48	79	74	0.5784	0.577
8	3.1-6	1-14 tod	1.5	2	39	3050	feminir	72	22534.29	154.65	57.9	113	71	102	146	36	63	337	0.5789	0.5786
2	1.1-3	n„o	1.55	8		3150	mascul	44		175.95	129.6	139.5	84	107	146	43	80	117	0.5829	0.5896
8		n„o	1.6	1	41	2980	masculin		26957.99	176.8	89.3	145	101	93	208	57	137	73	0.5753	0.5844
20	10	n„o	1.62	2	37	2900	feminir	16	11064.55	166.75	57.4	111	66	91	158	70	75	52	0.5781	0.5782
			1.47	1		3130	masculin		11078.26	159.6	62.2	132	78	89	197	55	128	58	0.58	0.5792
6	1.1-3	n„o	1.6	1	39	2630	masculin		13987.04	179.9	71.6	120	63.5	91	158	36	109	64	0.5781	0.5784
8	1.1-3	n„o	1.53	4		3760	feminir	26												
12	10	1-14 par	1.63	1	41	3460	feminino													
16	10	n„o	1.74	1	41	4110	masculin			191	77.2	115	70.5	63	190	63	113	49	0.5929	0.5817
7	1 ou -	1-14 par	1.57	1		1430	masculin		18920.92	168.7	72.1	126.5	77.5	90	216	49	130	198	0.5804	0.566
9	3.1-6	1-14 tod	1.54	1	41	3800	feminino		16004.04	165.25	66.2	121	69	84	201	50	133	69	0.5803	0.585
8	1.1-3	15 + tod	1.61	2	39	3790	mascul	90												
6	1.1-3	n„o	1.5	2	42	3310	feminir	11	18090.68	161.7	55.2	125	78.5	77	147	59	82	49	0.5758	0.573
8	3.1-6	1-14 par	1.64	5		1160	feminir	12												
4	1.1-3	n„o	1.47	2	40	3570	feminir	30	15726.08	160.3	57.2	90.5	54.5	76	206	68	110	128	0.5783	0.5785
2	1.1-3	n„o	1.57	2	41	3470	mascul	23	22658.32	176.8	84.7	136	73.5	77	151	44	92	97	0.5933	0.5997
2	1.1-3	1-14 tod	1.52	2	41	2950	mascul	143	6770.915	166.1	59	160.5	86.5	123	147	59	71	94	0.5784	0.5722
5	1 ou -	1-14 tod	1.56	2	40	2530	feminir	22	23679.01	160.55	64.2	120.5	84	101	179	68	98	53	0.5803	0.5782
5	1.1-3	n„o	1.56	6	39	3630	feminir	94	42767.61	166.4	91.6	113	74.5	86	164	49	107	50	0.5888	0.5786
3	1.1-3	n„o	1.59	2		3100	feminir	16	20426.9	162.3	58.7	123	76.5	76	172	62	97	88	0.5799	0.5787
0	1.1-3	n„o	1.55	15	40	3900	feminir	11	26581.48	156.8	63.1	100.5	65.5	84	166	70	84	51	0.5753	0.5922
9	3.1-6	n„o	1.48	2	39	2840	feminir	39												
5	1.1-3	n„o	1.49	3	42	3350	mascul	99	11614.75	163.9	63.4	115.5	74	97	169	50	100	125	0.5784	0.5764
17	10	n„o	1.71	4	39	3750	feminir	39	19384.97	176.65	62.2	101	68.5	90	168	75	79	57		0.5459
12	3.1-6	n„o	1.51	3	41	3050	feminir	25												
3	1 ou -	1-14 tod	1.7	1	40	3050	feminino		22339.43	164.8	62.9	104.5	70.5	97	195	72	109	56	0.5778	0.578
5	1 ou -	1-14 tod	1.55	2	40	3050	mascul	26	17062.69	169.1	72.4	151.5	93	92	228	68	136	93	0.579	
15	6.1-10	n„o	1.56	2	39	2850	feminir	14	39061.79	153.4	75.8	129.5	83	90	221	63	116	258	0.5785	0.5801
16	10	n„o	1.54	5		1950	feminir	24	13458.38	158.8	50.6	103	59	79	140	70	60	35	0.5774	0.5781

pescmae	prenda	pfumomae	paltmae	pgesta	pidgest	ppn	psex	pint	dmgtotdxa	daltura2012	dpeso	dsysmed	ddiamed	dglicose	dcolesterol	dhdl	dldl	dtrig	dECMICE	dECMICD
8	1.1-3	n„o	1.54	1	40	3750	feminino													
3	1 ou -	n„o	1.49	1		2450	feminino													
1	1 ou -	15 + tod	1.53	2		2910	mascul	16												
1	1 ou -	n„o	1.62	1	41	3400	feminino													
5	1.1-3	1-14 par		1	40	4300	masculin		11490.53	175	77.4	119	65	80	121	43	65	73	0.578	0.5799
4	1 ou -	1-14 tod	1.6	2	38	2780	feminir	15												
4	1 ou -	n„o	1.54	5	37	3350	mascul	19												
11	1.1-3	n„o	1.53	9	39	3170	feminir	33												
2	1 ou -	1-14 tod	1.61	1	40	3430	masculin		40539.89	176.4	110.8	134	89.5	87	229	61	144	139	0.6024	0.5913
11	1 ou -	n„o	1.57	1	37	3520	masculin													
8	3.1-6	n„o	1.57	2	35	2300	feminir	19	36929.11	161.25	73.8	113	71.5	74	148	60	83	45	0.5783	0.5788
12	3.1-6	n„o	1.5	1	39	3350	feminino		20000.92	161.35	59.2	100.5	74	73	249	64	163	131	0.578	0.5821
4	1.1-3	n„o	1.6	5	41	4300	mascul	24	25832.12	179.6	96	126.5	73.5	90	182	37	96	235	0.5896	0.5844
4	1.1-3	n„o	1.62	4	40	3150	feminir	45	23825.7	169.85	68.5	107	69	84	209	63	127	61	0.5797	0.5783
5	1.1-3	n„o	1.58	2	41	2700	mascul	19												
14	6.1-10	n„o	1.63	1	41	3900	feminino		38841.54	167.3	83.1	117.5	73	82	220	74	123	75	0.5778	0.5794
10	1.1-3	n„o	1.63	1	39	3200	masculin													
7	1.1-3	n„o	1.61	1	39	2550	feminino		35425.35	160.15	77.9	104	70	79	148	66	75	41		
3	1.1-3	n„o	1.63	3	40	3270	mascul	39												
6	3.1-6	n„o	1.61	1	40	3300	feminino		24551.28	159.55	62.8	103	67.5	68	154	44	97	52	0.5781	0.5782
11	1 ou -	1-14 tod	1.61	3		2490	masculin		17615.2	178.6	77.6	129	85	80	175	45	115	64	0.5783	0.5781
8	1 ou -	15 + tod	1.58	2	37	2800	feminir	50												
5	1.1-3	1-14 tod	1.56	2		2600	mascul	33	24867.44	166.8	78.2	144	90	88	250	67	164	108	0.5831	0.6439
2	1.1-3	n„o	1.53	1	39	3180	feminino													
3	3.1-6	n„o	1.61	2	38	3000	feminir	162	34070.2	169.6	78.5	120.5	75.5	74	216	90	103	112	0.7557	0.6367
10	1.1-3	1-14 tod	1.69	3	36	2100	mascul	21	33655.02	178.4	105.3	122.5	62	97	175	45	98	166	0.5746	
7	1.1-3	n„o	1.62	1	41	4000	masculin													
8	1.1-3	1-14 tod	1.58	1	35	1900	masculin		22142.02	170.6	83.6	117.5	74.5	96	177	41	103	189	0.5747	0.5983
13	10	n„o	1.54	1	40	3600	feminino		12379.13	160.95	44.6	105	71.5	81	189	79	94	66	0.578	0.5778
7	3.1-6	n„o	1.73	2		3230	feminir	26	47475.18	171.6	103.6	141	95.5	94	183	51	91	210	0.6328	0.6058
17	1.1-3	1-14 par	1.57	2	42	3280	feminir	87	52345.2	162.05	100.3	118	83	117	213	38	113	282	0.5924	
4		n„o	1.52	4	40	3650	feminir	20	19073.87	160.9	56.5	124	84.5	106	222	53	154	91	0.575	0.5842
5	3.1-6	n„o	1.52	1	40	3730	masculin													
5	1.1-3	15 + tod	1.67	4	39	3300	feminir	33												
7	1 ou -	n„o	1.65	1	40	4150	masculin													
8	1.1-3	n„o	1.63	4	39	3900	feminir	21	22282.61	168.55	64.8	111.5	71.5	78	211	78	98	204	0.5689	0.5781
5	1.1-3	15 + tod	1.57	3	43	3400	feminir	50												
9	3.1-6	1-14 tod	1.55	1	40	3060	feminino													
6	3.1-6	n„o	1.6	4	34	1990	mascul	31	41602.33	179.5	107.7	155	84	98	215	64	123	142	0.5932	0.5811
7	6.1-10	n„o	1.58	3	40	3350	mascul	36	15202.71	173.75	68.1	132.5	89.5	68	200	67	110	181	0.635	0.5844
5	6.1-10	n„o	1.62	4	39	3850	mascul	21												
5	1.1-3	1-14 tod	1.61	2	39	2750	mascul	11	19543.88	175.6	80.4	135.5	75.5	84	192	34	125	187	0.5646	0.5535
5	1.1-3	1-14 tod	1.53	1	39	2700	masculin													
5	1.1-3	n„o	1.47	4	37	5200	feminir	37	37832.5	161.4	83.7	116.5	73.5	88	188	53	121	50	0.5731	0.582
6	1.1-3	n„o	1.68	1	40	4000	masculin		24452.91	182.05	89.8	133	80.5	86	200	49	128	107	0.6323	0.5795
8	1.1-3	1-14 tod	1.58	1	35	1900	masculin													
8	1.1-3	1-14 tod	1.7	1	38	2750	masculin		25034.9	174.15	78.5	131.5	76.5	82	179	47	110	149	0.5745	0.5738
2	1.1-3	1-14 tod	1.57	1	39	2450	feminino		36231.73	162.3	77.2	117	74	89	254	74	156	133	0.5784	

pescmae	prenda	pfumomae	paltmae	pgesta	pidgest	ppn	psex	pint	dmgtotdxa	daltura2012	dpeso	dsysmed	ddiamed	dglicose	dcolesterol	dhdl	dldl	dtrig	dECMICE	dECMICD
16	3.1-6	n„o	1.6	1	41	3060	feminino													
17	10	1-14 par	1.69	2	41	4200	mascul	28	18945.88	182	88.2	128.5	77	87	171	41	103	142	0.5784	0.5773
11	3.1-6	n„o	1.7	1	38	2970	feminino		27039.1	168.7	65.8	124	73	87	145	60	73	49	0.5739	0.5788
4	1.1-3	1-14 tod	1.56	4	39	3280	mascul	43												
0	1 ou -	1-14 tod	1.6	4		3500	feminir	24	37415.86	157.7	78	109.5	77	84	173	70	90	80	0.578	0.5783
6	1.1-3	1-14 tod	1.65	1		3270	feminino		16412.68	159.15	55.2	110	65	96	162	60	95	66	0.5665	0.5796
16	10	1-14 tod	1.61	4		2500	feminir	24												
4	1.1-3	1-14 tod	1.53	3	37	3450	mascul	23	22054.64	181.3	84.2	128.5	71.5	78	186	64	110	48	0.5788	0.5785
17	10	15 + tod	1.7	3	38	3500	feminir	13												
15	1.1-3	n„o	1.49	1	35	1550	feminino		26582.17	159.4	63	119.5	76	98	242	79	120	264	0.5784	0.5714
14	6.1-10	n„o	1.56	1	40	3400	masculin		17008.87	179.8	74.6	128	78.5	76	243	60	146	179	0.5762	0.5778
6	1.1-3	n„o	1.54	2	39	3250	feminir	127	44104.46	157.6	86.3	132.5	97	82	190	51	120	109		
5	1.1-3	n„o	1.54	2		3550	feminir	62	21685.47	159.15	63	104	59	100	183	63	98	77	0.5789	0.5797
13	3.1-6	n„o	1.68	1	40	3050	feminino													
17	6.1-10	n„o	1.66	1	40	3650	masculin		30451.97	176.8	92.5	126	81	95	299	57	194	303	0.5941	0.5751
5	1.1-3	n„o	1.53	2	33	2000	mascul	12												
12	6.1-10	n„o	1.53	2	40	3490	feminir	48	22429.18	156.65	61.9	112	68.5	89	220	58	146	109		
5	1.1-3	n„o	1.58	4	39	3400	mascul	12	18370.99	172.8	74.1	125	73	88	218	55	140	101	0.6241	0.7995
11	1.1-3	1-14 tod	1.72	1	39	3200	masculin		15709.5	183.35	76.4	127.5	68.5	79	217	71	120	138	0.5995	0.5723
4	1.1-3	1-14 tod	1.56	1	37	3900	feminino		52976.75	168.5	102.9	128.5	86	63	189	53	123	49	0.5604	
17	6.1-10	n„o	1.64	3	42	2400	mascul	18	26437.07	180.7	87.9	136.5	83.5	102	215	44	105	398	0.5838	0.578
4	1 ou -	1-14 tod	1.61	1	43	3370	masculin													
5	1.1-3	n„o	1.56	6	41	4050	feminir	19												
5	1 ou -	n„o	1.54	1		1650	feminino													
6	1.1-3	n„o	1.62	1		3920	masculin													
2	1.1-3	n„o	1.55	5		3780	feminir	143	28858.27	164.6	67.9	109.5	73	69	172	60	101	60	0.5701	0.5762
16	10	n„o	1.62	1	39	2050	feminino		12640.72	161.6	53.7	106.5	69	99	168	68	84	80	0.5819	0.5561
5	3.1-6	n„o	1.52	1	39	3050	masculin		37071.88	169.85	95.3	153	106	97	195	34	127	161	0.581	0.5777
4	3.1-6	1-14 tod	1.62	4	39	2920	mascul	59												
0	3.1-6	n„o	1.62	2	36	3450	mascul	38	16110.76	182.4	77.4	117.5	77.5	81	285	49	220	66	0.5892	0.6489
3	1.1-3	n„o	1.53	2		2360	feminir	10	13617.51	158.1	50.2	98	58.5	88	199	44	106	215	0.5605	0.569
4	1.1-3	15 + tod	1.45	11	36	3650	mascul	58	22995.63	166.3	80.9	122.5	72	127	130	63	39	159	0.5916	0.5796
5	1.1-3	n„o	1.48	4		3440	feminir	65												
11	1.1-3	n„o	1.62	2	39	3650	feminir	61												
5	3.1-6	1-14 tod	1.53	6	39	3770	mascul	55												
3	1.1-3	1-14 tod	1.64	2	40	4990	mascul	28	24763.56	186.7	93.4	103.5	64	90	139	38	89	59	0.5797	0.5859
3	1.1-3	1-14 tod	1.53	3	40	2720	feminir	28												
2	3.1-6	n„o	1.64	10	39	3720	feminir	29	51414.22	173	109.8	119.5	73	99	160	58	85	159	0.6009	0.5903
12	3.1-6	n„o	1.52	1	41	3270	feminino		32771.83	171.55	75.5	113.5	72.5	78	218	95	106	106	0.5785	0.5792
5	1.1-3	n„o	1.6	1	41	3270	masculin													
2	1 ou -	n„o	1.55	2	37	3340	feminir	15												
0	1 ou -	n„o	1.5	4	37	2050	masculin													
9	3.1-6	15 + tod	1.47	4	38	2800	feminir	40	20918.85	154.8	62.6	106.5	70.5	78	189	65	113	51	0.5801	0.5752
0	1.1-3	15 + tod	1.55	5		3280	feminir	60												
9	3.1-6	n„o	1.5	7	37	3080	mascul	24												
5	1.1-3	n„o	1.65	1	42	3050	feminino			156									0.5764	0.5953
4	1.1-3	1-14 tod	1.54	1	37	3100	masculin		15519.12	171.55	71	122	63	60	178	61	100	115	0.5641	0.5623
0	1 ou -	n„o	1.6	3		2560	feminir	16	31785.1	167.4	82.4	106.5	63.5	88	188	37	131	116	0.5762	0.5795

pescmae	prenda	pfumomae	paltmae	pgesta	pidgest	ppn	psex	pint	dmgtotdxa	daltura2012	dpeso	dsysmed	ddiamed	dglicose	dcolesterol	dhdl	dldl	dtrig	dECMICE	dECMICD
4	1 ou -	15 + tod	1.5	3	39	3390	feminir	14	27947.76	156.35	65.5	114	72	69	176	71	97	49	0.5791	0.5833
6	3.1-6	n,,o	1.57	3	38	3300	mascul	94	12191.98	168.6	59.7	112	76.5	82	154	77	65	43	0.5788	0.5777
5	1 ou -	n,,o	1.58	2	43	2940	feminir	13	13083.16	156.1	46.8	93.5	58.5	81	161	64	90	46		
11	3.1-6	n,,o	1.62	2	42	2520	feminir	18												
5	1.1-3	n,,o	1.54	4	40	3800	mascul	39												
5	10	n,,o	1.49	7		3100	mascul	13												
2	1.1-3	1-14 tod	1.62	4	36	2630	mascul	16	38710.55	172.65	110.5	159.5	99.5	82	187	50	120	89	0.5949	0.5743
5	3.1-6	15 + tod	1.57	2	39	3180	feminir	49	42047.27	161.9	88.9	131.5	92.5	104	218	74	104	240	0.5865	0.5745
0	1 ou -	n,,o	1.51	1		3620	feminino		15463.73	162.8	55.7	101	66	87	178	65	107	47	0.5752	0.5694
15	10	n,,o	1.7	3	41	3700	feminir	38	26593.71	171.85	68.3	106.5	68	84	200	71	113	108	0.5777	0.5728
4	1.1-3	n,,o	1.57	2		3970	mascul	57												
7	1.1-3	1-14 tod	1.57	2		3200	mascul	16	27184.68	166.45	84.1	129	83	102	249	46	173	149	0.5833	0.5748
16	10	1-14 tod	1.55	2	39	3320	masculin													
3	1.1-3	1-14 tod	1.57	1		3250	feminino		27456.48	161.15	67	115.5	74	88	229	94	106	161		
5	1.1-3	n,,o	1.72	1	42	3450	feminino													
5	1.1-3	n,,o	1.51	3	42	3650	mascul	51												
1	1 ou -	1-14 tod	1.51	4	39	3300	mascul	120	7402.993	169.9	66.1	107	54	98	153	60	79	40	0.5582	0.5683
6	1 ou -	n,,o	1.6	1	38	3200	feminino		30627.91	160.15	68	114.5	77.5	109	200	76	108	100	0.5843	0.5741
4	1.1-3	15 + tod	1.54	1	42	3470	masculin													
4	1.1-3	n,,o	1.5	2	36	2800	mascul	47	16905.79	172.2	78.5	136	72.5	132	192	58	100	222	0.5772	0.5851
9	10	n,,o	1.55	4	38	3000	mascul	38												
2	1 ou -	n,,o	1.49	2		2980	feminir	21												
0	1 ou -	n,,o	1.53	2		3470	mascul	18												
14	1.1-3	n,,o		1	38	3250	feminino		21273.99	158.7	59.6	105.5	71	70	180	64	104	92	0.5739	0.588
8	6.1-10	n,,o		2	40	2900	mascul	73	20252.1	173.15	83.7	114.5	70.5	106	180	50	112	89	0.5794	0.578
17	10	n,,o	1.53	2	41	4080	mascul	23												
14	10	1-14 tod	1.59	2	39	3080	feminir	41												
2	3.1-6	n,,o	1.6	1	36	3730	masculin		19357.85	180.8	76.9	125.5	83	86	152	58	79	81	0.5755	0.5618
5	1 ou -	n,,o	1.58	1	39	3200	masculin		15791.43	168.9	71.6	110	59.5	97	164	44	79	205	0.5697	0.578
4	1.1-3	15 + tod	1.49	2	40	3210	feminir	34	42868.2	158.6	86.1	107	69	105	154	40	101	82	0.5853	0.5798
12	3.1-6	n,,o	1.64	2	37	2880	mascul	91	33425.77	177	97.5	150.5	99.5	515	686	97	75	3111		
12	3.1-6	n,,o	1.55	2	41	3040	feminir	84	19965.34	152.9	52.6	107	64.5	83	211	103	97	40	0.5778	
5	1 ou -	1-14 tod	1.68	2		2030	feminir	17	21714.51	163.35	62.8	110	68.5	93	179	38	97	253	0.5779	0.5734
1	1 ou -	1-14 tod	1.54	2	39	3050	mascul	23	15225.4	176.9	78.9	127.5	81	96	257	41	150	256	0.5824	0.597
9	1.1-3	1-14 tod	1.53	1		2760	feminino		36802.54	154	76.9	118.5	83.5	86	190	60	117	73	0.5666	
8	3.1-6	n,,o	1.63	4	38	3700	mascul	27	30311.28	179.75	107.1	134	88	91	248	44	152	336	0.6335	0.6173
12	6.1-10	n,,o	1.53	2	39	3750	mascul	151												
1	1.1-3	n,,o	1.51	1	38	3100	feminino			157.8	73.1	112.5	78	98	261	45	147	356	0.5795	0.602
9	1.1-3	n,,o	1.66	3	39	4050	feminir	115												
1	1.1-3	15 + tod	1.54	1		2530	feminino		25938.69	152	61.5	103	71	87	121	40	69	47	0.5727	0.5608
12	6.1-10	n,,o	1.65	1	42	3250	feminino													
15	6.1-10	n,,o	1.61	3		4170	mascul	31												
4	1.1-3	15 + tod	1.54	2	43	2580	feminir	12	28283.3	153.4	66.8	103.5	65.5	90	188	57	112	97	0.5723	
11	10	n,,o	1.62	2	40	3570	feminir	63												
6	1 ou -	n,,o	1.43	1	29	1050	masculin													
5	1 ou -	1-14 par	1.54	1		2600	feminino													
7	1 ou -	15 + tod	1.67	5	40	3380	mascul	21	5659.225	179.75	75.3	124	66.5	83	192	58	110	114	0.5791	0.5723
3	6.1-10	1-14 par	1.6	2	40	3200	mascul	12												

pescmae	prenda	pfumomae	paltmae	pgesta	pidgest	ppn	psex	pint	dmgtotdxa	daltura2012	dpeso	dsysmed	ddiamed	dglicose	dcolesterol	dhdl	dldl	dtrig	dECMICE	dECMICD
6	1.1-3	1-14 tod	1.61	1	34	2170	feminino		37164.25	158.25	81.5	108.5	69	97	193	43	124	125	0.5761	0.5778
5	1.1-3	n„o	1.56	3	39	3710	mascul	16	4053.314	173.4	69.9	135.5	72	75	203	73	111	117		
4	1.1-3	1-14 par	1.53	2	40	3720	feminir	22	34673.8	152.4	72.4	113	69	81	239	64	150	117	0.5684	0.5732
14	3.1-6	n„o	1.55	3	39	2700	feminir	107												
7	3.1-6	1-14 tod	1.44	3		2250	feminir	24												
5	1.1-3	n„o	1.52	6	39	3350	mascul	82	24054.5	163.45	74.7	123.5	76.5	110	162	43	99	106	0.5635	0.5788
0	1 ou -	1-14 tod	1.57	3		2230	feminir	180	44932.07	158.7	93	127	82	136	179	62	98	122		
2	1 ou -	n„o	1.58	2		1500	mascul	24	27295.61	179.15	92	129	74.5	89	158	60	88	67	0.578	0.5785
9	6.1-10	n„o	1.56	3	38	3250	feminir	150												
12	1.1-3	n„o	1.49	2	39	3700	mascul	12												
11	3.1-6	n„o	1.76	1	41	4560	masculin		35487.26	191.8	108.5	120.5	80.5	85	226	46	142	218	0.6466	0.6209
20	10	n„o	1.67	2	39	4170	feminir	23	16511.16	172.6	66.1	129	83	79	200	88	95	80	0.578	0.5772
1	1.1-3	n„o	1.58	3	40	3050	mascul	158	44219.48	168.5	103	113	75	89	198	47	118	178	0.5648	0.5802
4	3.1-6	n„o	1.53	2	38	4440	mascul	17												
5	1.1-3	1-14 tod	1.55	4	42	3350	mascul	17												
5	1 ou -	n„o	1.56	6	41	4500	feminir	44	27758.29	159.8	64.4	106	70	155	198	71	102	115	0.5782	0.5444
5	3.1-6	n„o	1.62	4	41	3950	mascul	109												
8	1.1-3	n„o	1.65	8	39	3400	mascul	50												
4	1 ou -	n„o	1.57	1	42	3250	masculin		17557.03	181.85	80.2	132	80.5	78	129	45	75	47	0.5807	0.5767
12	3.1-6	n„o	1.58	3	38	3450	mascul	25	19794.14	185.5	91.1	129	71.5	102	226	42	141	209	0.6352	0.6044
3	1.1-3	n„o	1.64	2	40	3710	mascul	51	28313.64	181.3	105.4	134	84	117	147	46	80	123	0.5779	0.5787
4	1 ou -	n„o	1.57	3	42	3150	feminir	60												
4	1 ou -	15 + tod	1.58	4		2850	feminir	64												
5	1.1-3	n„o	1.56	2	41	3320	feminir	29	25226.33	156.75	60.9	127	85.5	105	167	57	101	41	0.5835	0.5807
4	1 ou -	1-14 tod	1.58	1	34	2310	feminino		16201.3	154.5	54.5	121	71	63	181	78	85	86	0.5778	0.5708
10	1.1-3	n„o	1.56	1	38	3700	feminino		39336.32	171.5	84.6	111.5	65	80	176	73	80	140	0.5846	
7	3.1-6	1-14 tod	1.54	1	37	2620	masculin		7805.716	176.1	70.7	123.5	74.5	75	255	72	163	126	0.5783	0.5758
5	1.1-3	n„o	1.63	2		3550	feminir	36	32081.37	176	77.1	121.5	80	91	158	57	95	40	0.578	0.5803
6	3.1-6	n„o	1.61	1	40	2800	masculin			176	142	123.5	84.5	82	260	84	153	111	0.6202	0.6232
0	1 ou -	n„o	1.59	9		4540	mascul	35				153	96							
8	1.1-3	n„o	1.58	1	39	2600	feminino		30613.6	148.35	63.3	118	81	81	177	66	99	65		
6	1.1-3	15 + tod	1.63	1	41	3750	masculin													
8	3.1-6	1-14 tod	1.65	1	39	2700	masculin		24992.39	181.4	85.4	113.5	65.5	77	180	48	119	77	0.5789	0.5789
2	1 ou -	n„o	1.5	1	41	3200	feminino													
5	1 ou -	n„o	1.61	2		3350	feminir	44												
4	1 ou -	15 + par	1.57	4		1750	mascul	13												
8	1.1-3	n„o	1.61	2		2600	mascul	29												
5	1 ou -	n„o	1.54	2	36	2700	feminir	38												
7	10	n„o	1.54	10		4250	mascul	203	13142.26	172.4	85.5	145	88	76	139	33	92	54	0.5792	0.576
4	1.1-3	n„o	1.61	3		1920	feminir	12	21558.88	161.65	60.8	120.5	74	81	230	50	160	99	0.5767	0.5698
8	1.1-3	n„o	1.5	5	40	3080	mascul	11												
11	1.1-3	n„o	1.55	4	38	2950	mascul	127	25766.21	169	88.2	131	76	79	237	58	138	211	0.5849	0.5788
5	1.1-3	n„o	1.53	1	41	2900	masculin		7047.652	172.25	58	139.5	85.5	86	197	56	126	62	0.579	0.5781
5	1.1-3	1-14 tod	1.57	10		2590	feminir	112	16124.66	163.35	58.2	109.5	70	80	179	62	110	37		
7	6.1-10	1-14 tod	1.57	3	38	3480	mascul	20	14202.92	169	74.6	108	62	110	172	71	95	54	0.5782	0.5817
5	1 ou -	1-14 par	1.69	2		3430	feminir	24												
8	1.1-3	n„o	1.5	2		3700	feminir	93	13995.49	160.6	58.9	127.5	90	63	169	78	76	83	0.6058	0.5785
5	1 ou -	1-14 par	1.58	1		3350	masculin		23405.6	171.45	79.1	125	84.5	92	185	50	118	91	0.5756	0.5907

pescmae	prenda	pfumomae	paltmae	pgesta	pidgest	ppn	psex	pint	dmgtotdxa	daltura2012	dpeso	dsysmed	ddiamed	dglicose	dcolesterol	dhdl	dldl	dtrig	dECMICE	dECMICD
9	10	n„o	1.64	2	40	3750	mascul	30												
8	10	n„o	1.61	2		1730	feminino		18739.97	158.25	59.9	119.5	77.5	95	190	69	100	105		0.5826
10	3.1-6	n„o	1.58	1	40	3850	masculin		24238.53	169.35	73.5	117.5	71	69	239	89	109	163	0.5667	0.5675
0	1 ou -	1-14 tod	1.54	2	38	2800	mascul	69	13404.08	178.9	85.2	141.5	91.5	95	140	61	67	45	0.5843	0.5779
11	10	n„o	1.55	3	40	3090	mascul	43	19310.22	179	86.2	144	65	88	188	58	120	69	0.5832	0.6422
16	3.1-6	n„o	1.56	1	39	3240	masculin													
7	3.1-6	n„o	1.54	2	40	3340	feminino		26927.97	162.2	63.6	98	62.5	80	213	67	134	89	0.5782	0.5797
5	1.1-3	15 + tod	1.49	2	38	2900	mascul	82	21527.91	170.85	72	120	78.5	89	200	41	133	188		
4	1.1-3	1-14 tod	1.55	1		2750	feminino		24307.01	158.2	61.2	120.5	81	90	175	67	88	149		
2	1 ou -	1-14 tod	1.58	5		3200	mascul	13	19436.08	171.3	79.3	149.5	91	95	386	95	263	85	0.6578	0.6816
9	1.1-3	1-14 tod	1.59	5		3900	mascul	58												
1	1 ou -	1-14 par	1.61	3	41	2670	feminir	25												
9	3.1-6	n„o	1.56	1	39	3450	masculin													
10	3.1-6	n„o	1.61	1	39	3140	masculin		19795.76	175.45	78.4	146	66						0.5894	0.5742
10	3.1-6	n„o	1.6	1		3050	masculin													
5	3.1-6	n„o	1.6	1	42	3300	masculin		21913.9	171.9	77	142.5	78.5	81	225	52	144	192	0.5766	0.5807
4	1.1-3	1-14 par	1.66	1	39	4080	masculin		34375.02	178.25	95.6	132.5	74	88	232	47	157	86		
5	3.1-6	n„o	1.68	5	38	3950	mascul	141	37245.82	188.1	114.5	149	84	128	230	37	105	383	0.5802	
8	1.1-3	1-14 par	1.63	2	28	970	feminir	13												
0	1 ou -	n„o	1.55	2	42	3960	feminir	43	38689.91	171.7	81.6	110	71.5	71	243	73	144	182		0.5934
11	1.1-3	n„o	1.71	2		2200	feminir	15												
9	3.1-6	n„o	1.49	2	40	3170	feminir	96												
2	1 ou -	1-14 tod	1.61	3		2850	mascul	32												
0	1.1-3	n„o	1.52	7	40	3350	mascul	123	23873.79	166.95	74.1	112.5	81.5	151	286	67	200	142	0.5975	0.5783
5	1.1-3	n„o	1.54	4	40	3000	feminir	12	43087.72	151	82	127.5	94.5	83	178	72	94	65	0.5825	
16	3.1-6	n„o	1.56	2	39	3250	feminir	59												
16	6.1-10	15 + tod	1.54	1	35	2400	masculin													
4	1.1-3	n„o	1.43	2	38	2680	mascul	18	13832.64	169.7	67.5	117.5	75.5	83	192	50	125	70	0.5812	0.6197
5	1.1-3	n„o	1.61	1	36	2150	masculin		11026.37	173.5	77.3	131.5	83.5	82	198	53	123	111	0.5804	0.5754
16	10	n„o	1.63	1	41	3600	feminino		25657.88	171.3	69.2	116	76	98	189	82	88	117	0.5718	0.5697
5	1.1-3	15 + tod	1.55	6	40	2530	mascul	26												
3	1 ou -	n„o	1.5	4	39	3500	mascul	11												
0	1 ou -	15 + tod	1.37	10	42	2820	mascul	65												
7	1.1-3	1-14 tod	1.61	1	37	2870	feminino		39834.86	166.05	79.7	115.5	75.5	103	180	53	90	263	0.57	0.58
5	1.1-3	n„o	1.47	3	42	2950	feminir	38	32151.54	157.8	69.6	111.5	76	89	151	59	67	128	0.5766	0.581
5	1.1-3	1-14 tod	1.59	3	40	3000	mascul	50	29983.3	175.2	85.3	115.5	77	88	162	48	98	61	0.5654	0.5734
12	6.1-10	15 + par	1.47	1	39	3080	masculin		25284.85	172.1	85.1	152.5	96.5	86	243	63	152	111	0.5798	
11	1.1-3	n„o	1.47	2	39	3200	mascul	34	12669.66	167.75	60.4	106	62	99	199	65	118	69	0.576	0.5771
8	1.1-3	1-14 par		2	41	3240	feminir	25												
7	1.1-3	1-14 par	1.5	2	38	2400	feminir	21						99	132	60	66	48		
7	1.1-3	n„o	1.57	2	41	2810	feminir	39												
3	1.1-3	n„o	1.54	2	42	3720	mascul	19	31002.71	169.5	95.1	141	79	95	226	40	151	160	0.5789	
5	1.1-3	n„o	1.57	2		3750	mascul	37	36415.27	180.3	101.1	141.5	106	102	250	55	140	290	0.5958	0.5941
1	1 ou -	n„o	1.66	7	42	2730	feminir	99												
17	6.1-10	n„o	1.62	1	40	3580	masculin		32206.62	182.2	93.9	118.5	80	80	235	65	150	91	0.558	0.5805
8	1.1-3	n„o	1.64	4		3950	feminir	20	22124.86	156.3	57.4	113	73.5	77	164	78	69	104	0.5784	0.581
6	3.1-6	15 + tod	1.54	2	39	2270	feminir	24												
5	1 ou -	n„o		1	38	3100	feminino		22733.19	166.5	62.3	125	71.5	77	215	72	119	151		0.5811

pescmae	prenda	pfumomae	paltmae	pgesta	pidgest	ppn	psex	pint	dmgtotdxa	daltura2012	dpeso	dsysmed	ddiamed	dglicose	dcolesterol	dhdl	dldl	dtrig	dECMICE	dECMICD
4	1.1-3	n„o	1.63	2	40	4400	feminino													
9	3.1-6	1-14 par	1.66	1	38	2920	feminino		46107.74	165.3	102.7	115.5	68	87	202	74	112	78	0.5863	0.5881
10	3.1-6	n„o	1.62	2	38	4320	feminir	63	29969.57	163.7	72.2	109	70	90	184	58	107	106	0.5783	0.5786
8	1 ou -	n„o	1.5	1	39	2950	masculin		20447.19	164.6	73.1	121	73	111	198	31	104	355	0.6284	0.5792
4	1.1-3	n„o	1.54	5	36	2500	mascul	20	10847.23	174	67.2	140	71	84	140	57	69	58	0.5781	0.5782
6	1.1-3	n„o	1.58	2	40	4270	feminir	36	23158.06	162.4	62.4	115	76.5	74	240	92	130	88	0.5769	0.5771
3	1.1-3	1-14 tod	1.6	3	43	3200	feminir	41	46776.84	157.4	105.9	123	72	114	187	62	103	144	0.5806	0.5883
11	1.1-3	1-14 tod	1.64	1	43	3260	feminino		37148.13	167.2	87.6	119	84	82	161	48	98	75	0.5663	0.575
5	3.1-6	n„o	1.64	2	39	3150	feminir	69												
10	1.1-3	1-14 tod	1.55	2	36	2850	feminir	24	19366.44	160.45	59.8	109.5	68	87	229	68	133	143	0.5729	0.578
5	1 ou -	15 + tod	1.49	2	40	3070	feminir	49	16605.54	155.35	55.4	119	73	86	208	62	130	71	0.6316	0.6169
6	1.1-3	n„o	1.6	1	38	2850	feminino		14949.37	157.1	48	109.5	72.5	86	157	60	83	56	0.5838	0.5773
5	1 ou -	1-14 tod	1.47	1		3050	feminino		41066.1	147.8	74.8	101	70.5	83	158	57	92	48	0.5783	0.6203
6	1.1-3	n„o	1.49	3	38	2820	feminir	14												
9	1.1-3	1-14 tod	1.51	1	40	2890	masculin		10355.43	166.5	64.1	124	72.5	74	199	66	114	100	0.5782	
17	6.1-10	n„o	1.64	1	40	2250	masculin													
6	1.1-3	15 + tod	1.47	1		2810	masculin													
4	1 ou -	n„o	1.52	1	39	3000	feminino		43825.8	153.7	82.2	108.5	78	107	188	44	132	98		
3	1 ou -	n„o	1.5	2	39	3780	feminir	92												
5	1.1-3	1-14 tod	1.53	9	42	3760	feminir	102	45612.5	162.3	94.6	124	80.5	92	195	46	87	357	0.5796	0.5811
4	6.1-10	n„o	1.57	1	37	3170	masculin													
5	1.1-3	n„o	1.56	2	39	2210	feminir	51	10914.2	152.1	40.6	96	64	75	147	68	60	122	0.5755	0.5389
4	1 ou -	n„o	1.49	1	39	2840	masculin													
9	3.1-6	n„o	1.52	6	41	3350	feminir	34	39559.42	161.75	83.6	122.5	76	73	163	49	101	76	0.5801	0.577
2	1 ou -	1-14 par		3		3450	feminir	114	20913.93	161.7	59.2	117.5	80	75	169	36	103	168	0.5373	0.5672
6	1.1-3	n„o	1.53	3	39	3910	feminir	34	11899.44	160.45	49.1	111	72.5	67	184	78	87	93	0.5783	0.58
5	1 ou -	15 + tod	1.54	1	41	2910	feminino		36016.15	159.3	73.5	103.5	73	85	186	57	102	149	0.607	0.6453
4	6.1-10	n„o	1.61	2	39	4180	feminir	33	35116.47	162.7	83.5	114.5	73.5	73	160	44	100	105	0.5633	0.5776
0	1 ou -	1-14 tod	1.63	2		2380	mascul	27	25284.75	167.95	84.2	142.5	86.5	108	250	44	149	311	0.5798	0.5878
4	1 ou -	n„o	1.61	5	38	3470	mascul	16	3530.797	177.9	64.9	145.5	80.5	76	189	87	88	65	0.6382	0.6358
2	1.1-3	n„o	1.64	2	38	2850	feminir	15												
6	1 ou -	n„o	1.52	6		3340	feminir	34	35898.63	160.7	79.5	131	85	75	195	75	105	80	0.5853	0.5772
5	1.1-3	n„o	1.46	2		3420	mascul	13	14971.86	166.15	68	124.5	81	95	143	50	76	83	0.8009	0.5782
5	1.1-3	n„o	1.56	5	42	3470	feminino													
10	6.1-10	n„o	1.67	3	38	3500	mascul	37	11867.29	179.3	70.3	134	69.5						0.5774	0.5797
4	1 ou -	n„o	1.58	1		3170	feminino													
4	1.1-3	1-14 tod	1.51	1	41	2800	masculin		36521.74	175.55	116.3	138.5	82	103	159	43	79	236	0.6929	0.6092
15	10	n„o	1.62	1	39	3520	masculin													
13	1.1-3	1-14 tod	1.67	2	40	3960	feminir	42	17192.21	159.85	51.7	117	75	78	213	66	125	131	0.5789	0.5815
8	1.1-3	n„o	1.5	4	40	3700	mascul	17												
12	1.1-3	1-14 par	1.64	2	42	3500	feminir	66	26480.38	162.9	66.5	105	67.5	86	170	59	89	110	0.5783	0.5824
3	1.1-3	n„o	1.56	2	38	3170	feminir	40	14547.24	159.9	49	110	64.5	83	183	65	100	80	0.5963	0.5783
2	1.1-3	n„o	1.6	3	37	3730	feminir	33	56345.15	163.15	107.8	135	85	84	204	78	105	101	0.5849	0.5859
5	1.1-3	1-14 tod	1.64	1	38	3160	feminino													
5	3.1-6	n„o	1.55	2	41	3270	mascul	13	26252.41	171.55	89.4	135.5	73.5	88	197	46	133	124	0.5755	0.5713
5	1.1-3	n„o	1.6	3	41	3350	feminir	74	26786.05	167.15	63.6	107	71	68	175	58	107	68		
5	1.1-3	n„o	1.58	1	43	4430	feminino		17953	159.65	56	113	65.5	87	189	71	106	63	0.5907	0.5786
7	1.1-3	1-14 tod	1.61	1	40	2930	feminino													

pescmae	prenda	pfumomae	paltmae	pgesta	pidgest	ppn	psex	pint	dmgtotdxa	daltura2012	dpeso	dsysmed	ddiamed	dglicose	dcolesterol	dhdl	dldl	dtrig	dECMICE	dECMICD
6	1 ou -	1-14 tod	1.59	1	39	3590	masculin		19216.6	180.7	72.3	127	76.5	104	161	40	99	171	0.5802	0.593
9	6.1-10	n„o	1.55	3	41	3200	feminir	80	18243.99	158.75	54.7	131.5	78.5	65	146	61	74	40	0.5773	0.5784
5	1.1-3	n„o		2		3630	mascul	14	12668.38	173.95	62.5	141	84	139	200	100	83	84	0.5608	0.5735
1	1 ou -	n„o	1.47	1		2740	masculin													
12	6.1-10	n„o	1.68	2	42	3200	mascul	31	22564.82	186.8	78.6	121.5	82	77	201	32	142	157	0.579	0.579
0	1 ou -	1-14 tod	1.56	4	40	2930	mascul	31	5961.054	165.95	58.6	121.5	73.5	80	158	60	90	48		0.5632
5	1.1-3	n„o	1.57	1	38	3120	feminino		60765.17	165.5	116.8	126	82	88	196	64	121	90		0.5739
9	1 ou -	n„o	1.57	4	40	3300	mascul	74	24708.7	182.7	84.4	118	67	126	207	46	100	297	0.5792	0.5808
3	1.1-3	1-14 tod	1.55	3		3900	mascul	16												
8	1.1-3	n„o	1.48	1	41	3450	masculin		20635.25	167	71	125.5	85	82	220	59	140	117	0.584	0.5739
11	1.1-3	n„o	1.6	2	39	3720	mascul	35	7507.23	173.8	65.4	129.5	79.5	69	145	57	61	139	0.5689	0.5775
9	6.1-10	n„o		3	38	3400	mascul	65	14621.49	176.1	68.4	132.5	84.5	60	150	75	62	81		
6	1 ou -	15 + tod	1.41	10	41	2030	mascul	24	3992.597	162.4	49.3	112.5	68	98	135	58	70	39	0.5784	0.576
2	1.1-3	n„o	1.65	3	40	3500	mascul	60												
5	1.1-3	1-14 par	1.53	1	40	3250	feminino		27994.96	158.8	74.5	131	86.5	78	163	50	94	102	0.6195	0.6575
4	1.1-3	n„o	1.51	7		3160	feminir	45	28102.55	154.9	62.8	120.5	71.5	94	214	63	138	91	0.5803	0.5824
8	1.1-3	n„o	1.54	1		3580	masculin		14173.07	173.55	63.8	124	74	112	192	63	118	73	0.5758	0.571
15	10	n„o	1.54	1		2770	feminino													
8	1.1-3	n„o	1.54	4	39	4100	masculin													
1	1 ou -	n„o	1.56	2	38	3300	mascul	126												
3	1 ou -	n„o	1.54	1	40	3400	masculin		9120.391	179.85	63.9	134	75	82	227	45	161	79	0.5781	0.5873
4	1.1-3	1-14 tod	1.7	4	39	2750	feminir	32	30243.99	161	74.2	124	82.5	65	140	33	97	40	0.5793	0.5663
2	1 ou -	n„o	1.54	2	41	3560	mascul	24	29612.92	170.55	93.9	132	81	94	180	48	116	92	0.5874	0.5956
1	1 ou -	15 + tod	1.55	3	41	3170	feminir	36	23918.22	167.8	67.3	128.5	77	105	191	63	88	234	0.5784	0.5856
5	1.1-3	n„o	1.61	1	40	3360	masculin													
4	1.1-3	15 + tod	1.58	1	37	2650	feminino		18483.48	159.9	58	129	82	88	178	73	92	50	0.5606	0.5869
2	1 ou -	15 + tod	1.6	3	39	3530	mascul	53	23480.22	180.3	92.1	125	74	93	173	47	117	52	0.5636	0.5766
3	1.1-3	n„o	1.58	4	42	2900	feminir	21	25792.78	152.6	62	118.5	78.5	85	193	43	134	96		0.5732
8	1.1-3	n„o	1.56	5	40	3300	feminir	48	29019.8	160.15	63.6	141.5	94						0.5615	0.5746
1	1.1-3	1-14 tod	1.58	5		3900	mascul	12												
3	1.1-3	15 + tod	1.59	13		3600	feminir	28												
1	1 ou -	n„o	1.52	2	41	3050	mascul	38	9742.716	178.25	66.8	119	67.5	128	162	79	71	34		0.57
3	1.1-3	n„o	1.52	3	42	3430	feminir	10	22349.98	143	58.5	105	59.5	74	169	46	88	170		
7	3.1-6	1-14 tod	1.63	3	39	2700	feminir	32												
5	1.1-3	n„o	1.56	2	40	4000	mascul	101												
2	1.1-3	n„o	1.54	1	39	2750	feminino		21921.53	163.2	55.7	134.5	91	73	219	95	108	63	0.5716	0.572
4	1.1-3	15 + tod	1.58	2		3650	mascul	45												
4	1.1-3	n„o	1.57	1	39	3680	masculin		20645.8	182.15	75	122	77	84	190	65	113	40		
8	1.1-3	1-14 tod	1.57	1	38	3050	masculin		23060.38	175.7	70.2	127	88	90	227	63	136	250	0.5968	0.5979
8	1.1-3	n„o	1.65	5		3800	feminir	52	11403	157.9	47.6	114	73.5	100	165	64	87	66	0.5756	0.5807
1	1.1-3	n„o	1.69	2		3900	mascul	40	16156.01	180.7	81.7	114.5	65.5	103	168	40	107	101	0.6118	0.5816
3	3.1-6	n„o	1.49	1		3050	feminino													
7	1.1-3	n„o	1.6	5	39	2950	mascul	27												
16	10	n„o	1.59	1		2450	masculin		29810.94	166.85	78.5	124.5	75.5	80	222	69	129	122		
3	1.1-3	n„o	1.58	1	41	3300	masculin		9595.336	168.05	76.4	139.5	72.5	94	217	61	113	203	0.5783	0.5772
5	1.1-3	n„o	1.47	1	41	3350	feminino													
0	3.1-6	n„o	1.59	3	42	3690	feminir	93	54386	167.5	97.9	114	81	85	153	76	60	76	0.5782	0.58
9	1.1-3	n„o	1.51	1		3150	masculin													

pescmae	prenda	pfumomae	paltmae	pgesta	pidgest	ppn	psex	pint	dmgtotdxa	daltura2012	dpeso	dsysmed	ddiamed	dglicose	dcolesterol	dhdl	dldl	dtrig	dECMICE	dECMICD
5	1.1-3	n„o	1.59	7		3400	feminir	50	38290.27	160.45	80.7	122.5	76	87	270	67	180	124		
16	3.1-6	n„o	1.63	1	39	3500	masculin			182.25	130.2	123	86.5	75	187	52	110	107		
3	1.1-3	n„o	1.63	4		3850	mascul	20												
7	1 ou -	n„o	1.48	1	40	3300	masculin													
12	3.1-6	n„o	1.46	5	39	3250	feminir	45	39101.93	161.35	77.7	123.5	86	83	217	87	119	69	0.5784	0.578
9	10	n„o	1.64	7	42	3200	mascul	17												
4	1.1-3	15 + tod	1.53	9		3000	feminir	12	14371.16	157.5	53	117.5	78.5	69	137	59	67	39	0.5449	0.5641
0	1 ou -	n„o	1.53	2	38	3710	mascul	35												
4	1.1-3	15 + tod	1.67	1	40	3560	feminino		32120.5	166.2	77	120.5	83.5	76	149	63	65	76	0.5855	0.5968
2	1.1-3	n„o	1.55	4	40	3350	mascul	83	2608.573	169.85	60.6	111	66.5	79	115	40	67	37	0.578	0.573
0	1.1-3	1-14 tod	1.6	2		1800	feminino													
5	1 ou -	n„o	1.44	5	39	3030	feminir	143	32990.07	150	67.5	117.5	75.5	112	209	59	136	127	0.6115	0.583
6	1.1-3	1-14 tod	1.52	3		2460	mascul	9	25693.67	173.4	90.1	145.5	92.5	87	177	64	103	67	0.7914	0.8518
5	1.1-3	1-14 par	1.54	1	40	2860	feminino		24690.93	155.85	64.5	122	85	80	209	68	128	102	0.5792	0.5784
7	1.1-3	n„o	1.62	1		3100	feminino			165.6	125.7	115	78.5	111	259	68	158	174		
16	3.1-6	n„o	1.61	4	37	2880	mascul	42	30414.2	181.9	95.3	132.5	74.5	90	183	47	118	99	0.579	0.5807
3	1 ou -	n„o	1.57	10	38	2320	mascul	30	19407.34	169.9	77.2	125	73.5	106	203	34	118	260	0.5881	0.5779
5	1.1-3	1-14 tod	1.6	3	40	3470	mascul	27	16927.92	167.9	79.2	142	78.5	113	258	46	152	299		
12	1 ou -	n„o	1.64	2	42	4050	feminino		26288.99	169	69.4	120.5	77	93	160	68	76	79	0.5343	0.5665
5	3.1-6	1-14 tod	1.56	1	41	2650	feminino													
3	1.1-3	1-14 tod	1.58	1	38	3000	masculin		30962.66	177.95	94.1	155.5	94	62	170	60	93	68		
9	3.1-6	1-14 par	1.58	3	41	3460	feminir	41	32392.49	154.25	67	111.5	73.5	84	220	78	123	136	0.5799	0.5803
5	1 ou -	n„o	1.56	3	39	3550	feminir	20												
3	3.1-6	n„o	1.63	2	42	3360	feminir	56	42364.25	167.85	90.8	114.5	80.5	72	180	75	79	141		
8	1.1-3	n„o	1.6	1	38	3380	feminino		23253.8	160.6	60.1	119.5	71.5	82	195	38	130	128		0.5778
0	3.1-6	1-14 tod	1.55	5	39	3320	feminir	48	19063.13	167.55	58	116	65	79	176	62	88	111	0.5784	0.5703
2	1.1-3	1-14 tod	1.6	1	43	3070	masculin													
9	1 ou -	15 + tod	1.6	3	39	3870	mascul	45												
12	10	n„o	1.51	3	39	4490	mascul	14												
15	6.1-10	n„o	1.53	1	40	3320	masculin													
6	1.1-3	n„o	1.57	1	42	3150	feminino		26444.31	156.1	62.6	99	57.5	91	171	67	88	81	0.5754	0.5805
2	1 ou -	n„o	1.53	7	36	2760	feminir	24												
5	1.1-3	1-14 tod	1.58	1		3300	feminino		45626.11	161.8	92.1	123	79.5	91	140	68	57	91	0.5714	0.572
9	1.1-3	1-14 par	1.65	1	39	3810	feminino			168	121.4	126.5	83.5	88	216	61	149	46	0.6255	0.582
5	1.1-3	n„o	1.56	3	41	4250	feminir	76	38256.67	170.7	80.5	106.5	64	72	185	64	101	127		
11	1.1-3	1-14 tod	1.55	3	38	2150	feminir	64	18539.91	154.8	57.6	113.5	67	79	230	61	140	233		
8	3.1-6	1-14 par	1.6	1	42	3530	masculin													
3	3.1-6	n„o	1.65	1	38	2970	masculin			174.6	122.3	157	104	88	190	65	97	156		
7	1.1-3	n„o	1.54	1	39	2950	masculin		16485.6	163.95	67.8	118	72	81	185	53	120	69	0.5775	
3	1.1-3	n„o	1.51	1	39	3170	masculin		32291.31	178.1	115.5	141.5	78.5	133	195	40	98	349	0.616	0.5985
11	1.1-3	1-14 par	1.55	1		2740	feminino													
5	1 ou -	n„o	1.52	2	39	2750	feminir	25	18000.04	173.5	66.2	124.5	75.5	83	180	67	96	86	0.5867	0.5946
1	1.1-3	n„o	1.62	4	38	2820	mascul	38	46953.71	180.15	111.5	132	83.5	94	185	85	87	94	0.5833	0.5794
9	1.1-3	1-14 par	1.58	2	42	2850	feminir	28	38284.75	161.85	82.2	139.5	94.5	72	230	75	137	123	0.5831	0.5807
10	3.1-6	1-14 tod	1.6	2	38	3170	feminir	54												
13	1.1-3	n„o	1.59	1	40	3630	masculin		22190.05	172.65	73.6	114	72						0.576	0.5731
4	1.1-3	n„o	1.67	2		3000	feminir	15	27169.7	160.15	65.1	111	67.5	88	160	82	67	68	0.5789	0.5793
14	3.1-6	n„o	1.52	1	40	3100	feminino		20382.69	160.5	59.8	88	55.5	78	130	41	78	48	0.5791	0.5778

pescmae	prenda	pfumomae	paltmae	pgesta	pidgest	ppn	psex	pint	dmgtotdxa	daltura2012	dpeso	dsysmed	ddiamed	dglicose	dcolesterol	dhdl	dldl	dtrig	dECMICE	dECMICD
3	1.1-3	n„o	1.5	2	40	3720	feminino													
5	1.1-3	n„o	1.55	1	42	3930	feminino		19371.6	161.6	53.5	157	91	97	239	87	132	117	0.5815	0.5662
7	1.1-3	n„o	1.51	2	41	3600	mascul	33	28571.28	179	91.7	136.5	78.5	104	231	52	119	371	0.5789	0.6263
14	1.1-3	1-14 tod	1.61	1	38	3250	masculin													
5	1.1-3	1-14 tod	1.68	2	38	2850	feminir	55												
1	1 ou -	n„o	1.5	2	40	2850	feminir	149	14358.38	155.35	47.4	93	58.5	77	136	58	63	62	0.5725	0.5586
8	1.1-3	1-14 tod	1.65	5		3740	mascul	13	21996.91	180.1	89.9	122	75	85	192	57	122	77	0.5961	0.5891
12	1 ou -	n„o	1.64	4	36	2240	feminir	27												
18	10	n„o	1.64	1	40	3350	masculin		30965.76	187.8	99.9	126	69.5	77	247	67	164	99		
14	6.1-10	n„o	1.61	1	36	2780	feminino		15915.92	167.75	56.5	103.5	71	78	146	67	69	44	0.5777	0.577
5	1 ou -	1-14 tod	1.6	4	38	4460	feminir	51												
9	1.1-3	1-14 par	1.59	2	38	3850	feminir	24	36800.39	167.8	84.1	110	72.5	76	208	55	129	119	0.5753	0.5811
5	1.1-3	n„o	1.62	2	38	2620	mascul	25												
6	1.1-3	1-14 tod	1.5	1	38	2660	masculin		11430.34	170.55	74.5	118	75	100	161	57	75	128	0.579	0.578
14	10	n„o	1.51	3	39	2700	feminir	54	16648.06	157.55	50.8	100.5	73.5	78	144	36	76	146	0.5778	0.577
2	1 ou -	1-14 tod	1.46	4	39	2470	feminir	22												
4	1.1-3	n„o	1.58	2		2600	feminir	20	26310.84	149.45	65.9	124.5	83.5	95	182	39	97	217	0.6768	0.6806
9	6.1-10	15 + par	1.58	2	38	2820	feminir	79	13637.55	146.2	49.6	117.5	64	98	170	63	90	84	0.5791	0.5778
8	1.1-3	n„o	1.55	1	43	3200	feminino		19999.01	162.8	60.3	112.5	68	78	230	74	140	68	0.5779	0.5835
5	1.1-3	n„o	1.54	3		2830	feminir	19	42747.59	165.5	89.2	112	76.5	88	266	40	176	287		0.5797
2	1 ou -	1-14 tod	1.6	3	40	3050	mascul	27												
5	3.1-6	n„o	1.62	5	38	3310	mascul	88												
5	3.1-6	1-14 tod	1.68	1	36	2930	masculin		17953.8	168.65	73.6	125	71.5	78	217	52	138	130	0.7166	0.5798
2	1 ou -	n„o	1.5	5		2650	mascul	138												
1	3.1-6	n„o	1.55	9		2790	feminir	208	49699.07	176.1	109.6	127	70	260	228	47	142	188	0.5908	
5	1.1-3	n„o	1.59	3	38	3100	feminir	32	15410.76	160.1	52.3	111.5	68	78	225	60	150	67	0.578	0.5793
7	1.1-3	15 + tod	1.61	1	40	3150	feminino		61173.55	168.45	113.2	117.5	70	69	231	70	140	95	0.6209	0.615
5	3.1-6	n„o	1.46	5	40	2850	feminir	40		150.7		105.5	75						0.5776	0.5763
4	1.1-3	n„o	1.59	2	41	3640	feminir	12	33921.35	159.4	78.9	113	75.5	67	222	45	149	170	0.5987	0.6064
2	1 ou -	1-14 tod	1.4	8		1440	feminir	16												
6	1.1-3	n„o	1.57	1	39	2550	masculin		13732.18	174.2	81.3	125.5	66.5						0.5739	0.5792
16	6.1-10	n„o	1.49	2	32	650	feminir	21												
13	6.1-10	n„o	1.54	1	43	3330	feminino		24903.83	160	65.6	108.5	69.5	97	182	62	102	120	0.5772	0.5814
6	1.1-3	1-14 par	1.57	3	43	2650	feminir	15	24404.83	153.25	60.4	107	64.5	91	158	55	95	64	0.584	
12	6.1-10	1-14 par	1.69	4		3680	feminir	62	13755.75	164.5	58.5	113	68.5	83	179	90	61	118	0.5658	0.5656
0	1.1-3	15 + tod	1.63	7	41	3150	feminir	54	36826.14	169.15	84.4	123.5	76.5	66	240	54	168	102	0.5867	
6	3.1-6	n„o	1.5	1	40	3070	masculin		34363.68	166	87.8	130	87	75	218	53	139	127	0.5796	0.6106
5	1.1-3	1-14 par	1.56	2	41	3660	feminir	123												
11	1.1-3	n„o	1.56	3	39	3930	mascul	60												
5	1.1-3	n„o	1.43	4	38	3250	mascul	69												
6	3.1-6	n„o	1.51	3	40	3920	mascul	27	26552.76	174.75	90.6	121	70.5	92	240	39	142	276	0.5836	0.5952
5	1 ou -	15 + tod	1.52	5	39	3700	mascul	32												
13	3.1-6	n„o	1.59	1	39	3920	masculin		14871.42	171	68.9	133.5	82.5	84	189	60	113	87	0.5771	0.5762
5	1.1-3	n„o	1.47	3	39	3310	feminir	33	9858.344	162.3	51.7	116.5	71.5	67	155	64	76	65	0.5499	0.5843
5	1.1-3	15 + tod	1.6	5	42	3150	mascul	15	13316.15	167.85	71.8	148	78.5	87	151	42	99	70	0.5803	0.5788
2	1 ou -	1-14 tod	1.57	4	37	3650	mascul	52	23980.55	171	82.4	125.5	82	88	186	48	110	150	0.5786	0.5683
0	1 ou -	15 + tod	1.63	8		3730	feminir	45												
4	1.1-3	15 + tod	1.64	3	38	2750	feminir	20	25416.65	161.55	69.7	107	75	79	180	63	107	55	0.5786	0.5848

pescmae	prenda	pfumomae	paltmae	pgesta	pidgest	ppn	psex	pint	dmgtotdxa	daltura2012	dpeso	dsysmed	ddiamed	dglicose	dcolesterol	dhdl	dldl	dtrig	dECMICE	dECMICD
4	1.1-3	n„o	1.59	4	39	3180	feminir	34	14913.05	158	50.7	96	64	80	177	63	98	50	0.5339	0.5697
9	1.1-3	15 + par	1.58	4	40	4000	mascul	11	23461.1	170.05	92.8	128.5	82.5	77	285	53	170	336	0.5883	0.5792
4	1.1-3	1-14 tod	1.54	1	40	3150	feminino													
13	10	n„o	1.63	1	38	2370	feminino													
0	1 ou -	n„o	1.47	4		1980	feminir	13												
6	1.1-3	n„o	1.51	3	36	3430	mascul	17												
3	1 ou -	n„o	1.56	1	39	3420	masculin		6657.483	158.65	62.7	120.5	83	76	191	68	111	46	0.5787	0.5798
17	3.1-6	n„o	1.62	1	42	3050	feminino		22221.12	164.15	57.9	114	75	97	205	85	105	68	0.5812	0.5574
9	10	n„o	1.62	6	26	850	feminir	29												
5	3.1-6	1-14 par	1.6	1	39	3700	masculin		12340.95	186.1	74.4	108	71	85	158	53	80	123	0.5781	0.5814
5	1.1-3	n„o	1.55	1	39	2700	feminino													
0	1.1-3	n„o	1.57	8		3700	mascul	51												
5	3.1-6	15 + tod	1.62	2	39	3100	feminir	112												
0	1.1-3	n„o	1.54	6	38	1890	feminir	56												
13	3.1-6	n„o	1.53	1	39	2800	masculin		18378.78	176.5	87.7	132	82	74	166	41	117	43	0.5717	0.5564
2	1.1-3	n„o	1.6	2	40	4060	mascul	76												
8	1.1-3	n„o	1.45	4		3350	feminir	22												
9	6.1-10	n„o		6	36	3130	mascul	34	17350.47	168.8	74.6	127	72	99	195	47	108	220	0.5815	0.5998
4	1.1-3	n„o	1.56	1		2860	feminino		17210.83	161.8	55.3	130.5	76.5	75	160	66	87	48		
11	1.1-3	1-14 tod	1.55	3	38	2230	feminir	64	23305.15	156.5	65.7	110	56.5	91	280	73	149	343		
3	1.1-3	n„o	1.44	6	41	3590	feminir	67	13991.97	160.5	53.5	106.5	68	91	149	61	73	64	0.5789	0.5785
0	1.1-3	n„o		7		1940	feminir	131	22343.55	161.3	61.4	102.5	72	80	216	51	137	140	0.5787	0.5797
10	3.1-6	n„o	1.52	2	40	3210	feminir	22	28964.56	162	72.2	111.5	70	79	184	60	105	78	0.5687	0.5782
5	1.1-3	n„o	1.52	2	37	2930	mascul	20												
1	1 ou -	n„o	1.57	1	40	3800	masculin													
8	6.1-10	15 + tod	1.61	1		2060	feminino													
3	1 ou -	1-14 tod	1.5	2		2460	mascul	14												
6	1.1-3	1-14 tod	1.53	3		3250	mascul	51												
4	1.1-3	n„o	1.53	1		3100	masculin		14370.61	180.25	80.6	117	70.5	84	142	53	84	33		0.5742
6	3.1-6	15 + tod	1.67	2		2900	mascul	28	21234.84	179.2	84.8	137	69	65	174	48	107	108	0.5784	0.5775
5	1.1-3	n„o	1.6	2	39	3230	mascul	54	22083.44	174.4	77.8	124	79.5	100	235	51	105	401	0.5769	0.5734
0	1.1-3	n„o	1.54	3		3340	mascul	41												
3	1.1-3	1-14 tod	1.64	2	40	3110	feminir	52	31850.99	170.45	75.5	103	65	87	163	41	93	119	0.5803	0.5778
17	10	n„o	1.67	1	41	4000	feminino		23584.73	168.5	73.6	115	75.5	87	210	62	132	53	0.5781	0.5774
0	1 ou -	n„o	1.56	3		3400	mascul	19	38163.4	175.35	103.2	135	88.5	106	217	46	124	161	0.5878	0.5788
9	3.1-6	n„o	1.65	1	43	3100	feminino		24563.75	164.2	65	109.5	68	96	202	58	121	114	0.5776	0.5577
3	1.1-3	n„o	1.55	4	39	2760	mascul	32	24223.58	162.65	77	124.5	77	78	218	55	130	141	0.578	0.5823
5	1 ou -	15 + tod	1.56	2		3750	feminir	21												
5	1.1-3	n„o	1.57	2	40	3500	mascul	17												
0	3.1-6	n„o	1.48	7	42	3350	feminir	77												
5	1.1-3	n„o	1.53	2	36	3000	mascul	24	34195.65	177.45	109.3	151.5	85.5	106	267	47	169	235	0.6458	0.5883
12	1.1-3	n„o		1		3050	feminino		16439.95	154.05	53.3	114.5	72.5	67	234	99	108	42	0.5789	0.5745
3	1.1-3	1-14 tod	1.6	4	40	3950	mascul	28												
5	1 ou -	1-14 tod	1.6	1	39	3070	masculin													
18	6.1-10	1-14 par	1.53	2	42	3300	feminino			161.75		94	58.5						0.5771	0.579
2	1.1-3	15 + tod	1.48	2		3190	mascul	12	2610.235	170.1	56.9	112.5	68.5	50	150	70	72	45	0.578	0.5762
9	10	n„o	1.52	2	38	3600	feminir	47												
12	6.1-10	n„o	1.72	2	41	4120	mascul	28	20260.31	183.8	81.5	126	74.5	67	260	65	159	162	0.576	0.5708

pescmae	prenda	pfumomae	paltmae	pgesta	pidgest	ppn	psex	pint	dmgtotdxa	daltura2012	dpeso	dsysmed	ddiamed	dglicose	dcolesterol	dhdl	dldl	dtrig	dECMICE	dECMICD
5	1.1-3	1-14 tod	1.5	3	40	3230	feminir	25	27803.52	150.6	67.6	115.5	74	82	232	57	145	196	0.5791	
6	1 ou -	1-14 tod	1.45	1	41	3410	feminino		23546.71	155.7	64	127	80	82	215	65	140	72		
0	1 ou -	n„o	1.47	3	39	3170	mascul	70												
1	1.1-3	1-14 tod	1.44	2		2800	mascul	35												
6	3.1-6	n„o	1.63	4	40	2250	feminir	56	21900.35	155.6	58	94	67	79	145	43	88	81	0.5948	0.5856
5	1.1-3	n„o	1.64	1	39	3370	feminino													
0	1 ou -	n„o	1.5	3	40	2760	feminir	66												
7	1.1-3	n„o	1.56	2	40	3780	mascul	44	21800.72	173.8	77.4	124.5	69.5	104	192	57	122	80		0.6031
8	10	n„o	1.59	2	39	3000	mascul	39												
12	10	n„o	1.57	3	41	4530	mascul	34	25729.81	174.4	92.6	127	74	94	200	75	115	47	0.5813	0.5941
6	1.1-3	1-14 tod	1.49	1	39	2700	feminino		20682.24	155.5	55.7	164.5	101	82	215	85	116	65	0.5705	0.5814
5	1.1-3	n„o	1.66	2	41	3600	feminir	60												
3	1 ou -	1-14 par	1.52	3	41	3500	feminir	23	48070.54	157.15	91.2	105	73.5	96	255	56	175	181	0.5656	0.5624
0	1.1-3	1-14 par	1.55	1	43	2950	masculin													
3	1.1-3	n„o	1.5	2	42	3450	feminir	62	36023.27	155.4	84.6	118	81.5	90	222	48	122	280	0.5877	0.6887
6	1.1-3	1-14 tod	1.49	1		2720	masculin		16707.79	171.15	76.7	111	64	92	207	55	130	104	0.5741	
8	1.1-3	1-14 tod	1.55	1		2540	feminino		20347.22	157.2	54.4	93.5	61.5	79	188	74	102	58	0.5787	0.5781
8	1.1-3	n„o	1.55	2	39	3450	mascul	61	29774.43	178.6	97	130	77	80	177	56	104	89	0.5743	0.5788
9	1 ou -	n„o	1.57	4	40	3600	mascul	74	16389.82	159.2	55.2	113	68	106	231	88	115	149	0.5779	0.574
5	1 ou -	n„o	1.55	5		3400	mascul	13												
4	1.1-3	15 + tod	1.63	6	37	3700	mascul	13	27368.34	174.6	89.8	130.5	79.5	74	226	46	148	168		
14	1.1-3	1-14 tod	1.64	1	39	3530	masculin													
4	1.1-3	n„o	1.52	4	36	2870	feminir	24	30445.3	158.3	74	115	83	87	169	63	88	137		
0	1.1-3	n„o	1.47	4		2550	mascul	28	3899.626	164.5	58.1	121	67.5	108	149	72	69	53	0.58	0.57
11	3.1-6	1-14 tod	1.57	1	41	2990	feminino													
7	3.1-6	n„o	1.54	1		3550	feminino		17469.63	154.45	56.6	100.5	67.5	416	138	54	78	49	0.5886	0.5957
1	1.1-3	n„o	1.57	3	40	3550	mascul	15												
10	3.1-6	n„o	1.68	1	40	4370	feminino		37788.95	172.6	79.3	111	74	80	162	57	88	56		0.5809
7	1.1-3	n„o	1.62	6	40	3170	feminir	27	17259.89	160.75	56.4	111.5	68	95	136	46	73	87	0.5783	0.5763
4	1.1-3	1-14 tod	1.51	2	40	3100	feminir	21												
5	1.1-3	1-14 tod	1.55	1	38	2700	masculin		15769.85	174.85	77.4	131	79.5	91	211	73	128	44		
7	3.1-6	n„o	1.59	4	36	1700	feminir	45												
5	1.1-3	n„o	1.59	4	43	3790	feminir	40	36068.35	169.15	92.4	122.5	83.5	78	207	65	133	56	0.5777	
5	1 ou -	1-14 par	1.5	2	38	2760	mascul	11	23862.84	170.5	79	115	61.5	84	190	44	118	162	0.5798	0.5816
5	6.1-10	n„o	1.56	11	41	4200	mascul	23	11394.75	167.35	63.2	104	72	64	192	46	121	122	0.5782	0.5715
2	1 ou -	n„o	1.55	1	38	2180	feminino		14831.15	161.25	55.5	109.5	69.5	70	135	34	78	117		
12	1.1-3	n„o	1.61	1	41	3660	feminino													
8	6.1-10	n„o	1.59	1		3960	feminino		25026.75	164.05	64.5	119.5	71.5	76	250	86	135	112	0.5871	0.5798
2	1.1-3	1-14 tod	1.57	4	35	2850	feminir	13												
9	1.1-3	1-14 tod	1.62	3	41	3300	feminir	115	13127.09	159.4	54.1	104	65	108	190	79	94	65	0.5731	0.5768
12	1.1-3	n„o	1.61	1	43	3470	feminino		48702.64	161.9	97.3	113.5	75.5	81	200	45	127	118	0.5674	
3	1.1-3	n„o	1.73	2	42	3100	feminir	147	28707.49	165.55	71.4	110.5	70	69	239	84	132	116	0.5743	0.5642
6	1 ou -	n„o	1.56	1		3650	masculin		20995.21	173	75.1	120.5	82	98	225	55	149	97	0.5817	0.5791
16	10	n„o	1.55	1	38	2600	feminino													
5	1.1-3	n„o	1.53	4	41	3500	feminir	49	29335.64	156.2	65.2	107	74	99	193	55	121	124	0.5779	0.5877
6	1.1-3	1-14 par	1.54	1	41	3350	masculin		4933.54	177.6	56.7	138.5	85	88	225	65	140	105	0.5751	0.575
17	10	n„o	1.61	1	41	3680	masculin													
8	3.1-6	n„o	1.47	1	39	3350	masculin													

pescmae	prenda	pfumomae	paltmae	pgesta	pidgest	ppn	psex	pint	dmgtotdxa	daltura2012	dpeso	dsysmed	ddiamed	dglicose	dcolesterol	dhdl	dldl	dtrig	dECMICE	dECMICD
8	1.1-3	n„o	1.58	3	38	3680	feminir	48	13660.97	163.15	49.2	110	75.5	83	178	59	110	63	0.5778	0.5777
5	3.1-6	n„o	1.59	2	39	3870	mascul	21												
2	1.1-3	n„o	1.58	1	39	4200	masculin													
5	1.1-3	1-14 par	1.55	2	40	3500	mascul	50												
5	1.1-3	n„o	1.51	3	38	3380	mascul	15	16260.1	162.2	70.3	128.5	87.5	83	198	67	109	100	0.6045	0.6057
2	1 ou -	n„o	1.51	4	42	3600	mascul	29	11792.61	171.35	66	127	80.5	100	172	65	90	94	0.5457	0.5481
12	6.1-10	n„o	1.65	4	39	3850	feminir	23												
5	1.1-3	1-14 tod	1.44	1		2020	masculin													
8	1.1-3	1-14 tod	1.65	1	38	3600	feminino			164.85		98	66						0.5773	0.5911
4	1.1-3	n„o	1.58	1	41	3060	feminino		43726.83	165.9	101.9	138	92.5	89	226	54	136	210		
4	3.1-6	n„o	1.65	2	40	3300	feminir	66	30441.03	152	70.7	124	75.5	139	238	75	144	94		0.5879
4	1.1-3	n„o	1.6	2		3020	feminir	20	24663.49	151.75	58.7	123.5	81.5	82	165	65	90	71		0.5612
3	1 ou -	n„o	1.52	1		2820	feminino													
5	1.1-3	n„o	1.51	2	38	2850	feminir	31	16858.34	148.9	54.9	134.5	97	80	182	65	103	65		
16	3.1-6	15 + tod	1.6	6	39	3100	feminir	34	29044.21	159.05	71	98.5	68	94	190	72	100	108		
11	1.1-3	n„o	1.56	1		3450	masculin													
5	6.1-10	1-14 par	1.61	2		3650	feminir	24	19864.96	163.3	59.2	124	79.5	79	239	89	114	179	0.5875	0.5541
3	1.1-3	n„o	1.45	5	39	3480	mascul	87												
4	1.1-3	1-14 par	1.51	1	39	2950	feminino		19562.6	157.95	53.5	125.5	93	98	220	78	123	71	0.5578	0.5691
7	1.1-3	15 + tod	1.61	2	41	3200	mascul	67												
3	1.1-3	1-14 tod	1.64	6		4170	mascul	24	15118	172.35	73.5	115	69	99	165	64	79	107	0.5795	0.5774
3	1 ou -	1-14 tod	1.59	5	40	3480	mascul	12												
15	6.1-10	1-14 tod	1.59	2	39	3000	mascul	15												
8	6.1-10	n„o	1.5	1	40	2460	masculin		40268.3	156.2	95.7	151	96	112	204	41	100	380	0.6439	0.6206
11	3.1-6	1-14 tod	1.6	4	40	3200	feminir	47												
3	1 ou -	n„o	1.52	2	39	3030	feminir	16	12377.98	152.15	49.5	112.5	67.5	79	182	70	101	40	0.578	0.5781
3	1 ou -	n„o	1.48	1	35	2760	masculin													
9	6.1-10	n„o	1.51	3	38	3700	feminir	26	27253.41	160.7	65.5	109.5	62.5	85	178	77	88	66	0.5829	0.579
10	3.1-6	n„o	1.69	1		3700	masculin			178.1	123.9	132.5	88	98	282	49	155	268	0.579	0.586
4	1 ou -	n„o	1.65	3	36	3100	mascul	57	18024.8	183.7	78.8	135.5	83.5	87	163	38	92	132	0.5715	0.5783
0	1.1-3	n„o	1.55	11	39	3350	feminir	32	27413.63	165.5	70.8	110	75	90	232	78	124	183	0.5716	0.5757
4	1.1-3	1-14 par	1.56	4		3400	mascul	13	3813.742	173.15	63.1	104	70	83	102	30	60	71	0.6705	0.7559
7	1.1-3	n„o	1.53	1	39	3720	masculin		28535.72	170.75	80.2	114	64	84	203	47	134	103	0.6549	0.5832
12	6.1-10	n„o		2	39	3130	feminir	45												
4	1.1-3	1-14 tod	1.47	3		3550	feminir	62	24902.27	162.35	64.5	110.5	68	103	143	62	66	96	0.5784	0.575
4	1.1-3	n„o	1.52	1	39	3600	masculin													
5	1.1-3	n„o	1.58	9	31	750	mascul	27												
3	1.1-3	n„o	1.62	2	43	3200	feminir	30	22397.27	165.2	63.5	129	74	83	148	52	82	85	0.5801	0.5749
17	3.1-6	n„o	1.58	2	38	3650	feminir	25	20448.3	160.9	56.6	110.5	65	77	232	80	131	100	0.5806	0.5697
5	1 ou -	1-14 tod	1.53	3		2430	feminir	95												
4	1 ou -	n„o	1.52	1	39	3450	masculin		29277.14	167	86.2	125	85	127	269	54	177	324		0.5792
7	1.1-3	n„o	1.58	5	37	3230	mascul	20	5735.35	175.2	61.2	114.5	74	78	197	61	124	38	0.5795	0.5793
2	1 ou -	n„o	1.5	1	39	3450	masculin		19692.51	171.7	78.3	119	61.5	82	130	32	79	91	0.5771	0.5796
5	3.1-6	n„o	1.48	4		3800	feminir	39	21617.54	153.1	57.7	101	62.5	78	178	72	94	48	0.5777	0.57
7	1.1-3	n„o	1.59	4	36	2540	feminir	82	17301.05	159.5	55.1	122	74	100	210	75	117	84	0.5778	0.5779
7	1.1-3	15 + tod	1.57	2	41	3750	mascul	21	23551.49	178.5	88.5	112	73	84	175	44	90	218	0.5786	0.5774
5	1.1-3	n„o	1.64	2		3710	mascul	37												
14	1.1-3	1-14 tod	1.64	1		3150	masculin													

pescmae	prenda	pfumomae	paltmae	pgesta	pidgest	ppn	psex	pint	dmgtotdxa	daltura2012	dpeso	dsysmed	ddiamed	dglicose	dcolesterol	dhdl	dldl	dtrig	dECMICE	dECMICD
2	1.1-3	n„o	1.57	6	38	3200	feminir	36												
0	1.1-3	n„o	1.6	3	41	2800	mascul	36												
6	1 ou -	n„o	1.67	8	42	4960	mascul	22												
12	1.1-3	n„o	1.62	1	41	2630	feminino		46513.18	161.75	100.9	128.5	85	85	187	40	102	256	0.6484	0.5898
4	1.1-3	n„o	1.55	6	41	3150	feminir	39	30272.98	163.75	73.9	112	66.5	82	222	55	145	117	0.576	0.585
8	1.1-3	1-14 tod	1.62	2		3600	mascul	12	19223.95	179.9	85.8	137.5	73	68	239	48	148	194	0.581	0.5787
3	1 ou -	n„o	1.57	1		3070	feminino													
8	10	15 + tod		3	38	2690	mascul	40	29292.96	171.55	101.8	136	82	132	219	33	83	728	0.5825	0.5841
5	6.1-10	n„o	1.54	2	41	3150	mascul	21	20789.94	171.2	75.3	138	70	83	171	56	88	133	0.5806	0.6397
11	6.1-10	n„o	1.5	2	41	3250	feminir	34												
3	1.1-3	n„o	1.56	4	40	3670	mascul	18												
0	1 ou -	n„o	1.58	2		3200	mascul	70												
5	3.1-6	n„o	1.63	1	39	3300	feminino													
5	1 ou -	15 + tod	1.59	3		2600	feminir	30												
5	1.1-3	1-14 tod	1.67	1	37	3200	masculin													
9	6.1-10	n„o	1.63	2	40	4400	mascul	62												
9	3.1-6	15 + tod	1.62	4	43	4030	mascul	80												
9	3.1-6	1-14 par	1.7	1	43	3550	masculin													
8	3.1-6	n„o	1.5	2	39	2750	masculin													
10	6.1-10	n„o	1.58	2		3000	feminir	14												
10	3.1-6	n„o	1.56	1	40	2780	masculin		9902.673	174.6	60.7	127.5	79.5	97	250	59	161	170	0.5711	
5	1.1-3	n„o	1.63	1	41	3550	masculin													
9	10	n„o	1.63	3	40	3800	feminir	52	16416.73	170.55	58.1	110.5	70.5	91	197	70	111	63	0.5786	0.574
5	1.1-3	n„o	1.61	2	42	3650	mascul	48												
6	1.1-3	n„o	1.62	1	38	3200	masculin													
4	1.1-3	n„o	1.57	2	42	3100	mascul	76												
5	1.1-3	1-14 tod	1.66	2	40	2500	feminir	57												
5	1 ou -	n„o	1.51	2	38	3000	mascul	29												
5	1 ou -	1-14 tod	1.6	1	41	3690	masculin		18408	165.9	82	136.5	73	119	263	44	131	292	0.5806	0.5817
5	1.1-3	1-14 tod	1.52	2	42	3400	mascul	73	8339.074	170.35	65.6	113	69.5	80	186	69	107	78	0.5749	0.579
7	1.1-3	n„o	1.56	2	39	3010	feminir	32	23823.33	161.95	64.3	112.5	70.5	70	205	54	105	273	0.5776	0.6033
6	1.1-3	n„o	1.56	1	39	3750	feminino		58781.43	161.95	106.5	108.5	77.5	79	180	67	100	62		0.5769
6	1.1-3	1-14 par	1.58	4		3700	feminir	11	21577.29	160.8	61.2	109.5	72.5	84	199	55	123	116	0.5768	0.5756
5	1.1-3	15 + tod	1.54	2	40	3460	mascul	28	5128.48	166.3	56.6	113.5	70.5	86	181	58	100	137	0.5781	0.5759
18	3.1-6	n„o	1.65	1	42	3380	masculin		6088.981	181.35	61.4	115.5	67	91	149	51	90	70	0.5742	0.5744
3	1.1-3	n„o	1.62	3	39	3260	mascul	14	15417.3	183.6	72.6	122	72	84	154	43	97	106		0.5763
4	1.1-3	n„o	1.47	2		2630	mascul	18	9661.269	166.25	56.6	125.5	72	78	171	56	92	107	0.5772	0.576
9	3.1-6	n„o	1.66	2		4250	feminir	41	18949.21	168.15	70.1	112	70	76	148	51	89	53	0.5784	0.5812
9	1.1-3	n„o	1.63	2	38	3070	feminir	24												
11	3.1-6	1-14 tod	1.53	3	39	3600	mascul	43												
4	1.1-3	n„o	1.64	2	40	4100	mascul	18	34965.61	178	110.4	117	71.5	83	212	48	138	145		0.5932
7	3.1-6	1-14 tod	1.65	2	38	3800	mascul	31		181.5	109.6	116	85.5	110	196	45	91	335	0.5767	0.579
12	6.1-10	n„o	1.61	3	38	3750	mascul	40												
8	1.1-3	n„o	1.6	1	39	3060	masculin													
2	1.1-3	1-14 tod	1.49	6		3350	feminir	31												
3	1.1-3	1-14 tod	1.48	4		3300	mascul	18												
0	1.1-3	n„o	1.52	11		2980	feminir	103	6068.988	166.15	43.9	125.5	87	81	180	79	92	51		
5	1.1-3	15 + tod	1.55	8	40	2830	mascul	24	16456.81	160.9	62.2	118	67.5	79	230	73	139	84	0.5604	

pescmae	prenda	pfumomae	paltmae	pgesta	pidgest	ppn	psex	pint	dmgtotdxa	daltura2012	dpeso	dsysmed	ddiamed	dglicose	dcolesterol	dhdl	dldl	dtrig	dECMICE	dECMICD
2	1 ou -	n„o	1.6	2	37	3470	feminir	61												
8	1.1-3	15 + tod	1.56	3	41	3650	feminir	13												
4	1 ou -	n„o	1.62	2	40	3270	mascul	17	12550.79	168.25	74.2	119	69	105	183	61	98	113		
8	1.1-3	n„o	1.62	2	39	3370	feminir	26	29556.26	162.4	72.4	116	85	94	181	77	83	137	0.578	0.5767
0	1 ou -	n„o	1.54	2	39	2050	mascul	50	20596.13	179.35	87.4	120	71.5	84	168	58	98	104	0.5713	0.5776
3	1 ou -	1-14 par	1.56	3	40	3150	feminir	12	25788.15	157.45	66.3	108.5	72.5	74	140	48	84	47	0.5735	0.6281
4	1 ou -	n„o	1.44	2		2050	feminir	20	12168.85	146.4	45.3	93	60	90	177	38	109	106	0.5645	0.5799
5	3.1-6	n„o	1.53	4	38	3600	feminir	50		157.65	120.1	124.5	93	77	213	45	146	132		
5	1.1-3	1-14 tod	1.52	5	40	2720	feminir	17	38788.05	160.4	88.2	105.5	58.5	129	218	58	143	87		
3	1.1-3	n„o	1.65	2	40	4230	mascul	31	9922.255	190	71.9	121	76.5	66	163	32	113	130	0.5807	0.5789
4	1.1-3	n„o	1.56	4		3150	mascul	47	16878.98	174.95	70.6	139.5	73	84	195	61	111	97	0.5553	0.6043
3	1 ou -	15 + tod	1.55	1		2900	feminino		17047.85	163.7	57	112.5	68	87	153	65	78	70	0.5801	0.5515
16	10	1-14 tod	1.57	2	38	2750	mascul	13												
6	1.1-3	1-14 tod	1.57	2	39	3250	feminir	40												
11	3.1-6	1-14 par	1.57	1	41	3260	masculin													
12	3.1-6	n„o	1.6	1	38	4450	masculin		8574.268	182.25	74.2	133	77	88	148	68	69	40	0.5771	0.5782
9	3.1-6	n„o	1.58	2	42	3340	feminir	24	57195.49	168.6	110.5	123	78	90	200	63	122	71	0.5808	0.5783
5	1.1-3	n„o	1.52	1	38	2870	masculin													
11	6.1-10	n„o	1.5	2		2800	feminir	43												
4	1.1-3	n„o	1.53	3	42	2600	feminir	14												
0	3.1-6	n„o	1.55	2	37	4270	mascul	85												
15	3.1-6	1-14 tod	1.59	1	36	2510	feminino		26163.52	159.5	64.4	110	72.5	100	189	71	110	48	0.5807	0.5493
11	3.1-6	n„o	1.58	2	41	2700	feminir	16												
5	6.1-10	n„o	1.52	3	36	3000	mascul	169	14552.58	173.35	86.3	125	67.5	72	180	85	80	67	0.5796	0.6014
0	1 ou -	n„o	1.48	1		2750	feminino		14757.65	155.4	50.9	110.5	63.5	73	254	66	173	92		
1	3.1-6	n„o	1.63	2	39	3870	mascul	86	46876.74	188.95	118.1	139	82	96	184	54	115	99	0.5797	0.5788
5	1.1-3	1-14 par	1.57	2	40	3500	feminir	53												
5	1.1-3	n„o	1.51	4		4070	mascul	22	27670.94	171.3	96.7	139	76	141	298	47	155	407	0.5801	0.5772
16	6.1-10	n„o	1.54	1	38	2860	feminino		28653.18	167.35	72.6	111.5	78	84	215	49	136	198	0.5799	0.5822
9	3.1-6	15 + tod	1.57	1	39	3570	masculin		25352.7	180.3	86.2	125	72.5	103	211	50	127	159	0.5903	0.5766
4	10	n„o		3	37	2630	mascul	16	11476.2	178.8	72.2	117	75.5	88	172	64	98	40	0.578	0.5781
1	1.1-3	n„o	1.59	3		2440	mascul	106												
10	3.1-6	n„o	1.65	4	38	4200	mascul	24	17910.04	182.4	82.3	117.5	63.5	76	175	56	103	78	0.64	0.5909
9	3.1-6	1-14 tod	1.53	2	38	3750	mascul	33												
1	1 ou -	n„o	1.49	1		3180	masculin													
0	1 ou -	n„o	1.43	1	33	1630	masculin													
7	1.1-3	n„o	1.58	2	38	2920	feminir	32	31086.3	164	75.2	106.5	65	85	191	57	113	107		0.5739
12	3.1-6	1-14 tod	1.61	2	39	2750	mascul	37	4186.39	181.45	67.9	103	52	110	139	38	67	170		
10	1.1-3	1-14 tod	1.6	2		2620	mascul	13												
9	10	n„o	1.56	4	39	3180	feminir	45												
5	1 ou -	1-14 tod	1.55	3		2580	feminir	62	44847.93	157.85	97.7	116	72	98	238	64	156	85		
1	1.1-3	n„o	1.54	8	40	4440	feminir	25	26841.74	161.6	66.6	103.5	71.5	72	135	50	76	44	0.5783	0.5744
8	3.1-6	n„o	1.51	1	42	3730	feminino		49285.08	161.6	101.8	129.5	87	79	228	70	140	106		
12	3.1-6	n„o	1.49	4	39	2630	feminir	122		162.2										
3	1 ou -	n„o	1.66	1	39	2930	feminino													
6	1.1-3	n„o	1.6	4		3100	mascul	57												
6	1.1-3	1-14 par	1.61	3	40	4400	masculin		13116.06	176.6	70.6	124	75.5	75	141	50	82	46	0.5707	0.5798
0	1 ou -	n„o	1.56	3		3590	feminir	37												

pescmae	prenda	pfumomae	paltmae	pgesta	pidgest	ppn	psex	pint	dmgtotdxa	daltura2012	dpeso	dsysmed	ddiamed	dglicose	dcolesterol	dhdl	dldl	dtrig	dECMICE	dECMICD
4	3.1-6	n,,o	1.64	4	43	2950	mascul	135	10674.02	181.2	65.1	115.5	71.5	86	153	47	99	39	0.578	0.5742
3	1 ou -	n,,o	1.64	2	37	3250	feminir	17	6732.61	171.65	51.9	102.5	74.5	65	125	40	59	84	0.5776	0.5765
11	10	n,,o	1.56	4	38	3300	feminir	56	17764.56	162.55	51.2	100	59	66	165	39	115	58	0.5782	0.5781
5	1.1-3	n,,o	1.58	3		3500	mascul	31												
5	3.1-6	n,,o	1.57	2		3600	mascul	71												
16	6.1-10	n,,o	1.48	1	37	2770	masculin		16278.17	164.7	70.1	133.5	83	74	219	53	147	90	0.576	0.578
7	1 ou -	1-14 tod	1.58	2	41	3060	mascul	28	36509.61	176.05	100.3	128	88.5	91	216	59	139	88		
0	3.1-6	15 + tod	1.55	1		1430	masculin													
5	10	n,,o	1.6	5	40	3650	mascul	14	29344.72	176.5	85	111	67	76	155	42	91	135	0.5844	0.5855
12	6.1-10	n,,o	1.57	3	38	4280	feminir	72	34516.15	162.2	78.9	106	66.5	79	183	71	97	67	0.5783	0.5786
6	1.1-3	n,,o		4	39	3110	mascul	77												
4	1.1-3	n,,o	1.51	1	40	3050	masculin													
10	1.1-3	n,,o	1.6	1		3730	masculin													
9	10	n,,o	1.56	4	41	4080	mascul	30	13499.22	175.75	72.6	133	68.5	94	175	77	82	91	0.5742	0.5779
5	3.1-6	n,,o	1.58	9	36	3350	feminir	110	31906.31	162.25	72.4	111.5	61.5	67	190	64	108	102	0.5788	0.5374
7	1.1-3	n,,o	1.54	5	40	2730	feminir	30	39656.76	152.95	76	118	85.5	86	217	78	118	143	0.5797	0.5776
4	1.1-3	1-14 tod	1.61	2	40	3550	mascul	76	16293.48	172.6	77.8	135	94.5	83	144	63	62	79	0.5791	0.5789
4	1.1-3	1-14 tod	1.46	1		2480	masculin													
5	1.1-3	n,,o	1.52	2	39	4190	mascul	25												
3	1 ou -	n,,o	1.42	4		2290	mascul	11												
2	1.1-3	n,,o	1.61	3	37	3600	feminir	17												
5	3.1-6	n,,o	1.55	4		1200	feminir	85												
5	1.1-3	n,,o	1.56	1	39	3350	masculin		18830.55	173.5	72.9	113.5	68	96	167	42	94	134	0.5432	
3	1.1-3	1-14 par	1.64	3	37	3820	mascul	30												
11	6.1-10	1-14 par	1.64	1		3420	masculin													
8	1.1-3	n,,o	1.54	1	36	2450	masculin			185.3	121.7	122	77	87	173	45	96	161	0.5822	0.5796
12	6.1-10	n,,o		3	38	3100	feminir	123	25006.3	167.2	65.2	107.5	70.5	83	165	48	102	47	0.5783	0.5787
7	1 ou -	1-14 tod	1.55	2	43	1350	feminir	14												
3	1 ou -	15 + tod	1.58	2	41	2050	feminir	93	29221.68	160	71.6	123	71.5	99	182	38	91	377		
1	3.1-6	n,,o	1.49	1	38	2550	masculin		17488.31	174.1	74.3	107.5	64	76	187	56	118	63	0.5778	0.5781
4	1 ou -	1-14 par	1.58	3	42	3950	mascul	56	15840.42	170.65	82	114	65.5	95	177	59	107	55	0.5775	0.5796
1	1 ou -	n,,o	1.47	6	37	2700	feminir	93												
1	1.1-3	15 + tod	1.55	3		3700	mascul	61	25348.06	163.1	73.7	124	82.5	83	220	64	116	242	0.5782	
9	1.1-3	1-14 tod	1.66	3	38	3610	mascul	30	13568.13	185.6	80.4	146	80	93	187	74	97	68	0.5837	0.5448
3	1.1-3	n,,o	1.57	1	39	2980	masculin		29340.29	178.65	97.4	132	84	75	172	33	101	203	0.5793	0.5847
12	6.1-10	n,,o	1.58	3	39	3470	mascul	42	41692.61	185.6	107.7	133.5	77	91	143	50	79	60		
8	1.1-3	1-14 tod	1.59	1	40	2520	feminino		24343.58	160.15	61.9	144	96	103	179	63	97	88	0.5797	0.5752
6	10	n,,o	1.6	1	38	4300	masculin													
8	1 ou -	n,,o	1.52	1		2970	feminino		19832.67	152.75	57.8	122	72.5	86	207	67	131	51	0.5758	0.5777
2	1 ou -	1-14 tod	1.55	1		3460	masculin		13012.16	174.75	71.8	130.5	81	101	145	46	86	89	0.578	0.5751
4	1.1-3	n,,o	1.5	3		4000	feminir	17	32528.32	159.65	71.7	119	84	71	148	53	72	82	0.5784	
3	1.1-3	n,,o	1.56	3	38	3000	mascul	143	19537.44	175.4	85.3	142.5	97	82	199	49	135	61	0.6428	0.5814
4	1.1-3	15 + tod	1.5	3	39	2800	mascul	27												
5	1 ou -	n,,o	1.54	1	40	3140	masculin		20756.94	178.5	82.7	122.5	81.5	93	137	52	65	70	0.572	0.5658
5	1 ou -	n,,o	1.51	1	41	3750	masculin		40046.94	172.6	106.6	143.5	87.5	83	175	48	98	164		
8	3.1-6	n,,o	1.6	2	37	3300	mascul	12	10206.01	177.55	69.4	121.5	67	88	156	49	92	48	0.5758	0.578
3	1.1-3	n,,o	1.59	2	37	2950	feminir	70	25545.48	167.8	66.8	104.5	75	90	205	68	107	191		
9	1.1-3	1-14 par	1.6	2	39	2700	feminir	23	22689.07	160	61.6	136	84.5	84	135	36	74	117	0.5998	0.561

pescmae	prenda	pfumomae	paltmae	pgesta	pidgest	ppn	psex	pint	dmgtotdxa	daltura2012	dpeso	dsysmed	ddiamed	dglicose	dcolesterol	dhdl	dldl	dtrig	dECMICE	dECMICD
5	1.1-3	n,,o	1.5	1	38	3070	feminino		41160.9	154.25	87.5	114.5	75.5	86	241	64	140	203	0.5795	0.6071
4	1.1-3	n,,o	1.54	2	40	3350	feminir	38												
4	1 ou -	1-14 tod	1.57	1	38	3500	masculin													
0	1 ou -	n,,o	1.68	4	42	3100	feminir	98		166.85	122.4	128.5	86.5	96	160	53	88	78	0.6114	
6	1.1-3	n,,o	1.52	3	40	3000	feminino		23909.37	166.85	63.6	118.5	74.5	76	186	71	101	43	0.5776	0.5795
5	3.1-6	n,,o	1.52	3	41	3800	mascul	78												
6	1.1-3	n,,o	1.56	1		3220	masculin		13599.88	171.8	77.1	116	72.5	66	186	60	110	62	0.6324	0.5788
4	1 ou -	n,,o	1.63	2	40	3380	feminir	36												
0	3.1-6	n,,o	1.55	4	41	3550	feminir	46		156.35	93.7	104	69.5	91	195	45	122	141	0.5785	0.5789
9	1.1-3	n,,o	1.54	2	40	3400	mascul	23												
7	1.1-3	n,,o	1.57	2	37	3430	mascul	22												
9	3.1-6	1-14 tod	1.66	2	41	4030	feminir	21	30326.16	171.8	70.8	108.5	74.5	84	168	64	84	94	0.5791	0.5777
5	1.1-3	15 + tod	1.58	3	41	3060	feminir	13												
11	1.1-3	n,,o	1.75	1	36	3430	feminino					124.5	82							
3	1.1-3	n,,o	1.58	1	39	3600	masculin		5342.974	163.65	66.1	136	74	113	195	54	128	70	0.5776	0.578
18	3.1-6	n,,o	1.57	1	39	3000	feminino													
5	1.1-3	n,,o	1.56	1	39	3120	feminino		31233.65	157.05	74.5	117	78.5	74	150	58	82	75	0.5869	0.5781
5	3.1-6	n,,o	1.63	1	39	3220	masculin		22997.6	176.7	85.2	128.5	82	81	179	58	104	99	0.6033	0.5854
7	1.1-3	n,,o	1.56	2	37	3020	mascul	19	3617.07	177	58.6	124.5	69.5	67	156	60	87	40	0.5782	0.5781
13	3.1-6	n,,o	1.57	1	40	3250	feminino													
6	1 ou -	15 + tod	1.55	1	39	3000	feminino		35959.54	162	80.7	114	72.5	91	210	72	117	97	0.5796	0.5891
9	1 ou -	n,,o	1.57	1	40	3400	masculin													
9	6.1-10	n,,o	1.57	2	39	3300	feminir	17	32916.62	166.1	69.8	103	74.5	75	189	58	97	180		
5	3.1-6	1-14 tod	1.55	5		3550	feminir	125												
3	1 ou -	n,,o	1.63	5	41	3500	mascul	28	33608.8	172.65	106.1	135.5	82	95	191	34	110	263	0.5901	0.5792
4	1.1-3	1-14 tod	1.62	3	42	2950	mascul	15	21084.63	176.7	83.6	111	60	88	150	51	86	90	0.5786	0.5801
8	3.1-6	1-14 tod	1.65	1	40	2800	feminino													
8	1.1-3	1-14 par	1.6	1	37	3000	masculin		40244.92	182.55	119.4	161.5	95.5	92	220	52	88	434	0.6101	0.6
2	1.1-3	n,,o	1.56	2	43	2770	feminir	44	42685.05	159.6	84.5	122.5	79	65	225	55	141	158	0.5812	0.5787
5	6.1-10	n,,o	1.51	3	38	2990	feminir	118												
5	1 ou -	1-14 tod	1.51	1	41	3350	feminino		26465.62	155.05	59.7	116.5	82.5	104	248	55	178	83	0.579	0.5729
6	1.1-3	1-14 tod	1.56	1		2980	masculin													
3	1 ou -	n,,o	1.65	3	40	3900	feminino		39218.12	170.1	91.4	116.5	74.5	65	149	61	66	85	0.5946	0.5796
11	1.1-3	1-14 tod	1.51	1	42	2600	feminino		13227.36	159.2	50	116.5	66.5	92	217	74	120	94	0.578	0.5764
2	1 ou -	n,,o	1.46	3	40	3250	mascul	54	7360.153	161.45	55.7	125.5	81.5	90	196	56	128	45		0.5796
4	1.1-3	n,,o	1.55	3		3450	masculin													
3	1 ou -	15 + tod	1.59	1		3600	feminino		13026.77	160.1	57.3	118	72.5	73	203	58	130	71	0.5783	0.5786
0	1 ou -	n,,o	1.56	2		3270	feminir	18												
16	10	n,,o	1.58	2	38	3500	feminir	16	22498.82	166.75	66.3	106	58.5	87	166	78	75	50	0.6482	0.5808
4	1 ou -	n,,o	1.61	2	39	3250	feminir	16	48119.25	167.15	103.8	133.5	77.5	88	229	56	142	208	0.5857	0.6585
2	1.1-3	n,,o	1.53	3	37	3700	feminir	18	54542.24	158.6	100	114.5	68.5	98	157	56	76	156		0.5789
9	1.1-3	1-14 tod	1.58	2	41	3650	feminir	12												
5	1.1-3	1-14 tod	1.62	4		2900	feminir	29	29645.67	159.5	74.8	121	77	138	158	46	96	88	0.5613	
3	1.1-3	n,,o	1.56	1	41	3040	masculin													
8	1.1-3	1-14 tod	1.66	1	40	4000	masculin		18319.76	181.2	77.5	120	80.5	87	215	46	127	196	0.5811	0.5856
3	1 ou -	n,,o	1.48	4	38	2940	feminir	92	16814.14	159.6	54.7	115	71.5	79	119	44	60	74	0.5591	0.5785
6	1.1-3	n,,o	1.52	4	39	3000	feminino													
15	6.1-10	n,,o	1.58	1	38	2960	masculin													

pescmae	prenda	pfumomae	paltmae	pgesta	pidgest	ppn	psex	pint	dmgtotdxa	daltura2012	dpeso	dsysmed	ddiamed	dglicose	dcolesterol	dhdl	dldl	dtrig	dECMICE	dECMICD
5	1.1-3	15 + tod	1.56	2		3150	feminir	41	36393.74	165.45	79.6	137.5	85	88	175	50	101	118	0.5784	0.576
0	1 ou -	15 + tod	1.48	1		2780	masculin		3577.36	171.45	61.1	119	62.5	82	105	37	57	52	0.5793	0.583
9	1.1-3	n„o	1.58	3	37	3800	mascul	56	18898.57	181.75	86	147	86	67	168	42	114	60	0.6197	0.6154
5	1.1-3	1-14 tod	1.59	2		3000	mascul	49	17999.39	170.6	73	125	79.5	105	148	42	72	149	0.577	0.5737
6	1.1-3	n„o	1.55	1	41	3200	feminino													
2	1 ou -	n„o	1.5	4		3030	feminir	13												
5	3.1-6	n„o	1.55	5	40	3500	feminir	99												
9	1.1-3	n„o	1.51	1	37	2940	feminino													
5	1 ou -	1-14 tod	1.52	1	36	3050	masculin		11179.08	169.7	62.6	122	70	87	190	71	98	82	0.5609	0.579
4	1 ou -	1-14 tod	1.55	3		3380	feminir	54												
5	1 ou -	n„o	1.47	4	38	2680	mascul	53												
16	10	n„o	1.6	1	37	3100	masculin		41547.57	179.15	110.5	134.5	76.5	84	182	58	110	59	0.5581	
5	1.1-3	n„o	1.52	5	38	3320	mascul	32												
6	1.1-3	n„o	1.58	1		2380	feminino													
4	1 ou -	1-14 tod	1.56	1	38	2920	feminino		21909.83	158.15	57	103	71.5	75	159	65	78	52	0.5713	0.5783
10	1.1-3	n„o	1.58	1	38	3350	masculin		15950.61	173.3	77.4	114.5	62	87	171	63	79	162	0.6218	0.5792
6	1.1-3	1-14 tod	1.51	3	42	3800	feminir	41	33759.02	164.5	81.1	102.5	58.5	105	279	50	174	298	0.5892	0.5626
3	1.1-3	n„o	1.61	2	43	4040	feminino													
15	10	n„o	1.65	2	38	3500	mascul	24	51164.1	183.1	119.4	129	85	84	174	47	107	121	0.5796	0.5795
6	1.1-3	n„o	1.62	2	39	4620	mascul	60	39805.09	181.1	110.6	129	82.5	100	206	44	123	215	0.5933	0.5919
5	3.1-6	n„o	1.65	3	41	3840	feminir	60	41473.88	164.4	81.7	118	75.5	81	175	72	83	110	0.5779	0.5775
0	1.1-3	15 + tod	1.5	4	39	2750	mascul	21												
13	1 ou -	n„o		2	38	2980	feminir	21	33210.05	149.8	66.5	99.5	68.5	115	188	57	104	128		
4	1 ou -	1-14 tod	1.54	3	39	2800	feminir	35												
1	1.1-3	1-14 tod	1.48	3		2700	feminir	35												
8	1.1-3	1-14 tod	1.5	4		3550	mascul	24												
6	1.1-3	n„o	1.58	1	39	3200	masculin													
2	1 ou -	n„o	1.55	4	38	2950	feminir	73												
7	1.1-3	n„o	1.55	1	41	3700	feminino		36158.92	167.9	83.6	121	81.5	80	215	54	126	193	0.5784	0.5726
4	1.1-3	n„o	1.49	5	39	4000	feminir	13												
5	1 ou -	1-14 tod	1.57	3	38	2840	feminir	61	28358.44	155.7	68	103	66	83	195	64	104	124	0.5793	0.5447
0	1 ou -	1-14 par	1.52	3	38	2600	feminir	12												
10	3.1-6	n„o	1.46	2	41	3540	mascul	57	16297.17	166.2	73.8	126	72.5	100	230	61	139	199	0.5768	0.5801
12	3.1-6	1-14 par	1.65	1	39	2900	feminino													
5	1 ou -	n„o	1.56	4		2980	masculin													
7	6.1-10	1-14 tod	1.52	2	40	3050	mascul	18	22746.95	173.85	81.9	130.5	71.5	89	194	75	102	63	0.5785	0.5809
5	1 ou -	n„o	1.42	3		3100	mascul	26	15834.75	157.6	66.8	121.5	75.5	95	143	47	87	50	0.5261	0.5746
9	1.1-3	n„o	1.6	1	43	3350	feminino		26004.38	160.8	65.5	113.5	77.5	128	215	95	107	70	0.5751	0.5774
7	3.1-6	n„o	1.52	1	41	3110	feminino													
4	1.1-3	n„o	1.64	4	42	3440	feminir	36	25024.47	171.7	66.2	105	72	68	140	60	66	65	0.5629	0.5777
						2650	masculin													
16	3.1-6	n„o	1.76	1	40	3750	masculin		20436.25	188.15	89.1	137	75	82	170	62	91	67	0.5659	0.578
2	1.1-3	1-14 tod	1.55	2	40	3600	mascul	27				127	70							
9	1.1-3	n„o	1.56	5	40	3090	feminir	15												
5	1 ou -	1-14 tod	1.58	2	38	3700	mascul	25												
7	3.1-6	n„o	1.53	1	39	1990	feminino			144.5	57	104	65.5	66	198	52	128	94	0.5789	0.5669
11	3.1-6	1-14 tod	1.61	2		2600	feminir	26	34980.31	163.75	74.3	109	71	64	204	90	98	67	0.5737	0.5781
3	1.1-3	n„o	1.58	4	27	1070	feminir	12												

pescmae	prenda	pfumomae	paltmae	pgesta	pidgest	ppn	psex	pint	dmgtotdxa	daltura2012	dpeso	dsysmed	ddiamed	dglicose	dcolesterol	dhdl	dldl	dtrig	dECMICE	dECMICD
3	1.1-3	n„o	1.65	1	39	2880	masculin		10251.67	174.25	64.7	114	68.5	92	186	46	123	101	0.5785	0.5782
5	3.1-6	n„o	1.53	3	39	3880	feminir	112												
3	1.1-3	1-14 tod	1.57	4		2700	feminir	39	35396.5	159.9	71.5	102	72	81	230	46	155	87	0.5844	0.5794
4	1.1-3	n„o	1.53	4	40	4100	mascul	13	12461.11	173.65	74.6	115.5	63	87	186	64	109	71	0.5781	0.5781
4	1 ou -	1-14 tod	1.6	1	40	3520	feminino		20629.94	164.8	61.2	95.5	54.5	77	158	54	75	140	0.578	0.5776
9	3.1-6	1-14 par	1.6	3	39	3580	mascul	24												
2	1.1-3	n„o	1.63	2	41	3500	mascul	63												
4	1.1-3	1-14 tod	1.57	1		2700	masculin					102	67							
5	1.1-3	1-14 tod	1.54	2	39	3350	mascul	34	10637.79	177.6	71.4	109.5	66.5	86	163	60	91	69	0.5779	0.5776
4	1.1-3	n„o	1.59	1	40	3260	feminino		27094.76	158	70	108.5	70.5	102	214	75	122	73	0.5794	0.5811
0	1 ou -	15 + tod	1.54	3	41	3790	mascul	12	23991.86	171.35	85.6	121	70	85	168	56	93	86		
5	1.1-3	n„o	1.52	1	39	3300	masculin		15209.31	167.55	82.4	151.5	72.5	91	183	51	112	125	0.593	0.6499
2	1.1-3	n„o	1.51	2		3150	masculin													
8	1 ou -	n„o	1.52	1	39	3370	feminino		37476.14	153.5	80.5	128	80	82	229	47	145	192		
12	1.1-3	n„o	1.55	3		3810	feminir	32	57888.71	164.95	113.9	118	67.5	95	213	41	132	185	0.5814	0.6208
5		n„o	1.51	2	40	2800	feminir	30												
4	1 ou -	15 + tod	1.67	2	43	3950	mascul	32	19261.85	188.45	90.3	147	72.5	94	168	56	98	91	0.5782	0.5781
5	1.1-3	n„o	1.49	2		2890	mascul	19												
5	1.1-3	1-14 tod	1.62	2	39	2650	feminir	27	31515.77	168.5	76.9	131.5	87.5	96	201	57	124	111	0.5776	0.5846
16	10	n„o	1.64	2	39	3100	feminir	72	17092.13	163.6	57.3	98	65.5	86	258	121	120	86	0.5727	0.5413
10	3.1-6	n„o	1.56	4	42	3860	feminir	25	21985.46	162.85	58	119	83.5	85	197	74	103	83	0.5782	0.5898
5	3.1-6	n„o	1.44	2		3220	feminir	29												
3	1 ou -	15 + par	1.51	3		1530	mascul	198												
8	1 ou -	n„o	1.61	2	40	3130	mascul	129												
5	1 ou -	1-14 par	1.54	1	40	3320	masculin		14481.02	170.35	77.7	112.5	68.5	89	175	49	90	185	0.5663	0.5592
12	3.1-6	n„o	1.55	3	39	3200	mascul	33	37316.67	172.8	104.7	133	76	95	191	54	120	95		
5	3.1-6	n„o	1.48	4	39	4400	mascul	61		167.35	124.1	153	103	180	159	51	91	99		
8	6.1-10	n„o	1.6	3	37	3300	mascul	32												
22	3.1-6	n„o	1.64	1	38	3270	feminino													
6	1 ou -	n„o	1.54	3	40	2400	feminir	45	17466.73	156.95	53.2	106.5	69	68	155	66	70	80	0.5764	0.5552
4	1.1-3	n„o	1.56	2	41	3950	feminir	73	23805.01	163.35	62.5	105	63	85	169	63	84	105		0.549
3	1.1-3	n„o	1.52	2	39	4050	mascul	108	32895.72	170.6	94.3	119	81.5	83	208	44	123	182	0.5821	0.6656
4	1.1-3	n„o	1.55	2	39	3000	feminir	100	22366.38	159.8	57.8	115	71	96	177	51	108	125	0.539	0.5739
9	1.1-3	n„o	1.5	2	42	2650	mascul	38	10400.72	157.6	53.7	132	74.5	85	159	49	91	90	0.5754	
6	1.1-3	1-14 tod	1.61	1		3740	masculin		36256.96	178.2	94.6	118.5	66	101	200	51	110	179	0.6575	0.5816
5	1.1-3	n„o	1.63	5	39	3450	mascul	24												
10	1.1-3	1-14 par	1.56	1	38	3120	masculin													
11	6.1-10	n„o	1.53	2	39	3130	mascul	34												
5	3.1-6	1-14 tod	1.56	4	37	3050	mascul	33												
5	1.1-3	n„o	1.57	5	36	2700	feminir	21												
5	3.1-6	n„o	1.5	5	36	3000	feminir	55	26037.9	155.6	63.3	103.5	67	83	165	47	103	68	0.5771	0.5665
16	6.1-10	n„o	1.57	2	39	3600	feminir	22	15676.1	159.6	49.8	105	81.5	64	150	51	82	76		
8	1.1-3	n„o	1.62	3	37	3250	feminir	14	23209.8	170.8	69.3	112.5	72	77	176	56	112	50	0.5768	0.5779
10	3.1-6	1-14 tod	1.67	1	40	2860	masculin		23470.74	174	67.1	118	71	102	260	68	143	214		
17	10	15 + tod	1.58	2	39	2520	feminir	26	21931.83	164	62	104.5	68	84	170	60	82	143	0.5773	0.5604
13	3.1-6	n„o	1.56	1	41	3620	masculin													
9	6.1-10	n„o	1.6	4		3350	feminir	71												
13	1.1-3	n„o	1.57	2	40	4470	mascul	46	10388.09	175.3	79.7	123	67	96	172	57	96	150	0.5799	0.5818

pescmae	prenda	pfumomae	paltmae	pgesta	pidgest	ppn	psex	pint	dmgtotdxa	daltura2012	dpeso	dsysmed	ddiamed	dglicose	dcolesterol	dhdl	dldl	dtrig	dECMICE	dECMICD
0	1 ou -	n„o		12	40	3610	feminir	18												
2	3.1-6	1-14 tod	1.59	2	42	3700	feminir	30		159.25		118.5	76.5						0.5794	0.578
12	1.1-3	n„o	1.59	1	40	4200	masculin													
16	3.1-6	n„o	1.63	3	42	3150	feminino													
5	1.1-3	1-14 tod	1.48	4	37	3160	mascul	43												
2	1.1-3	1-14 par	1.5	2	38	3150	mascul	23	9682.855	166.45	66.2	149.5	80.5	67	140	64	65	50	0.578	0.5781
5	1.1-3	1-14 par	1.55	1	41	4050	masculin		23154.44	168.2	83.6	137.5	88	81	168	56	73	240	0.5775	0.5853
4	1.1-3	n„o	1.56	2		3280	feminir	24												
5	1.1-3	1-14 par	1.5	2	39	3670	mascul	86	29954.61	178.6	97.1	120	78	109	173	38	81	290	0.6496	0.5794
0	1.1-3	n„o	1.64	7	38	2980	feminir	44	21849.31	167.5	65.3	119.5	79	87	190	69	103	68	0.5697	0.5422
2	3.1-6	n„o	1.49	2	39	3490	feminir	114	41550.99	153.95	91.5	114	77.5	80	165	57	89	77	0.5885	0.5959
5	1 ou -	n„o	1.47	1	40	4020	masculin		27275.82	183.15	85.9	128.5	81	88	212	54	140	97		0.5878
3	1 ou -	1-14 par	1.62	1	41	3600	masculin		27811.02	184.1	93.1	147	87.5	104	172	62	98	51	0.5785	0.5772
9	3.1-6	n„o	1.53	4	42	3100	feminir	40	24016.94	164.5	63.9	109.5	73	91	200	62	94	283	0.5778	0.5761
3	1.1-3	15 + tod	1.59	2	41	2900	feminino		26419.03	165.75	68.6	112.5	72.5	79	194	60	115	113	0.5782	0.579
8	1.1-3	n„o	1.5	5	40	3750	feminir	36	13333.56	154.8	57.4	108	65	76	167	43	109	93	0.5771	0.5775
0	1 ou -	n„o	1.53	5		2870	feminir	18	42281.97	160.2	85.3	112	74	85	151	48	89	88		0.5831
9	1 ou -	n„o	1.6	3	41	3250	feminir	38	39528.1	152.4	80.9	105	66	97	217	87	98	128	0.5781	0.577
3	1 ou -	15 + tod	1.58	3		1200	mascul	39												
16	6.1-10	n„o	1.61	1	42	3580	feminino		17848.27	162.45	54.5	115.5	72	88	218	76	121	105	0.5632	0.5777
8	3.1-6	n„o	1.58	1	41	3150	feminino		24199.4	159.3	66.6	113	70	86	178	54	112	52	0.5752	0.579
16	10	n„o	1.55	3	39	3600	mascul	90	45706.06	169.1	111.4	117.5	73	110	207	39	102	288	0.5799	0.6257
9	1.1-3	n„o	1.59	2	40	3800	mascul	30	37544.63	186.25	98.6	131	83.5	114	214	40	145	165	0.5893	0.5823
11	6.1-10	n„o	1.5	2	38	3100	mascul	29	29269.33	163.75	79.3	123	79	86	255	55	160	268	0.5933	0.5793
3	1 ou -	n„o	1.56	4		2050	mascul	56												
3	1 ou -	n„o	1.55	1		3270	feminino		24027.35	164.7	62.4	105.5	65.5	90	178	78	86	92	0.5677	0.5754
	1.1-3	1-14 tod	1.52	4	37	2950	feminir	17	29633.08	159.55	69.9	111.5	73.5	97	155	44	97	87	0.5764	0.5676
5	1.1-3	n„o	1.64	2	37	2930	feminino		36505.24	166.05	77.3	111.5	70	105	180	73	79	127	0.5789	0.5819
5	1.1-3	15 + tod	1.56	1	37	3090	feminino		17112.11	164.75	60.5	117.5	75	82	232	58	156	109	0.5787	0.5882
5	1.1-3	1-14 tod	1.63	3	40	3400	mascul	11	19220.6	171.8	77.1	130.5	76.5	93	168	62	89	88	0.5781	0.5783
7	1.1-3	1-14 tod	1.52	3		3450	feminir	13		161.35		112	65.5						0.5781	0.5781
9	1.1-3	n„o	1.64	2		3170	mascul	18												
5	1.1-3	n„o	1.67	3	39	3480	mascul	116		173.35	143.9	154	88	91	221	49	146	162		0.5711
6	1 ou -	1-14 par	1.53	1	38	3300	feminino		10220.8	154.05	51.6	120.5	78.5	68	161	64	76	101	0.612	0.5831
5	1.1-3	1-14 tod	1.6	4	38	2630	mascul	13	34410.8	175.6	102.3	160.5	103.5	95	233	68	148	127	0.5782	0.5824
6	3.1-6	n„o	1.53	2		2700	feminir	17	22673.3	169.55	65.3	103	71.5	71	188	75	92	89	0.5785	0.5849
15	3.1-6	1-14 par	1.5	2	43	2220	feminir	20												
5	1.1-3	1-14 tod	1.65	2	42	3670	mascul	52	29728.07	174.05	89.5	122.5	87	80	149	43	85	104	0.6504	0.6104
10	1.1-3	15 + par	1.56	1	36	3100	masculin		26245.72	186.95	96.8	133.5	80.5	91	154	35	89	144		0.5794
9	1.1-3	n„o	1.62	1	37	2870	feminino		18206.18	170.8	59.3	121.5	79	101	189	74	101	87	0.5597	
9	3.1-6	n„o	1.58	2		3360	mascul	56	22266.2	170.85	85.1	139	82	66	246	59	160	149	0.5708	0.5738
7	1.1-3	n„o	1.54	2	43	2770	mascul	19	23774.88	172.1	80.3	136	78	80	229	51	164	54	0.5794	0.5794
13	3.1-6	n„o	1.59	1	40	3600	feminino													
5	1.1-3	n„o	1.52	3		3740	mascul	71												
3	1.1-3	n„o	1.58	7	40	2870	feminir	75	17484.49	165.4	61.9	96	63.5	81	197	54	135	144	0.5724	0.5768
2	3.1-6	15 + tod	1.54	2	39	2610	feminino													
4	1.1-3	1-14 tod	1.58	2		2650	mascul	60												
8	1.1-3	n„o	1.51	1	37	2810	feminino		30938.34	160	69.4	105	77.5	104	193	82	95	61	0.5442	0.5368

pescmae	prenda	pfumomae	paltmae	pgesta	pidgest	ppn	psex	pint	dmgtotdxa	daltura2012	dpeso	dsysmed	ddiamed	dglicose	dcolesterol	dhdl	dldl	dtrig	dECMICE	dECMICD
9	3.1-6	n„o	1.61	2	42	3920	mascul	81												
16	3.1-6	1-14 par	1.62	1	41	3450	masculin													
10	1.1-3	1-14 tod	1.63	2	39	2950	mascul	14												
5	1.1-3	1-14 tod	1.48	2	40	3150	feminir	45												
3	1.1-3	n„o	1.53	6	42	3710	feminir	148	27928.76	150.95	64.3	105	79.5	89	188	63	85	169	0.5678	0.5674
8	10	n„o	1.68	1	39	3220	feminino													
4	1.1-3	1-14 par	1.59	1	43	3300	masculin		8478.179	172.4	59.6	136.5	89.5	71	169	61	88	123	0.5981	0.6353
5	1.1-3	1-14 tod	1.56	2		2920	feminir	52	17829.49	159.2	57.3	122	81	76	128	53	68	38	0.5639	0.5671
7	1.1-3	1-14 tod	1.66	3	40	3610	feminir	132	29694.21	163.6	72.3	108.5	65	79	190	53	124	67	0.5777	0.5773
8	1.1-3	n„o	1.55	1	40	3440	feminino													
4	1.1-3	15 + tod	1.51	1	38	2550	feminino		46757.53	157.3	97.9	120.5	88	140	305	87	200	181	0.59	0.57
5	1 ou -	n„o	1.52	1	35	2700	feminino													
5	1 ou -	15 + tod	1.61	1	40	2750	feminino		23937.51	164.5	68.6	119	84	65	181	48	111	91	0.6472	0.5763
8	1 ou -	1-14 tod	1.62	2		3340	feminino													
8	10	n„o	1.73	4	37	3150	feminir	12												
8	3.1-6	n„o	1.56	6		3700	mascul	47	12136.78	177.15	75.7	135	81	90	141	54	59	128	0.5648	0.5731
12	3.1-6	n„o	1.79	4	40	3770	mascul	36	15327.08	187.7	92.5	140	83.5	67	142	49	78	63	0.5793	0.5907
4	1 ou -	n„o	1.53	1	41	3400	masculin													
15	3.1-6	n„o	1.56	2	37	2940	feminir	22	14814.91	165.05	54.2	116.5	78.5	66	214	79	110	118	0.5759	0.5769
8	1.1-3	n„o	1.62	1	41	3580	feminino													
12	3.1-6	15 + par	1.61	2	40	3700	feminir	45												
8	1.1-3	n„o	1.54	1	42	3500	masculin		31978.09	172.2	102.1	133	86.5	84	194	47	126	154	0.6222	0.6123
7	1.1-3	1-14 tod	1.51	2	41	3130	feminir	24	27608.49	156.45	66	116	81.5	73	303	89	200	95	0.5781	0.561
5	1 ou -	n„o	1.57	2	41	4460	mascul	14		179.45	123	132	82	90	193	47	126	94	0.6475	0.6883
17	10	n„o	1.55	2	39	3050	feminir	49	14181.24	161.1	49.8	100	55.5	77	186	63	104	105	0.5545	0.5245
6	3.1-6	1-14 par	1.58	2		3400	feminir	20	24403.14	161.7	62.8	101	68.5	110	198	80	105	56	0.5653	0.5754
5	3.1-6	n„o	1.64	2	39	3500	mascul	90				134.5	96	85	128	44	65	68		
5	1 ou -	n„o	1.59	3	37	2420	feminir	25												
2	1.1-3	1-14 par	1.5	2	40	3200	mascul	50	16094.49	175.3	79.6	116.5	72.5	92	170	49	112	46	0.5824	0.5785
2	1 ou -	n„o	1.59	4	40	3420	mascul	26	21229.84	178.1	83.8	117.5	73	57	194	41	129	110	0.5781	0.5761
5	1 ou -	15 + tod	1.56	5	38	2930	mascul	42												
2	1 ou -	n„o	1.48	1		1780	feminino		17940.93	151.6	57.6	131.5	86	98	220	76	122	97		0.5782
9	3.1-6	n„o	1.56	3	39	2830	feminir	33	37068.92	160.05	79.8	123.5	83.5						0.5812	0.5783
16	3.1-6	n„o	1.63	1	38	3000	feminino		18597.68	165.8	56	110.5	70	82	150	78	54	107	0.5613	0.5783
3	1.1-3	n„o	1.57	2		3350	feminir	35												
4	1.1-3	n„o	1.69	2	36	2820	mascul	26												
5	1.1-3	n„o	1.51	2	39	3700	feminir	39												
5		n„o	1.5	6		2900	mascul	170												
8	1 ou -	n„o	1.37	1	38	2500	feminino													
10	3.1-6	1-14 tod	1.55	1	39	3050	feminino		34458.65	151.1	73.1	114.5	75	76	215	75	126	77	0.6023	0.5776
15	1.1-3	1-14 tod		1	41	3050	feminino													
12	6.1-10	n„o	1.56	5	39	3200	mascul	160	36293.83	178.75	96.4	125	84.5	166	169	57	100	94	0.5678	
18	6.1-10	n„o	1.7	1	40	3750	masculin		22539.36	183.8	89.2	125.5	75.5	72	135	48	73	72	0.5809	
5		n„o	1.49	4	39	3580	feminir	19												
14	3.1-6	n„o	1.55	1	40	3650	feminino		49173.82	155.6	91	116.5	77.5	93	195	61	122	64	0.5714	0.5787
4	1 ou -	1-14 tod	1.53	3		3500	mascul	20												
9	3.1-6	n„o	1.5	3	38	3450	mascul	31	19503.19	176.4	82.2	133.5	72.5	99	194	46	106	248	0.5899	0.5781
7	3.1-6	1-14 par	1.51	2	40	3050	mascul	16	6655.101	152.45	51.5	148	76.5	76	185	58	112	63	0.5756	0.5785

pescmae	prenda	pfumomae	paltmae	pgesta	pidgest	ppn	psex	pint	dmgtotdxa	daltura2012	dpeso	dsysmed	ddiamed	dglicose	dcolesterol	dhdl	dldl	dtrig	dECMICE	dECMICD
3	1.1-3	n„o	1.45	6	38	2940	mascul	19												
8	1.1-3	1-14 tod	1.56	2		3350	mascul	23	8882.511	168.55	57.3	128.5	69.5	101	173	76	77	65	0.5758	0.5655
4	1 ou -	n„o	1.62	1		3170	feminino		21267.3	161.85	61.9	107.5	65	78	218	57	136	160	0.553	0.57
6	1 ou -	n„o	1.59	1	38	3030	feminino													
14	1.1-3	n„o	1.54	1	40	3420	feminino													
6	1.1-3	1-14 tod	1.46	2	39	3100	mascul	14												
7	1.1-3	15 + tod	1.54	2	40	2570	mascul	38	16465.91	176	71.9	123.5	63.5	89	152	57	85	43	0.5851	0.5783
5	1.1-3	1-14 par	1.58	3	35	2120	mascul	18												
3	1.1-3	15 + tod	1.53	4	41	2060	mascul	57												
15	3.1-6	n„o	1.54	2	41	3580	feminir	12	33288.31	170.25	76.4	126	76	78	196	53	122	135	0.5738	0.5605
7	3.1-6	n„o	1.6	3	37	2100	feminir	58	49559.04	168.3	97.1	125	84	98	259	65	158	175		
5	1.1-3	15 + tod	1.6	1	39	3000	feminino													
5	1.1-3	15 + tod	1.54	3	40	3550	feminir	41												
8	1 ou -	1-14 tod	1.61	1	38	2100	feminino													
4	1.1-3	n„o	1.52	4	41	2800	mascul	15	6395.413	159.2	58.1	135	74	76	129	62	55	51	0.5821	0.5845
8	1 ou -	n„o	1.56	2	39	3030	mascul	42	26803.19	172.4	88.5	129.5	77.5	95	224	49	144	147	0.6182	0.5828
6	1.1-3	n„o	1.56	1		2950	masculin		20578.34	179.15	89.1	126.5	65.5	83	245	56	140	271	0.6243	0.5899
9	6.1-10	n„o	1.65	1	40	4350	masculin		27853.56	185.15	102.3	124	71	136	195	41	86	328	0.5761	0.5791
2	1.1-3	1-14 tod	1.51	8	37	2450	feminir	20												
8	1.1-3	n„o	1.61	3	40	3260	feminir	39	32160.76	155.4	78.6	119	80	96	177	38	108	178	0.58	0.6037
17	10	1-14 par	1.68	3		3600	mascul	12												
17	6.1-10	n„o	1.48	2	39	3750	feminir	23	29676.35	156.9	65.6	114.5	74	89	190	76	98	69	0.5755	
10	1 ou -	1-14 tod	1.52	1	39	3350	masculin		25916.81	170.65	96.1	131.5	80.5	101	281	61	184	203	0.5854	0.5857
10	1.1-3	1-14 tod	1.52	1		3820	masculin		22881.68	176.35	87.5	126	66	66	169	44	112	95	0.67	0.72
7	1.1-3	n„o	1.54	3		3470	feminir	51	13397.15	156.75	50.8	123	82.5	81	265	76	154	299	0.5778	0.5524
4	1.1-3	n„o	1.53	3	38	3900	feminir	117												
8	3.1-6	1-14 tod	1.51	2	38	2700	feminir	47	11603.35	156.3	48	117	72.5	61	133	42	79	49	0.5672	0.5659
6	1.1-3	n„o	1.62	1	42	3700	masculin			187.25	170.2	137	91.5	76	227	70	138	96		
0	1 ou -	n„o	1.62	7		3530	mascul	19	27805.61	178.9	95.8	132.5	87	99	143	28	94	87	0.579	0.5819
5	6.1-10	n„o	1.54	2		3150	feminir	12												
2	1 ou -	n„o	1.6	1	38	2600	masculin												0.6628	0.582
8	1.1-3	1-14 tod	1.57	2	40	3430	mascul	54	23343.85	165.25	85.1	136.5	82.5	103	172	44	106	114	0.5753	0.5789
5	1.1-3	n„o	1.64	4	40	3200	feminir	21	22586.68	160.25	58.9	99	69.5	86	240	87	126	179	0.5444	
10	1.1-3	n„o	1.65	1	39	2900	masculin		20038.6	180.9	79.4	126	70	73	219	44	135	286	0.5791	0.5799
0	1.1-3	15 + tod	1.51	2		3650	mascul	28	15100.34	168	73.4	133.5	76.5	86	119	46	62	40	0.5589	0.5782
11	1.1-3	1-14 tod	1.48	1	40	2850	masculin		16280.8	172.05	71.4	116.5	72.5	93	245	53	166	106	0.5766	0.5703
9	1.1-3	n„o	1.48	1	38	3600	feminino		19230.52	170.95	65.8	118	72	80	180	44	126	67		
10	3.1-6	n„o	1.61	1		3050	feminino		16584.73	182.7	60.8	109	74.5	75	161	72	78	49	0.5777	0.5612
8	1.1-3	n„o	1.52	2	40	3200	mascul	93	22874.96	171.7	72.8	127	82.5	91	172	49	98	129	0.5723	0.6433
8	3.1-6	15 + par	1.61	3	42	3650	feminir	15	47022.5	162.6	99.6	122.5	82	77	189	78	95	92	0.5738	
13	10	n„o	1.66	2	36	2680	masculin		6847.211	172.6	71.4	132.5	57.5	69	132	55	63	82	0.5776	0.578
4	1.1-3	n„o	1.63	1	40	4350	masculin		7273.845	182.1	66.8	110	64	63	159	63	83	54	0.6058	
6	3.1-6	1-14 tod	1.66	3	41	3180	feminir	55												
2	1 ou -	15 + tod	1.54	1	39	2130	masculin		22805.43	160.85	77.7	127	67.5	89	264	57	183	145	0.6649	0.581
9	3.1-6	n„o	1.52	1	40	3510	feminino			158.95		111.5	66							0.5842
9	1.1-3	n„o	1.55	1	38	2970	feminino		42798.2	154.3	89.5	153.5	107	96	162	55	89	113		
9	3.1-6	1-14 par	1.52	1	38	3400	masculin		28237.69	183.25	91.3	134	84.5	85	239	54	158	97		
6	1.1-3	n„o	1.46	3		2850	feminir	11	25932.43	159.1	64.7	145.5	88.5	83	174	52	98	129	0.5801	0.5753

pescmae	prenda	pfumomae	paltmae	pgesta	pidgest	ppn	psex	pint	dmgtotdxa	daltura2012	dpeso	dsysmed	ddiamed	dglicose	dcolesterol	dhdl	dldl	dtrig	dECMICE	dECMICD
16	10	1-14 tod	1.55	1	40	3300	feminino													
6	1.1-3	n„o	1.52	1	41	2920	feminino		15015.76	151.9	49	97.5	63.5	96	160	55	96	60	0.5788	0.5784
0	3.1-6	n„o	1.49	4	36	3150	feminir	51	15556.58	150.9	52.7	86	53	77	152	77	61	55	0.5834	0.577
5	1.1-3	n„o	1.6	1	42	3250	masculin													
12	3.1-6	n„o	1.65	1	39	2970	feminino													
11	3.1-6	n„o	1.63	2	41	4000	feminir	14	41606.59	176.8	89.9	135.5	89	89	270	74	160	175	0.5864	0.5943
8	1.1-3	n„o	1.51	2	39	3700	masculin		21037.67	178.1	83.5	144.5	71	133	184	63	83	141	0.5624	0.5788
9	1.1-3	n„o	1.56	4		4500	mascul	46												
5	1 ou -	n„o	1.58	3	39	3580	feminir	60												
3	3.1-6	15 + tod	1.54	6		3400	feminir	35	66274.64	157.2	117.7	112	73.5	94	155	50	73	187	0.584	0.5906
15	3.1-6	15 + tod	1.58	1	40	3150	masculin			177.15	120.1	144.5	71.5	103	176	35	122	112	0.6183	0.5982
12	10	15 + tod	1.6	1	37	3500	masculin		20817.58	172.5	79	111	68	81	218	80	121	75		
7	1 ou -	1-14 par	1.5	3	40	3410	mascul	27												
5	1.1-3	n„o		4		4120	mascul	34	18697.33	171.15	72.8	122	72.5	103	201	70	120	68		
8	3.1-6	n„o	1.48	2	39	3930	feminir	63		170.15	75.8	108	64							
14	1.1-3	1-14 par	1.76	1	39	3000	masculin			196.1	100.9	130.5	74.5	73	189	38	108	223	0.5797	0.5825
10	3.1-6	n„o	1.6	2	40	4030	mascul	27	31817.24	189.35	102.8	144.5	75	117	249	39	157	283	0.5794	0.5784
3	1.1-3	15 + tod	1.55	5	39	3420	mascul	12	5034.431	164.2	63.8	99.5	61.5	65	185	54	109	154	0.5783	0.5785
9	3.1-6	n„o	1.53	2	40	3640	feminir	27	29838.6	165.3	70.6	105.5	67.5	66	173	57	101	86	0.5898	0.5896
5	10	n„o	1.61	3		3640	mascul	23												
0	1 ou -	1-14 par	1.55	3		3670	mascul	18												
3	1 ou -	n„o	1.47	6	40	3810	mascul	55												
5	3.1-6	n„o	1.58	6	36	3390	mascul	106	26490.43	173.1	80.3	130	82.5	78	186	47	115	131		
7	1 ou -	n„o	1.5	1	38	2430	feminino		21394.66	154.5	61.5	125.5	77	139	253	43	91	382	0.5649	0.5761
0	3.1-6	15 + tod	1.59	6	39	3550	mascul	20												
6	1.1-3	1-14 tod	1.61	2	42	2660	mascul	17	16525.98	169.5	75.3	101.5	60.5	75	190	57	122	66	0.5682	0.5781
0	1.1-3	n„o	1.55	6		3000	mascul	30	20375.58	168.05	76.4	114	71	74	224	51	161	65		
7	3.1-6	n„o	1.51	3	42	4400	mascul	55	19898.16	180.05	90	124.5	77	125	271	59	139	263	0.5798	0.5882
7	1.1-3	15 + tod	1.53	2		3800	mascul	17												
13	6.1-10	n„o	1.62	3	38	3570	mascul	72	6948.965	174.7	65.1	122	64.5	83	238	75	148	111	0.5771	0.5782
6	1.1-3	n„o	1.58	3	38	3150	feminir	19	34317.65	159.45	79.6	142	93.5	81	172	52	106	87	0.5806	0.5798
5	1.1-3	n„o	1.44	2	37	3100	feminir	28	21462.23	152.85	58.9	88	52.5	77	186	55	117	68	0.5776	0.5786
4	1.1-3	n„o	1.6	1	41	3980	masculin		28829.29	175.6	96.6	114.5	65.5	72	168	46	100	105	0.5865	0.5756
5	1 ou -	1-14 tod	1.5	9		2640	feminir	11	46191.4	160.05	97.4	129.5	81	118	168	54	101	148	0.576	0.5831
10	1.1-3	n„o	1.46	1	41	2700	feminino		16860.36	154.95	56.2	152.5	105.5	83	270	91	164	92	0.5787	0.5841
5	1 ou -	n„o	1.56	4		3780	mascul	29	18282.45	176.7	81.3	129.5	77.5	78	122	42	73	52		
5	1.1-3	n„o	1.5	2		1480	feminino		21981.67	155.7	57.6	108.5	70	99	188	60	106	126	0.5682	0.576
17	3.1-6	n„o	1.62	2	41	3650	feminir	51	36991.55	157.7	76.4	105	73.5	88	167	58	93	69	0.5791	
5	1.1-3	n„o	1.52	4		2360	feminir	11												
0	3.1-6	1-14 tod	1.49	5	38	3000	feminir	81	21725.46	162.2	67.1	107	65.5	74	160	57	86	59	0.5771	0.5803
7	1.1-3	1-14 par	1.54	1	41	3000	masculin		8194.133	175.3	74.7	137.5	78.5	76	225	58	139	145	0.634	0.585
2	1.1-3	n„o	1.56	4	40	3950	feminir	43				84.5	59.5							
12	3.1-6	n„o	1.51	2	38	3280	mascul	24												
12	3.1-6	1-14 tod	1.54	2	40	3510	feminir	37	28548.99	167.8	70.1	108	57.5	69	234	52	159	120	0.5756	0.5752
17	1.1-3	n„o	1.56	1	40	3200	feminino		21140.9	163.1	53.4	107.5	75.5	88	157	78	55	112		0.5716
5	3.1-6	n„o	1.57	8	38	3400	feminir	25	19306.15	161.65	55.5	101	65	80	160	37	111	75	0.5772	0.5464
3	1.1-3	1-14 tod	1.58	1	40	3150	feminino													
7	1.1-3	1-14 par	1.6	2	40	3270	feminir	37	21145.02	158.7	57	113.5	72	76	150	52	81	58	0.5784	0.5783

pescmae	prenda	pfumomae	paltmae	pgesta	pidgest	ppn	psex	pint	dmgtotdxa	daltura2012	dpeso	dsysmed	ddiamed	dglicose	dcolesterol	dhdl	dldl	dtrig	dECMICE	dECMICD
5	1.1-3	n„o	1.56	4		3360	mascul	45												
5	1.1-3	n„o	1.56	2		2950	feminir	11	21234.6	157.35	54.9	126	85.5						0.5786	0.5783
4	1 ou -	1-14 par	1.62	7	39	3280	feminir	14												
8	1.1-3	n„o	1.57	1	38	3100	feminino		17493.23	158.3	51.9	100.5	64	58	200	75	110	89	0.5829	0.5775
5	3.1-6	n„o	1.56	1	37	3160	masculin		9382.245	170.45	64.6	117	65.5	78	183	65	110	35	0.578	0.5779
10	3.1-6	n„o	1.5	3	37	3050	mascul	34	13576.69	169.55	73.2	125	83.5	66	209	75	115	90	0.5659	0.578
2	1 ou -	n„o	1.46	11	40	3000	mascul	42	13055.15	158.3	69.9	117.5	68.5	82	136	40	75	99	0.5683	0.5732
6	3.1-6	1-14 par	1.54	2	42	3200	feminir	59												
5	1.1-3	n„o	1.59	4	36	4300	feminir	33	24081.97	161.8	63.7	98.5	64	89	225	88	121	86	0.5764	0.5768
6	1.1-3	n„o	1.51	3	37	2450	mascul	151	4327.687	165.4	57.6	116.5	64	88	138	48	80	62	0.5935	0.5781
17	6.1-10	1-14 tod	1.56	2	40	3860	mascul	17												
8	6.1-10	1-14 par	1.58	1		2990	feminino		33474.46	163.9	78.1	111.5	69	65	141	50	64	132	0.5789	0.5799
5	3.1-6	n„o	1.72	4	39	3050	feminir	16	18080.72	161	55.4	102.5	62	79	208	78	117	88		
3	1 ou -	15 + tod	1.56	5		3550	feminir	25												
5	1.1-3	n„o	1.48	3	39	3400	feminir	72	29734.21	167.85	73.5	119.5	78.5	81	254	75	158	125	0.5854	0.5763
11	1.1-3	n„o	1.45	2	40	3700	mascul	41	23487.8	171.7	91.4	120.5	62.5	86	209	56	135	114	0.59	0.57
8	1.1-3	n„o	1.56	6	37	3410	mascul	19		193.35	83.5	127	69	85	168	49	107	79	0.5764	0.5781
2	1.1-3	n„o	1.5	9	42	3570	mascul	33	26101.98	175.65	82.4	124.5	76.5	90	178	44	103	187	0.5769	0.5575
5	1.1-3	n„o	1.43	4	40	3000	feminir	13												
13	6.1-10	n„o	1.47	2	38	2800	feminir	51	24431.14	150	59.7	137	99	76	191	51	122	134	0.578	0.573
10	6.1-10	1-14 par	1.56	1	39	3750	feminino													
15	6.1-10	n„o	1.62	2	39	3850	feminir	65	41505.4	166.9	90	136.5	85.5	90	142	48	78	115	0.5766	0.6027
4	1 ou -	15 + tod	1.61	2	42	2800	feminir	33	8832.814	164.8	54.5	113	61.5	75	158	60	84	41	0.5735	0.5705
10	3.1-6	n„o	1.51	1		2000	feminino		23420.78	158.1	59.5	126.5	77	86	200	76	84	197		0.5811
5	1 ou -	n„o	1.57	1	36	2800	masculin		32290.12	180	98.8	127.5	84.5	92	235	47	163	111		0.5751
5	1.1-3	1-14 tod	1.55	3	39	2650	feminir	128	24538.96	172	63.7	115	62.5	78	143	42	83	96		0.589
12	10	n„o	1.59	3	40	3700	feminir	11												
12	3.1-6	n„o	1.6	4	41	3430	mascul	21	19809.07	182.75	90.8	124	77.5	96	184	70	101	86	0.5784	0.5821
3	1.1-3	n„o	1.51	2	38	3050	mascul	41	15825.04	162.6	68.1	109.5	63.5	85	192	70	101	93	0.5779	0.577
0	1.1-3	n„o	1.45	3	41	3350	mascul	18												
9	1.1-3	1-14 tod	1.53	2		3400	masculin			171.15	128.4	133	90.5	89	190	43	117	135	0.6031	
1	1.1-3	n„o	1.53	3	38	2850	feminir	99		159.35	132.4	123.5	81.5	75	205	54	130	96		
6	6.1-10	n„o	1.62	3	39	3570	feminir	15												
5	1.1-3	n„o	1.61	2	40	2950	feminir	30	33753.15	155.8	67.9	107.5	71	136	244	75	151	120	0.5827	0.5686
10	3.1-6	1-14 tod	1.62	2	43	2950	feminir	30	36425.04	169.15	81.6	132.5	86.5	113	306	79	200	148	0.578	0.578
7	1 ou -	n„o	1.57	1		3900	feminino		28024.95	166.1	69.5	114	79.5	99	212	64	128	118	0.5781	0.5782
9	3.1-6	15 + tod	1.47	6	37	2270	mascul	26												
6	1 ou -	n„o	1.57	3	39	3250	mascul	57	18270.04	157.7	75.4	135.5	80	93	320	65	182	400	0.5628	0.5842
4	1.1-3	n„o	1.52	2	41	3850	feminir	40	31627.76	159.7	71.7	96.5	60	117	198	64	103	167	0.5782	0.5768
7	1.1-3	n„o	1.65	4	42	4370	mascul	43		192.4	160.9	136.5	77.5	99	191	39	119	201	0.5866	0.6362
8	6.1-10	1-14 tod	1.71	2	39	4750	feminino													
3	3.1-6	n„o	1.59	2	39	2780	masculin		15817.67	164.95	66.9	115.5	75	97	269	80	177	58	0.5823	0.6063
8	10	n„o	1.57	7	37	3430	feminir	33	14279.39	168.1	55.2	117.5	70.5	60	147	60	74	55	0.5774	0.5792
11	1.1-3	n„o	1.49	1	35	2400	masculin													
8	1.1-3	n„o	1.59	3	43	3700	mascul	17	12790.87	168.5	69.1	119.5	64	90	186	50	119	76	0.5778	0.5787
9	1.1-3	n„o	1.54	3	42	3950	feminir	26	11534.56	166.05	56.9	102.5	61.5	97	190	43	125	120	0.5793	0.5892
18	6.1-10	n„o		1	34	2400	feminino													
9	1.1-3	n„o	1.64	6	37	4000	mascul	25												

pescmae	prenda	pfumomae	paltmae	pgesta	pidgest	ppn	psex	pint	dmgtotdxa	daltura2012	dpeso	dsysmed	ddiamed	dglicose	dcolesterol	dhdl	dldl	dtrig	dECMICE	dECMICD
3	1.1-3	n,,o	1.53	2	41	3400	feminir	178	22484.69	154.15	58.2	115.5	75.5	69	156	45	102	53	0.5806	0.5898
18	10	n,,o	1.65	1	41	3350	feminino													
12	3.1-6	n,,o	1.52	1	42	3000	feminino													
5	1.1-3	n,,o	1.65	2	41	3500	mascul	55	24262.77	175.45	82.4	154.5	100.5	109	166	34	76	297	0.5806	0.578
15	6.1-10	n,,o	1.67	1	36	3200	feminino													
8	6.1-10	n,,o	1.6	1	40	3510	feminino													
16	6.1-10	n,,o	1.57	2	40	3300	mascul	98												
4	3.1-6	1-14 par	1.51	1		2480	feminino		8514.988	160.3	44.9	113.5	80	135	211	69	109	234	0.5767	0.5734
4	3.1-6	15 + tod	1.56	4		3050	feminir	12												
0	1.1-3	n,,o	1.59	4	41	3660	feminir	17	36741.57	155.55	79.1	96	64	71	250	97	137	85	0.5833	0.5787
5	3.1-6	n,,o	1.69	1	38	3100	feminino													
6	1.1-3	n,,o	1.57	1	39	3210	masculin													0.5616
10	3.1-6	n,,o	1.62	1	40	1830	feminino													
6	1.1-3	n,,o	1.53	4	37	3130	mascul	25	14168.19	169.15	74.9	116	59.5	98	155	38	87	188	0.5795	0.5803
5	1 ou -	n,,o	1.49	2	40	3570	mascul	21	11184.46	162.25	62.4	107	73.5	112	215	62	119	229	0.5775	
4	1 ou -	1-14 par	1.53	2		3330	feminir	57	47326.3	157.8	94.3	114.5	74.5	76	203	83	113	58	0.605	0.5894
5	1.1-3	1-14 tod	1.56	2	40	3480	feminir	121	34429.46	161.7	75.2	110	70.5	89	197	75	110	59		
8	1.1-3	n,,o	1.47	3	39	3080	masculin		15214.97	159.5	63.3	146.5	87.5	107	267	64	191	67	0.6112	0.5447
4	1 ou -	n,,o	1.54	2	34	1960	mascul	18	7293.448	174.15	63.5	137	79	78	200	97	79	75	0.581	0.5784
12	3.1-6	1-14 tod	1.56	1	41	3510	feminino		39293.43	164.6	81.3	129	95.5	112	200	46	126	145	0.6079	0.5814
11	10	n,,o	1.68	1	38	3380	masculin		19291.2	178.75	81	112	71.5	74	142	58	68	83	0.5673	0.5795
5	1.1-3	n,,o	1.61	1	40	3670	feminino		35510.93	161.65	76	111.5	71	85	180	59	102	105	0.5712	0.5707
5	1.1-3	1-14 tod	1.57	1	40	3700	feminino													
4	1.1-3	1-14 tod	1.4	2	38	2770	mascul	57	25434.45	166.85	83.1	135	70.5	90	199	40	124	164	0.5967	
3	1.1-3	n,,o	1.62	2		3100	feminir	66	26318.49	159.05	66.3	126.5	85	102	208	70	118	128	0.5766	0.5779
4	1.1-3	1-14 tod	1.49	1	39	2650	masculin													
7	3.1-6	n,,o	1.5	1	42	3050	masculin		5528.515	162.45	57.9	149.5	92	79	174	62	95	97	0.5766	0.5677
3	1.1-3	n,,o	1.7	1	39	3650	feminino		37508.6	164.85	91.6	127.5	83	77	172	51	98	130	0.6664	0.5788
5	1.1-3	n,,o	1.52	2	37	2700	feminir	33	19821.18	163.35	60.7	99	56	79	223	108	92	69	0.5716	0.5569
4	1.1-3	n,,o	1.6	2	39	3350	mascul	57												
1	1.1-3	1-14 par	1.58	4	41	3730	mascul	82	10023.88	170.05	68	149.5	99	99	194	101	71	72	0.6213	0.5847
9	1.1-3	n,,o	1.58	1	38	2910	masculin													
16	6.1-10	15 + tod	1.55	5	40	3500	mascul	38	24998.99	177.4	82.2	136	93	113	167	69	82	90	0.5716	0.5686
5	1.1-3	n,,o	1.59	3		3880	mascul	54												
4	1 ou -	15 + tod	1.45	8	40	3110	feminir	22												
7	3.1-6	15 + tod	1.65	3	42	3200	feminir	58	21959.39	165.4	62.9	102.5	70	87	240	82	145	64	0.575	0.582
9	6.1-10	15 + tod	1.54	7	37	2500	feminir	14	19039.13	168	63.8	118	72.5	80	148	67	57	100	0.5781	0.5759
5	1.1-3	n,,o	1.56	6	42	3950	feminir	24												
7	1.1-3	n,,o	1.54	2	39	3250	feminir	38												
5	1.1-3	15 + tod	1.63	10	41	3430	mascul	29	18333.02	176.9	79.4	126	72	95	170	63	92	87	0.5456	0.5797
11	10	n,,o	1.56	1	39	4350	masculin		31522.17	179.25	92	117.5	75.5	95	157	39	65	287	0.5741	0.5767
8	3.1-6	n,,o	1.54	1		3800	masculin		22441.82	170.6	83.2	138	74	72	186	47	117	131	0.5784	0.5728
5	1.1-3	1-14 tod	1.54	4	40	3070	mascul	37	4901.433	172.65	56.8	144	85	64	171	64	92	98	0.5737	0.578
11	1.1-3	1-14 tod	1.63	1	41	2750	feminino		47196.21	156.35	97.8	123	86.5	79	226	67	135	178	0.5822	0.5795
8	1.1-3	1-14 par	1.57	1	38	3000	feminino		38790.51	164.5	84.3	114.5	75.5	85	140	44	83	45		
7	3.1-6	n,,o	1.57	3	39	3770	feminir	23	10684.23	161.7	50.4	103	59	82	179	79	90	30	0.5704	0.5787
6	1.1-3	1-14 par	1.57	1	39	3180	feminino													
7	1.1-3	n,,o	1.56	3	31	1530	feminir	32												

pescmae	prenda	pfumomae	paltmae	pgesta	pidgest	ppn	psex	pint	dmgtotdxa	daltura2012	dpeso	dsysmed	ddiamed	dglicose	dcolesterol	dhdl	dldl	dtrig	dECMICE	dECMICD
5	1.1-3	n„o	1.56	2	41	2930	feminir	67												
5	1.1-3	1-14 par	1.56	2		2950	feminir	37												
16	3.1-6	1-14 par	1.61	5	40	3780	feminir	14	46509.47	166.25	87.8	104.5	70.5	88	223	70	119	180	0.5781	
16	10	1-14 tod	1.6	5		3550	mascul	52	26691.52	182.2	87.7	125.5	83	91	178	46	122	60	0.5799	0.5891
9	3.1-6	n„o	1.53	3		3350	mascul	20	5074.288	180.75	69.3	125	71	85	177	88	66	103		
18	10	1-14 par		1	41	3900	feminino													
3	1.1-3	1-14 tod	1.51	2	40	2600	mascul	10												
9	1.1-3	1-14 tod	1.66	1	39	3650	masculin		11667.61	177.45	65.3	120	71.5	82	135	62	63	47	0.5765	0.5768
9	3.1-6	n„o	1.54	1	42	3700	feminino			159.25	74.1	110	76	80	214	58	129	166	0.5774	0.5701
6	1.1-3	n„o	1.65	2		3500	mascul	56	26231.18	172.2	84.1	130.5	83.5	83	213	39	125	205	0.5793	0.585
0	1.1-3	n„o	1.68	1	43	3650	masculin		37398.34	175.65	98.7	140.5	96	97	212	49	144	81	0.5853	
5	1.1-3	n„o	1.66	1	37	3600	feminino		42893.16	172.8	102.7	143	82.5	86	220	55	134	162	0.6216	0.6593
1	3.1-6	n„o	1.57	1	40	3150	masculin													
0	1 ou -	n„o	1.52	1	39	3600	masculin		14636.09	173.55	69.7	107	64.5	71	217	55	137	99	0.5615	0.5812
7	3.1-6	n„o	1.68	1		1600	feminino													
4	1 ou -	1-14 tod	1.61	1	37	3870	feminino		18214.83	166.4	59.2	97	63.5	90	160	52	93	63	0.5727	0.5783
5	3.1-6	n„o	1.62	1	38	3300	feminino		19869.83	165.5	58.4	129	89	84	184	71	87	114	0.5798	0.5628
4	1.1-3	n„o	1.48	1	36	3000	masculin		16767.99	170.15	74.8	121	62.5	81	218	56	148	60		0.561
4	1 ou -	1-14 tod	1.5	3	39	2860	feminir	24												
5	1.1-3	n„o	1.56	1	40	3400	masculin													
6	1.1-3	n„o	1.48	2	39	3000	feminir	60												
6	1.1-3	n„o	1.55	3	38	3700	mascul	94												
7	1.1-3	1-14 par	1.62	1	37	2680	feminino		27878.71	160.2	71.1	130	93	86	168	53	100	91	0.583	0.573
9	3.1-6	n„o	1.7	2	38	3750	feminir	39	45754.67	177.55	92.5	119	77	85	190	53	108	181	0.5725	0.5799
4	3.1-6	n„o	1.56	1	40	3500	feminino		22734.24	163.55	62.4	109	71	88	220	104	93	92	0.5699	0.5687
9	1.1-3	15 + tod	1.59	2		3750	mascul	55	5632.565	183.75	79.1	138.5	69	86	159	51	86	94		
3	3.1-6	n„o	1.53	2	40	3510	mascul	43	11988.02	163.1	65.1	110	62.5	88	185	48	120	77		
7	3.1-6	1-14 tod	1.52	2	39	3750	mascul	26												
5	1.1-3	n„o	1.54	1	40	3670	masculin													
7	3.1-6	n„o	1.57	4	38	3450	feminir	151	22733.34	170.95	68.3	110.5	59.5	72	190	74	95	96	0.5785	0.6066
9	3.1-6	n„o	1.56	1	41	3500	masculin													
8	6.1-10	15 + tod	1.62	3	33	1900	mascul	36												
17	10	1-14 tod	1.58	1	39	3010	feminino		13865.05	159	48	103	70.5	111	149	67	65	82		
2	1 ou -	n„o	1.48	2	40	3550	mascul	21	11297.56	169.8	62.4	120.5	70	97	160	40	87	168	0.5853	0.568
5	1 ou -	1-14 par	1.54	2	42	3360	feminino		40772.84	155	81.5	110	73	75	181	43	106	155	0.5698	0.5679
7	1.1-3	n„o	1.63	1	39	3870	masculin		19677.04	185.6	82.1	135	73.5	47	94	34	50	47	0.5708	0.5782
8	1.1-3	n„o	1.42	2	40	3710	feminir	70												
7	6.1-10	n„o	1.58	3	36	2800	mascul	29												
6	3.1-6	n„o	1.55	2	39	3540	mascul	16	30469.04	175	101.6	130.5	76.5	89	152	40	93	114		
16	10	n„o	1.65	3		3200	mascul	103												
8	3.1-6	n„o	1.64	1	42	3320	feminino		41666.36	176.25	101.8	112	68	74	245	52	159	227	0.591	0.5882
6	3.1-6	n„o	1.57	4	34	2400	mascul	22	6863.33	175.95	66.1	104.5	62	75	190	60	97	258	0.5781	0.5782
12	6.1-10	1-14 tod	1.65	1	39	3500	masculin													
6	1.1-3	1-14 tod	1.56	1	39	3400	masculin		11815.46	166.45	61.2	116.5	73	80	140	65	60	52	0.5791	0.578
8	1.1-3	n„o	1.55	1	37	2550	masculin													
12	1.1-3	1-14 tod	1.51	2		3280	mascul	40	9845.624	173.5	63.7	122.5	65.5	81	168	52	100	62	0.5834	0.5809
5	3.1-6	15 + tod	1.56	5	39	3480	mascul	24	35312.02	173.9	99.9	143.5	73.5	83	195	65	120	40	0.5772	0.5886
8	1.1-3	1-14 tod	1.52	3		2680	feminir	11	16358.43	149.6	48.6	99	66.5	64	176	69	96	76		

pescmae	prenda	pfumomae	paltmae	pgesta	pidgest	ppn	psex	pint	dmgtotdxa	daltura2012	dpeso	dsysmed	ddiamed	dglicose	dcolesterol	dhdl	dldl	dtrig	dECMICE	dECMICD
3	1.1-3	n„o	1.62	2	42	3230	feminir	36	20508.21	165.9	68.2	105	61.5	81	190	50	126	80	0.5762	0.5789
5	3.1-6	n„o	1.62	1		2700	feminino													
5	3.1-6	15 + tod	1.54	2	39	3200	feminir	69	38900.66	152.2	77.1	103.5	70.5	89	165	61	90	105		
14	1.1-3	n„o	1.56	1	41	3640	masculin		19688.9	180.25	79.6	119	65	60	175	62	94	90	0.6181	0.5805
0	1 ou -	1-14 par	1.54	11		3000	feminir	22												
4	3.1-6	n„o	1.54	2	40	2850	feminir	131												
5	1.1-3	n„o	1.6	1		2450	feminino													
4	1.1-3	n„o	1.47	1	39	3250	masculin		12641.18	169.3	64	121	74	80	225	75	127	135	0.5803	0.5784
12	3.1-6	1-14 tod	1.5	1	40	3170	masculin		39421.77	175.1	110.6	132.5	82.5	72	247	72	120	321		
17	3.1-6	n„o	1.53	2	41	3330	feminir	34	16679.04	165.35	58.6	113.5	69.5	68	195	88	90	74	0.5788	0.5734
2	1 ou -	n„o	1.56	2	40	3310	mascul	47	22017.65	173.6	79.5	120.5	82	70	176	46	107	85		
9	1.1-3	n„o	1.63	7	38	3600	mascul	32	18381.17	171.5	82.3	133	69	105	242	42	112	430	0.5804	0.5771
0	1.1-3	1-14 tod	1.46	3	37	3000	feminir	32	36433.57	155.8	81.7	111.5	81	75	133	53	65	69	0.574	0.5739
2	1 ou -	n„o	1.56	1	38	2800	masculin		7935.463	171.2	53.5	130.5	82.5	68	210	60	130	105	0.579	0.5638
9	1.1-3	1-14 tod	1.54	5	41	2700	mascul	13	9235.178	176.6	68.3	117	59	79	127	49	67	52		
10	6.1-10	n„o	1.51	1	39	2880	feminino		55270.69	155.6	107.5	127	92	104	226	62	129	198		
16	10	1-14 tod	1.5	2	38	3100	feminir	43												
5	1 ou -	15 + tod	1.52	7		3100	feminir	30												
1	1 ou -	1-14 par	1.51	3		2750	mascul	97												
5	1.1-3	1-14 tod	1.57	2	39	3270	feminir	94	39594.33	172.65	99.5	113.5	73.5	86	215	55	144	117	0.638	0.5783
14	3.1-6	n„o	1.55	1	41	2850	feminino		33033.41	159.35	77.1	114	79.5	78	191	70	100	127	0.5779	0.5752
2	1.1-3	1-14 tod	1.58	2	36	2750	feminir	30	26152.79	162.25	73.4	104	69	86	174	70	84	79	0.5777	0.5784
5	3.1-6	n„o	1.55	1	41	4070	masculin		19667.09	173.65	80.5	136.5	74	112	213	60	138	51		
5	1.1-3	n„o	1.59	4	39	3430	mascul	211	15488.21	171	69.5	121.5	73	47	111	29	70	45	0.5879	0.5744
4	1 ou -	1-14 tod	1.54	1		3750	feminino													
15	1.1-3	1-14 tod	1.47	2	39	3080	mascul	85												
11	3.1-6	1-14 tod	1.52	2	40	2530	feminir	80												
11	3.1-6	n„o	1.66	1	36	2760	feminino													
3	1 ou -	n„o	1.57	7	41	3200	mascul	117	4675.508	174.15	68.2	128	82.5	70	188	68	110	55	0.577	0.5787
8	1.1-3	n„o	1.47	2	40	2370	feminir	87												
6	1.1-3	1-14 tod	1.57	1	39	2310	masculin		18900.28	174.85	74.8	125	81.5	84	185	62	108	71	0.5781	
7	1.1-3	n„o	1.55	3	38	2800	feminir	24	38214.8	156.05	78.6	108	61.5	82	142	49	85	57	0.5806	0.5782
4	1 ou -	n„o	1.5	2	36	2330	mascul	19	15562.64	168.55	65.4	127	69.5	67	185	63	104	71	0.5805	0.5782
8	3.1-6	1-14 tod	1.56	2	39	3680	mascul	72	9312.748	184.15	70.1	125	64.5	92	179	68	100	57	0.5777	0.5699
8	1 ou -	n„o	1.7	2	40	3140	feminir	26												
5	1.1-3	1-14 tod	1.46	1	40	3350	feminino													
4	1 ou -	n„o	1.54	2	41	2650	mascul	37	14835.43	168.8	70.8	126	78.5	74	127	62	52	45	0.5751	0.5784
7	3.1-6	15 + tod	1.55	2	39	3490	mascul	68	35394.33	168.3	99.1	123.5	69.5	79	198	62	120	64	0.5833	0.5981
9	1.1-3	n„o	1.6	1	40	3700	masculin		14870.48	177.9	81.1	131	62.5	93	166	78	70	72	0.578	0.5797
5	1.1-3	n„o	1.6	3	40	3210	mascul	29	30698.01	179.7	95.4	137.5	86							0.5859
5	1.1-3	1-14 par	1.55	1	39	3750	masculin		17905.27	173.45	78.9	141.5	91.5	69	189	48	126	53	0.589	0.6245
16	6.1-10	n„o	1.59	2	42	2400	mascul	25												
4	1.1-3	n„o	1.69	2	36	3000	mascul	26	31172.08	188.2	101.2	136	68.5	89	237	43	151	221	0.567	0.5796
5	1.1-3	n„o	1.5	5	39	3760	mascul	90												
5	1 ou -	n„o	1.46	1	40	3320	feminino													
8	1.1-3	1-14 par	1.58	1	41	2950	masculin													
12	10	n„o	1.54	3	38	3920	mascul	71												
7	1.1-3	1-14 par	1.63	1	39	3650	masculin													

pescmae	prenda	pfumomae	paltmae	pgesta	pidgest	ppn	psex	pint	dmgtotdxa	daltura2012	dpeso	dsysmed	ddiamed	dglicose	dcolesterol	dhdl	dldl	dtrig	dECMICE	dECMICD
9	1.1-3	n„o	1.55	1		3430	feminino		41013.26	164.9	87.6	118	83	75	147	49	83	76		0.577
5	1 ou -	1-14 tod	1.59	1	41	3200	feminino			154.8		104.5	65						0.5782	0.5778
10	1.1-3	n„o	1.61	1	40	3300	masculin													
5	1.1-3	n„o	1.65	2	39	3950	mascul	35		181.8	123.8	135.5	77.5	94	235	48	160	118		
3	1.1-3	n„o	1.58	1	41	3340	masculin		21086.33	168.9	75.3	119	73.5	82	192	64	106	113	0.5786	0.6324
5	1 ou -	n„o	1.63	2		2190	mascul	22	27313.82	175.45	90.6	123	85.5	81	185	34	123	157	0.5822	0.5751
16	10	n„o	1.6	1	41	3300	feminino													
5	1 ou -	n„o	1.53	1	39	2900	masculin		8934.127	174.9	66.2	150.5	94	72	145	36	81	156		0.579
5	3.1-6	n„o	1.52	4	40	2630	feminir	18	25023.74	155	62.4	98	63	77	170	65	92	48	0.585	0.5781
15	3.1-6	n„o	1.55	1	40	3300	masculin													
5	1.1-3	1-14 tod	1.55	2	41	4150	mascul	63												
0	1 ou -	n„o	1.51	7		3880	mascul	18												
5	1 ou -	n„o	1.52	1	40	2550	masculin		19339.28	170	80	134	92.5	89	260	61	151	244	0.5874	0.589
5	1.1-3	n„o	1.53	4	36	2680	feminir	33		155.65		121	85.5	91	236	69	135	158		0.5795
0	1.1-3	n„o	1.59	4	35	2300	feminir	65		163	83.6	115.5	83.5	93	185	66	100	111		
3	1 ou -	n„o	1.5	7		3660	mascul	34												
4	1.1-3	1-14 tod	1.55	4		2730	feminir	20	14040.27	161.3	56.2	109.5	64.5	114	167	52	87	116	0.5566	0.578
6	1.1-3	1-14 par	1.46	1	39	2600	feminino													
6	3.1-6	n„o	1.62	4		3460	feminir	57												
12	1.1-3	n„o	1.68	3		3470	mascul	24												
3	3.1-6	n„o	1.59	4	39	3650	mascul	30	18612.17	169.5	77.2	131.5	78.5	90	159	41	100	71	0.5786	0.5798
4	1 ou -	n„o	1.55	1		1350	masculin													
8	1.1-3	n„o	1.55	1	37	3500	feminino		35413.09	157.7	75	128.5	76.5	86	181	61	113	67	0.5652	0.5767
2	1.1-3	1-14 tod	1.55	3	38	3360	mascul	148	35049.42	165.9	92.4	139.5	83.5	82	250	62	154	239	0.5947	0.6251
8	3.1-6	1-14 tod	1.6	2	40	3550	mascul	43	12242.15	171.6	61.4	116	69.5	71	175	56	95	117	0.5926	0.5902
5	1.1-3	n„o	1.55	1	39	3060	feminino		25437.48	154.2	62.7	107.5	73	96	189	67	108	65	0.5775	0.5729
8	1.1-3	n„o	1.65	2		4020	feminir	63	19411	175.4	65.6	117	72.5	91	219	60	140	119	0.5903	0.5816
5	1.1-3	1-14 par	1.59	2	40	3730	feminir	17	29327	164.6	70.3	114	75	101	202	74	111	79	0.5747	0.5784
14	6.1-10	n„o	1.58	2	40	3600	feminir	36	27450.22	167.95	66.5	116.5	73.5	81	140	43	79	148	0.579	0.6376
3	1.1-3	n„o	1.53	4	37	3200	mascul	18												
11	1.1-3	n„o	1.58	2		3030	feminir	18	25213.16	168.25	63.3	119.5	78	79	177	55	105	124	0.5653	0.574
4	1.1-3	n„o	1.63	3	39	2800	feminir	78	36526.59	163.75	75.8	103	73	82	156	66	82	62	0.5788	0.5799
8	1.1-3	n„o	1.56	2	38	2560	mascul	26												
3	1 ou -	n„o	1.51	1		2800	feminino		17926.18	158.95	58.2	106	67	79	301	66	210	111	0.5834	0.5782
16	1.1-3	1-14 tod	1.55	1	38	2900	masculin													
12	3.1-6	n„o	1.55	2		3120	mascul	20	19485.55	164.5	75.3	136.5	75	115	220	49	144	88	0.5859	0.5781
17	3.1-6	n„o	1.52	1	41	2960	masculin													
2	1.1-3	n„o	1.6	9	39	4080	mascul	132	16462.88	175	80	117	74	87	162	63	86	69		
5	1.1-3	n„o	1.47	5	41	3280	mascul	16	17889.55	170.85	69.2	101.5	66.5	96	245	52	169	86	0.5779	0.5745
8	1.1-3	1-14 par	1.55	6	39	3800	mascul	57	36614.79	175.65	100.8	140	89	89	142	40	80	132	0.5842	0.5787
5	3.1-6	n„o	1.5	2		3070	feminir	28												
4	1.1-3	1-14 tod	1.52	3	41	3380	feminir	75												
2	1.1-3	1-14 tod	1.58	3	40	2800	mascul	33	30560.39	171.85	93.2	128.5	76.5	85	182	45	104	175	0.5772	0.577
3	1 ou -	1-14 tod	1.51	6	40	2550	feminir	57	12374.57	161.8	49.1	104	69	69	167	58	92	66	0.5771	0.5749
2	1.1-3	n„o	1.58	2	40	2900	mascul	58	20955.82	181.25	82.6	125	69.5	75	145	44	89	75	0.5791	0.5888
5	1.1-3	n„o	1.62	1	40	3470	feminino													
6	1 ou -	n„o	1.52	1		2930	masculin		13469.18	178.45	84.2	127	66.5	86	162	47	88	106	0.5796	0.5756
6	1 ou -	n„o	1.5	1	40	3030	masculin													

pescmae	prenda	pfumomae	paltmae	pgesta	pidgest	ppn	psex	pint	dmgtotdxa	daltura2012	dpeso	dsysmed	ddiamed	dglicose	dcolesterol	dhdl	dldl	dtrig	dECMICE	dECMICD
5	1 ou -	n„o	1.5	6	40	3100	feminir	33												
6	1.1-3	n„o	1.56	1	37	3850	masculin													
5	1.1-3	n„o	1.49	6		3750	feminir	15	32449.25	162.3	72.1	101	71	82	153	54	85	56	0.5652	0.572
1	1.1-3	15 + tod	1.54	5	39	3160	mascul	70	34034.7	177.7	98.8	112	70	87	161	43	99	91	0.5782	0.6058
16	6.1-10	n„o	1.4	3	36	2930	mascul	39	52978.24	160.95	115.8	140.5	83.5	86	148	54	80	45	0.5814	0.5644
5	1.1-3	n„o	1.55	1	41	3340	masculin		22400.15	178.45	76.7	129	79.5	83	160	42	86	189	0.5747	0.5789
9	6.1-10	n„o	1.53	2		3700	feminir	151												
5	1.1-3	15 + tod	1.54	3		2500	feminir	47	24275.97	151.4	62.5	107.5	73.5	87	240	54	159	171	0.5861	0.5599
7	1.1-3	n„o	1.55	2	40	3000	mascul	120	13555.37	171.5	69	119	77	91	151	63	79	69	0.5779	0.5607
9	10	1-14 tod	1.61	1	39	3880	feminino													
4	1.1-3	1-14 par	1.46	5	39	3030	feminir	33	12450.73	159.75	55.3	110	61	105	172	65	92	59	0.5777	0.5778
1	1.1-3	n„o	1.58	1	41	3300	feminino													
4	1.1-3	n„o	1.53	1	39	2950	feminino			155		123	88	98	217	56	140	108	0.5972	0.5772
8	6.1-10	n„o	1.58	1	40	3100	feminino		29736.82	160.25	71.1	112.5	69.5	70	208	43	148	84	0.5804	0.584
8	1.1-3	n„o	1.51	1	41	3250	feminino		27133.65	165.8	70.8	114	82	92	195	79	98	62	0.5827	0.566
7	1.1-3	n„o	1.63	1		2950	masculin		19475.8	168.65	71.3	122	80	88	219	51	155	68	0.5967	0.6308
11	6.1-10	n„o	1.6	1	41	3600	masculin		31165.55	174.8	99.6	166	97.5	82	225	57	142	127		0.5843
5	1.1-3	15 + tod	1.64	2	36	3030	mascul	74	6438.051	184.3	67.4	114	78.5	98	179	66	99	64	0.5753	0.5774
7	1.1-3	1-14 tod	1.59	3	38	3300	mascul	12												
4	1.1-3	n„o	1.54	2	40	3500	feminir	69	20328.61	153.3	55.2	94	63.5	71	165	74	78	94	0.5714	0.5727
1	1.1-3	15 + tod	1.51	7		2730	feminir	72	38280.14	157.35	78.4	109	76	86	228	71	137	130	0.6137	0.5824
3	1 ou -	n„o	1.55	1	38	3500	masculin													
9	1.1-3	n„o	1.54	2	38	3450	mascul	22												
16	6.1-10	n„o	1.44	4	33	2350	feminir	10	19242.52	145.05	50.6	120	83.5	92	197	72	115	75	0.5771	0.575
11	6.1-10	n„o	1.55	1		1680	feminino													
1	1 ou -	n„o	1.5	2		3290	feminir	47	24524.35	157.4	66.7	104.5	72.5	108	159	34	117	47	0.5765	0.5875
4	3.1-6	n„o	1.48	2	42	2980	feminir	54	24240.22	158.35	66.6	118	81	119	166	42	103	89	0.5715	0.5762
2	1.1-3	n„o	1.57	2	39	3050	mascul	65												
9	1.1-3	1-14 par	1.7	2	41	3650	mascul	25	29831.05	174.6	89.6	129.5	83.5	105	200	75	101	143	0.5707	0.579
3	1.1-3	1-14 tod	1.5	2	39	2850	feminir	247	29086.31	161.65	71.9	114.5	76.5	115	179	65	90	161		
9	6.1-10	15 + tod	1.65	3		2250	feminir	114	14952.6	149.8	46.7	137.5	84	95	224	59	140	167	0.5781	0.5776
8	3.1-6	n„o	1.6	3		4750	mascul	11												
5	3.1-6	n„o	1.6	1		3930	masculin													
4	1.1-3	n„o	1.54	5	42	3100	feminir	54	12677.71	160.05	46.6	117	76	72	174	67	95	52	0.5772	0.5739
18	6.1-10	n„o	1.6	2		4150	mascul	18	19673.08	175.8	76	118.5	67.5	89	219	53	132	166	0.5745	0.5789
12	3.1-6	1-14 tod	1.55	2	39	2270	mascul	129												
4	1 ou -	1-14 tod	1.51	8	36	2600	feminir	53	17629.79	155.55	51.4	103	67	84	180	72	91	45		0.55
4	1.1-3	n„o	1.57	2	41	3840	feminir	16	55072.87	169.35	97.6	111.5	74	92	215	81	117	88	0.5781	0.5784
5	1.1-3	n„o	1.61	2	36	2850	feminir	62	12539.5	166.2	54.2	109.5	70	78	138	56	65	69	0.5782	0.5782
4	3.1-6	n„o	1.61	7	40	3260	mascul	144												
3	1.1-3	1-14 tod	1.61	1	38	2350	masculin													
2	3.1-6	n„o		4	40	4000	feminir	132												
11	6.1-10	n„o	1.57	3	40	3700	feminir	56												
5	1.1-3	n„o	1.56	6		4920	feminir	25												
1	1 ou -	n„o	1.46	3	39	2500	feminir	57												
7	1.1-3	1-14 tod	1.46	1	40	2900	masculin													
16	3.1-6	n„o	1.66	1	40	3200	feminino		30700.79	178.35	78.9	120	77	70	160	60	80	86		
7	3.1-6	n„o	1.65	1	38	2750	masculin													

pescmae	prenda	pfumomae	paltmae	pgesta	pidgest	ppn	psex	pint	dmgtotdxa	daltura2012	dpeso	dsysmed	ddiamed	dglicose	dcolesterol	dhdl	dldl	dtrig	dECMICE	dECMICD
7	1.1-3	1-14 tod	1.53	1	38	3220	masculin		17330.9	182.5	80.4	114.5	68	113	140	40	73	242	0.5968	0.5803
12	6.1-10	n„o	1.48	2	42	3100	feminir	20												
11	1.1-3	n„o	1.64	2		3100	feminir	9	16812.34	163.3	61	110.5	74	78	142	46	85	40	0.5717	0.5788
16	10	n„o	1.58	2	38	3100	feminir	36	33565.27	159.5	77.6	110	70	116	240	98	113	179	0.5783	0.5836
12	6.1-10	n„o	1.59	1	42	3550	feminino		16773.85	166.3	59.6	118.5	74.5	82	218	67	141	76	0.578	0.5741
2	1.1-3	n„o	1.58	3		3150	mascul	47	2166.957	177.2	53.9	126	75.5	65	158	53	93	61	0.577	0.5774
3	1.1-3	n„o	1.6	1	38	3700	masculin		32541.13	190	108.1	132	83	95	203	47	110	255	0.5836	0.5797
8	1.1-3	n„o	1.59	4	40	3160	mascul	19												
1	1.1-3	n„o	1.52	1	39	3050	feminino		13598.17	163.8	58.6	112	71	82	173	86	78	52	0.5804	0.579
5	1.1-3	n„o	1.55	3	39	3400	mascul	38	28765.34	168.5	89.6	136	86	93	146	44	81	87	0.5741	
2	1.1-3	n„o	1.68	4	39	3200	mascul	22	3260.671	177.55	59.4	133	84	90	185	62	105	62	0.578	
