# Supplementary material for: Association between Birth Interval and Cardiovascular Outcomes at 30 Years of Age: A Prospective Cohort Study from Brazil
Source: PLoS One. 2016 Feb 18;11(2):e0149054. doi: 10.1371/journal.pone.0149054 (PMC4758625; doi:10.1371/journal.pone.0149054)
Supplement: S2 File — Regression coefficients (95% CI) for birth interval, as a continuous outcome variable, and confounding variables (Table A). Regression coefficients (95% CI) for cardiovascular risk factors at age 30 years and confounding variables (Table B). Maternal and Offspring Characteristics averages (mean or median) at 30 years, Stratified by Birth Interval and by gender (Table C). (DOCX) [file pone.0149054.s002.docx]

**Association Between Birth Interval and Cardiovascular Outcomes at 30 years of Age: a Prospective Cohort Study from Brazil**

D Devakumar, PC Hallal, BL Horta, FC Barros, JCK Wells

**Table A. Regression coefficients (95% CI) for birth interval, as a continuous outcome variable, and confounding variables**

|  | Maternal age at delivery (years) | Maternal education (years) | Family income (multiples of 1982 minimum wage) | Maternal BMI at beginning of pregnancy (kg/m^2^) | Birth order |
| --- | --- | --- | --- | --- | --- |
| Birth interval (months) | 2.717 (2.550, 2.884) | -2.070 (-3.333, -0.807) | 2.092 (1.058, 3.125) | 2.102 (1.796, 2.407) | 1.255 (0.705, 1.806) |

**Table B. Regression coefficients (95% CI) for cardiovascular risk factors at age 30 years and confounding variables**

|  | Maternal age at delivery (years) | Maternal education (years) | Family income (multiples of 1982 minimum wage) | Maternal BMI at beginning of pregnancy (kg/m^2^) | Birth order |
| --- | --- | --- | --- | --- | --- |
| Height (cm) | 0.043 (-0.005, 0.092) | 1.278 (0.920, 1.635) | 1.026 (0.731, 1.320) | 0.040 (-0.047, 0.127) | -0.231 (-0.381, -0.081) |
| Weight (kg) | 0.012 (-0.082, 0.106) | 0.926 (0.230, 1.621) | 0.709 (0.135, 1.283) | 0.975 (0.810, 1.139) | -0.370 (-0.659, -0.080) |
| Body mass index (kg/m^2^) | -0.010 (-0.040, 0.019) | -0.125 (-0.343, 0.094) | -0.107 (-0.287, 0.073) | 0.336 (0.286, 0.387) | -0.054 (-0.145, 0.037) |
| Fat-free mass (kg) | -0.014 (-0.075, 0.046) | 0.148 (-0.300, 0.595) | 0.131 (-0.237, 0.500) | 0.220 (0.111, 0.329) | -0.127 (-0.313, 0.059) |
| Fat mass (kg) | 0.055 (-0.006, 0.115) | 0.638 (0.189, 1.086) | 0.524 (0.155, 0.893) | 0.588 (0.482, 0.695) | -0.232 (-0.418, -0.045) |
| Visceral fat (cm) | -0.007 (-0.018, 0.005) | -0.147 (-0.230, -0.063) | -0.147 (-0.216, -0.078) | 0.050 (0.030, 0.070) | 0.012 (-0.023, 0.047) |
| Subcutaneous fat (cm) | 0.005 (-0.001, 0.011) | 0.042 (-0.002, 0.086) | 0.033 (-0.003, 0.070) | 0.055 (0.045, 0.065) | -0.017 (-0.036, 0.001) |
| Systolic blood pressure (mmHg) | -0.088 (-0.161, -0.016) | 0.227 (-0.311, 0.766) | -0.285 (-0.729, 0.159) | 0.167 (0.038, 0.296) | -0.344 (-0.569, -0.119) |
| Diastolic blood pressure (mmHg) | -0.063 (-0.112, -0.014) | 0.189 (-0.174, 0.553) | -0.273 (-0.573, 0.027) | 0.167 (0.079, 0.255) | -0.233 (-0.385, -0.081) |
| Left carotid thickness (mm)  multiplied by 1000 | -0.025 (-0.159, 0.109) | -0.728 (-1.721, 0.265) | -0.941(-1.753, -0.129) | 0.477 (0.244, 0.709) | -0.062 (-0.473, 0.167) |
| Right carotid thickness (mm) multiplied by 1000 | -0.017 (-0.137, 0.104) | -0.377 (-1.265, 0.512) | -0.279 (-1.001, 0.444) | 0.300 (0.091, 0.510) | -0.202 (-0.570, 0.167) |
| Ln Glucose (mg/dL) | -0.001 (-0.002, 0.000) | -0.002 (-0.010, 0.006) | -0.007 (-0.014, -0.001) | 0.000 (-0.001, 0.002) | -0.000 (-0.004, 0.003) |
| Cholesterol (mmol/L) | -0.016 (-0.218, 0.187) | 2.536 (1.032, 4.040) | 1.851 (0.607, 3.094) | 0.222 (-0.140, 0.583) | -0.677 (-1.302, -0.051) |
| High density lipoprotein (mmol/L) | 0.085 (0.011, 0.159) | 1.501 (0.954, 2.047) | 1.614 (1.164, 2.065) | -0.065 (-0.199, 0.068) | -0.136 (-0.364, 0.092) |
| Low density lipoprotein (mmol/L) | -0.096 (-0.251, 0.058) | 0.255 (-0.892, 1.403) | 0.072 (-0.876, 1.020) | 0.188 (-0.085, 0.460) | -0.464(-0.940, 0.013) |
| Ln Triglycerides (mmol/L) | -0.001 (-0.004, 0.002) | 0.042 (0.020, 0.064) | 0.022 (0.004, 0.041) | 0.003 (-0.002, 0.008) | -0.009 (-0.018, 0.001) |

**Table C. Maternal and Offspring Characteristics averages (mean or median) at 30 years, Stratified by Birth Interval and by gender**

|  | Birth interval |  |  |  |  |  |
| --- | --- | --- | --- | --- | --- | --- |
|  | <18 months | 18 to <24 months | 24 to <36 months | 36 to <48 months | 48 to <71 months | >71 months |
| **30 year outcomes** |  |  |  |  |  |  |
| Height (cm) | Males: 173.4 | Males: 173.9 | Males: 175.1 | Males: 174.0 | Males: 173.7 | Males: 174.2 |
|  | Females: 160.5 | Females: 161.8 | Females: 161.3 | Females: 162.1 | Females: 161.4 | Females: 161.8 |
| Weight (kg) | Males: 81.0 | Males: 81.6 | Males: 81.8 | Males: 81.8 | Males: 80.6 | Males: 82.7 |
|  | Females: 68.0 | Females: 68.1 | Females: 68.9 | Females: 69.1 | Females: 69.3 | Females: 72.0 |
| Body mass index (kg/m^2^) | Males: 26.9 | Males: 26.9 | Males: 26.6 | Males: 26.9 | Males: 26.7 | Males: 27.2 |
|  | Females: 26.4 | Females: 26.0 | Females: 26.5 | Females: 26.2 | Females: 26.5 | Females: 27.6 |
| Fat-free mass (dexa) | Males: 59.6 | Males: 59.7 | Males: 60.8 | Males: 60.0 | Males: 59.9 | Males: 59.2 |
|  | Females: 40.9 | Females: 41.2 | Females: 41.6 | Females: 41.8 | Females: 41.4 | Females: 42.0 |
| Fat mass (kg) | Males: 18.8 | Males: 18.9 | Males: 19.5 | Males: 20.2 | Males: 19.9 | Males: 22.0 |
|  | Females: 26.3 | Females: 26.9 | Females: 27.4 | Females: 26.9 | Females: 27.8 | Females: 29.2 |
| Visceral fat (cm) median | Males: 6.9 | Males: 6.7 | Males: 6.6 | Males: 6.6 | Males: 6.7 | Males: 6.4 |
|  | Females: 4.6 | Females: 4.6 | Females: 4.7 | Females: 4.6 | Females: 4.7 | Females: 4.9 |
| Subcutaneous fat (cm) median | Males: 1.7 | Males: 1.7 | Males: 1.7 | Males: 1.8 | Males: 1.7 | Males: 1.9 |
|  | Females: 2.2 | Females: 2.2 | Females: 2.4 | Females: 2.4 | Females: 2.4 | Females: 2.5 |
| Systolic blood pressure (mmHg) | Males: 127.3 | Males: 128.2 | Males: 128.5 | Males: 127.7 | Males: 127.9 | Males: 126.9 |
|  | Females: 114.6 | Females: 113.0 | Females: 114.3 | Females: 113.7 | Females: 114.0 | Females: 114.4 |
| Diastolic blood pressure (mmHg) | Males: 76.2 | Males: 76.5 | Males: 76.9 | Males: 75.8 | Males: 77.6 | Males: 77.0 |
|  | Females: 73.6 | Females: 72.5 | Females: 73.6 | Females: 72.8 | Females: 73.8 | Females: 73.8 |
| Left carotid thickness (mm) | Males: 0.59 | Males: 0.58 | Males: 0.59 | Males: 0.59 | Males: 0.59 | Males: 0.59 |
|  | Females: 0.58 | Females: 0.58 | Females: 0.58 | Females: 0.58 | Females: 0.58 | Females: 0.58 |
| Right carotid thickness (mm) | Males: 0.59 | Males: 0.58 | Males: 0.58 | Males: 0.59 | Males: 0.58 | Males: 0.58 |
|  | Females: 0.58 | Females: 0.58 | Females: 0.58 | Females: 0.58 | Females: 0.58 | Females: 0.58 |
| Glucose (mg/dL) | Males: 91.9 | Males: 90.2 | Males: 95.4 | Males: 95.3 | Males: 90.5 | Males: 94.9 |
|  | Females: 85.7 | Females: 88.1 | Females: 84.4 | Females: 85.4 | Females: 85.3 | Females: 87.0 |
| Cholesterol (mmol/L) | Males: 194.9 | Males: 188.3 | Males: 193.1 | Males: 189.3 | Males: 191.4 | Males: 194.1 |
|  | Females: 184.1 | Females: 183.1 | Females: 189.7 | Females: 189.3 | Females: 189.1 | Females: 192.0 |
| High density lipoprotein (mmol/L) | Males: 54.5 | Males: 55.0 | Males: 53.1 | Males: 53.0 | Males: 53.5 | Males: 54.3 |
|  | Females: 62.4 | Females: 62.9 | Females: 62.8 | Females: 63.6 | Females: 62.9 | Females: 62.8 |
| Low density lipoprotein (mmol/L) | Males: 115.0 | Males: 108.9 | Males: 111.0 | Males: 108.8 | Males: 110.6 | Males: 111.0 |
|  | Females: 102.5 | Females: 101.6 | Females: 107.6 | Females: 106.4 | Females: 106.4 | Females: 109.6 |
| Triglycerides (mmol/L) median | Males: 106 | Males: 91 | Males: 112 | Males: 99 | Males: 113 | Males: 99 |
|  | Females: 79 | Females: 80 | Females: 86 | Females: 87 | Females: 91 | Females: 91 |
